# Transcriptional profiling reveals the expression of novel genes in response to various stimuli in the human dermatophyte *Trichophyton rubrum*

**DOI:** 10.1186/1471-2180-10-39

**Published:** 2010-02-08

**Authors:** Nalu TA Peres, Pablo R Sanches, Juliana P Falcão, Henrique CS Silveira, Fernanda G Paião, Fernanda CA Maranhão, Diana E Gras, Fernando Segato, Rodrigo A Cazzaniga, Mendelson Mazucato, Jeny R Cursino-Santos, Roseli Aquino-Ferreira, Antonio Rossi, Nilce M Martinez-Rossi

**Affiliations:** 1 Departamento de Genética, Faculdade de Medicina de Ribeirão Preto, Universidade de São Paulo, Ribeirão Preto 14049-900, SP, Brazil; 2 Departamento de Bioquímica e Imunologia, Faculdade de Medicina de Ribeirão Preto, Universidade de São Paulo, Ribeirão Preto 14049-900, SP, Brazil; 3 Current address: Faculdade de Ciências Farmacêuticas de Ribeirão Preto, Universidade de São Paulo, 14040-903, Ribeirão Preto, SP, Brazil

## Abstract

**Background:**

Cutaneous mycoses are common human infections among healthy and immunocompromised hosts, and the anthropophilic fungus *Trichophyton rubrum *is the most prevalent microorganism isolated from such clinical cases worldwide. The aim of this study was to determine the transcriptional profile of *T. rubrum *exposed to various stimuli in order to obtain insights into the responses of this pathogen to different environmental challenges. Therefore, we generated an expressed sequence tag (EST) collection by constructing one cDNA library and nine suppression subtractive hybridization libraries.

**Results:**

The 1388 unigenes identified in this study were functionally classified based on the Munich Information Center for Protein Sequences (MIPS) categories. The identified proteins were involved in transcriptional regulation, cellular defense and stress, protein degradation, signaling, transport, and secretion, among other functions. Analysis of these unigenes revealed 575 *T. rubrum *sequences that had not been previously deposited in public databases.

**Conclusion:**

In this study, we identified novel *T. rubrum *genes that will be useful for ORF prediction in genome sequencing and facilitating functional genome analysis. Annotation of these expressed genes revealed metabolic adaptations of *T. rubrum *to carbon sources, ambient pH shifts, and various antifungal drugs used in medical practice. Furthermore, challenging *T. rubrum *with cytotoxic drugs and ambient pH shifts extended our understanding of the molecular events possibly involved in the infectious process and resistance to antifungal drugs.

## Background

*Trichophyton rubrum *is a cosmopolitan dermatophyte that colonizes human skin and nails and is the most prevalent cause of human dermatophytoses [1,2]. During the initial stages of the infection, dermatophytes induce the expression of adhesins and unspecific proteases and keratinases that have optimum activity at acidic pH values [3], which is probably because the human skin has an acidic pH value [4]. The secretion of these proteases, which have been identified as an important step in fungal pathogenicity and virulence [5,6], act on keratinous and nonkeratinous substrates to release peptides that are further hydrolyzed to amino acids by putative peptidases. The metabolism of some amino acids shifts the extracellular pH from acidic to alkaline values at which most known keratinolytic proteases have optimal enzymatic activity [7-9]. *T. rubrum *also responds to the environmental pH by altering its gene expression profile [9,10].

Molecular studies have been performed with human pathogens such as *Candida albicans*, *Histoplasma capsulatum*, and *Paracoccidioides brasiliensis*, and the results thus obtained have helped to determine the fungal transcriptional profile and characterize the genes involved in host-pathogen interactions and environmental stress responses [11-13]. Previously, a collection of *T. rubrum *expressed sequence tags (ESTs) was obtained from distinct developmental phases [14,15]. However, determining the transcriptional profiles in response to different cell stimuli is necessary for extending our understanding of diverse cellular events, and the results from such studies may reveal new signal transduction networks and the activation of specific metabolic pathways. Functional analysis of the genes involved in these molecular events will help in evaluating their roles as putative cellular targets in the development of new antifungal agents.

Our study aimed to extend the *T. rubrum *genomic database by adding expressed gene resources that cover different aspects of cellular metabolism. Moreover, the data can help to generate useful information to screen valuable genes for functional and postgenomic analyses. The EST collection described here revealed the metabolic adaptations of the human pathogen *T. rubrum *to changes in the ambient pH and carbon sources and also provided information on the adaptive responses to several cytotoxic drugs.

## Results and Discussion

The EST collection described here was obtained from a cDNA library and nine independent suppression subtractive hybridization (SSH) libraries. A total of 2735 high-quality sequences with an average sequence length of 339 nucleotides were generated. These ESTs were assembled in 296 contigs and 1092 singletons, resulting in 1388 unique sequences with a redundancy of 49.3% (Table 1). The majority of the contigs assembled ESTs from a maximum of four libraries, suggesting that these genes are expressed under environmental stress or specific growth conditions. The search results and GenBank submission numbers for each EST are shown in Additional file 1. Analysis of these 1388 unigenes revealed 666 sequences that had no similarity to the sequences in the GenBank dbEST, which contains 37890 *T. rubrum *sequences. Of the 666 sequences, 404 had no similarities to the sequences in the nonredundant database (Table 1). Additional analysis revealed that of the 666 sequences, 91 were present in the *Tr*ED database [16]. Thus, 575 novel genes were identified, representing a marked increase in the number of expressed genes identified in the dermatophyte *T. rubrum*. These genes and the corresponding libraries in which they were identified are highlighted in Additional file 2.

**Table 1:** General features of *T. rubrum *EST libraries

Library	GenBank accession No.	No. of raw ESTs	No. of contigs	No. of singletons	Unique genes	No. of unigenes matching GenBank database (NR)^(a)^	No. of unigenes without match to GenBank dbEST database^(b)^	
							
							matching GenBank database (NR)^(c)^	without match to GenBank database (NR)
Total	FE524602-FE527336	2735	296	1092	1388	681 (49.1%)	262 (18.9%)	404 (29.1%)
1	FE524602-FE525578	977	75	545	620	235 (37.9%)	73 (11.8%)	207 (33.4%)
2	FE525579-FE525681	103	23	14	37	24 (64.9%)	18 (48.6%)	10 (27.0%)
3	FE525682-FE525782	101	7	76	83	46 (55.4%)	19 (22.9%)	20 (24.1%)
4	FE525783-FE526029	247	64	56	120	62 (51.7%)	31 (25.8%)	36 (30.0%)
5	FE526030-FE526148	119	7	50	57	26 (45.6%)	7 (12.3%)	17 (29.8%)
6	FE526149-FE526246	98	12	5	17	11 (64.7%)	5 (29.4%)	3 (17.6%)
7	FE526247-FE526554	308	36	59	95	69 (72.6%)	25 (26.3%)	17 (17.9%)
8	FE526555-FE526754	200	30	18	48	27 (56.3%)	21 (43.8%)	15 (31.3%)
9	FE526755-FE527126 and FG235008-FG235038	372	43	248	291	162 (55.7%)	53 (18.2%)	74 (25.4%)
10	FE527127-FE527336	210	26	143	169	106 (62.7%)	34 (20.1%)	23 (13.6%)

^(a) ^Unigenes with similarity to the sequences in the nonredundant NCBI database (1e-3) using BLASTx.

^(b) ^Unigenes with no similarity to the sequences in the dbEST-NCBI database (1e-20) using BLASTn-Organism: *Trichophyton rubrum *(taxid:5551).

^(c) ^*T. rubrum *protein sequences identified in this database were excluded from this analysis.

**Figure 1:**
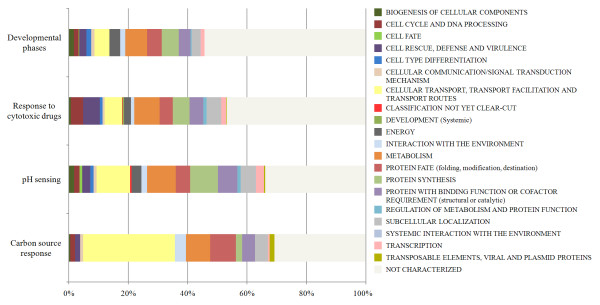
***T. rubrum *unigenes functional categorization, according to MIPS**. The unigenes were grouped in four different stimuli.

The 1388 unigenes identified in this study were functionally classified based on the Munich Information Center for Protein Sequences (MIPS) categories. The classification led to the identification of putative proteins involved in transcriptional regulation, cellular defense and stress, protein degradation, signaling, transport, and secretion, among other functions (Additional file 2). However, many of these unigenes (54.28% involved in the developmental phases, 46.87% in exposure to cytotoxic drugs, 33.93% in pH sensing, and 30.81% in carbon source responses) were not classified by the MIPS annotation (Fig. 1). Growth of *T. rubrum *in a keratinocyte serum-free medium (Library 1) revealed 207 novel genes (Table 1; Additional file 2) in comparison to the *T. rubrum *sequences deposited in public databases, which include an EST collection that was previously generated during the growth of *T. rubrum *in Sabouraud liquid medium [14]. This suggests that the expression of these 207 novel genes is nutrient-dependent. Functional grouping of these genes, which were identified on the basis of their ESTs, revealed their possible involvement in various cellular processes such as basic metabolism, conidial germination, and hyphal growth, among other functions (see Additional file 2).

### Challenging *T. rubrum* with cytotoxic drugs

Numerous signal-transduction pathways are used by fungi to sense and overcome the toxic effects of antifungal drugs [17]. Our aim in this study was to identify metabolic events that occur during the initial stages of drug exposure; therefore, we created an EST collection by challenging the dermatophyte *T. rubrum *with cytotoxic drugs, including most of the antifungals used in medical practice. These drugs, which belong to the azole and allylamine/thiocarbamate classes, were fluconazole (FLC), imazalil (IMZ), itraconazole (ITRA), ketoconazole (KTC), tioconazole (TIO), and terbinafine (TRB). All of these compounds inhibit the biosynthesis of ergosterol. *T. rubrum *was also challenged with the following cytotoxic drugs: amphotericin B (AMB), griseofulvin (GRS), benomyl (BEN), undecanoic acid (UDA), cycloheximide (CHX), chloramphenicol (CAP), acriflavin (ACR), ethidium bromide (EB), and 4-nitroquinoline 1-oxide (4NQO) [18-20]. Approximately 300 unigenes were identified in these experiments and only 70 of these were exclusive to drug challenge (Additional file 2). Drug exposure induced the transcription of several multidrug resistance genes, as previously reported in studies in which *T. rubrum *was exposed to sub-inhibitory levels of KTC, AMB, or other drugs [21,22]. One of these genes [GenBank: FE526598] encodes a putative multidrug resistance protein (MDR) that accumulates in the mycelia when the organism is independently exposed to various cytotoxic agents. Overexpression of this gene has been previously reported in the myceliaof *T. rubrum *exposed to the antimycotic agents ACR, GRS, ITRA, or FLC [23]. Disruption of this gene increased the susceptibility of the mutant strain to TRB in comparison with the control, suggesting that this transporter modulates *T. rubrum *drug susceptibility [23].

Some of the ESTs that were overexpressed in the mycelia of *T. rubrum *upon exposure to cytotoxic drugs showed similarities to genes encoding the Pol protein [GenBank: FE526593], carboxylic ester hydrolase [GenBank: FE525618], DNA mismatch repair protein [GenBank: FE525624], and cooper resistance-associated P-type ATPase protein [GenBank: FE526224], among others. These results had been previously validated by northern blot analyses in mycelia of *T. rubrum *grown in the presence of TRB or GRS [20]. Upregulation of ESTs similar to the *pol *gene of the *Cg*ret retrotransposon element from *Glomerella cingulata *(anamorph: *Colletotrichum gloeosporioides*) suggests that *T. rubrum *evinces an adaptive response to environmental stress. Interestingly, overexpression of this gene was also observed in mycelia of *T. rubrum *grown in keratin as the carbon source (Additional file 2), which suggests the involvement of this retrotransposon in nonspecific responses, leading to stress adaptation.

Overexpression of an EST encoding salicylate 1-monooxigenase, a naphthalene-degrading enzyme [GenBank: FE525605] (Additional file 2), was exclusive to *T. rubrum *that had been challenged with cytotoxic drugs, including TRB (Library 2). A possible mechanism of resistance to TRB was evidenced in the model fungus *Aspergillus nidulans *and involved the overexpression of the salicylate 1-monooxigenase gene *salA*, probably due to a multicopy effect [24]. Moreover, plasmids carrying the *salA *gene of *A. nidulans *were able to transform a *T. rubrum *strain from TRB-sensitive to TRB-resistant, suggesting that a similar resistance mechanism could help *T. rubrum *to overcome the inhibitory effect of TRB, which has a naphthalene nucleus present in its molecular structure (not shown).

### pH and carbon source signaling

Among the most important virulence factors identified in dermatophytes are proteases that have optimal activity at acidic pH and are secreted during the initial stages of fungal infection [3,25,26]. The hydrolysis of skin proteins releases amino acids such as glycine, the metabolism of which shifts the extracellular pH from acidic to alkaline values [8]. This effect is required for the growth and maintenance of the dermatophyte in the host [7-9,27]. Therefore, identification of *T. rubrum *genes that are differentially expressed in response to shifts in the ambient pH provides useful information on pH sensing during host infection. When the media was supplemented with glucose as the carbon source, we identified 339 genes that were overexpressed at pH 5.0 and 169 genes that were overexpressed in response to alkaline pH conditions (Additional file 2). Functional grouping of these ESTs led to the identification of genes involved in various cellular processes, such as membrane remodeling, cellular transport, iron uptake, defense, metabolism, signal transduction, and virulence.

Interestingly, the transcription of the gene encoding an acetamidase [GenBank: FE526983] was stimulated in an acidic milieu (Additional file 2). This enzyme hydrolyses acetamide, releasing acetate and ammonia. Acetate is metabolized to acetyl-CoA by acetyl-CoA synthase, with the concomitant secretion of ammonia, which raises the extracellular pH to alkaline values [28-30]. The metabolism of amino acids that generate cytoplasmic acetyl-CoA shifts the extracellular pH from acidic to alkaline values [31], an effectobserved in *in vitro *cultures of *T. rubrum *[8]. The metalloenzyme urease (the *T. rubrum *urease gene [GenBank: FE526454] was identified in our unigenesdatabase) catalyzes the hydrolysis of urea to ammonia during the parasitic cycle of *Coccidioides posadasii *and also creates an alkaline microenvironment at the infection site. Ammonia secretion contributes to host tissue damage, thereby enhancing the virulence of this human respiratory pathogen [32] (Table 2).

**Table 2:** Putative proteins required for fungal virulence.

Accession no. of one EST	Library	Virulence determinant	Function in fungi	Reference number
FE526884	9	isocitrate lyase	Glyoxylate cycle enzyme	[43,44]
FE525405	1	malate synthase	Glyoxylate cycle enzyme	[43,44]
FE525119	1	citrate synthase	Glyoxylate cycle enzyme	[43,44]
FE526004	4	phospholipase B	Gene inactivation attenuates virulence in *Cryptococcus neoformans *and *Candida albicans*	[63,64]
FE526464	7	subtilisin-like protease Sub3	Sub3 is a dominant protease secreted by *Trichophyton rubrum *during host infection	[65]
FE526467	1, 7, 10	subtilisin-like protease Sub5	Putative *Trichophyton rubrum *virulence factor	[9]
FE526356	7	metalloprotease Mep3	MEP3 is produced by *M. canis *during guinea pigs infection	[66]
FE526553	7	metalloprotease Mep4	Mep4 is a dominant protease secreted by *Trichophyton rubrum *during host infection	[65]
FE526905	9	carboxypeptidase	Important for the assimilation of nitrogenous substrates during infection and contributes to the virulence of dermatophytes	[50]
FE524895	1	dipeptidyl-peptidase V	Dipeptidyl peptidases as potential virulence factors for *Microsporum canis*	[67]
FE526224	2, 7, 8	copper resistance-associated P-type ATPase	Cu-ATPase mutants showed reduced virulence in *Listeria monocytogenes *and *Criptococcus neoformans*	[52,53,68]
FE526598	2, 7, 8	*Tru*MDR2	Gene inactivation attenuates the virulence of *Trichophyton rubrum in vitro*	[40]
FE525063	1	mannosyltransferase	Gene inactivation attenuates the virulence of *Candida albicans *and *Aspergillus fumigatus*	[69,70]
FE526454	7	urease	Gene inactivation reduces the amount of ammonia secreted *in vitro *and attenuates the virulence of *Coccidioides posadasii*	[32]
FE526352	1, 7	glucosamine-6-phosphate deaminase	Gene inactivation attenuates the virulence of *Candida albicans *in a murine model	[71]
FE524999	1	glyceraldehyde-3-phosphate dehydrogenase (GAPDH)	GAPDH contributes to the adhesion of *Paracoccidioides brasiliensis *to host tissues and to the dissemination of infection.	[72]
FE527290	10	thioredoxin TrxA	Putative *Trichophyton mentagrophytes *virulence factor	[73]

The overexpression of the ESTs from SSH libraries was confirmed by reverse Northern hybridization and/or Northern blot.

**Figure 2:**
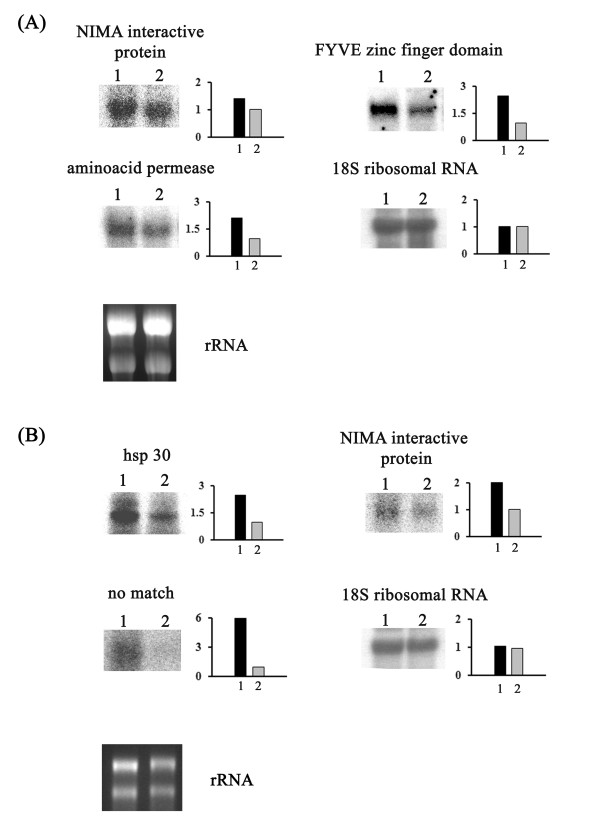
**Northern blot analysis of transcripts using total RNA**. (A) Overexpression of genes encoding the NIMA interactive protein [GenBank: FE526568], FYVE protein [GenBank: FE526741], and aminoacid permease [GenBank: FE526515] in *T. rubrum *mycelia exposed to acidic pH for 30 min (Library 8). Lanes 1 and 2 represent the H6 strain incubated at pH 5.0 and pH 8.0 (control), respectively. (B)Overexpression of genes encoding hs p30 [GenBank: FE526362], NIMA interactive protein [GenBank: FE526568], and a no-match transcript [GenBank: FE526434] in *T. rubrum *grown in keratin for 72 h (Library 7). Lanes 1 and 2 represent the H6 strain cultured with keratin or glucose (control) as the carbon source, respectively. Ethidium-bromide-stained rRNA bands are shown to allow comparison of the quantities of loaded RNAs. Hybridization with the 18S rRNA gene was performed as an additional loading control for northern blots. Bars show fold expression, determined from the intensity measured by densitometric analysis.

Overexpression of some genes under acidic pH conditions was confirmed by northern blot analysis (Fig. 2A). One of these encodes a protein carrying the FYVE zinc finger domain [GenBank: FE526741]. FYVE domains are found in several eukaryotic nonnuclear proteins that are involved in many cellular functions, including cytoskeletal regulation, signal transduction, and vesicle transport [33,34]. Most of the proteins that carry the FYVE domain function in the recruitment of cytosolic proteins by binding to phosphatidylinositol 3-phosphate, which is mainly found in the endosome and functions as a regulator of endocytic membrane trafficking [35]. Interestingly, the anchoring of FYVE proteins to phosphatidylinositol 3-phosphate-enriched membranes is strongly pH-dependent and is enhanced by an acidic cytosolic environment [36,37]. A relevant gene that is overexpressed at alkaline pH values encodes an iron-sulfur cluster protein [GenBank: FE527227], a cofactor for several proteins involved in electron transfer in redox and nonredox catalysis, in gene regulation, and as sensors of oxygen and iron [38]. Some genes involved in the acquisition of iron by *C. albicans *are also overexpressed at pH 8.0, suggesting that alkaline pH induces iron starvation [39]. Thus, genes overexpressed at either acidic or alkaline pH values are probably involved in the initial stages of dermatophyte infection and maintenance in the host tissue, respectively.

Identification of the ESTs involved in keratin metabolism may also help in determining the genes necessary for installation and maintenance of the pathogen in the host. We identified 95 keratin-enriched transcripts, and 17 ESTs which were involved in glucose metabolism (Table 1; Additional file 2). It was previously observed that the pH of the medium remained at a value of approximately 5.0 during mycelial growth when glucose was the carbon source. In contrast, during growth on keratin or a mixture of glycine and glycerol, the extracellular pH shifted from acidic to alkaline, reaching pH values from 8.3 to 8.9 [8,9]. Growth on keratin at alkaline pH values revealed the overexpression of several proteases and membrane transporter protein genes (Additional file 2) such as subtilisin protease SUB 5 [GenBank: FE526467], metalloprotease Mep3 [GenBank: FE526356], MFS oligopeptide transporter [GenBank:FE526458], MDR protein [GenBank: FE526598], Cu^2+^-ATPase [GenBank: FE526224], V-type ATPase, subunit B [GenBank: FE526350], and an aminoacid permease [GenBank: FE526515] [9,40]. Most of these genes were not overexpressed when the initial culture pH was adjusted to 8.0 and glucose was used as the carbon source (Library 10) (Additional file 2). This suggests that a combination of an ambient pH shift and keratin as the carbon source is necessary to induce the expression of these genes. Interestingly, the gene encoding NIMA interactive protein [GenBank: FE526568] was overexpressed in keratin cultures, in response to cytotoxic drugs, and after mycelial exposure for 30 min at pH 5.0, suggesting that this gene may be involved in unspecific stress responses. Overexpression of the NIMA interactive protein gene in mycelia of *T. rubrum *exposed to acid pH (Fig. 2A) or grown in keratin as the only carbon source (Fig. 2B) was confirmed by northern blot analysis. In fact, this protein is a member of the NIMA family of kinases and is expressed in response to unspecific cellular stresses [41]. Furthermore, the *hsp30 *gene [GenBank: FE526362] and a transcript with no significant similarity [GenBank: FE526434] were confirmed to be overexpressed when keratin was used as the carbon source (Fig. 2B). The *HSP30 *gene of *Saccharomyces cerevisiae *is strongly induced when the fungus is exposed to various stresses, including heat shock and glucose starvation [42]. Similar to many other heat shock proteins, HSP30 increases cellular tolerance to stress.

### Genes that contribute to virulence

The ESTs shown in Table 2 reveal *T. rubrum *genes that encode putative proteins similar to the virulence factors identified in other fungi. Three of the five glyoxylate cycle enzymes were identified in our EST database, i.e., isocitrate lyase and malate synthase, which are key enzymes of this cycle, together with citrate synthase. The glyoxylate cycle is required for the full virulence of *C. albicans *[43], *Mycobacterium tuberculosis *[44,45], and *P. brasiliensis *[46]. Moreover, nutritional stress conditions *in vitro *also require upregulation of the glyoxylate cycle enzymes in *P. brasiliensis *[46].

Secreted enzymes such as phospholipases, peptidases, and proteases are crucial for dermatophyte virulence since these pathogens infect the stratum corneum, nails, or hair [47-49]. During infection, *T. rubrum *carboxypeptidases may contribute to fungal virulence by cooperating with endoproteases and aminopeptidases to degrade compact keratinized tissues into short peptides and amino acids that can be assimilated [50] (Table 2).

Various membrane transporters are virulence factors that are commonly involved in bacterial and fungal pathogenesis and ensure successful colonization of the host. For example, transmembrane proteins involved in the transport of metallic ions appear to play an important role in microbial pathogenesis [51] as demonstrated in the Cu^2+^-ATPase mutants of *Listeria monocytogenes *[52] and *Criptococcus neoformans *[53] that show reduced virulence. In the latter case, the Δ*vph1 *mutant did not display laccase activity, which is an essential virulence factor of this pathogen [53]. Moreover, an ATP-binding cassette (ABC) transporter listed in Table 2 is overexpressed in mycelia cultured in keratin, suggesting its involvement in *T. rubrum *pathogenicity. In addition, the strain carrying a disrupted version of this MDR gene (Δ*TruMDR2*) showed low infectious capability characterized by reduced growth of *T. rubrum *on human nails [40].

## Conclusions

We identified 575 novel ESTs and obtained new molecular data related to *T. rubrum *growth, pH and carbon source signaling, and stress responses to antifungal challenges. It is clear that additional studies are necessary to define the functioning of whole genes and fully understand the regulation of these complex adaptive responses. However, the various ESTs identified in this work provide new insights into different aspects of *T. rubrum *biology, revealing new sources for functional genome analysis. *T. rubrum *genes that encode putative proteins similar to virulence factors described for other fungi were among the ESTs identified. The transcriptional profile also suggested that several genes could function in environmental stress responses. Thus, our study can help to better understand the molecular mechanisms of the adaptive responses possibly involved in dermatophyte infection and antifungal resistance.

## Methods

### Strains and culture conditions

The H6 (ATCC MYA-3108) and F6 mutant (a fluconazole-resistant strain isolated in our laboratory) strains of *T. rubrum *were cultured on Sabouraud dextrose agar plates (SDA) as described earlier [54]. The F6 cultures were supplemented with fluconazole (200 μg/mL). Conidia from these strains were used to construct the cDNA (library 1) or were inoculated in Sabouraud dextrose broth (SDB) and incubated for 72 h at 28°C on an orbital shaker at 180 rpm. The resulting mycelia were aseptically transferred to the desired culture media, and these were used to construct each of the SSH libraries.

### Construction of the libraries

One cDNA library (Library 1) and nine SSH libraries (Libraries 2 to 10) were constructed. The SSH libraries were performed between the tester and driver DNA, with the cDNA population containing the differentially expressed transcripts being the tester, and the reference cDNA (control) being the driver.

Total RNA was extracted from approximately 100 mg of frozen mycelia obtained from each procedure by using the Nucleospin^® ^RNA Plant (Macherey-Nagel) or TRIZOL Reagent (Invitrogen), and treated with DNase Amp Grade (Invitrogen), in accordance with the manufacturer's instructions. Double-stranded cDNAs were obtained with the SMART PCR Synthesis kit (BD Biosciences) to amplify the cDNAs before the SSH procedure or the cDNA cloning step. The exceptions were libraries 2, 6, and 7, in which poly(A^+^) RNA was isolated from total RNA using Oligotex mRNA spin columns (Qiagen) or the PolyA Tract^® ^mRNA Isolation System (Promega). Library 1 (cDNA library) was constructed with the Creator SMART cDNA Library Construction Kit (BD Biosciences). *Sfi*I-digested cDNAs were unidirectionally cloned into the pDNR-LIB vector and transformed into *Escherichia coli *TOP10F' electrocompetent cells. Libraries 2 to 10 were prepared by using the PCR-Select cDNA Subtraction kit (BD Biosciences). The cDNAs obtained from each SSH were cloned into the pCR 2.1 TOPO TA cloning system (Invitrogen) or pGEM-T cloning vector (Promega) and transformed into *Escherichia coli *Mos-Blue-competent cells.

#### Library 1. Developmental phase-enriched transcripts

Conidia from the H6 strain were incubated in keratinocyte serum-free medium (KGM-SFM, Gibco) for 16, 24, 48, and 72 h at 37°C. The cDNA transcribed from total RNA extracted from mycelia incubated in each experiment were mixed and used to construct the cDNA library as described above.

#### Library 2. Cytotoxic drug-enriched transcripts

Mycelia obtained from the H6 strain were exposed to each of the following cytotoxic drugs: ACR (2.5 μg/mL), BEN (2.5 μg/mL), CAP (50 mg/mL), CHX (30 mg/mL), EB (2.5 μg/mL), FLC (130 μg/mL), 4NQO (10 μg/mL), GRS (2.0 μg/mL), IMZ (4.0 μg/mL), ITRA (30 μg/mL), KTC (10 μg/mL), TRB (0.1 μg/mL), TIO (0.5 μg/mL), or UDA (50 μg/mL). The final concentration of each drug corresponds to its sub-inhibitory concentration. The cultures were incubated for 15 min at 28°C, aiming the identification of genes expressed early during exposure to cytotoxic drugs. SSH was performed between the tester (mixture of cDNA transcribed from total RNA extracted from mycelia exposed to each drug) and driver (mRNA obtained from mycelia incubated into drug-free medium).

#### Library 3. AMB-enriched transcripts

Tester: mycelia obtained from the H6 strain were aseptically transferred to RPMI 1640 (Gibco) containing AMB (0.5 μg/mL) and incubated for 90 min at 28°C. Driver: mycelia were transferred to a drug-free medium.

#### Library 4. FLC-enriched transcripts in the F6 mutant

Tester: mycelia from the F6 strain were transferred to fresh SDB containing FLC (250 μg/mL), and incubated for 1 h at 28°C. Driver: mycelia from the H6 strain were inoculated in the drug-free medium.

#### Library 5. FLC-repressed transcripts in the F6 mutant

Tester: mycelia from the H6 strain were aseptically transferred to fresh SDB, and incubated for 1 h at 28°C. Driver: mycelia from the F6 strain were aseptically transferred to SDB containing FLC (250 μg/mL).

#### Library 6. Glucose-enriched transcripts

Tester: mycelia from the H6 strain were transferred to minimal medium supplemented with 55 mM glucose and 70 mM sodium nitrate (MM) [55] at pH 5.0 and incubated for 72 h at 28°C. Driver: mycelia were aseptically transferred to keratin medium (KM) containing MM supplemented with 2.5 g/L keratin (Sigma) as the carbon source (pH 5.0).

#### Library 7. Keratin-enriched transcripts

Tester: mycelia from the H6 strain were transferred to KM and incubated for 72 h at 28°C. Driver: mycelia were transferred to MM [55].

#### Library 8. pH 5.0-enriched transcripts (30-min exposure)

Tester: mycelia from the H6 strain were transferred to MM [55] containing 2.0 mM inorganic phosphate (Pi) (low-Pi MM) (pH 5.0), and incubated for 30 min at 28°C. Driver: mycelia were transferred to low-Pi MM (pH 8.0).

#### Library 9. pH 5.0-enriched transcripts (60-min exposure)

Tester: mycelia from the H6 strain were transferred to low-Pi MM (pH 5.0), and incubated for 1 h at 28°C. Driver: mycelia were transferred to low-Pi MM (pH 8.0).

#### Library 10. pH 8.0-enriched transcripts (60-min exposure)

Tester: mycelia from the H6 strain were transferred to low-Pi MM (pH 8.0), and incubated for 1 h at 28°C. Driver: mycelia transferred to low-Pi MM (pH 5.0).

### cDNA sequencing and validation of differentially expressed genes

The cDNAs corresponding to differentially expressed sequences in the SSH libraries were amplified by PCR, and the products were screened by reverse Northern hybridization, as described earlier [56]. The plasmids from arrayed clones that visually exhibited positive differential expression were sequenced using the M13 forward or reverse primers and BigDye Terminator Cycle Sequencing Kit in an automated ABI Prism^® ^377 DNA Sequencer (Applied Biosystems).

For validating differential gene expression by northern blot analysis, *T. rubrum *was cultivated as described for constructing the subtractive suppressive cDNA libraries. Samples containing approximately 15 μg of total RNA were extracted with the Illustra RNAspin Isolation kit (GE Healthcare) and separated by electrophoresis on a 1.5% agarose gel containing formaldehyde. They were blotted onto Hybond-N+ membranes and hybridized with cDNA probes labeled with [α-^32^P]dCTP.

### EST processing pipeline and annotation

EST processing included base calling, quality control by Phred, and trimming (which involves the removal of low-quality vector and adapter sequences) by Cross Match [57,58]. The accepted sequences contained at least 80 nucleotides with a Phred quality value higher than 20. Assembly of ESTs into clusters of overlapping sequences (contigs) was carried out with the CAP3 program using default parameters [59]. Singletons represent sequences that have no overlap with other ESTs. Unigenes (the number of contigs plus the number of singletons) are nonredundant sequences obtained after CAP3 assembly. Redundancy was estimated as the total number of ESTs minus the number of unigenes divided by the total number of ESTs, and the resulting value was transformed into a percentage.

For EST annotation, the unigenes were compared to the EST database (dbEST) and the nonredundant protein database (nr-GenBank) at the National Center for Biotechnology Information (NCBI, Bethesda, MD; http://www.ncbi.nlm.nih.gov). *T. rubrum *dbEST consists of ESTs from this species deposited in the public database. High-throughput scripts for the BLAST algorithms BLASTx and BLASTn [60] were used to search the nr-GenBank and *T. rubrum *dbEST, respectively, using the Blosum 62 matrix and default BLAST parameters. Similarity search against dbEST using the BLASTn algorithm, excluding the sequences previously deposited by our group, was regarded to be significant when the expected value (e-value) was less than 1e^-20^. For BLASTx searching, the top 5 scoring hits with e-values lower than 1e^-3 ^were used to annotate each EST. Sequences that did not return alignments with the established e-value cut-offs were considered as no-matches. Our results were also compared to TrED database http://www.mgc.ac.cn/TrED. The functional classification of these unigenes was performed according to the Functional Catalogue created by the Munich Information Center for Protein Sequences (MIPS), gathered through a BLAST comparison of the query sequence (unigenes) against MIPS-annotated proteins from *Saccharomyces cerevisiae*, *Neurospora crassa*, *Fusarium graminearum*, and *Ustilago maydis *[61,62]. This retrieves the MIPS accession number from the best hit (considering a minimum e-value of 1e^-3^), which in turn retrieves the functional category from the MIPS FunCat table. All computer analyses were performed on Intel-based computers (P4 and Xeon) using the Linux-based operating system Fedora 6. The scripts and programs were developed using the PERL language, and the web pages were created using CGI, Javascript, and HTML.

## Authors' contributions

NTAP participated in the construction of the cDNA gene library, clone isolation, data analysis, and drafted the manuscript. PRS performed the statistical and bioinformatics analyses. JPF participated in the construction of the cDNA gene library and clone isolation. FGP, HCSS, FCAM, DEG, FS, RAC, and JRCS constructed the SSH libraries, performed the northern blots, and collaborated on data analysis. RAF and MM were responsible for strain identification, designing of the culture and growth conditions, and cDNA sequencing. AR and NMMR designed the project, supervised the research study, and prepared the manuscript. All authors read and approved the final manuscript.

## Supplementary Material

Additional file 1 ***T. rubrum *EST database**. The data show the complete list of ESTs that are differentially expressed in *T. rubrum *under different experimental conditions. Click here for file

Additional file 2 ***T. rubrum *unigenes database**. The data show the complete list of unigenes that are differentially expressed in *T. rubrum *under each experimental condition, the novel *T. rubrum *genes (highlighted) and their MIPS categorization. Click here for file
