# Supplementary material for: Transcriptional profiling reveals the expression of novel genes in response to various stimuli in the human dermatophyte Trichophyton rubrum
Source: BMC Microbiol. 2010 Feb 8;10:39. doi: 10.1186/1471-2180-10-39 (PMC2831883; doi:10.1186/1471-2180-10-39)
Supplement: Additional file 1 — T. rubrum EST database. The data show the complete list of ESTs that are differentially expressed in T. rubrum under different experimental conditions. [file 1471-2180-10-39-S1.PDF]

Additional file 1. *T. rubrum* EST database

GenBank Accession No.	Similarity			
	Sequence ID	Gene product	Organism	E-value
FE524602	-	No significant similarity	-	-
FE524603	-	No significant similarity	-	-
FE524604	-	No significant similarity	-	-
FE524605	-	No significant similarity	-	-
FE524606	ref|XP_001241782.1|	conserved hypothetical protein	*Coccidioides immitis RS*	6e-22
FE524607	-	No significant similarity	-	-
FE524608	-	No significant similarity	-	-
FE524609	ref|XP_001247341.1|	hypothetical protein CIMG_01112	*Coccidioides immitis RS*	2e-24
FE524610	emb|CAK40436.1|	unnamed protein product	*Aspergillus niger*	7e-08
FE524611	-	No significant similarity	-	-
FE524612	ref|XP_001264707.1|	S-adenosylmethionine synthetase	*Neosartorya fischeri NRRL 181*	8e-22
FE524613	-	No significant similarity	-	-
FE524614	-	No significant similarity	-	-
FE524615	-	No significant similarity	-	-
FE524616	-	No significant similarity	-	-
FE524617	-	No significant similarity	-	-
FE524618	-	No significant similarity	-	-
FE524619	-	No significant similarity	-	-
FE524620	sp|P10255|CYPH_NEUCR	Peptidyl-prolyl cis-trans isomerase, mitochondrial precursor (PPIase) (Rotamase) (Cyclophilin) (Cyclosporin A-binding protein) (CPH) gb|AAA33584.1| cyclophilin precursor emb|CAA35681.1| cyclophilin (mitochondrial precursor)	*Neurospora crassa*	3e-17
FE524621	-	No significant similarity	-	-
FE524622	-	No significant similarity	-	-
FE524623	-	No significant similarity	-	-
FE524624	ref|XP_001246797.1|	polyubiquitin	*Coccidioides immitis RS*	3e-32
FE524625	ref|XP_001241788.1|	hypothetical protein CIMG_05684	*Coccidioides immitis RS*	1e-13
FE524626	-	No significant similarity	-	-
FE524627	ref|XP_001241788.1|	hypothetical protein CIMG_05684	*Coccidioides immitis RS*	5e-14
FE524628	-	No significant similarity	-	-
FE524629	ref|XP_001267336.1|	outer mitochondrial membrane protein porin	*Neosartorya fischeri NRRL 181*	9e-18

FE524630	-	No significant similarity	-	-
FE524631	ref|XP_001247117.1|	60S ribosomal protein L44	*Coccidioides immitis RS*	2e-53
FE524632	gb|AAO52807.1|	hypothetical protein	*Bacillus megaterium*	1e-04
FE524633	ref|XP_001241788.1|	hypothetical protein CIMG_05684	*Coccidioides immitis RS*	1e-11
FE524634	-	No significant similarity	-	-
FE524635	-	No significant similarity	-	-
FE524636	-	No significant similarity	-	-
FE524637	dbj|BAB12047.1|	alpha-crystallin-related protein	*Arthroderma benhamiae*	1e-18
FE524638	-	No significant similarity	-	-
FE524639	ref|XP_001241788.1|	hypothetical protein CIMG_05684	*Coccidioides immitis RS*	2e-11
FE524640	-	No significant similarity	-	-
FE524641	-	No significant similarity	-	-
FE524642	-	No significant similarity	-	-
FE524643	ref|XP_729762.1|	senescence-associated protein	*Plasmodium yoelii yoelii str. 17XNL*	5e-06
FE524644	gb|AAO52807.1|	hypothetical protein	*Bacillus megaterium*	2e-04
FE524645	ref|XP_001267438.1|	hypothetical protein NFIA_043590	*Neosartorya fischeri NRRL 181*	1e-11
FE524646	ref|XP_001240361.1|	hypothetical protein CIMG_07524	*Coccidioides immitis RS*	3e-06
FE524647	-	No significant similarity	-	-
FE524648	-	No significant similarity	-	-
FE524649	-	No significant similarity	-	-
FE524650	-	No significant similarity	-	-
FE524651	-	No significant similarity	-	-
FE524652	ref|XP_001241788.1|	hypothetical protein CIMG_05684	*Coccidioides immitis RS*	3e-12
FE524653	-	No significant similarity	-	-
FE524654	-	No significant similarity	-	-
FE524655	-	No significant similarity	-	-
FE524656	-	No significant similarity	-	-
FE524657	-	No significant similarity	-	-
FE524658	ref|XP_001241788.1|	hypothetical protein CIMG_05684	*Coccidioides immitis RS*	5e-14
FE524659	ref|XP_001217109.1|	methionyl-tRNA synthetase	*Aspergillus terreus NIH2624*	4e-04
FE524660	gb|AAP05987.3|	70 kDa heat shock protein	*Paracoccidioides brasiliensis*	8e-77
FE524661	-	No significant similarity	-	-
FE524662	sp|P0C2C8|ATP7_ASPTN	ATP synthase D chain, mitochondrial precursor	*Aspergillus terreus NIH2624*	1e-55

FE524663	ref|XP_001268300.1|	chaperone/heat shock protein Hsp12, putative	*Aspergillus clavatus NRRL 1*	5e-20
FE524664	ref|XP_001211604.1|	predicted protein	*Aspergillus terreus NIH2624*	1e-05
FE524665	ref|XP_001273409.1|	dihydrolipoamide acetyltransferase component of pyruvate dehydrogenase	*Aspergillus clavatus NRRL 1*	1e-11
FE524666	-	No significant similarity	-	-
FE524667	ref|XP_001273409.1|	dihydrolipoamide acetyltransferase component of pyruvate dehydrogenase	*Aspergillus clavatus NRRL 1*	2e-05
FE524668	-	No significant similarity	-	-
FE524669	-	No significant similarity	-	-
FE524670	-	No significant similarity	-	-
FE524671	-	No significant similarity	-	-
FE524672	ref|XP_001216088.1|	splicing factor U2AF 23 kDa subunit	*Aspergillus terreus NIH2624*	4e-08
FE524673	-	No significant similarity	-	-
FE524674	-	No significant similarity	-	-
FE524675	-	No significant similarity	-	-
FE524676	sp|P09188|PGK_PENCH	Phosphoglycerate kinase emb|CAA31756.1| PGK protein	*Penicillium chrysogenum*	8e-15
FE524677	-	No significant similarity	-	-
FE524678	-	No significant similarity	-	-
FE524679	gb|AAF04257.1|	subtilisin-like serine protease	*Neospora caninum*	7e-05
FE524680	gb|AAB05810.1|	super cysteine rich protein; SCRP	*Homo sapiens*	4e-10
FE524681	-	No significant similarity	-	-
FE524682	dbj|BAB12047.1|	alpha-crystallin-related protein	*Arthroderma benhamiae*	3e-09
FE524683	-	No significant similarity	-	-
FE524684	ref|XP_001211720.1|	60S ribosomal protein L12	*Aspergillus terreus NIH2624*	6e-14
FE524685	-	No significant similarity	-	-
FE524686	-	No significant similarity	-	-
FE524687	-	No significant similarity	-	-
FE524688	-	No significant similarity	-	-
FE524689	gb|EDJ96333.1|	ubiquitin fusion protein	*Magnaporthe grisea 70-15*	7e-35
FE524690	-	No significant similarity	-	-
FE524691	-	No significant similarity	-	-
FE524692	-	No significant similarity	-	-

FE524693	-	No significant similarity	-	-
FE524694	emb|CAJ83813.1|	CHK1 checkpoint homolog (S. pombe)	*Xenopus tropicalis*	3e-09
FE524695	ref|XP_752703.1|	Arp2/3 complex subunit (Arp2), putative	*Aspergillus fumigatus Af293*	2e-69
FE524696	ref|XP_001241781.1|	conserved hypothetical protein	*Coccidioides immitis RS*	2e-19
FE524697	-	No significant similarity	-	-
FE524698	-	No significant similarity	-	-
FE524699	ref|XP_001259633.1|	ATP synthase F1, beta subunit, putative	*Neosartorya fischeri NRRL 181*	5e-38
FE524700	ref|XP_746929.1|	vacuolar protein sorting protein, putative	*Aspergillus fumigatus Af293*	2e-12
FE524701	-	No significant similarity	-	-
FE524702	-	No significant similarity	-	-
FE524703	ref|XP_001263333.1|	proteasome subunit alpha type	*Neosartorya fischeri NRRL 181*	5e-26
FE524704	ref|XP_001222064.1|	40S ribosomal protein S13	*Chaetomium globosum CBS 148.51*	7e-52
FE524705	-	No significant similarity	-	-
FE524706	-	No significant similarity	-	-
FE524707	ref|XP_001244930.1|	predicted protein	*Coccidioides immitis RS*	1e-08
FE524708	-	No significant similarity	-	-
FE524709	-	No significant similarity	-	-
FE524710	-	No significant similarity	-	-
FE524711	-	No significant similarity	-	-
FE524712	ref|XP_755632.1|	cytochrome c oxidase polypeptide vib	*Aspergillus fumigatus Af293*	3e-31
FE524713	-	No significant similarity	-	-
FE524714	gb|EAL88345.2|	60S ribosomal protein L27e	*Aspergillus fumigatus Af293*	1e-18
FE524715	-	No significant similarity	-	-
FE524716	-	No significant similarity	-	-
FE524717	-	No significant similarity	-	-
FE524718	-	No significant similarity	-	-
FE524719	-	No significant similarity	-	-
FE524720	-	No significant similarity	-	-
FE524721	-	No significant similarity	-	-
FE524722	-	No significant similarity	-	-
FE524723	pir||B24264	proline-rich protein MP3 - mouse (fragment)	*Mus musculus*	1e-05

FE524724	-	No significant similarity	-	-
FE524725	-	No significant similarity	-	-
FE524726	-	No significant similarity	-	-
FE524727	ref|XP_001267666.1|	hypothetical protein NFIA_061330	*Neosartorya fischeri NRRL 181*	3e-19
FE524728	ref|XP_001245526.1|	hypothetical protein CIMG_04967	*Coccidioides immitis RS*	3e-28
FE524729	-	No significant similarity	-	-
FE524730	-	No significant similarity	-	-
FE524731	-	No significant similarity	-	-
FE524732	-	No significant similarity	-	-
FE524733	-	No significant similarity	-	-
FE524734	gb|AAY23172.1|	cytochrome c oxidase polypeptide VIa	*Penicillium chrysogenum*	3e-08
FE524735	-	No significant similarity	-	-
FE524736	-	No significant similarity	-	-
FE524737	-	No significant similarity	-	-
FE524738	-	No significant similarity	-	-
FE524739	gb|ABI52743.1|	10 kDa putative secreted protein	*Argas monolakensis*	2e-08
FE524740	-	No significant similarity	-	-
FE524741	ref|XP_001248928.1|	elongation factor 1-gamma	*Coccidioides immitis RS*	8e-32
FE524742	-	No significant similarity	-	-
FE524743	-	No significant similarity	-	-
FE524744	emb|CAL46260.1|	putative mitochondrial inner membrane protein 1	*Botryotinia fuckeliana*	6e-13
FE524745	ref|XP_001213975.1|	protein kinase dsk1	*Aspergillus terreus NIH2624*	3e-17
FE524746	sp|Q9P8I0|PUT2_EMENI	Delta-1-pyrroline-5-carboxylate dehydrogenase, mitochondrial precursor (P5C dehydrogenase) gb|AAF72527.1|AF252630_1 delta-1-pyrroline-5-carboxylate dehydrogenase	*Aspergillus nidulans*	8e-17
FE524747	-	No significant similarity	-	-
FE524748	-	No significant similarity	-	-
FE524749	-	No significant similarity	-	-
FE524750	-	No significant similarity	-	-
FE524751	emb|CAJ83813.1|	CHK1 checkpoint homolog (S. pombe)	*Xenopus tropicalis*	6e-09
FE524752	dbj|BAE64245.1|	unnamed protein product	*Aspergillus oryzae*	5e-22
FE524753	-	No significant similarity	-	-
FE524754	dbj|BAE65103.1|	unnamed protein product	*Aspergillus oryzae*	2e-07
FE524755	-	No significant similarity	-	-
FE524756	gb|AAW69337.1|	ERV2 protein-like protein	*Magnaporthe grisea*	4e-17

FE524757	-	No significant similarity	-	-
FE524758	-	No significant similarity	-	-
FE524759	-	No significant similarity	-	-
FE524760	-	No significant similarity	-	-
FE524761	-	No significant similarity	-	-
FE524762	-	No significant similarity	-	-
FE524763	-	No significant similarity	-	-
FE524764	ref|XP_001245839.1|	arginyl-tRNA synthetase	*Coccidioides immitis RS*	1e-53
FE524765	-	No significant similarity	-	-
FE524766	-	No significant similarity	-	-
FE524767	gb|AAR24348.1|	14-3-3-like protein 2	*Paracoccidioides brasiliensis*	3e-81
FE524768	-	No significant similarity	-	-
FE524769	ref|XP_001246797.1|	polyubiquitin	*Coccidioides immitis RS*	8e-93
FE524770	ref|XP_001267666.1|	hypothetical protein NFIA_061330	*Neosartorya fischeri NRRL 181*	3e-07
FE524771	ref|XP_001241781.1|	conserved hypothetical protein	*Coccidioides immitis RS*	8e-17
FE524772	ref|XP_001271667.1|	60S ribosomal protein L11	*Aspergillus clavatus NRRL 1*	3e-63
FE524773	-	No significant similarity	-	-
FE524774	-	No significant similarity	-	-
FE524775	-	No significant similarity	-	-
FE524776	-	No significant similarity	-	-
FE524777	-	No significant similarity	-	-
FE524778	-	No significant similarity	-	-
FE524779	-	No significant similarity	-	-
FE524780	-	No significant similarity	-	-
FE524781	-	No significant similarity	-	-
FE524782	ref|XP_001263817.1|	UBX domain protein	*Neosartorya fischeri NRRL 181*	1e-21
FE524783	ref|XP_001241788.1|	hypothetical protein CIMG_05684	*Coccidioides immitis RS*	5e-14
FE524784	-	No significant similarity	-	-
FE524785	-	No significant similarity	-	-
FE524786	-	No significant similarity	-	-
FE524787	-	No significant similarity	-	-
FE524788	-	No significant similarity	-	-
FE524789	-	No significant similarity	-	-
FE524790	gb|AAX27763.1|	SJCHGC01957 protein	*Schistosoma japonicum*	4e-16

FE524791	-	No significant similarity	-	-
FE524792	ref|XP_001028186.1|	hypothetical protein TTHERM_02641280	*Tetrahymena thermophila SB210*	1e-11
FE524793	ref|XP_001242478.1|	hypothetical protein CIMG_06374	*Coccidioides immitis RS*	3e-06
FE524794	dbj|BAD90390.1|	mFLJ00348 protein	*Mus musculus*	4e-04
FE524795	-	No significant similarity	-	-
FE524796	-	No significant similarity	-	-
FE524797	gb|AAL76232.1|AF408429_1	metallothionein	*Microsporum canis*	2e-04
FE524798	ref|XP_001241788.1|	hypothetical protein CIMG_05684	*Coccidioides immitis RS*	2e-11
FE524799	-	No significant similarity	-	-
FE524800	-	No significant similarity	-	-
FE524801	ref|XP_001243274.1|	hypothetical protein CIMG_07170	*Coccidioides immitis RS*	5e-39
FE524802	-	No significant similarity	-	-
FE524803	ref|XP_001270912.1|	proteasome regulatory particle subunit (RpnL), putative	*Aspergillus clavatus NRRL 1*	2e-61
FE524804	ref|XP_001214565.1|	succinate-semialdehyde dehydrogenase	*Aspergillus terreus NIH2624*	1e-05
FE524805	-	No significant similarity	-	-
FE524806	ref|XP_750061.1|	ribosomal protein L29/heparin/heparan sulfate interacting protein	*Aspergillus fumigatus Af293*	3e-20
FE524807	-	No significant similarity	-	-
FE524808	gb|AAC13689.1|	ubiquitin fusion protein	*Magnaporthe grisea*	3e-47
FE524809	ref|XP_001241788.1|	hypothetical protein CIMG_05684	*Coccidioides immitis RS*	2e-14
FE524810	-	No significant similarity	-	-
FE524811	ref|XP_001271742.1|	acyl-CoA dehydrogenase, putative	*Aspergillus clavatus NRRL 1*	6e-23
FE524812	-	No significant similarity	-	-
FE524813	-	No significant similarity	-	-
FE524814	ref|XP_750122.1|	UPF0136 domain protein	*Aspergillus fumigatus Af293*	8e-25
FE524815	-	No significant similarity	-	-
FE524816	-	No significant similarity	-	-
FE524817	-	No significant similarity	-	-
FE524818	emb|CAJ83813.1|	CHK1 checkpoint homolog (S. pombe)	*Xenopus tropicalis*	2e-09
FE524819	-	No significant similarity	-	-
FE524820	-	No significant similarity	-	-
FE524821	ref|XP_001241788.1|	hypothetical protein CIMG_05684	*Coccidioides immitis RS*	1e-13
FE524822	-	No significant similarity	-	-

FE524823	-	No significant similarity	-	-
FE524824	ref|XP_750146.1|	Rhodanese domain protein	*Aspergillus fumigatus Af293*	5e-37
FE524825	-	No significant similarity	-	-
FE524826	-	No significant similarity	-	-
FE524827	-	No significant similarity	-	-
FE524828	-	No significant similarity	-	-
FE524829	-	No significant similarity	-	-
FE524830	ref|XP_001271126.1|	ubiquitin	*Aspergillus clavatus NRRL 1*	7e-16
FE524831	-	No significant similarity	-	-
FE524832	emb|CAJ83813.1|	CHK1 checkpoint homolog (S. pombe)	*Xenopus tropicalis*	2e-09
FE524833	ref|XP_001247270.1|	hypothetical protein CIMG_01041	*Coccidioides immitis RS*	3e-12
FE524834	ref|XP_001241788.1|	hypothetical protein CIMG_05684	*Coccidioides immitis RS*	5e-14
FE524835	-	No significant similarity	-	-
FE524836	-	No significant similarity	-	-
FE524837	-	No significant similarity	-	-
FE524838	ref|XP_001241990.1|	tubulin gamma chain	*Coccidioides immitis RS*	1e-95
FE524839	-	No significant similarity	-	-
FE524840	-	No significant similarity	-	-
FE524841	ref|XP_001210407.1|	elongation factor 1-beta	*Aspergillus terreus NIH2624*	4e-07
FE524842	-	No significant similarity	-	-
FE524843	-	No significant similarity	-	-
FE524844	-	No significant similarity	-	-
FE524845	ref|XP_001275472.1|	60S ribosomal protein L3	*Aspergillus clavatus NRRL 1*	8e-25
FE524846	-	No significant similarity	-	-
FE524847	-	No significant similarity	-	-
FE524848	ref|XP_001241788.1|	hypothetical protein CIMG_05684	*Coccidioides immitis RS*	5e-14
FE524849	sp|P39457|PLB1_PENCH	Lysophospholipase precursor (Phospholipase B) emb|CAA42906.1| lysophospholipase	*Penicillium chrysogenum*	4e-04
FE524850	ref|XP_747457.1|	DUF895 domain membrane protein	*Aspergillus fumigatus Af293*	3e-25
FE524851	-	No significant similarity	-	-
FE524852	-	No significant similarity	-	-
FE524853	-	No significant similarity	-	-
FE524854	-	No significant similarity	-	-

FE524855	ref|XP_001212274.1|	40S ribosomal protein S19	*Aspergillus terreus NIH2624*	1e-48
FE524856	-	No significant similarity	-	-
FE524857	ref|XP_001241788.1|	hypothetical protein CIMG_05684	*Coccidioides immitis RS*	2e-13
FE524858	-	No significant similarity	-	-
FE524859	-	No significant similarity	-	-
FE524860	ref|XP_001241788.1|	hypothetical protein CIMG_05684	*Coccidioides immitis RS*	2e-13
FE524861	-	No significant similarity	-	-
FE524862	gb|AAM54368.1|	elongation factor 1-alpha	*Trichophyton rubrum*	3e-47
FE524863	ref|XP_001247280.1|	alternative oxidase, mitochondrial precursor	*Coccidioides immitis RS*	4e-11
FE524864	-	No significant similarity	-	-
FE524865	-	No significant similarity	-	-
FE524866	-	No significant similarity	-	-
FE524867	-	No significant similarity	-	-
FE524868	-	No significant similarity	-	-
FE524869	-	No significant similarity	-	-
FE524870	-	No significant similarity	-	-
FE524871	-	No significant similarity	-	-
FE524872	-	No significant similarity	-	-
FE524873	-	No significant similarity	-	-
FE524874	-	No significant similarity	-	-
FE524875	gb|AAO52807.1|	hypothetical protein	*Bacillus megaterium*	1e-04
FE524876	ref|XP_001274544.1|	N-acetylglucosaminyl-phosphatidylinositol deacetylase, putative	*Aspergillus clavatus NRRL 1*	2e-20
FE524877	ref|XP_001129064.1|	PREDICTED: hypothetical protein	*Homo sapiens*	8e-04
FE524878	ref|XP_001247341.1|	hypothetical protein CIMG_01112	*Coccidioides immitis RS*	5e-13
FE524879	-	No significant similarity	-	-
FE524880	ref|XP_828313.1|	hypothetical protein Tb11.18.0001	*Trypanosoma brucei TREU927*	9e-21
FE524881	ref|XP_385039.1|	hypothetical protein FG04863.1	*Gibberella zeae PH-1*	1e-06
FE524882	-	No significant similarity	-	-
FE524883	gb|ABB20530.1|	3-hydroxyphenylacetate 6 hydroxylase	*Emericella nidulans*	6e-12
FE524884	ref|XP_001241788.1|	hypothetical protein CIMG_05684	*Coccidioides immitis RS*	5e-14
FE524885	-	No significant similarity	-	-
FE524886	-	No significant similarity	-	-
FE524887	emb|CAE72981.1|	Hypothetical protein CBG20323	*Caenorhabditis briggsae*	5e-16
FE524888	-	No significant similarity	-	-
FE524889	-	No significant similarity	-	-

FE524890	ref|XP_001257424.1|	ubiquitin C-terminal hydrolase, putative	*Neosartorya fischeri NRRL 181*	2e-08
FE524891	ref|XP_001275443.1|	galactose-1-phosphate uridylyltransferase	*Aspergillus clavatus NRRL 1*	2e-34
FE524892	-	No significant similarity	-	-
FE524893	-	No significant similarity	-	-
FE524894	-	No significant similarity	-	-
FE524895	gb|EU076569.1|	Dipeptidyl-peptidase 5 (DPP5)	*Trichophyton equinum*	2e-35
FE524896	-	No significant similarity	-	-
FE524897	ref|XP_749924.1|	t-complex protein 1, beta subunit, putative	*Aspergillus fumigatus Af293*	6e-52
FE524898	-	No significant similarity	-	-
FE524899	ref|XP_001358279.1|	GA19372-PA	*Drosophila pseudoobscura*	3e-10
FE524900	-	No significant similarity	-	-
FE524901	ref|XP_001241788.1|	hypothetical protein CIMG_05684	*Coccidioides immitis RS*	4e-14
FE524902	ref|XP_001267666.1|	hypothetical protein NFIA_061330	*Neosartorya fischeri NRRL 181*	9e-31
FE524903	-	No significant similarity	-	-
FE524904	emb|CAJ83813.1|	CHK1 checkpoint homolog (S. pombe)	*Xenopus tropicalis*	2e-09
FE524905	-	No significant similarity	-	-
FE524906	ref|XP_001247816.1|	superoxide dismutase	*Coccidioides immitis RS*	1e-08
FE524907	-	No significant similarity	-	-
FE524908	-	No significant similarity	-	-
FE524909	ref|XP_001247341.1|	hypothetical protein CIMG_01112	*Coccidioides immitis RS*	4e-08
FE524910	-	No significant similarity	-	-
FE524911	ref|XP_001264967.1|	30S ribosomal subunit S4, putative	*Neosartorya fischeri NRRL 181*	2e-28
FE524912	pir||T02955	probable cytochrome P450 monooxygenase - maize (fragment	*probable cytochrome P450 monooxygenas*	5e-06
FE524913	ref|XP_001241788.1|	hypothetical protein CIMG_05684	*Coccidioides immitis RS*	2e-13
FE524914	-	No significant similarity	-	-
FE524915	gb|ABH10636.1|	elongation factor 2	*Coccidioides posadasii*	1e-33
FE524916	-	No significant similarity	-	-
FE524917	-	No significant similarity	-	-
FE524918	sp|P14728|YAV2_XANCV	Hypothetical 82 kDa avirulence protein in avrBs3 region	*Xanthomonas euvesicatoria*	6e-10
FE524919	ref|XP_001268551.1|	conidial hydrophobin RodB	*Aspergillus clavatus*	1e-04

			NRRL 1	
FE524920	ref|XP_001208527.1|	cytochrome c oxidase assembly protein COX11, mitochondrial precursor	*Aspergillus terreus NIH2624*	1e-26
FE524921	ref|XP_001267676.1|	hypothetical protein NFIA_043490	*Neosartorya fischeri NRRL 181*	4e-08
FE524922	ref|XP_001241788.1|	hypothetical protein CIMG_05684	*Coccidioides immitis RS*	3e-04
FE524923	-	No significant similarity	-	-
FE524924	-	No significant similarity	-	-
FE524925	-	No significant similarity	-	-
FE524926	gb|AAX82553.1|	120 kDa pistil extensin-like protein	*Nicotiana langsdorfii*	2e-04
FE524927	-	No significant similarity	-	-
FE524928	ref|XP_571345.1|	ketol-acid reductoisomerase	*Cryptococcus neoformans var. neoformans JEC21*	7e-13
FE524929	-	No significant similarity	-	-
FE524930	gb|EAL88350.2|	translation elongation factor EF-1 alpha subunit , putative	*Aspergillus fumigatus Af293*	2e-22
FE524931	ref|XP_001243230.1|	40S ribosomal protein S23	*Coccidioides immitis RS*	5e-19
FE524932	-	No significant similarity	-	-
FE524933	-	No significant similarity	-	-
FE524934	-	No significant similarity	-	-
FE524935	-	No significant similarity	-	-
FE524936	gb|EDJ97592.1|	sodium P-type ATPase, putative	*Magnaporthe grisea 70-15*	6e-04
FE524937	ref|XP_001258284.1|	polyketide synthase, putative	*Neosartorya fischeri NRRL 181*	5e-14
FE524938	ref|XP_001244923.1|	hypothetical protein CIMG_04364	*Coccidioides immitis RS*	2e-04
FE524939	-	No significant similarity	-	-
FE524940	gb|EAW96235.1|	hCG2043380	*Homo sapiens*	1e-06
FE524941	-	No significant similarity	-	-
FE524942	-	No significant similarity	-	-
FE524943	-	No significant similarity	-	-
FE524944	-	No significant similarity	-	-
FE524945	-	No significant similarity	-	-
FE524946	pir||T02955	probable cytochrome P450 monooxygenase - maize (fragment)	*Zea mays*	6e-06
FE524947	-	No significant similarity	-	-
FE524948	-	No significant similarity	-	-
FE524949	ref|XP_001241788.1|	hypothetical protein CIMG_05684	*Coccidioides immitis RS*	2e-09

FE524950	gb|EAL88703.2|	adenylate cyclase AcyA	*Aspergillus fumigatus Af293*	3e-13
FE524951	ref|XP_001264585.1|	COPII-coated vesicle protein SurF4/Erv29, putative	*Neosartorya fischeri NRRL 181*	1e-27
FE524952	-	No significant similarity	-	-
FE524953	gb|AAY66614.1|	putative secreted protein	*Ixodes scapularis*	5e-05
FE524954	pir||T02955	probable cytochrome P450 monooxygenase - maize (fragment	*Zea mays*	6e-07
FE524955	ref|XP_001267666.1|	hypothetical protein NFIA_061330	*Neosartorya fischeri NRRL 181*	1e-17
FE524956	ref|XP_001241788.1|	hypothetical protein CIMG_05684	*Coccidioides immitis RS*	5e-14
FE524957	ref|XP_001257681.1|	actin	*Neosartorya fischeri NRRL 181*	3e-42
FE524958	-	No significant similarity	-	-
FE524959	sp|P14728|YAV2_XANCV	Hypothetical 82 kDa avirulence protein in avrBs3 region	*Xanthomonas euvesicatoria*	6e-10
FE524960	ref|XP_001248928.1|	elongation factor 1-gamma	*Coccidioides immitis RS*	2e-04
FE524961	ref|XP_001276223.1|	WD domain protein	*Aspergillus clavatus NRRL 1*	2e-23
FE524962	-	No significant similarity	-	-
FE524963	-	No significant similarity	-	-
FE524964	ref|XP_001241788.1|	hypothetical protein CIMG_05684	*Coccidioides immitis RS*	5e-14
FE524965	ref|XP_001242010.1|	60S ribosomal protein L5	*Coccidioides immitis RS*	2e-46
FE524966	dbj|BAA10929.1|	cytochrome P450 like_TBP	*Nicotiana tabacum*	1e-21
FE524967	-	No significant similarity	-	-
FE524968	-	No significant similarity	-	-
FE524969	gb|AAF23950.1|	NTR	*Cercopithecine herpesvirus 12*	1e-05
FE524970	-	No significant similarity	-	-
FE524971	-	No significant similarity	-	-
FE524972	-	No significant similarity	-	-
FE524973	-	No significant similarity	-	-
FE524974	ref|XP_001241788.1|	hypothetical protein CIMG_05684	*Coccidioides immitis RS*	5e-14
FE524975	-	No significant similarity	-	-
FE524976	ref|XP_001274835.1|	60S ribosomal protein L7	*Aspergillus clavatus NRRL 1*	3e-49
FE524977	-	No significant similarity	-	-
FE524978	ref|XP_001274835.1|	60S ribosomal protein L7	*Aspergillus clavatus NRRL 1*	2e-49

FE524979	-	No significant similarity	-	-
FE524980	ref|XP_001212080.1|	enolase	*Aspergillus terreus NIH2624*	7e-04
FE524981	ref|XP_747833.1|	60s ribosomal protein L24, putative	*Aspergillus fumigatus Af293*	2e-52
FE524982	ref|XP_001271153.1|	FHA domain protein SNIP1, putative	*Aspergillus clavatus NRRL 1*	1e-09
FE524983	-	No significant similarity	-	-
FE524984	-	No significant similarity	-	-
FE524985	emb|CAJ83813.1|	CHK1 checkpoint homolog (S. pombe)	*Xenopus tropicalis*	2e-09
FE524986	ref|XP_001265902.1|	alkaline serine protease	*Neosartorya fischeri NRRL 181*	2e-12
FE524987	-	No significant similarity	-	-
FE524988	ref|XP_001265902.1|	alkaline serine protease	*Neosartorya fischeri NRRL 181*	2e-32
FE524989	-	No significant similarity	-	-
FE524990	-	No significant similarity	-	-
FE524991	-	No significant similarity	-	-
FE524992	ref|XP_752433.1|	Vacuolar ATP synthase subunit H, putative	*Aspergillus fumigatus Af293*	2e-39
FE524993	ref|XP_001268628.1|	40S ribosomal protein S25, putative	*Aspergillus clavatus NRRL 1*	2e-25
FE524994	ref|XP_001274344.1|	1,4-alpha-glucan branching enzyme	*Aspergillus clavatus NRRL 1*	3e-19
FE524995	ref|XP_001248308.1|	60S ribosomal protein L28	*Coccidioides immitis RS*	1e-48
FE524996	-	No significant similarity	-	-
FE524997	ref|XP_001248308.1|	60S ribosomal protein L28	*Coccidioides immitis RS*	2e-44
FE524998	-	No significant similarity	-	-
FE524999	gb|EEQ29198.1|	glyceraldehyde-3-phosphate dehydrogenase	*Microsporum canis CBS113480*	8e-44
FE525000	ref|XP_001267428.1|	NADH-ubiquinone oxidoreductase 21 kDa subunit, putative	*Neosartorya fischeri NRRL 181*	8e-41
FE525001	ref|XP_001268300.1|	chaperone/heat shock protein Hsp12, putative	*Aspergillus clavatus NRRL 1*	1e-23
FE525002	dbj|BAA10929.1|	cytochrome P450 like_TBP	*Nicotiana tabacum*	4e-25
FE525003	-	No significant similarity	-	-
FE525004	emb|CAD22154.1|	pherophorin-dz1 protein	*Volvox carteri f. nagariensis*	2e-13

FE525005	-	No significant similarity	-	-
FE525006	-	No significant similarity	-	-
FE525007	ref|XP_001241788.1|	hypothetical protein CIMG_05684	*Coccidioides immitis RS*	6e-12
FE525008	-	No significant similarity	-	-
FE525009	-	No significant similarity	-	-
FE525010	-	No significant similarity	-	-
FE525011	-	No significant similarity	-	-
FE525012	-	No significant similarity	-	-
FE525013	-	No significant similarity	-	-
FE525014	-	No significant similarity	-	-
FE525015	emb|CAJ83813.1|	CHK1 checkpoint homolog (S. pombe)	*Xenopus tropicalis*	3e-09
FE525016	-	No significant similarity	-	-
FE525017	-	No significant similarity	-	-
FE525018	ref|XP_001248361.1|	histone H2A	*Coccidioides immitis RS*	6e-04
FE525019	ref|XP_001254275.1|	PREDICTED: hypothetical protein	*Bos taurus*	3e-15
FE525020	-	No significant similarity	-	-
FE525021	-	No significant similarity	-	-
FE525022	ref|XP_001266185.1|	nucleoside diphosphate kinase	*Neosartorya fischeri NRRL 181*	2e-52
FE525023	dbj|BAD26579.1|	cytochrome P450 like_TBP	*Citrullus lanatus*	2e-07
FE525024	ref|XP_001241788.1|	hypothetical protein CIMG_05684	*Coccidioides immitis RS*	3e-11
FE525025	-	No significant similarity	-	-
FE525026	-	No significant similarity	-	-
FE525027	gb|AAC13689.1|	ubiquitin fusion protein	*Magnaporthe grisea*	1e-38
FE525028	-	No significant similarity	-	-
FE525029	-	No significant similarity	-	-
FE525030	-	No significant similarity	-	-
FE525031	ref|XP_001258304.1|	benzoate 4-monooxygenase cytochrome P450	*Neosartorya fischeri NRRL 181*	3e-09
FE525032	ref|XP_001259888.1|	ABC metal ion transporter, putative	*Neosartorya fischeri NRRL 181*	1e-15
FE525033	ref|XP_001241788.1|	hypothetical protein CIMG_05684	*Coccidioides immitis RS*	4e-14
FE525034	ref|XP_001241788.1|	hypothetical protein CIMG_05684	*Coccidioides immitis RS*	3e-12
FE525035	ref|XP_001261370.1|	Ribosomal protein S28e	*Neosartorya fischeri NRRL 181*	9e-24
FE525036	-	No significant similarity	-	-
FE525037	ref|XP_001257579.1|	2-methylcitrate dehydratase, putative	*Neosartorya fischeri NRRL 181*	5e-42

FE525038	-	No significant similarity	-	-
FE525039	sp|Q9C3Z6|RLA0_PODAN	60S acidic ribosomal protein P0 gb|AAK11262.1|AF331714_1 ribosomal protein P0	*Podospora anserina*	1e-05
FE525040	gb|ABH10639.1|	fructose biphosphate aldolase	*Coccidioides posadasii*	1e-59
FE525041	-	No significant similarity	-	-
FE525042	ref|XP_001241788.1|	hypothetical protein CIMG_05684	*Coccidioides immitis RS*	5e-14
FE525043	-	No significant similarity	-	-
FE525044	-	No significant similarity	-	-
FE525045	-	No significant similarity	-	-
FE525046	-	No significant similarity	-	-
FE525047	ref|XP_001264877.1|	heavy metal ion transporter, putative	*Neosartorya fischeri NRRL 181*	1e-10
FE525048	ref|XP_001247280.1|	alternative oxidase, mitochondrial precursor	*Coccidioides immitis RS*	1e-04
FE525049	-	No significant similarity	-	-
FE525050	ref|XP_001244689.1|	hypothetical protein CIMG_04130	*Coccidioides immitis RS*	8e-05
FE525051	-	No significant similarity	-	-
FE525052	-	No significant similarity	-	-
FE525053	ref|NP_173553.1|	ATEXT3 (EXTENSIN 3); structural constituent of cell wall	*Arabidopsis thaliana*	4e-05
FE525054	-	No significant similarity	-	-
FE525055	-	No significant similarity	-	-
FE525056	ref|XP_001241788.1|	hypothetical protein CIMG_05684	*Coccidioides immitis RS*	1e-13
FE525057	ref|XP_001267676.1|	hypothetical protein NFIA_043490	*Neosartorya fischeri NRRL 181*	2e-06
FE525058	-	No significant similarity	-	-
FE525059	ref|XP_001265868.1|	ribosomal protein L34 protein, putative	*Neosartorya fischeri NRRL 181*	1e-07
FE525060	-	No significant similarity	-	-
FE525061	-	No significant similarity	-	-
FE525062	-	No significant similarity	-	-
FE525063	gb|EEQ28799.1|	alpha 1,6 mannosyltransferase	*Microsporum canis CBS 113480*	2e-18
FE525064	-	No significant similarity	-	-
FE525065	-	No significant similarity	-	-
FE525066	-	No significant similarity	-	-
FE525067	ref|XP_001261287.1|	tetratricopeptide repeat domain protein	*Neosartorya fischeri NRRL 181*	2e-05
FE525068	-	No significant similarity	-	-
FE525069	-	No significant similarity	-	-

FE525070	-	No significant similarity	-	-
FE525071	-	No significant similarity	-	-
FE525072	ref|XP_001241788.1|	hypothetical protein CIMG_05684	*Coccidioides immitis RS*	3e-12
FE525073	-	No significant similarity	-	-
FE525074	-	No significant similarity	-	-
FE525075	ref|XP_001272337.1|	AIF-like mitochondrial oxidoreductase (Nfrl), putative	*Aspergillus clavatus NRRL 1*	3e-17
FE525076	-	No significant similarity	-	-
FE525077	dbj|BAB12232.1|	fructose 1,6-bisphosphate aldolase	*Aspergillus oryzae*	2e-09
FE525078	ref|XP_001275347.1|	40S ribosomal protein S9	*Aspergillus clavatus NRRL 1*	8e-41
FE525079	ref|XP_001268220.1|	TBC domain protein, putative	*Aspergillus clavatus NRRL 1*	1e-12
FE525080	-	No significant similarity	-	-
FE525081	-	No significant similarity	-	-
FE525082	-	No significant similarity	-	-
FE525083	-	No significant similarity	-	-
FE525084	-	No significant similarity	-	-
FE525085	-	No significant similarity	-	-
FE525086	ref|XP_001264756.1|	adenosylhomocysteinase	*Neosartorya fischeri NRRL 181*	1e-60
FE525087	-	No significant similarity	-	-
FE525088	-	No significant similarity	-	-
FE525089	-	No significant similarity	-	-
FE525090	-	No significant similarity	-	-
FE525091	-	No significant similarity	-	-
FE525092	-	No significant similarity	-	-
FE525093	-	No significant similarity	-	-
FE525094	-	No significant similarity	-	-
FE525095	-	No significant similarity	-	-
FE525096	ref|XP_001246644.1|	protein translation factor SUI1	*Coccidioides immitis RS*	2e-41
FE525097	-	No significant similarity	-	-
FE525098	-	No significant similarity	-	-
FE525099	-	No significant similarity	-	-
FE525100	-	No significant similarity	-	-
FE525101	-	No significant similarity	-	-
FE525102	ref|XP_001263624.1|	eukaryotic translation initiation factor 4, putative	*Neosartorya fischeri NRRL 181*	2e-25

FE525103	gb|AAQ10290.1|	class V chitin synthase	*Coccidioides posadasii*	5e-06
FE525104	ref|XP_001241788.1|	hypothetical protein CIMG_05684	*Coccidioides immitis RS*	8e-13
FE525105	-	No significant similarity	-	-
FE525106	ref|XP_001268551.1|	conidial hydrophobin RodB	*Aspergillus clavatus NRRL 1*	5e-05
FE525107	ref|XP_001276223.1|	WD domain protein	*Aspergillus clavatus NRRL 1*	8e-25
FE525108	-	No significant similarity	-	-
FE525109	-	No significant similarity	-	-
FE525110	ref|XP_747995.1|	nucleoside diphosphate kinase	*Aspergillus fumigatus Af293*	2e-32
FE525111	gb|EAW96235.1|	hCG2043380	*Homo sapiens*	1e-06
FE525112	-	No significant similarity	-	-
FE525113	-	No significant similarity	-	-
FE525114	ref|XP_001243230.1|	40S ribosomal protein S23	*Coccidioides immitis RS*	1e-19
FE525115	-	No significant similarity	-	-
FE525116	ref|XP_001274770.1|	60S ribosomal protein L13	*Aspergillus clavatus NRRL 1*	4e-12
FE525117	-	No significant similarity	-	-
FE525118	ref|YP_293958.1|	putative membrane protein	*Emiliania huxleyi virus 86*	8e-08
FE525119	ref|XP_001240069.1|	ATP-citrate synthase subunit 1	*Coccidioides immitis RS*	3e-28
FE525120	-	No significant similarity	-	-
FE525121	-	No significant similarity	-	-
FE525122	-	No significant similarity	-	-
FE525123	-	No significant similarity	-	-
FE525124	gb|AAO52807.1|	hypothetical protein	*Bacillus megaterium*	2e-04
FE525125	ref|XP_001260256.1|	replication factor A 1, rfa1	*Neosartorya fischeri NRRL 181*	1e-22
FE525126	-	No significant similarity	-	-
FE525127	ref|XP_001271667.1|	60S ribosomal protein L11	*Aspergillus clavatus NRRL 1*	3e-67
FE525128	ref|XP_001246273.1|	ADP-ribosylation factor 6	*Coccidioides immitis RS*	5e-21
FE525129	-	No significant similarity	-	-
FE525130	-	No significant similarity	-	-
FE525131	ref|XP_001210992.1|	homocitrate synthase, mitochondrial precursor	*Aspergillus terreus NIH2624*	1e-80
FE525132	-	No significant similarity	-	-

FE525133	-	No significant similarity	-	-
FE525134	gb|AAL09828.1|	beta-glucosidase 4	*Coccidioides immitis*	1e-16
FE525135	-	No significant similarity	-	-
FE525136	ref|XP_001241788.1|	hypothetical protein CIMG_05684	*Coccidioides immitis RS*	7e-12
FE525137	gb|AAX33296.1|	heat shock protein 90	*Paracoccidioides brasiliensis*	1e-31
FE525138	emb|CAK47870.1|	unnamed protein product	*Aspergillus niger*	3e-10
FE525139	-	No significant similarity	-	-
FE525140	gb|EAT87923.1|	hypothetical protein SNOG_04163	*Phaeosphaeria nodorum SN15*	3e-14
FE525141	-	No significant similarity	-	-
FE525142	-	No significant similarity	-	-
FE525143	ref|XP_001276036.1|	sulfatase domain protein	*Aspergillus clavatus NRRL 1*	9e-18
FE525144	gb|ABA33785.1|	RACK1-like protein	*Paracoccidioides brasiliensis*	2e-42
FE525145	-	No significant similarity	-	-
FE525146	-	No significant similarity	-	-
FE525147	-	No significant similarity	-	-
FE525148	-	No significant similarity	-	-
FE525149	-	No significant similarity	-	-
FE525150	-	No significant similarity	-	-
FE525151	ref|XP_001269061.1|	glycine cleavage system H protein	*Aspergillus clavatus NRRL 1*	5e-27
FE525152	emb|CAM67156.1|	Hypothetical repeat protein	*Leishmania infantum*	5e-06
FE525153	-	No significant similarity	-	-
FE525154	-	No significant similarity	-	-
FE525155	ref|XP_729762.1|	senescence-associated protein	*Plasmodium yoelii yoelii str. 17XNL*	4e-12
FE525156	-	No significant similarity	-	-
FE525157	-	No significant similarity	-	-
FE525158	-	No significant similarity	-	-
FE525159	-	No significant similarity	-	-
FE525160	-	No significant similarity	-	-
FE525161	-	No significant similarity	-	-
FE525162	-	No significant similarity	-	-
FE525163	-	No significant similarity	-	-
FE525164	-	No significant similarity	-	-

FE525165	ref|XP_001214063.1|	cytochrome c1, mitochondrial precursor	*Aspergillus terreus NIH2624*	8e-14
FE525166	ref|XP_755078.1|	fimbrin	*Aspergillus fumigatus Af293*	1e-13
FE525167	ref|XP_001248022.1|	threonyl-tRNA synthetase, cytoplasmic	*Coccidioides immitis RS*	7e-09
FE525168	-	No significant similarity	-	-
FE525169	-	No significant similarity	-	-
FE525170	-	No significant similarity	-	-
FE525171	-	No significant similarity	-	-
FE525172	ref|XP_001269707.1|	Ribosomal L18ae protein family	*Aspergillus clavatus NRRL 1*	5e-05
FE525173	-	No significant similarity	-	-
FE525174	-	No significant similarity	-	-
FE525175	gb|AAK13589.1|	rRNA intron-encoded homing endonuclease	*Oryza sativa*	3e-05
FE525176	-	No significant similarity	-	-
FE525177	-	No significant similarity	-	-
FE525178	ref|XP_001241788.1|	hypothetical protein CIMG_05684	*Coccidioides immitis RS*	1e-09
FE525179	gb|EDJ94901.1|	nucleoside diphosphate kinase	*Magnaporthe grisea 70-15*	3e-20
FE525180	ref|XP_001272046.1|	splicing factor 3a subunit 2, putative	*Aspergillus clavatus NRRL 1*	1e-10
FE525181	ref|XP_001241788.1|	hypothetical protein CIMG_05684	*Coccidioides immitis RS*	5e-08
FE525182	-	No significant similarity	-	-
FE525183	-	No significant similarity	-	-
FE525184	-	No significant similarity	-	-
FE525185	ref|XP_623110.1|	PREDICTED: similar to CG3195-PA, isoform A	*Apis mellifera*	3e-21
FE525186	ref|XP_001241788.1|	hypothetical protein CIMG_05684	*Coccidioides immitis RS*	3e-12
FE525187	gb|AAC78304.1|	ubiquitin/ribosomal fusion protein	*Schistosoma japonicum*	3e-20
FE525188	ref|XP_001243274.1|	hypothetical protein CIMG_07170	*Coccidioides immitis RS*	1e-08
FE525189	-	No significant similarity	-	-
FE525190	ref|XP_001241788.1|	hypothetical protein CIMG_05684	*Coccidioides immitis RS*	7e-13
FE525191	ref|XP_001239918.1|	hypothetical protein CIMG_09539	*Coccidioides immitis RS*	8e-35
FE525192	gb|ABA40770.1|	SJCHGC01393 protein	*Schistosoma japonicum*	6e-04
FE525193	emb|CAB51041.1|	putative transcription factor	*Periplaneta americana*	4e-14
FE525194	-	No significant similarity	-	-
FE525195	-	No significant similarity	-	-
FE525196	-	No significant similarity	-	-
FE525197	ref|XP_001247341.1|	hypothetical protein CIMG_01112	*Coccidioides immitis RS*	5e-18

FE525198	dbj|BAE65360.1|	unnamed protein product	*Aspergillus oryzae*	4e-04
FE525199	-	No significant similarity	-	-
FE525200	-	No significant similarity	-	-
FE525201	ref|XP_001257727.1|	TOM complex component Tom7, putative	*Neosartorya fischeri NRRL 181*	1e-13
FE525202	-	No significant similarity	-	-
FE525203	ref|XP_001273715.1|	acyl-CoA dehydrogenase, putative	*Aspergillus clavatus NRRL 1*	2e-17
FE525204	-	No significant similarity	-	-
FE525205	ref|XP_001269192.1|	proteasome regulatory particle subunit (Nas6), putative	*Aspergillus clavatus NRRL 1*	2e-10
FE525206	ref|XP_001265788.1|	UDP-glucose:sterol glycosyltransferase	*Neosartorya fischeri NRRL 181*	4e-05
FE525207	ref|XP_965645.1|	ATP synthase alpha chain, mitochondrial precursor	*Neurospora crassa OR74A*	4e-28
FE525208	ref|XP_001274770.1|	60S ribosomal protein L13	*Aspergillus clavatus NRRL 1*	2e-10
FE525209	-	No significant similarity	-	-
FE525210	sp|P0C2C8|ATP7_ASPTN	ATP synthase D chain, mitochondrial precursor	*Aspergillus terreus NIH2624*	4e-11
FE525211	-	No significant similarity	-	-
FE525212	ref|XP_001213339.1|	glutathione peroxidase	*Aspergillus terreus NIH2624*	5e-12
FE525213	-	No significant similarity	-	-
FE525214	-	No significant similarity	-	-
FE525215	ref|XP_001267676.1|	hypothetical protein NFIA_043490	*Neosartorya fischeri NRRL 181*	4e-08
FE525216	-	No significant similarity	-	-
FE525217	-	No significant similarity	-	-
FE525218	-	No significant similarity	-	-
FE525219	-	No significant similarity	-	-
FE525220	-	No significant similarity	-	-
FE525221	-	No significant similarity	-	-
FE525222	ref|XP_568855.1|	glucose transporter	*Cryptococcus neoformans var. neoformans JEC21*	2e-22
FE525223	-	No significant similarity	-	-
FE525224	-	No significant similarity	-	-

FE525225	-	No significant similarity	-	-
FE525226	-	No significant similarity	-	-
FE525227	ref|XP_001214668.1|	acetolactate synthase small subunit, mitochondrial precursor	*Aspergillus terreus NIH2624*	3e-05
FE525228	-	No significant similarity	-	-
FE525229	-	No significant similarity	-	-
FE525230	-	No significant similarity	-	-
FE525231	-	No significant similarity	-	-
FE525232	-	No significant similarity	-	-
FE525233	emb|CAJ83813.1|	CHK1 checkpoint homolog (S. pombe)	*Xenopus tropicalis*	2e-09
FE525234	ref|XP_001268551.1|	conidial hydrophobin RodB	*Aspergillus clavatus NRRL 1*	2e-04
FE525235	-	No significant similarity	-	-
FE525236	ref|XP_001275026.1|	oligopeptide transporter	*Aspergillus clavatus NRRL 1*	2e-27
FE525237	-	No significant similarity	-	-
FE525238	-	No significant similarity	-	-
FE525239	ref|XP_001241788.1|	hypothetical protein CIMG_05684	*Coccidioides immitis RS*	2e-13
FE525240	-	No significant similarity	-	-
FE525241	-	No significant similarity	-	-
FE525242	emb|CAJ83813.1|	CHK1 checkpoint homolog (S. pombe)	*Xenopus tropicalis*	2e-09
FE525243	-	No significant similarity	-	-
FE525244	-	No significant similarity	-	-
FE525245	-	No significant similarity	-	-
FE525246	sp|P56205|CYC_ASPNG	Cytochrome c	*Aspergillus niger*	1e-11
FE525247	gb|EAL85176.2|	glucosamine-6-phosphate deaminase, putative	*Aspergillus fumigatus Af293*	6e-25
FE525248	-	No significant similarity	-	-
FE525249	tpd|FAA00317.1|	TPA: polyubiquitin	*Aspergillus nidulans FGSC A4*	1e-18
FE525250	emb|CAJ83813.1|	CHK1 checkpoint homolog (S. pombe)	*Xenopus tropicalis*	2e-09
FE525251	ref|XP_001241788.1|	hypothetical protein CIMG_05684	*Coccidioides immitis RS*	1e-05
FE525252	ref|XP_752345.1|	stomatin family protein	*Aspergillus fumigatus Af293*	2e-06
FE525253	ref|XP_001246620.1|	40S ribosomal protein S4	*Coccidioides immitis RS*	3e-14
FE525254	ref|XP_001264282.1|	transcription factor TFIIA complex subunit Toa1, putative	*Neosartorya fischeri NRRL 181*	1e-05
FE525255	ref|XP_001266185.1|	nucleoside diphosphate kinase	*Neosartorya fischeri*	2e-16

			NRRL 181	
FE525256	-	No significant similarity	-	-
FE525257	-	No significant similarity	-	-
FE525258	-	No significant similarity	-	-
FE525259	-	No significant similarity	-	-
FE525260	-	No significant similarity	-	-
FE525261	emb|CAJ83813.1|	CHK1 checkpoint homolog (S. pombe)	*Xenopus tropicalis*	1e-08
FE525262	ref|XP_001132700.1|	PREDICTED: hypothetical protein	*Homo sapiens*	4e-17
FE525263	ref|XP_001267666.1|	hypothetical protein NFIA_061330	*Neosartorya fischeri NRRL 181*	6e-08
FE525264	ref|XP_001241788.1|	hypothetical protein CIMG_05684	*Coccidioides immitis RS*	2e-08
FE525265	ref|XP_001217121.1|	60S ribosomal protein L9-B	*Aspergillus terreus NIH2624*	2e-12
FE525266	-	No significant similarity	-	-
FE525267	-	No significant similarity	-	-
FE525268	-	No significant similarity	-	-
FE525269	-	No significant similarity	-	-
FE525270	-	No significant similarity	-	-
FE525271	-	No significant similarity	-	-
FE525272	ref|XP_001266191.1|	PHD transcription factor (Rum1), putative	*Neosartorya fischeri NRRL 181*	6e-17
FE525273	-	No significant similarity	-	-
FE525274	ref|XP_001267282.1|	60S ribosomal protein L36	*Neosartorya fischeri NRRL 181*	7e-05
FE525275	-	No significant similarity	-	-
FE525276	-	No significant similarity	-	-
FE525277	emb|CAD56870.1|	BclA protein	*Bacillus anthracis*	2e-06
FE525278	gb|EAT89258.1|	hypothetical protein SNOG_04053	*Phaeosphaeria nodorum SN15*	2e-11
FE525279	-	No significant similarity	-	-
FE525280	-	No significant similarity	-	-
FE525281	-	No significant similarity	-	-
FE525282	-	No significant similarity	-	-
FE525283	-	No significant similarity	-	-
FE525284	-	No significant similarity	-	-
FE525285	gb|ABH10645.1|	ATP synthase beta chain	*Coccidioides posadasii*	2e-15
FE525286	-	No significant similarity	-	-
FE525287	-	No significant similarity	-	-

FE525288	-	No significant similarity	-	-
FE525289	-	No significant similarity	-	-
FE525290	-	No significant similarity	-	-
FE525291	-	No significant similarity	-	-
FE525292	-	No significant similarity	-	-
FE525293	-	No significant similarity	-	-
FE525294	-	No significant similarity	-	-
FE525295	-	No significant similarity	-	-
FE525296	ref|XP_001246341.1|	probable cation-transporting ATPase	*Coccidioides immitis RS*	6e-24
FE525297	-	No significant similarity	-	-
FE525298	-	No significant similarity	-	-
FE525299	-	No significant similarity	-	-
FE525300	ref|XP_001241788.1|	hypothetical protein CIMG_05684	*Coccidioides immitis RS*	5e-10
FE525301	-	No significant similarity	-	-
FE525302	-	No significant similarity	-	-
FE525303	-	No significant similarity	-	-
FE525304	ref|XP_001264792.1|	aldehyde reductase (AKR1), putative	*Neosartorya fischeri NRRL 181*	1e-25
FE525305	-	No significant similarity	-	-
FE525306	-	No significant similarity	-	-
FE525307	-	No significant similarity	-	-
FE525308	-	No significant similarity	-	-
FE525309	-	No significant similarity	-	-
FE525310	-	No significant similarity	-	-
FE525311	-	No significant similarity	-	-
FE525312	-	No significant similarity	-	-
FE525313	ref|XP_001261298.1|	sugar transporter	*Neosartorya fischeri NRRL 181*	4e-24
FE525314	ref|XP_001241788.1|	hypothetical protein CIMG_05684	*Coccidioides immitis RS*	7e-15
FE525315	-	No significant similarity	-	-
FE525316	-	No significant similarity	-	-
FE525317	ref|XP_001240403.1|	proteasome component precursor	*Coccidioides immitis RS*	5e-17
FE525318	-	No significant similarity	-	-
FE525319	ref|XP_001268304.1|	BolA domain protein	*Aspergillus clavatus NRRL 1*	9e-15
FE525320	gb|AAG01549.3|AF291822_1	multidrug resistance protein MDR	*Trichophyton rubrum*	2e-07
FE525321	-	No significant similarity	-	-
FE525322	-	No significant similarity	-	-

FE525323	-	No significant similarity	-	-
FE525324	ref|XP_001241788.1|	hypothetical protein CIMG_05684	*Coccidioides immitis RS*	5e-14
FE525325	ref|XP_001209805.1|	2-methylcitrate synthase, mitochondrial precursor	*Aspergillus terreus NIH2624*	2e-19
FE525326	gb|EDK06386.1|	40S ribosomal protein S3	*Magnaporthe grisea 70-15*	2e-17
FE525327	-	No significant similarity	-	-
FE525328	ref|XP_001259371.1|	chorismate mutase	*Neosartorya fischeri NRRL 181*	7e-08
FE525329	-	No significant similarity	-	-
FE525330	gb|ABB18373.1|	chitobiase	*Coccidioides posadasii*	4e-14
FE525331	-	No significant similarity	-	-
FE525332	ref|XP_001244490.1|	ADP,ATP carrier protein	*Coccidioides immitis RS*	4e-22
FE525333	-	No significant similarity	-	-
FE525334	ref|XP_001271628.1|	5-methyltetrahydropteroyltriglutamate--homocysteine S-methyltransferase	*Aspergillus clavatus NRRL 1*	9e-05
FE525335	ref|XP_001265922.1|	IMP dehydrogenase, putative	*Neosartorya fischeri NRRL 181*	4e-26
FE525336	-	No significant similarity	-	-
FE525337	ref|XP_001241788.1|	hypothetical protein CIMG_05684	*Coccidioides immitis RS*	2e-09
FE525338	-	No significant similarity	-	-
FE525339	-	No significant similarity	-	-
FE525340	-	No significant similarity	-	-
FE525341	-	No significant similarity	-	-
FE525342	-	No significant similarity	-	-
FE525343	-	No significant similarity	-	-
FE525344	ref|XP_001239885.1|	60S ribosomal protein L32	*Coccidioides immitis RS*	1e-15
FE525345	ref|XP_001151718.1|	PREDICTED: hypothetical protein isoform 2	*Pan troglodytes*	5e-08
FE525346	-	No significant similarity	-	-
FE525347	-	No significant similarity	-	-
FE525348	gb|ABI52743.1|	10 kDa putative secreted protein	*Argas monolakensis*	3e-05
FE525349	-	No significant similarity	-	-
FE525350	-	No significant similarity	-	-
FE525351	sp|Q9P4E9|GSP1_CANAL	GTP-binding nuclear protein GSP1/Ran gb|AAF78478.1|AF190700_1 small G-protein Gsp1p	*Candida albicans*	7e-24
FE525352	gb|EAT91215.1|	predicted protein	*Phaeosphaeria nodorum SN15*	5e-09
FE525353	-	No significant similarity	-	-

FE525354	gb|AAF23950.1|	NTR	*Cercopithecine herpesvirus 12*	4e-09
FE525355	-	No significant similarity	-	-
FE525356	-	No significant similarity	-	-
FE525357	ref|XP_001241788.1|	hypothetical protein CIMG_05684	*Coccidioides immitis RS*	5e-14
FE525358	-	No significant similarity	-	-
FE525359	ref|XP_001214052.1|	GTP-binding protein ypt3	*Aspergillus terreus NIH2624*	1e-05
FE525360	-	No significant similarity	-	-
FE525361	ref|XP_001193920.1|	PREDICTED: hypothetical protein, partial	*Strongylocentrotus purpuratus*	3e-05
FE525362	-	No significant similarity	-	-
FE525363	-	No significant similarity	-	-
FE525364	-	No significant similarity	-	-
FE525365	-	No significant similarity	-	-
FE525366	ref|XP_751123.1|	12 kDa heat shock protein	*Aspergillus fumigatus Af293*	5e-10
FE525367	ref|XP_001241783.1|	hypothetical protein CIMG_05679	*Coccidioides immitis RS*	5e-06
FE525368	ref|XP_001243753.1|	60S ribosomal protein L17	*Coccidioides immitis RS*	4e-24
FE525369	ref|XP_001266324.1|	UPF0047 domain protein	*Neosartorya fischeri NRRL 181*	2e-23
FE525370	-	No significant similarity	-	-
FE525371	dbj|BAE73006.1|	hypothetical protein	*Macaca fascicularis*	1e-06
FE525372	-	No significant similarity	-	-
FE525373	-	No significant similarity	-	-
FE525374	ref|XP_001241788.1|	hypothetical protein CIMG_05684	*Coccidioides immitis RS*	1e-12
FE525375	-	No significant similarity	-	-
FE525376	-	No significant similarity	-	-
FE525377	gb|AAO73810.2|	heat shock protein CLPA	*Paracoccidioides brasiliensis*	7e-06
FE525378	-	No significant similarity	-	-
FE525379	-	No significant similarity	-	-
FE525380	-	No significant similarity	-	-
FE525381	-	No significant similarity	-	-
FE525382	ref|XP_001270964.1|	extracellular thaumatin domain protein, putative	*Aspergillus clavatus NRRL 1*	1e-08
FE525383	ref|XP_001241788.1|	hypothetical protein CIMG_05684	*Coccidioides immitis RS*	1e-11
FE525384	-	No significant similarity	-	-

FE525385	ref|XP_001246338.1|	eukaryotic translation initiation factor 2 alpha subunit (eIF-2-alpha)	*Coccidioides immitis RS*	1e-18
FE525386	-	No significant similarity	-	-
FE525387	ref|XP_001241788.1|	hypothetical protein CIMG_05684	*Coccidioides immitis RS*	1e-04
FE525388	ref|XP_001268377.1|	60S ribosomal protein L31e	*Aspergillus clavatus NRRL 1*	4e-27
FE525389	-	No significant similarity	-	-
FE525390	-	No significant similarity	-	-
FE525391	-	No significant similarity	-	-
FE525392	-	No significant similarity	-	-
FE525393	ref|XP_001259633.1|	ATP synthase F1, beta subunit, putative	*Neosartorya fischeri NRRL 181*	9e-22
FE525394	gb|ABF13477.1|	DRK1 histidine kinase	*Ajellomyces dermatitidis*	8e-19
FE525395	ref|XP_755487.1|	transmembrane domain-containing protein	*Aspergillus fumigatus Af293*	1e-11
FE525396	-	No significant similarity	-	-
FE525397	-	No significant similarity	-	-
FE525398	-	No significant similarity	-	-
FE525399	-	No significant similarity	-	-
FE525400	-	No significant similarity	-	-
FE525401	ref|XP_001247329.1|	tyrosyl-tRNA synthetase, cytoplasmic	*Coccidioides immitis RS*	9e-06
FE525402	ref|XP_753079.1|	long chain fatty alcohol oxidase, putative	*Aspergillus fumigatus Af293*	2e-07
FE525403	-	No significant similarity	-	-
FE525404	gb|ABF13597.1|	dermatan-binding protein PA5541	*Propionibacterium acnes*	7e-06
FE525405	ref|XP_001209812.1|	malate synthase	*Aspergillus terreus NIH2624*	8e-32
FE525406	-	No significant similarity	-	-
FE525407	gb|EAL84560.2|	protein kinase activator Bem1, putative	*Aspergillus fumigatus Af293*	6e-06
FE525408	emb|CAJ83813.1|	CHK1 checkpoint homolog (S. pombe)	*Xenopus tropicalis*	3e-09
FE525409	ref|XP_001212958.1|	40S ribosomal protein S8-B	*Aspergillus terreus NIH2624*	7e-09
FE525410	gb|AAM54368.1|	elongation factor 1-alpha	*Trichophyton rubrum*	1e-46
FE525411	-	No significant similarity	-	-
FE525412	-	No significant similarity	-	-
FE525413	-	No significant similarity	-	-
FE525414	-	No significant similarity	-	-

FE525415	ref|XP_001267676.1|	hypothetical protein NFIA_043490	*Neosartorya fischeri NRRL 181*	1e-04
FE525416	-	No significant similarity	-	-
FE525417	-	No significant similarity	-	-
FE525418	-	No significant similarity	-	-
FE525419	-	No significant similarity	-	-
FE525420	-	No significant similarity	-	-
FE525421	emb|CAJ83813.1|	CHK1 checkpoint homolog (S. pombe)	*Xenopus tropicalis*	2e-09
FE525422	ref|XP_001267290.1|	5-methyltetrahydropteroyltriglutamate--homocysteine S-methyltransferase	*Neosartorya fischeri NRRL 181*	6e-34
FE525423	ref|XP_001241783.1|	hypothetical protein CIMG_05679	*Coccidioides immitis RS*	3e-07
FE525424	-	No significant similarity	-	-
FE525425	ref|XP_001271804.1|	cytochrome c peroxidase Ccp1, putative	*Aspergillus clavatus NRRL 1*	4e-37
FE525426	ref|XP_001266962.1|	transcription regulator BDF1, putative	*Neosartorya fischeri NRRL 181*	4e-08
FE525427	ref|YP_288338.1|	Surface protein from Gram-positive cocci, anchor region	*Thermobifida fusca YX*	4e-04
FE525428	ref|XP_568822.1|	UDP-glucose:glycoprotein glucosyltransferase	*Cryptococcus neoformans var. neoformans JEC21*	9e-38
FE525429	-	No significant similarity	-	-
FE525430	-	No significant similarity	-	-
FE525431	-	No significant similarity	-	-
FE525432	-	No significant similarity	-	-
FE525433	-	No significant similarity	-	-
FE525434	emb|CAJ83813.1|	CHK1 checkpoint homolog (S. pombe)	*Xenopus tropicalis*	2e-09
FE525435	-	No significant similarity	-	-
FE525436	-	No significant similarity	-	-
FE525437	-	No significant similarity	-	-
FE525438	ref|XP_001215933.1|	asparaginyl-tRNA synthetase	*Aspergillus terreus NIH2624*	2e-50
FE525439	-	No significant similarity	-	-
FE525440	-	No significant similarity	-	-
FE525441	-	No significant similarity	-	-
FE525442	ref|XP_868852.1|	uricase	*Aspergillus nidulans FGSC A4*	6e-35
FE525443	-	No significant similarity	-	-
FE525444	ref|XP_001241788.1|	hypothetical protein CIMG_05684	*Coccidioides immitis RS*	9e-07

FE525445	-	No significant similarity	-	-
FE525446	emb|CAJ83813.1|	CHK1 checkpoint homolog (S. pombe)	*Xenopus tropicalis*	2e-09
FE525447	-	No significant similarity	-	-
FE525448	ref|XP_001267336.1|	outer mitochondrial membrane protein porin	*Neosartorya fischeri NRRL 181*	5e-11
FE525449	-	No significant similarity	-	-
FE525450	-	No significant similarity	-	-
FE525451	-	No significant similarity	-	-
FE525452	gb|AAO52807.1|	hypothetical protein	*Bacillus megaterium*	9e-05
FE525453	ref|XP_001267336.1|	outer mitochondrial membrane protein porin	*Neosartorya fischeri NRRL 181*	3e-09
FE525454	-	No significant similarity	-	-
FE525455	-	No significant similarity	-	-
FE525456	ref|XP_001247752.1|	hypothetical protein CIMG_01523	*Coccidioides immitis RS*	6e-20
FE525457	-	No significant similarity	-	-
FE525458	-	No significant similarity	-	-
FE525459	ref|XP_001241788.1|	hypothetical protein CIMG_05684	*Coccidioides immitis RS*	4e-13
FE525460	ref|XP_001241788.1|	hypothetical protein CIMG_05684	*Coccidioides immitis RS*	3e-12
FE525461	ref|XP_001247341.1|	hypothetical protein CIMG_01112	*Coccidioides immitis RS*	4e-23
FE525462	-	No significant similarity	-	-
FE525463	-	No significant similarity	-	-
FE525464	-	No significant similarity	-	-
FE525465	-	No significant similarity	-	-
FE525466	-	No significant similarity	-	-
FE525467	ref|XP_001245555.1|	eukaryotic translation initiation factor 5A-2	*Coccidioides immitis RS*	2e-05
FE525468	-	No significant similarity	-	-
FE525469	-	No significant similarity	-	-
FE525470	-	No significant similarity	-	-
FE525471	ref|XP_001258207.1|	short chain dehydrogenase/reductase family	*Neosartorya fischeri NRRL 181*	5e-05
FE525472	ref|XP_001210407.1|	elongation factor 1-beta	*Aspergillus terreus NIH2624*	7e-12
FE525473	-	No significant similarity	-	-
FE525474	-	No significant similarity	-	-
FE525475	-	No significant similarity	-	-
FE525476	ref|XP_001241788.1|	hypothetical protein CIMG_05684	*Coccidioides immitis RS*	5e-14
FE525477	-	No significant similarity	-	-
FE525478	-	No significant similarity	-	-

FE525479	-	No significant similarity	-	-
FE525480	-	No significant similarity	-	-
FE525481	ref|XP_001257958.1|	ubiquinol cytochrome-c reductase subunit 9, putative	*Neosartorya fischeri NRRL 181*	2e-17
FE525482	ref|XP_001260275.1|	serine/threonine protein kinase (Ark1), putative	*Neosartorya fischeri NRRL 181*	8e-35
FE525483	ref|XP_001229506.1|	60S ribosomal protein L29	*Chaetomium globosum CBS 148.51*	7e-28
FE525484	ref|NP_941181.1|	chloramphenicol acetyltransferase	*Serratia marcescens*	4e-05
FE525485	-	No significant similarity	-	-
FE525486	-	No significant similarity	-	-
FE525487	-	No significant similarity	-	-
FE525488	-	No significant similarity	-	-
FE525489	ref|XP_001240451.1|	aspartyl aminopeptidase, putative	*Coccidioides immitis RS*	1e-08
FE525490	ref|XP_752593.1|	mitochondrial hypoxia responsive domain protein	*Aspergillus fumigatus Af293*	3e-30
FE525491	ref|XP_001264032.1|	conidial hydrophobin RodB	*Neosartorya fischeri NRRL 181*	7e-07
FE525492	-	No significant similarity	-	-
FE525493	-	No significant similarity	-	-
FE525494	-	No significant similarity	-	-
FE525495	-	No significant similarity	-	-
FE525496	-	No significant similarity	-	-
FE525497	gb|AAL31950.1|	CDH1-D	*Gallus gallus*	3e-11
FE525498	-	No significant similarity	-	-
FE525499	ref|XP_749092.1|	integral peroxisomal membrane protein	*Aspergillus fumigatus Af293*	4e-24
FE525500	-	No significant similarity	-	-
FE525501	ref|XP_001265155.1|	COPII-coated vesicle membrane protein Erv46, putative	*Neosartorya fischeri NRRL 181*	9e-33
FE525502	ref|XP_001241788.1|	hypothetical protein CIMG_05684	*Coccidioides immitis RS*	2e-13
FE525503	ref|XP_001241599.1|	predicted protein	*Coccidioides immitis RS*	6e-11
FE525504	-	No significant similarity	-	-
FE525505	-	No significant similarity	-	-
FE525506	pir||T02955	probable cytochrome P450 monooxygenase - maize (fragment)	*Zea mays*	6e-07
FE525507	ref|XP_001241788.1|	hypothetical protein CIMG_05684	*Coccidioides immitis RS*	5e-14
FE525508	-	No significant similarity	-	-
FE525509	ref|XP_001269838.1|	6-phosphogluconolactonase	*Aspergillus clavatus*	2e-34

			NRRL 1	
FE525510	-	No significant similarity	-	-
FE525511	ref|XP_001241788.1|	hypothetical protein CIMG_05684	*Coccidioides immitis RS*	1e-12
FE525512	ref|XP_001267282.1|	60S ribosomal protein L36	*Neosartorya fischeri NRRL 181*	7e-26
FE525513	ref|XP_453838.1|	unnamed protein product	*Kluyveromyces lactis*	5e-06
FE525514	-	No significant similarity	-	-
FE525515	-	No significant similarity	-	-
FE525516	gb|AAN63566.1|AF429823_1	septin 3	*Coccidioides immitis*	5e-08
FE525517	-	No significant similarity	-	-
FE525518	-	No significant similarity	-	-
FE525519	-	No significant similarity	-	-
FE525520	-	No significant similarity	-	-
FE525521	gb|AAO52807.1|	hypothetical protein	*Bacillus megaterium*	2e-04
FE525522	-	No significant similarity	-	-
FE525523	-	No significant similarity	-	-
FE525524	-	No significant similarity	-	-
FE525525	-	No significant similarity	-	-
FE525526	-	No significant similarity	-	-
FE525527	ref|XP_660794.1|	hypothetical protein AN3190.2	*Aspergillus nidulans FGSC A4*	7e-23
FE525528	-	No significant similarity	-	-
FE525529	-	No significant similarity	-	-
FE525530	-	No significant similarity	-	-
FE525531	-	No significant similarity	-	-
FE525532	-	No significant similarity	-	-
FE525533	-	No significant similarity	-	-
FE525534	-	No significant similarity	-	-
FE525535	-	No significant similarity	-	-
FE525536	ref|XP_001242406.1|	O-acetylhomoserine	*Coccidioides immitis RS*	5e-21
FE525537	-	No significant similarity	-	-
FE525538	-	No significant similarity	-	-
FE525539	-	No significant similarity	-	-
FE525540	-	No significant similarity	-	-
FE525541	-	No significant similarity	-	-
FE525542	-	No significant similarity	-	-
FE525543	ref|XP_001241788.1|	hypothetical protein CIMG_05684	*Coccidioides immitis RS*	5e-14
FE525544	ref|XP_001241788.1|	hypothetical protein CIMG_05684	*Coccidioides immitis RS*	5e-14

FE525545	-	No significant similarity	-	-
FE525546	-	No significant similarity	-	-
FE525547	ref|XP_001247210.1|	NAD-specific glutamate dehydrogenase (NAD-GDH)	*Coccidioides immitis RS*	3e-34
FE525548	gb|ABG74714.1|	Ac1147-like protein	*Diaphorina citri*	4e-21
FE525549	ref|XP_001241788.1|	hypothetical protein CIMG_05684	*Coccidioides immitis RS*	8e-14
FE525550	-	No significant similarity	-	-
FE525551	-	No significant similarity	-	-
FE525552	-	No significant similarity	-	-
FE525553	ref|XP_001266925.1|	alpha-aminoadipate reductase large subunit, putative	*Neosartorya fischeri NRRL 181*	4e-11
FE525554	-	No significant similarity	-	-
FE525555	ref|XP_001241783.1|	hypothetical protein CIMG_05679	*Coccidioides immitis RS*	3e-05
FE525556	emb|CAM19749.1|	novel KRAB box containing protein	*Mus musculus*	1e-08
FE525557	-	No significant similarity	-	-
FE525558	-	No significant similarity	-	-
FE525559	-	No significant similarity	-	-
FE525560	emb|CAJ83813.1|	CHK1 checkpoint homolog (S. pombe)	*Xenopus tropicalis*	3e-09
FE525561	-	No significant similarity	-	-
FE525562	-	No significant similarity	-	-
FE525563	-	No significant similarity	-	-
FE525564	-	No significant similarity	-	-
FE525565	-	No significant similarity	-	-
FE525566	-	No significant similarity	-	-
FE525567	-	No significant similarity	-	-
FE525568	ref|XP_001276134.1|	vacuolar aspartyl aminopeptidase Lap4, putative	*Aspergillus clavatus NRRL 1*	1e-10
FE525569	gb|AAB05810.1|	super cysteine rich protein; SCRP	*Homo sapiens*	9e-15
FE525570	ref|XP_001239110.1|	hypothetical protein CIMG_10132	*Coccidioides immitis RS*	1e-16
FE525571	-	No significant similarity	-	-
FE525572	ref|XP_001261472.1|	40S ribosomal protein S13	*Neosartorya fischeri NRRL 181*	2e-10
FE525573	ref|XP_001275347.1|	40S ribosomal protein S9	*Aspergillus clavatus NRRL 1*	1e-11
FE525574	-	No significant similarity	-	-
FE525575	-	No significant similarity	-	-
FE525576	-	No significant similarity	-	-
FE525577	-	No significant similarity	-	-
FE525578	-	No significant similarity	-	-

FE525579	dbj|BAE58435.1|	unnamed protein product	*Aspergillus oryzae*	2e-06
FE525580	ref|XP_001258253.1|	ABC transporter, putative	*Neosartorya fischeri NRRL 181*	1e-21
FE525581	ref|XP_001248139.1|	hypothetical protein CIMG_01910	*Coccidioides immitis RS*	2e-25
FE525582	-	No significant similarity	-	-
FE525583	ref|XP_001246822.1|	hypothetical protein CIMG_00593	*Coccidioides immitis RS*	3e-40
FE525584	-	No significant similarity	-	-
FE525585	gb|AAG01549.3|AF291822_1	multidrug resistance protein MDR	*Trichophyton rubrum*	3e-21
FE525586	ref|XP_001258253.1|	ABC transporter, putative	*Neosartorya fischeri NRRL 181*	3e-21
FE525587	ref|XP_001242594.1|	hypothetical protein CIMG_06490	*Coccidioides immitis RS*	2e-07
FE525588	ref|YP_840033.1|	Glyoxalase/bleomycin resistance protein/dioxygenase	*Burkholderia cenocepacia HI2424*	3e-11
FE525589	ref|XP_001258253.1|	ABC transporter, putative	*Neosartorya fischeri NRRL 181*	1e-21
FE525590	-	No significant similarity	-	-
FE525591	-	No significant similarity	-	-
FE525592	ref|YP_840033.1|	Glyoxalase/bleomycin resistance protein/dioxygenase	*Burkholderia cenocepacia HI2424*	2e-07
FE525593	gb|EAL84327.2|	short chain oxidoreductase (CsgA), putative	*Aspergillus fumigatus Af293*	2e-04
FE525594	ref|XP_001272698.1|	Dopey, N-terminal domain protein	*Aspergillus clavatus NRRL 1*	2e-09
FE525595	-	No significant similarity	-	-
FE525596	gb|AAG24792.1|AF264028_2	pol protein	*Glomerella cingulata*	2e-43
FE525597	ref|XP_001266096.1|	Pumilio-family RNA binding repeat protein	*Neosartorya fischeri NRRL 181*	3e-10
FE525598	-	No significant similarity	-	-
FE525599	ref|XP_001242594.1|	hypothetical protein CIMG_06490	*Coccidioides immitis RS*	5e-07
FE525600	gb|AAG01549.3|AF291822_1	multidrug resistance protein MDR	*Trichophyton rubrum*	3e-21
FE525601	-	No significant similarity	-	-
FE525602	ref|YP_840033.1|	Glyoxalase/bleomycin resistance protein/dioxygenase	*Burkholderia cenocepacia HI2424*	3e-11
FE525603	-	No significant similarity	-	-
FE525604	-	No significant similarity	-	-
FE525605	gb|EEQ28523.1|	Salicylate hydroxylase (Salicylate 1-monooxygenase)	*Microsporum canis* CBS 113480	6e-43
FE525606	ref|XP_001247286.1|	hypothetical protein CIMG_01057	*Coccidioides immitis RS*	1e-05

FE525607	ref|XP_001258253.1|	ABC transporter, putative	*Neosartorya fischeri NRRL 181*	1e-21
FE525608	gb|EAL84327.2|	short chain oxidoreductase (CsgA), putative	*Aspergillus fumigatus Af293*	7e-04
FE525609	-	No significant similarity	-	-
FE525610	ref|XP_001268233.1|	ABC transporter, putative	*Aspergillus clavatus NRRL 1*	2e-17
FE525611	ref|XP_662910.1|	hypothetical protein AN5306.2	*Aspergillus nidulans FGSC A4*	6e-11
FE525612	ref|XP_001229436.1|	hypothetical protein CHGG_02920	*Chaetomium globosum CBS 148.51*	3e-04
FE525613	ref|XP_001258253.1|	ABC transporter, putative	*Neosartorya fischeri NRRL 181*	2e-21
FE525614	-	No significant similarity	-	-
FE525615	ref|XP_001242594.1|	hypothetical protein CIMG_06490	*Coccidioides immitis RS*	6e-08
FE525616	-	No significant similarity	-	-
FE525617	-	No significant similarity	-	-
FE525618	ref|XP_750058.1|	carboxylic ester hydrolase (Ppe1), putative	*Aspergillus fumigatus Af293*	2e-24
FE525619	ref|YP_673529.1|	Glyoxalase/bleomycin resistance protein/dioxygenase	*Mesorhizobium sp. BNC1*	4e-09
FE525620	-	No significant similarity	-	-
FE525621	gb|ABF22673.1|	copper-sulfate regulated protein 1	*Ajellomyces capsulatus*	5e-15
FE525622	ref|XP_662910.1|	hypothetical protein AN5306.2	*Aspergillus nidulans FGSC A4*	2e-10
FE525623	ref|XP_001266096.1|	Pumilio-family RNA binding repeat protein	*Neosartorya fischeri NRRL 181*	4e-09
FE525624	ref|XP_001267191.1|	DNA mismatch repair protein Msh6, putative	*Neosartorya fischeri NRRL 181*	2e-33
FE525625	-	No significant similarity	-	-
FE525626	ref|XP_367322.1|	hypothetical protein MG07247.4	*Magnaporthe grisea 70-15*	2e-06
FE525627	gb|AAP23304.1|	NIMA interactive protein	*Emericella nidulans*	6e-25
FE525628	ref|XP_001262368.1|	DEAD helicases superfamily protein (Aquarius), putative	*Neosartorya fischeri NRRL 181*	1e-19
FE525629	-	No significant similarity	-	-
FE525630	-	No significant similarity	-	-
FE525631	-	No significant similarity	-	-

FE525632	ref|XP_001211183.1|	DNA polymerase gamma	*Aspergillus terreus NIH2624*	4e-22
FE525633	-	No significant similarity	-	-
FE525634	ref|XP_001242594.1|	hypothetical protein CIMG_06490	*Coccidioides immitis RS*	6e-08
FE525635	gb|AAP78735.1|	nonribosomal peptide synthase	*Alternaria brassicae*	8e-06
FE525636	ref|XP_001240454.1|	hypothetical protein CIMG_07617	*Coccidioides immitis RS*	3e-06
FE525637	ref|XP_001211183.1|	DNA polymerase gamma	*Aspergillus terreus NIH2624*	4e-22
FE525638	gb|AAG01549.3|AF291822_1	multidrug resistance protein MDR	*Trichophyton rubrum*	3e-21
FE525639	-	No significant similarity	-	-
FE525640	ref|XP_380291.1|	hypothetical protein FG00115.1	*Gibberella zeae PH-1*	5e-06
FE525641	gb|AAP78735.1|	nonribosomal peptide synthase	*Alternaria brassicae*	4e-04
FE525642	ref|XP_001272698.1|	Dopey, N-terminal domain protein	*Aspergillus clavatus NRRL 1*	4e-09
FE525643	gb|AAP78735.1|	nonribosomal peptide synthase	*Alternaria brassicae*	5e-06
FE525644	gb|AAG24792.1|AF264028_2	pol protein	*Glomerella cingulata*	1e-42
FE525645	ref|XP_001386445.1|	Salicylate hydroxylase (Salicylate 1-monooxygenase)	*Pichia stipitis CBS 6054*	1e-16
FE525646	-	No significant similarity	-	-
FE525647	-	No significant similarity	-	-
FE525648	-	No significant similarity	-	-
FE525649	-	No significant similarity	-	-
FE525650	-	No significant similarity	-	-
FE525651	ref|XP_001272698.1|	Dopey, N-terminal domain protein	*Aspergillus clavatus NRRL 1*	2e-08
FE525652	ref|XP_001242594.1|	hypothetical protein CIMG_06490	*Coccidioides immitis RS*	6e-08
FE525653	-	No significant similarity	-	-
FE525654	-	No significant similarity	-	-
FE525655	gb|ABF22673.1|	copper-sulfate regulated protein 1	*Ajellomyces capsulatus*	7e-18
FE525656	ref|XP_662910.1|	hypothetical protein AN5306.2	*Aspergillus nidulans FGSC A4*	3e-11
FE525657	ref|XP_001275274.1|	PB1 domain protein, putative	*Aspergillus clavatus NRRL 1*	8e-06
FE525658	ref|XP_662910.1|	hypothetical protein AN5306.2	*Aspergillus nidulans FGSC A4*	3e-11
FE525659	gb|AAG01549.3|AF291822_1	multidrug resistance protein MDR	*Trichophyton rubrum*	9e-21
FE525660	gb|AAP23304.1|	NIMA interactive protein	*Emericella nidulans*	6e-25
FE525661	ref|XP_001272698.1|	Dopey, N-terminal domain protein	*Aspergillus clavatus NRRL 1*	4e-09

FE525662	ref|XP_001272698.1|	Dopey, N-terminal domain protein	*Aspergillus clavatus NRRL 1*	4e-09
FE525663	-	No significant similarity	-	-
FE525664	gb|AAP78735.1|	nonribosomal peptide synthase	*Alternaria brassicae*	5e-06
FE525665	ref|XP_001258253.1|	ABC transporter, putative	*Neosartorya fischeri NRRL 181*	1e-21
FE525666	ref|XP_001226392.1|	hypothetical protein CHGG_08465	*Chaetomium globosum CBS 148.51*	3e-09
FE525667	ref|XP_001265341.1|	ribosomal protein/carboxylic ester hydrolase (Ppe1), putative	*Neosartorya fischeri NRRL 181*	2e-24
FE525668	ref|XP_001265341.1|	ribosomal protein/carboxylic ester hydrolase (Ppe1), putative	*Neosartorya fischeri NRRL 181*	2e-24
FE525669	ref|XP_001248139.1|	hypothetical protein CIMG_01910	*Coccidioides immitis RS*	7e-26
FE525670	ref|XP_001265341.1|	ribosomal protein/carboxylic ester hydrolase (Ppe1), putative	*Neosartorya fischeri NRRL 181*	2e-24
FE525671	ref|XP_001265341.1|	ribosomal protein/carboxylic ester hydrolase (Ppe1), putative	*Neosartorya fischeri NRRL 181*	2e-24
FE525672	gb|AAP78735.1|	nonribosomal peptide synthase	*Alternaria brassicae*	5e-06
FE525673	ref|YP_840033.1|	Glyoxalase/bleomycin resistance protein/dioxygenase	*Burkholderia cenocepacia HI2424*	5e-11
FE525674	ref|XP_001386445.1|	Salicylate hydroxylase (Salicylate 1-monooxygenase)	*Pichia stipitis CBS 6054*	2e-18
FE525675	ref|XP_001211183.1|	DNA polymerase gamma	*Aspergillus terreus NIH2624*	4e-22
FE525676	ref|XP_001211183.1|	DNA polymerase gamma	*Aspergillus terreus NIH2624*	4e-22
FE525677	-	No significant similarity	-	-
FE525678	-	No significant similarity	-	-
FE525679	ref|XP_662910.1|	hypothetical protein AN5306.2	*Aspergillus nidulans FGSC A4*	3e-11
FE525680	-	No significant similarity	-	-
FE525681	-	No significant similarity	-	-
FE525682	ref|XP_001215759.1|	woronin body major protein	*Aspergillus terreus NIH2624*	2e-26
FE525683	ref|XP_001272343.1|	indoleamine 2,3-dioxygenase family protein	*Aspergillus clavatus NRRL 1*	1e-27
FE525684	-	No significant similarity	-	-
FE525685	-	No significant similarity	-	-
FE525686	-	No significant similarity	-	-

FE525687	ref|XP_001269335.1|	AAA family ATPase/60S ribosome export protein Rix7, putative	*Aspergillus clavatus NRRL 1*	2e-12
FE525688	-	No significant similarity	-	-
FE525689	ref|XP_001268941.1|	methyltransferase small domain protein	*Aspergillus clavatus NRRL 1*	7e-23
FE525690	ref|XP_001262277.1|	alpha/beta fold family hydrolase, putative	*Neosartorya fischeri NRRL 181*	2e-06
FE525691	ref|XP_001272129.1|	LYR family protein	*Aspergillus clavatus NRRL 1*	2e-07
FE525692	ref|XP_001228475.1|	40s ribosomal protein S5	*Chaetomium globosum CBS 148.51*	2e-46
FE525693	ref|XP_001268941.1|	methyltransferase small domain protein	*Aspergillus clavatus NRRL 1*	2e-13
FE525694	-	No significant similarity	-	-
FE525695	-	No significant similarity	-	-
FE525696	ref|XP_001264172.1|	3-isopropylmalate dehydrogenase Leu2A	*Neosartorya fischeri NRRL 181*	6e-36
FE525697	-	No significant similarity	-	-
FE525698	dbj|BAB33421.1|	putative senescence-associated protein	*Pisum sativum*	3e-32
FE525699	gb|AAB05810.1|	super cysteine rich protein; SCRP	*Homo sapiens*	2e-14
FE525700	-	No significant similarity	-	-
FE525701	-	No significant similarity	-	-
FE525702	-	No significant similarity	-	-
FE525703	gb|AAL78196.1|AF126048_1	UDP-N-acetylglucosamine:dolichyl phosphate N-acetylglucosamine-1-phosphate transferase; GPT	*Aspergillus niger*	5e-63
FE525704	ref|XP_748912.1|	small nuclear ribonucleoprotein SmE, putative	*Aspergillus fumigatus Af293*	1e-05
FE525705	-	No significant similarity	-	-
FE525706	-	No significant similarity	-	-
FE525707	-	No significant similarity	-	-
FE525708	ref|XP_001244644.1|	hypothetical protein CIMG_04085	*Coccidioides immitis RS*	4e-04
FE525709	-	No significant similarity	-	-
FE525710	ref|XP_001213511.1|	type II proteins geranylgeranyltransferase beta subunit	*Aspergillus terreus NIH2624*	4e-35
FE525711	ref|XP_001247006.1|	hypothetical protein CIMG_00777	*Coccidioides immitis RS*	1e-08
FE525712	-	No significant similarity	-	-
FE525713	-	No significant similarity	-	-
FE525714	ref|XP_748019.1|	pyruvate dehydrogenase kinase, putative	*Aspergillus fumigatus*	5e-56

			Af293	
FE525715	-	No significant similarity	-	-
FE525716	dbj|BAB12047.1|	alpha-crystallin-related protein	*Arthroderma benhamiae*	3e-10
FE525717	dbj|BAE62191.1|	unnamed protein product	*Aspergillus oryzae*	2e-16
FE525718	-	No significant similarity	-	-
FE525719	ref|XP_001269249.1|	adenosylhomocysteinase	*Aspergillus clavatus NRRL 1*	1e-37
FE525720	-	No significant similarity	-	-
FE525721	dbj|BAB12047.1|	alpha-crystallin-related protein	*Arthroderma benhamiae*	8e-20
FE525722	ref|XP_001260869.1|	kinase activator (Atg17), putative	*Neosartorya fischeri NRRL 181*	3e-09
FE525723	ref|XP_001215759.1|	woronin body major protein	*Aspergillus terreus NIH2624*	5e-27
FE525724	-	No significant similarity	-	-
FE525725	ref|XP_001258025.1|	VHS domain protein	*Neosartorya fischeri NRRL 181*	1e-06
FE525726	-	No significant similarity	-	-
FE525727	-	No significant similarity	-	-
FE525728	-	No significant similarity	-	-
FE525729	ref|XP_001246822.1|	hypothetical protein CIMG_00593	*Coccidioides immitis RS*	7e-20
FE525730	ref|XP_001248599.1|	eukaryotic translation initiation factor 3 subunit 7 homolog	*Coccidioides immitis RS*	1e-24
FE525731	-	No significant similarity	-	-
FE525732	-	No significant similarity	-	-
FE525733	gb|EDK04135.1|	conserved hypothetical protein	*Magnaporthe grisea 70-15*	3e-04
FE525734	dbj|BAB12047.1|	alpha-crystallin-related protein	*Arthroderma benhamiae*	9e-18
FE525735	dbj|BAB12047.1|	alpha-crystallin-related protein	*Arthroderma benhamiae*	7e-20
FE525736	-	No significant similarity	-	-
FE525737	-	No significant similarity	-	-
FE525738	gb|ABB18373.1|	chitobiase	*Coccidioides posadasii*	4e-50
FE525739	-	No significant similarity	-	-
FE525740	ref|XP_001268837.1|	fibrillarin	*Aspergillus clavatus NRRL 1*	3e-36
FE525741	-	No significant similarity	-	-
FE525742	-	No significant similarity	-	-
FE525743	ref|XP_001276345.1|	histone H2A	*Aspergillus clavatus NRRL 1*	4e-19
FE525744	ref|XP_001261116.1|	cyanate hydratase, putative	*Neosartorya fischeri*	9e-21

			NRRL 181	
FE525745	ref|XP_750031.1|	mRNA cleavage and polyadenylation specificity factor complex subunit, putative	*Aspergillus fumigatus Af293*	1e-06
FE525746	gb|AAB05810.1|	super cysteine rich protein; SCRP	*Homo sapiens*	4e-14
FE525747	gb|AAB05810.1|	super cysteine rich protein; SCRP	*Homo sapiens*	2e-14
FE525748	ref|XP_001266066.1|	60S ribosomal protein L23	*Neosartorya fischeri NRRL 181*	2e-34
FE525749	-	No significant similarity	-	-
FE525750	ref|XP_001274417.1|	oxidoreductase	*Aspergillus clavatus NRRL 1*	4e-44
FE525751	-	No significant similarity	-	-
FE525752	ref|XP_001272850.1|	mitochondrial ATPase inhibitor, putative	*Aspergillus clavatus NRRL 1*	1e-05
FE525753	ref|XP_001274092.1|	cysteinyl-tRNA synthetase	*Aspergillus clavatus NRRL 1*	4e-51
FE525754	-	No significant similarity	-	-
FE525755	dbj|BAB12047.1|	alpha-crystallin-related protein	*Arthroderma benhamiae*	7e-20
FE525756	gb|EAL84586.2|	DNA repair protein (Tof1), putative	*Aspergillus fumigatus Af293*	1e-24
FE525757	ref|XP_001269377.1|	DUF814 domain protein	*Aspergillus clavatus NRRL 1*	2e-05
FE525758	-	No significant similarity	-	-
FE525759	dbj|BAB12047.1|	alpha-crystallin-related protein	*Arthroderma benhamiae*	1e-16
FE525760	-	No significant similarity	-	-
FE525761	ref|XP_752890.1|	conserved hypothetical protein	*Aspergillus fumigatus Af293*	4e-13
FE525762	ref|XP_750435.1|	protein phophatase 2C family protein	*Aspergillus fumigatus Af293*	3e-30
FE525763	ref|XP_001244432.1|	hypothetical protein CIMG_03873	*Coccidioides immitis RS*	8e-56
FE525764	ref|XP_001272790.1|	small nucleolar ribonucleoprotein complex subunit, putative	*Aspergillus clavatus NRRL 1*	4e-14
FE525765	-	No significant similarity	-	-
FE525766	ref|NP_983024.1|	ABR078Cp	*Ashbya gossypii ATCC 10895*	6e-09
FE525767	-	No significant similarity	-	-
FE525768	ref|XP_001266177.1|	mitochondrial large ribosomal subunit protein L1, putative	*Neosartorya fischeri NRRL 181*	3e-22
FE525769	emb|CAK42954.1|	unnamed protein product	*Aspergillus niger*	4e-24

FE525770	ref|XP_001266773.1|	DNA repair protein Rad50	*Neosartorya fischeri NRRL 181*	1e-19
FE525771	dbj|BAB12047.1|	alpha-crystallin-related protein	*Arthroderma benhamiae*	7e-20
FE525772	-	No significant similarity	-	-
FE525773	-	No significant similarity	-	-
FE525774	dbj|BAB12047.1|	alpha-crystallin-related protein	*Arthroderma benhamiae*	2e-19
FE525775	ref|XP_001263008.1|	heat shock protein Hsp30/Hsp42, putative	*Neosartorya fischeri NRRL 181*	1e-09
FE525776	-	No significant similarity	-	-
FE525777	-	No significant similarity	-	-
FE525778	-	No significant similarity	-	-
FE525779	ref|XP_748881.1|	NACHT domain protein, putative	*Aspergillus fumigatus Af293*	6e-04
FE525780	-	No significant similarity	-	-
FE525781	-	No significant similarity	-	-
FE525782	-	No significant similarity	-	-
FE525783	-	No significant similarity	-	-
FE525784	ref|XP_001246947.1|	glycolipid 2-alpha-mannosyltransferase	*Coccidioides immitis RS*	2e-06
FE525785	ref|XP_868845.1|	hypothetical protein AN9463.2	*Aspergillus nidulans FGSC A4*	8e-04
FE525786	ref|XP_001269539.1|	DUF887 domain protein	*Aspergillus clavatus NRRL 1*	3e-12
FE525787	-	No significant similarity	-	-
FE525788	-	No significant similarity	-	-
FE525789	ref|XP_001268953.1|	regulator of nonsense transcripts, putative	*Aspergillus clavatus NRRL 1*	5e-25
FE525790	-	No significant similarity	-	-
FE525791	ref|XP_001213531.1|	proteasome component PRE3 precursor	*Aspergillus terreus NIH2624*	2e-30
FE525792	ref|XP_001267735.1|	short chain dehydrogenase/reductase family protein	*Aspergillus clavatus NRRL 1*	6e-20
FE525793	ref|XP_001258400.1|	anthranilate synthase component I, putative	*Neosartorya fischeri NRRL 181*	1e-07
FE525794	ref|XP_001270328.1|	DUF803 domain protein	*Aspergillus clavatus NRRL 1*	6e-32
FE525795	-	No significant similarity	-	-
FE525796	gb|AAX33296.1|	heat shock protein 90	*Paracoccidioides brasiliensis*	7e-21

FE525797	ref|XP_001268331.1|	proteasome regulatory particle subunit (RpnI), putative	*Aspergillus clavatus NRRL 1*	1e-17
FE525798	-	No significant similarity	-	-
FE525799	dbj|BAE62495.1|	unnamed protein product	*Aspergillus oryzae*	6e-14
FE525800	-	No significant similarity	-	-
FE525801	ref|XP_001247141.1|	60S ribosomal protein L19	*Coccidioides immitis RS*	8e-06
FE525802	ref|XP_001274718.1|	amino acid permease, putative	*Aspergillus clavatus NRRL 1*	3e-12
FE525803	-	No significant similarity	-	-
FE525804	-	No significant similarity	-	-
FE525805	-	No significant similarity	-	-
FE525806	-	No significant similarity	-	-
FE525807	ref|XP_749497.1|	Ras GTPase activating protein, putative	*Aspergillus fumigatus Af293*	3e-04
FE525808	ref|XP_001260419.1|	60S ribosomal protein L10	*Neosartorya fischeri NRRL 181*	7e-39
FE525809	ref|XP_001265142.1|	DNA-directed DNA polymerase theta, putative	*Neosartorya fischeri NRRL 181*	1e-27
FE525810	ref|XP_001269539.1|	DUF887 domain protein	*Aspergillus clavatus NRRL 1*	3e-12
FE525811	-	No significant similarity	-	-
FE525812	ref|XP_682122.1|	eukaryotic peptide chain release factor subunit 1	*Aspergillus nidulans FGSC A4*	1e-70
FE525813	-	No significant similarity	-	-
FE525814	ref|XP_001248599.1|	eukaryotic translation initiation factor 3 subunit 7 homolog	*Coccidioides immitis RS*	2e-22
FE525815	ref|XP_001248599.1|	eukaryotic translation initiation factor 3 subunit 7 homolog	*Coccidioides immitis RS*	8e-22
FE525816	ref|XP_001212104.1|	serine/threonine-protein kinase ssp1	*Aspergillus terreus NIH2624*	1e-03
FE525817	ref|XP_754663.1|	DNA polymerase epsilon, catalytic subunit A/POL2, putative	*Aspergillus fumigatus Af293*	1e-20
FE525818	ref|XP_754663.1|	DNA polymerase epsilon, catalytic subunit A/POL2, putative	*Aspergillus fumigatus Af293*	8e-06
FE525819	-	No significant similarity	-	-
FE525820	dbj|BAE62495.1|	unnamed protein product	*Aspergillus oryzae*	1e-03
FE525821	ref|XP_754663.1|	DNA polymerase epsilon, catalytic subunit A/POL2, putative	*Aspergillus fumigatus Af293*	2e-19
FE525822	ref|XP_001267336.1|	outer mitochondrial membrane protein porin	*Neosartorya fischeri NRRL 181*	8e-41

FE525823	ref|XP_001258439.1|	60S ribosomal protein L7a	*Neosartorya fischeri NRRL 181*	1e-08
FE525824	-	No significant similarity	-	-
FE525825	ref|XP_001214623.1|	eukaryotic translation initiation factor 2 gamma subunit	*Aspergillus terreus NIH2624*	6e-41
FE525826	-	No significant similarity	-	-
FE525827	ref|ZP_00344940.1|	COG0458: Carbamoylphosphate synthase large subunit (split gene in MJ)	*Nostoc punctiforme PCC 73102*	8e-12
FE525828	ref|XP_001264383.1|	C2H2 transcription factor (Rpn4), putative	*Neosartorya fischeri NRRL 181*	3e-54
FE525829	-	No significant similarity	-	-
FE525830	-	No significant similarity	-	-
FE525831	ref|XP_001270884.1|	eukaryotic translation initiation factor 3 subunit 2i, putative	*Aspergillus clavatus NRRL 1*	2e-42
FE525832	ref|XP_001265914.1|	sugar transporter, putative	*Neosartorya fischeri NRRL 181*	4e-21
FE525833	-	No significant similarity	-	-
FE525834	-	No significant similarity	-	-
FE525835	-	No significant similarity	-	-
FE525836	-	No significant similarity	-	-
FE525837	ref|XP_001270884.1|	eukaryotic translation initiation factor 3 subunit 2i, putative	*Aspergillus clavatus NRRL 1*	5e-42
FE525838	ref|XP_001273963.1|	MFS transporter, putative	*Aspergillus clavatus NRRL 1*	4e-11
FE525839	gb|AAB06687.1|	complement fixation antigen gb|AAA96515.1| complement-fixation antige	*Coccidioides posadasii*	6e-11
FE525840	ref|XP_001258400.1|	anthranilate synthase component I, putative	*Neosartorya fischeri NRRL 181*	4e-05
FE525841	-	No significant similarity	-	-
FE525842	ref|XP_755151.1|	NEDD8-like protein (RubA), putative	*Aspergillus fumigatus Af293*	3e-12
FE525843	ref|XP_755086.1|	prolidase pepP, putative	*Aspergillus fumigatus Af293*	1e-40
FE525844	-	No significant similarity	-	-
FE525845	ref|XP_001271354.1|	glutamate-cysteine ligase Gcs1, putative	*Aspergillus clavatus NRRL 1*	8e-14
FE525846	ref|XP_001270884.1|	eukaryotic translation initiation factor 3 subunit 2i, putative	*Aspergillus clavatus NRRL 1*	2e-43
FE525847	ref|XP_808355.1|	formin	*Trypanosoma cruzi*	2e-04

			strain CL Brener	
FE525848	ref|XP_001272561.1|	heat shock Hsp30-like protein, putative	*Aspergillus clavatus NRRL 1*	4e-12
FE525849	ref|XP_001211399.1|	acetoacetyl-CoA reductase	*Aspergillus terreus NIH2624*	4e-30
FE525850	-	No significant similarity	-	-
FE525851	-	No significant similarity	-	-
FE525852	-	No significant similarity	-	-
FE525853	-	No significant similarity	-	-
FE525854	-	No significant similarity	-	-
FE525855	ref|XP_001260488.1|	actin cytoskeleton protein (VIP1), putative	*Neosartorya fischeri NRRL 181*	3e-10
FE525856	ref|XP_001258534.1|	acyltransferase, putative	*Neosartorya fischeri NRRL 181*	2e-14
FE525857	-	No significant similarity	-	-
FE525858	ref|XP_001259741.1|	ABC transporter, putative	*Neosartorya fischeri NRRL 181*	1e-22
FE525859	-	No significant similarity	-	-
FE525860	-	No significant similarity	-	-
FE525861	-	No significant similarity	-	-
FE525862	-	No significant similarity	-	-
FE525863	-	No significant similarity	-	-
FE525864	-	No significant similarity	-	-
FE525865	-	No significant similarity	-	-
FE525866	-	No significant similarity	-	-
FE525867	-	No significant similarity	-	-
FE525868	-	No significant similarity	-	-
FE525869	ref|XP_754941.1|	aminoalcoholphosphotransferase	*Aspergillus fumigatus Af293*	2e-14
FE525870	ref|XP_001257402.1|	C2HC5 finger protein	*Neosartorya fischeri NRRL 181*	7e-14
FE525871	-	No significant similarity	-	-
FE525872	ref|XP_001257402.1|	C2HC5 finger protein	*Neosartorya fischeri NRRL 181*	5e-13
FE525873	ref|ZP_00344940.1|	COG0458: Carbamoylphosphate synthase large subunit (split gene in MJ)	*Nostoc punctiforme PCC 73102*	8e-12
FE525874	ref|XP_001248278.1|	hypothetical protein CIMG_02049	*Coccidioides immitis RS*	1e-10
FE525875	ref|XP_001209708.1|	conserved hypothetical protein	*Aspergillus terreus*	2e-18

			NIH2624	
FE525876	ref|XP_001262831.1|	conserved hypothetical protein	*Neosartorya fischeri NRRL 181*	8e-06
FE525877	-	No significant similarity	-	-
FE525878	ref|XP_001269644.1|	N-acetyltransferase (Nat5), putative	*Aspergillus clavatus NRRL 1*	3e-21
FE525879	ref|XP_754941.1|	aminoalcoholphosphotransferase	*Aspergillus fumigatus Af293*	2e-14
FE525880	dbj|BAA81686.1|	expressed in cucumber hypocotyls	*Cucumis sativus*	1e-04
FE525881	-	No significant similarity	-	-
FE525882	-	No significant similarity	-	-
FE525883	sp|P00048|CYC_NEUCR	Cytochrome c emb|CAA29050.1| cytochrome c	*Neurospora crassa*	5e-11
FE525884	-	No significant similarity	-	-
FE525885	ref|XP_001246545.1|	phosphatidylethanolamine N-methyltransferase	*Coccidioides immitis RS*	1e-04
FE525886	ref|XP_001275966.1|	MFS multidrug transporter, putative	*Aspergillus clavatus NRRL 1*	3e-31
FE525887	-	No significant similarity	-	-
FE525888	-	No significant similarity	-	-
FE525889	ref|XP_001213531.1|	proteasome component PRE3 precursor	*Aspergillus terreus NIH2624*	6e-31
FE525890	-	No significant similarity	-	-
FE525891	-	No significant similarity	-	-
FE525892	-	No significant similarity	-	-
FE525893	ref|XP_001247301.1|	proteasome component	*Coccidioides immitis RS*	7e-24
FE525894	-	No significant similarity	-	-
FE525895	ref|XP_721980.1|	putative S-adenosyl-L-homocysteine hydrolase	*Candida albicans SC5314*	8e-08
FE525896	gb|ABK60177.1|	putative reverse transcriptase	*Zingiber officinale*	1e-03
FE525897	ref|XP_721980.1|	putative S-adenosyl-L-homocysteine hydrolase	*Candida albicans SC5314*	2e-10
FE525898	ref|XP_001260488.1|	actin cytoskeleton protein (VIP1), putative	*Neosartorya fischeri NRRL 181*	1e-08
FE525899	-	No significant similarity	-	-
FE525900	ref|XP_001266911.1|	acetyl-coenzyme A synthetase FacA	*Neosartorya fischeri NRRL 181*	9e-26
FE525901	-	No significant similarity	-	-
FE525902	-	No significant similarity	-	-
FE525903	ref|XP_001257912.1|	PH domain protein	*Neosartorya fischeri*	2e-10

			NRRL 181	
FE525904	ref|XP_001268018.1|	PH domain protein	*Aspergillus clavatus NRRL 1*	5e-11
FE525905	ref|XP_001258439.1|	60S ribosomal protein L7a	*Neosartorya fischeri NRRL 181*	1e-08
FE525906	-	No significant similarity	-	-
FE525907	ref|ZP_00344940.1|	COG0458: Carbamoylphosphate synthase large subunit (split gene in MJ)	*Nostoc punctiforme PCC 73102*	7e-15
FE525908	ref|XP_001264383.1|	C2H2 transcription factor (Rpn4), putative	*Neosartorya fischeri NRRL 181*	1e-35
FE525909	gb|ABG56823.1|	hypothetical protein	*Klebsiella pneumoniae*	1e-05
FE525910	ref|XP_001270884.1|	eukaryotic translation initiation factor 3 subunit 2i, putative	*Aspergillus clavatus NRRL 1*	2e-42
FE525911	-	No significant similarity	-	-
FE525912	-	No significant similarity	-	-
FE525913	-	No significant similarity	-	-
FE525914	ref|XP_001263576.1|	eukaryotic translation initiation factor 3 subunit 2i, putative	*Neosartorya fischeri NRRL 181*	5e-27
FE525915	-	No significant similarity	-	-
FE525916	ref|XP_755151.1|	NEDD8-like protein (RubA), putative	*Aspergillus fumigatus Af293*	1e-11
FE525917	-	No significant similarity	-	-
FE525918	ref|XP_001263576.1|	eukaryotic translation initiation factor 3 subunit 2i, putative	*Neosartorya fischeri NRRL 181*	1e-27
FE525919	-	No significant similarity	-	-
FE525920	gb|ABF82266.1|	heat shock protein 30	*Penicillium marneffei*	2e-11
FE525921	ref|XP_001264679.1|	conserved hypothetical protein	*Neosartorya fischeri NRRL 181*	4e-31
FE525922	-	No significant similarity	-	-
FE525923	ref|XP_001214623.1|	eukaryotic translation initiation factor 2 gamma subunit	*Aspergillus terreus NIH2624*	2e-25
FE525924	-	No significant similarity	-	-
FE525925	ref|XP_001247301.1|	proteasome component	*Coccidioides immitis RS*	2e-26
FE525926	-	No significant similarity	-	-
FE525927	ref|XP_721980.1|	putative S-adenosyl-L-homocysteine hydrolase	*Candida albicans SC5314*	2e-13
FE525928	-	No significant similarity	-	-
FE525929	-	No significant similarity	-	-

FE525930	gb|EAL86837.2|	sodium/phosphate symporter, putative	*Aspergillus fumigatus Af293*	2e-11
FE525931	-	No significant similarity	-	-
FE525932	ref|XP_001266911.1|	acetyl-coenzyme A synthetase FacA	*Neosartorya fischeri NRRL 181*	5e-29
FE525933	-	No significant similarity	-	-
FE525934	-	No significant similarity	-	-
FE525935	ref|XP_001257912.1|	PH domain protein	*Neosartorya fischeri NRRL 181*	9e-13
FE525936	-	No significant similarity	-	-
FE525937	-	No significant similarity	-	-
FE525938	-	No significant similarity	-	-
FE525939	ref|XP_001267735.1|	short chain dehydrogenase/reductase family protein	*Aspergillus clavatus NRRL 1*	4e-21
FE525940	ref|XP_001213531.1|	proteasome component PRE3 precursor	*Aspergillus terreus NIH2624*	1e-30
FE525941	ref|XP_001260315.1|	short chain dehydrogenase/reductase family protein	*Neosartorya fischeri NRRL 181*	1e-19
FE525942	ref|XP_001258400.1|	anthranilate synthase component I, putative	*Neosartorya fischeri NRRL 181*	1e-07
FE525943	ref|XP_001270328.1|	DUF803 domain protein	*Aspergillus clavatus NRRL 1*	2e-29
FE525944	-	No significant similarity	-	-
FE525945	gb|AAX33296.1|	heat shock protein 90	*Paracoccidioides brasiliensis*	7e-21
FE525946	-	No significant similarity	-	-
FE525947	-	No significant similarity	-	-
FE525948	dbj|BAE62495.1|	unnamed protein product	*Aspergillus oryzae*	1e-14
FE525949	-	No significant similarity	-	-
FE525950	ref|XP_001247141.1|	60S ribosomal protein L19	*Coccidioides immitis RS*	8e-06
FE525951	-	No significant similarity	-	-
FE525952	-	No significant similarity	-	-
FE525953	-	No significant similarity	-	-
FE525954	ref|XP_001275476.1|	pyruvate dehydrogenase kinase	*Aspergillus clavatus NRRL 1*	2e-11
FE525955	-	No significant similarity	-	-
FE525956	-	No significant similarity	-	-
FE525957	-	No significant similarity	-	-

FE525958	-	No significant similarity	-	-
FE525959	ref|XP_001265142.1|	DNA-directed DNA polymerase theta, putative	*Neosartorya fischeri NRRL 181*	1e-27
FE525960	ref|XP_001247141.1|	60S ribosomal protein L19	*Coccidioides immitis RS*	6e-11
FE525961	-	No significant similarity	-	-
FE525962	ref|XP_682122.1|	eukaryotic peptide chain release factor subunit 1	*Aspergillus nidulans FGSC A4*	3e-52
FE525963	-	No significant similarity	-	-
FE525964	ref|XP_001248599.1|	eukaryotic translation initiation factor 3 subunit 7 homolog	*Coccidioides immitis RS*	5e-21
FE525965	-	No significant similarity	-	-
FE525966	-	No significant similarity	-	-
FE525967	ref|XP_746577.1|	peroxisomal multifunctional beta-oxidation protein (MFP), putative	*Aspergillus fumigatus Af293*	2e-19
FE525968	ref|XP_001211399.1|	acetoacetyl-CoA reductase	*Aspergillus terreus NIH2624*	4e-30
FE525969	ref|XP_001268779.1|	BAP31 domain protein, putative	*Aspergillus clavatus NRRL 1*	4e-14
FE525970	-	No significant similarity	-	-
FE525971	-	No significant similarity	-	-
FE525972	ref|XP_001275966.1|	MFS multidrug transporter, putative	*Aspergillus clavatus NRRL 1*	3e-31
FE525973	-	No significant similarity	-	-
FE525974	-	No significant similarity	-	-
FE525975	-	No significant similarity	-	-
FE525976	-	No significant similarity	-	-
FE525977	-	No significant similarity	-	-
FE525978	-	No significant similarity	-	-
FE525979	-	No significant similarity	-	-
FE525980	-	No significant similarity	-	-
FE525981	-	No significant similarity	-	-
FE525982	-	No significant similarity	-	-
FE525983	ref|XP_754941.1|	aminoalcoholphosphotransferase	*Aspergillus fumigatus Af293*	2e-14
FE525984	ref|XP_001211399.1|	acetoacetyl-CoA reductase	*Aspergillus terreus NIH2624*	2e-29
FE525985	-	No significant similarity	-	-
FE525986	-	No significant similarity	-	-
FE525987	ref|XP_001209708.1|	conserved hypothetical protein	*Aspergillus terreus*	2e-18

			NIH2624	
FE525988	ref|ZP_00344940.1|	COG0458: Carbamoylphosphate synthase large subunit (split gene in MJ)	*Nostoc punctiforme PCC 73102*	8e-12
FE525989	-	No significant similarity	-	-
FE525990	-	No significant similarity	-	-
FE525991	-	No significant similarity	-	-
FE525992	ref|XP_754941.1|	aminoalcoholphosphotransferase	*Aspergillus fumigatus Af293*	2e-14
FE525993	-	No significant similarity	-	-
FE525994	ref|XP_001246545.1|	phosphatidylethanolamine N-methyltransferase	*Coccidioides immitis RS*	1e-04
FE525995	-	No significant similarity	-	-
FE525996	-	No significant similarity	-	-
FE525997	sp|P00048|CYC_NEUCR	Cytochrome c emb|CAA29050.1| cytochrome c	*Neurospora crassa*	5e-11
FE525998	ref|XP_001209915.1|	conserved hypothetical protein	*Aspergillus terreus NIH2624*	1e-07
FE525999	-	No significant similarity	-	-
FE526000	ref|XP_001269644.1|	N-acetyltransferase (Nat5), putative	*Aspergillus clavatus NRRL 1*	3e-21
FE526001	-	No significant similarity	-	-
FE526002	-	No significant similarity	-	-
FE526003	-	No significant similarity	-	-
FE526004	gb|ABG67901.1|	phospholipase B	*Trichophyton rubrum*	4e-19
FE526005	ref|XP_751277.1|	acyltransferase, putative	*Aspergillus fumigatus Af293*	4e-09
FE526006	-	No significant similarity	-	-
FE526007	ref|XP_001258439.1|	60S ribosomal protein L7a	*Neosartorya fischeri NRRL 181*	1e-08
FE526008	-	No significant similarity	-	-
FE526009	gb|EAL90029.2|	translation initiation factor EF-2 gamma subunit, putative	*Aspergillus fumigatus Af293*	4e-21
FE526010	-	No significant similarity	-	-
FE526011	-	No significant similarity	-	-
FE526012	ref|ZP_00344940.1|	COG0458: Carbamoylphosphate synthase large subunit (split gene in MJ)	*Nostoc punctiforme PCC 73102*	2e-11
FE526013	ref|XP_001264383.1|	C2H2 transcription factor (Rpn4), putative	*Neosartorya fischeri NRRL 181*	8e-51
FE526014	-	No significant similarity	-	-
FE526015	-	No significant similarity	-	-

FE526016	ref|XP_001270884.1|	eukaryotic translation initiation factor 3 subunit 2i, putative	*Aspergillus clavatus NRRL 1*	2e-42
FE526017	-	No significant similarity	-	-
FE526018	-	No significant similarity	-	-
FE526019	-	No significant similarity	-	-
FE526020	-	No significant similarity	-	-
FE526021	ref|XP_001270884.1|	eukaryotic translation initiation factor 3 subunit 2i, putative	*Aspergillus clavatus NRRL 1*	5e-42
FE526022	-	No significant similarity	-	-
FE526023	gb|AAB06687.1|	complement fixation antigen gb|AAA96515.1| complement-fixation antige	*Coccidioides posadasii*	6e-11
FE526024	ref|XP_755151.1|	NEDD8-like protein (RubA), putative	*Aspergillus fumigatus Af293*	1e-08
FE526025	-	No significant similarity	-	-
FE526026	ref|XP_001263576.1|	eukaryotic translation initiation factor 3 subunit 2i, putative	*Neosartorya fischeri NRRL 181*	3e-28
FE526027	-	No significant similarity	-	-
FE526028	-	No significant similarity	-	-
FE526029	emb|CAM14118.1|	novel protein containing SEA domains	*Danio rerio*	1e-04
FE526030	-	No significant similarity	-	-
FE526031	-	No significant similarity	-	-
FE526032	-	No significant similarity	-	-
FE526033	ref|XP_001271726.1|	tyrosyl-tRNA synthetase, mitochondrial precursor (tyrosine--tRNA ligase) (tyrrs)	*Aspergillus clavatus NRRL 1*	1e-27
FE526034	-	No significant similarity	-	-
FE526035	gb|AAO52807.1|	hypothetical protein	*Bacillus megaterium*	2e-04
FE526036	-	No significant similarity	-	-
FE526037	ref|XP_001270287.1|	60S ribosomal protein L24	*Aspergillus clavatus NRRL 1*	3e-34
FE526038	-	No significant similarity	-	-
FE526039	-	No significant similarity	-	-
FE526040	-	No significant similarity	-	-
FE526041	-	No significant similarity	-	-
FE526042	-	No significant similarity	-	-
FE526043	-	No significant similarity	-	-
FE526044	ref|XP_001264535.1|	MFS peptide transporter, putaitve	*Neosartorya fischeri NRRL 181*	3e-21
FE526045	-	No significant similarity	-	-
FE526046	-	No significant similarity	-	-

FE526047	-	No significant similarity	-	-
FE526048	ref|XP_001272561.1|	heat shock Hsp30-like protein, putative	*Aspergillus clavatus NRRL 1*	4e-06
FE526049	-	No significant similarity	-	-
FE526050	-	No significant similarity	-	-
FE526051	ref|XP_001260474.1|	oxidoreductase, 2-nitropropane dioxygenase family, putative	*Neosartorya fischeri NRRL 181*	2e-49
FE526052	ref|XP_751914.1|	cytochrome c peroxidase, putative	*Aspergillus fumigatus Af293*	3e-04
FE526053	-	No significant similarity	-	-
FE526054	sp|P59769|GRP78_ASPAW	78 kDa glucose-regulated protein homolog precursor (GRP 78) (Immunoglobulin heavy chain-binding protein homolog) (BiP) sp|P83616|GRP78_ASPNG 78 kDa glucose-regulated protein homolog precursor (GRP 78) (Immunoglobulin heavy chain-binding protein homolog) (	*Aspergillus kawachii*	1e-52
FE526055	ref|XP_001247978.1|	30 kDa heat shock protein	*Coccidioides immitis RS*	7e-04
FE526056	-	No significant similarity	-	-
FE526057	-	No significant similarity	-	-
FE526058	-	No significant similarity	-	-
FE526059	-	No significant similarity	-	-
FE526060	ref|XP_001257997.1|	NADH-ubiquinone oxidoreductase 304 kDa subunit precursor	*Neosartorya fischeri NRRL 181*	5e-67
FE526061	ref|XP_001271404.1|	heat shock protein Hsp30/Hsp42, putative	*Aspergillus clavatus NRRL 1*	2e-08
FE526062	-	No significant similarity	-	-
FE526063	-	No significant similarity	-	-
FE526064	-	No significant similarity	-	-
FE526065	-	No significant similarity	-	-
FE526066	-	No significant similarity	-	-
FE526067	-	No significant similarity	-	-
FE526068	-	No significant similarity	-	-
FE526069	-	No significant similarity	-	-
FE526070	-	No significant similarity	-	-
FE526071	-	No significant similarity	-	-
FE526072	gb|ABB96277.1|	hesp-767	*Melampsora lini*	7e-05
FE526073	-	No significant similarity	-	-
FE526074	-	No significant similarity	-	-
FE526075	-	No significant similarity	-	-

FE526076	ref|XP_001213531.1|	proteasome component PRE3 precursor	*Aspergillus terreus NIH2624*	1e-29
FE526077	ref|XP_001220639.1|	proteasome subunit beta type 7 precursor	*Chaetomium globosum CBS 148.51*	2e-25
FE526078	-	No significant similarity	-	-
FE526079	gb|EAL86837.2|	sodium/phosphate symporter, putative	*Aspergillus fumigatus Af293*	6e-12
FE526080	ref|XP_001266911.1|	acetyl-coenzyme A synthetase FacA	*Neosartorya fischeri NRRL 181*	5e-29
FE526081	-	No significant similarity	-	-
FE526082	-	No significant similarity	-	-
FE526083	-	No significant similarity	-	-
FE526084	ref|XP_001257912.1|	PH domain protein	*Neosartorya fischeri NRRL 181*	9e-13
FE526085	ref|XP_001212691.1|	30 kDa heat shock protein	*Aspergillus terreus NIH2624*	1e-03
FE526086	-	No significant similarity	-	-
FE526087	-	No significant similarity	-	-
FE526088	ref|XP_001272465.1|	MFS multidrug transporter, putative	*Aspergillus clavatus NRRL 1*	5e-23
FE526089	-	No significant similarity	-	-
FE526090	-	No significant similarity	-	-
FE526091	ref|XP_001240305.1|	hypothetical protein CIMG_07468	*Coccidioides immitis RS*	2e-32
FE526092	-	No significant similarity	-	-
FE526093	ref|XP_001239696.1|	predicted protein	*Coccidioides immitis RS*	1e-07
FE526094	-	No significant similarity	-	-
FE526095	-	No significant similarity	-	-
FE526096	-	No significant similarity	-	-
FE526097	gb|ABM92787.1|	phytase	*Aspergillus oryzae*	4e-08
FE526098	-	No significant similarity	-	-
FE526099	-	No significant similarity	-	-
FE526100	-	No significant similarity	-	-
FE526101	-	No significant similarity	-	-
FE526102	-	No significant similarity	-	-
FE526103	-	No significant similarity	-	-
FE526104	-	No significant similarity	-	-
FE526105	-	No significant similarity	-	-
FE526106	gb|EDJ95143.1|	60S ribosomal protein L7	*Magnaporthe grisea 70-*	5e-11

			15	
FE526107	-	No significant similarity	-	-
FE526108	-	No significant similarity	-	-
FE526109	ref|XP_001248014.1|	predicted protein	*Coccidioides immitis RS*	7e-04
FE526110	-	No significant similarity	-	-
FE526111	-	No significant similarity	-	-
FE526112	-	No significant similarity	-	-
FE526113	-	No significant similarity	-	-
FE526114	ref|XP_001271793.1|	DEAD/DEAH box helicase, putative	*Aspergillus clavatus NRRL 1*	1e-48
FE526115	-	No significant similarity	-	-
FE526116	-	No significant similarity	-	-
FE526117	ref|XP_001271793.1|	DEAD/DEAH box helicase, putative	*Aspergillus clavatus NRRL 1*	1e-49
FE526118	-	No significant similarity	-	-
FE526119	-	No significant similarity	-	-
FE526120	-	No significant similarity	-	-
FE526121	-	No significant similarity	-	-
FE526122	-	No significant similarity	-	-
FE526123	ref|XP_001239221.1|	GTP-binding nuclear protein GSP1/Ran	*Coccidioides immitis RS*	4e-25
FE526124	-	No significant similarity	-	-
FE526125	-	No significant similarity	-	-
FE526126	-	No significant similarity	-	-
FE526127	-	No significant similarity	-	-
FE526128	ref|XP_001276376.1|	bZIP transcription factor JlbA/IDI-4	*Aspergillus clavatus NRRL 1*	8e-05
FE526129	-	No significant similarity	-	-
FE526130	-	No significant similarity	-	-
FE526131	-	No significant similarity	-	-
FE526132	-	No significant similarity	-	-
FE526133	-	No significant similarity	-	-
FE526134	-	No significant similarity	-	-
FE526135	-	No significant similarity	-	-
FE526136	-	No significant similarity	-	-
FE526137	-	No significant similarity	-	-
FE526138	-	No significant similarity	-	-
FE526139	-	No significant similarity	-	-
FE526140	ref|XP_755151.1|	NEDD8-like protein (RubA), putative	*Aspergillus fumigatus*	7e-12

			Af293	
FE526141	-	No significant similarity	-	-
FE526142	-	No significant similarity	-	-
FE526143	-	No significant similarity	-	-
FE526144	-	No significant similarity	-	-
FE526145	-	No significant similarity	-	-
FE526146	-	No significant similarity	-	-
FE526147	-	No significant similarity	-	-
FE526148	-	No significant similarity	-	-
FE526149	gb|ABF22673.1|	copper-sulfate regulated protein 1	*Ajellomyces capsulatus*	7e-18
FE526150	ref|XP_001266920.1|	kynurenine aminotransferase, putative	*Neosartorya fischeri NRRL 181*	2e-62
FE526151	gb|AAP23304.1|	NIMA interactive protein	*Emericella nidulans*	3e-24
FE526152	ref|XP_001240454.1|	hypothetical protein CIMG_07617	*Coccidioides immitis RS*	3e-06
FE526153	gb|AAP23304.1|	NIMA interactive protein	*Emericella nidulans*	1e-25
FE526154	gb|AAP23304.1|	NIMA interactive protein	*Emericella nidulans*	6e-25
FE526155	ref|XP_001269482.1|	Leucine Rich Repeat domain protein	*Aspergillus clavatus NRRL 1*	3e-10
FE526156	gb|ABF22673.1|	copper-sulfate regulated protein 1	*Ajellomyces capsulatus*	7e-18
FE526157	gb|AAP23304.1|	NIMA interactive protein	*Emericella nidulans*	1e-26
FE526158	ref|XP_001266920.1|	kynurenine aminotransferase, putative	*Neosartorya fischeri NRRL 181*	3e-63
FE526159	gb|AAP23304.1|	NIMA interactive protein	*Emericella nidulans*	1e-26
FE526160	gb|AAP23304.1|	NIMA interactive protein	*Emericella nidulans*	5e-26
FE526161	gb|AAG01549.3|AF291822_1	multidrug resistance protein MDR	*Trichophyton rubrum*	1e-20
FE526162	gb|ABF22673.1|	copper-sulfate regulated protein 1	*Ajellomyces capsulatus*	7e-18
FE526163	-	No significant similarity	-	-
FE526164	gb|AAP23304.1|	NIMA interactive protein	*Emericella nidulans*	5e-26
FE526165	gb|AAP23304.1|	NIMA interactive protein	*Emericella nidulans*	1e-26
FE526166	gb|AAP23304.1|	NIMA interactive protein	*Emericella nidulans*	6e-25
FE526167	ref|XP_001269482.1|	Leucine Rich Repeat domain protein	*Aspergillus clavatus NRRL 1*	3e-10
FE526168	gb|AAG24792.1|AF264028_2	pol protein	*Glomerella cingulata*	3e-44
FE526169	gb|AAP23304.1|	NIMA interactive protein	*Emericella nidulans*	4e-26
FE526170	gb|AAP23304.1|	NIMA interactive protein	*Emericella nidulans*	3e-27
FE526171	gb|AAP23304.1|	NIMA interactive protein	*Emericella nidulans*	6e-25
FE526172	gb|ABF22673.1|	copper-sulfate regulated protein 1	*Ajellomyces capsulatus*	7e-18
FE526173	gb|AAG01549.3|AF291822_1	multidrug resistance protein MDR	*Trichophyton rubrum*	3e-21

FE526174	ref|XP_001269482.1|	Leucine Rich Repeat domain protein	*Aspergillus clavatus NRRL 1*	3e-10
FE526175	gb|AAP23304.1|	NIMA interactive protein	*Emericella nidulans*	6e-25
FE526176	gb|AAG24792.1|AF264028_2	pol protein	*Glomerella cingulata*	3e-44
FE526177	ref|XP_001240454.1|	hypothetical protein CIMG_07617	*Coccidioides immitis RS*	3e-06
FE526178	ref|XP_001265341.1|	ribosomal protein/carboxylic ester hydrolase (Ppe1), putative	*Neosartorya fischeri NRRL 181*	2e-24
FE526179	gb|AAP23304.1|	NIMA interactive protein	*Emericella nidulans*	1e-26
FE526180	-	No significant similarity	-	-
FE526181	ref|XP_001240454.1|	hypothetical protein CIMG_07617	*Coccidioides immitis RS*	3e-06
FE526182	ref|XP_001240454.1|	hypothetical protein CIMG_07617	*Coccidioides immitis RS*	3e-06
FE526183	gb|AAG24792.1|AF264028_2	pol protein	*Glomerella cingulata*	3e-44
FE526184	gb|ABF22673.1|	copper-sulfate regulated protein 1	*Ajellomyces capsulatus*	7e-18
FE526185	gb|AAP23304.1|	NIMA interactive protein	*Emericella nidulans*	6e-25
FE526186	gb|ABF22673.1|	copper-sulfate regulated protein 1	*Ajellomyces capsulatus*	7e-18
FE526187	gb|AAG24792.1|AF264028_2	pol protein	*Glomerella cingulata*	3e-44
FE526188	gb|AAG01549.3|AF291822_1	multidrug resistance protein MDR	*Trichophyton rubrum*	1e-20
FE526189	gb|AAP23304.1|	NIMA interactive protein	*Emericella nidulans*	6e-25
FE526190	-	No significant similarity	-	-
FE526191	gb|AAP23304.1|	NIMA interactive protein	*Emericella nidulans*	3e-13
FE526192	gb|AAG24792.1|AF264028_2	pol protein	*Glomerella cingulata*	3e-44
FE526193	ref|XP_001240454.1|	hypothetical protein CIMG_07617	*Coccidioides immitis RS*	3e-06
FE526194	gb|AAG24792.1|AF264028_2	pol protein	*Glomerella cingulata*	3e-44
FE526195	ref|XP_001269482.1|	Leucine Rich Repeat domain protein	*Aspergillus clavatus NRRL 1*	8e-04
FE526196	-	No significant similarity	-	-
FE526197	gb|AAP23304.1|	NIMA interactive protein	*Emericella nidulans*	6e-25
FE526198	ref|XP_001266920.1|	kynurenine aminotransferase, putative	*Neosartorya fischeri NRRL 181*	4e-62
FE526199	gb|AAP23304.1|	NIMA interactive protein	*Emericella nidulans*	3e-26
FE526200	-	No significant similarity	-	-
FE526201	gb|AAG24792.1|AF264028_2	pol protein	*Glomerella cingulata*	3e-44
FE526202	-	No significant similarity	-	-
FE526203	gb|AAP23304.1|	NIMA interactive protein	*Emericella nidulans*	2e-24
FE526204	gb|AAP23304.1|	NIMA interactive protein	*Emericella nidulans*	5e-26
FE526205	-	No significant similarity	-	-
FE526206	gb|AAG24792.1|AF264028_2	pol protein	*Glomerella cingulata*	1e-42
FE526207	gb|AAG24792.1|AF264028_2	pol protein	*Glomerella cingulata*	6e-43

FE526208	ref|XP_001240454.1|	hypothetical protein CIMG_07617	*Coccidioides immitis RS*	3e-06
FE526209	ref|XP_751710.1|	kynurenine aminotransferase, putative	*Aspergillus fumigatus Af293*	2e-60
FE526210	ref|XP_001265341.1|	ribosomal protein/carboxylic ester hydrolase (Ppe1), putative	*Neosartorya fischeri NRRL 181*	3e-25
FE526211	gb|AAG01549.3|AF291822_1	multidrug resistance protein MDR	*Trichophyton rubrum*	1e-20
FE526212	gb|AAG01549.3|AF291822_1	multidrug resistance protein MDR	*Trichophyton rubrum*	4e-13
FE526213	sp|Q2URJ0|PPME1_ASPOR	Protein phosphatase methylesterase 1 (PMe-1)	*Aspergillus oryzae*	2e-25
FE526214	ref|XP_001242594.1|	hypothetical protein CIMG_06490	*Coccidioides immitis RS*	1e-06
FE526215	-	No significant similarity	-	-
FE526216	-	No significant similarity	-	-
FE526217	-	No significant similarity	-	-
FE526218	gb|AAG24792.1|AF264028_2	pol protein	*Glomerella cingulata*	3e-44
FE526219	gb|AAG24792.1|AF264028_2	pol protein	*Glomerella cingulata*	3e-44
FE526220	gb|AAP23304.1|	NIMA interactive protein	*Emericella nidulans*	5e-22
FE526221	gb|AAG24792.1|AF264028_2	pol protein	*Glomerella cingulata*	3e-44
FE526222	gb|ABF22673.1|	copper-sulfate regulated protein 1	*Ajellomyces capsulatus*	2e-17
FE526223	dbj|BAC01275.1|	cytochrome P450nor	*Aspergillus oryzae*	2e-16
FE526224	gb|EEQ27465.1|	copper resistance-associated P-type ATPase, putative	*Microsporum canis CBS 113480*	8e-24
FE526225	ref|XP_001269482.1|	Leucine Rich Repeat domain protein	*Aspergillus clavatus NRRL 1*	3e-10
FE526226	gb|AAP23304.1|	NIMA interactive protein	*Emericella nidulans*	3e-22
FE526227	ref|XP_001262830.1|	sexual development protein EsdC, putative	*Neosartorya fischeri NRRL 181*	2e-11
FE526228	gb|AAP23304.1|	NIMA interactive protein	*Emericella nidulans*	5e-22
FE526229	ref|XP_001244970.1|	glucosamine-6-phosphate deaminase	*Coccidioides immitis RS*	9e-29
FE526230	gb|AAG01549.3|AF291822_1	multidrug resistance protein MDR	*Trichophyton rubrum*	2e-23
FE526231	gb|AAG24792.1|AF264028_2	pol protein	*Glomerella cingulata*	3e-44
FE526232	gb|AAP23304.1|	NIMA interactive protein	*Emericella nidulans*	1e-23
FE526233	gb|AAS45677.1|	subtilisin-like protease SUB5	*Trichophyton verrucosum*	6e-47
FE526234	gb|AAS45677.1|	subtilisin-like protease SUB5	*Trichophyton verrucosum*	8e-48
FE526235	ref|XP_001240454.1|	hypothetical protein CIMG_07617	*Coccidioides immitis RS*	3e-06
FE526236	ref|XP_751710.1|	kynurenine aminotransferase, putative	*Aspergillus fumigatus Af293*	9e-62
FE526237	ref|XP_001240454.1|	hypothetical protein CIMG_07617	*Coccidioides immitis RS*	4e-06

FE526238	gb|AAG01549.3|AF291822_1	multidrug resistance protein MDR	*Trichophyton rubrum*	2e-22
FE526239	gb|AAP23304.1|	NIMA interactive protein	*Emericella nidulans*	3e-24
FE526240	gb|AAP23304.1|	NIMA interactive protein	*Emericella nidulans*	4e-14
FE526241	gb|AAP23304.1|	NIMA interactive protein	*Emericella nidulans*	3e-24
FE526242	gb|AAG24792.1|AF264028_2	pol protein	*Glomerella cingulata*	3e-44
FE526243	ref|XP_001242594.1|	hypothetical protein CIMG_06490	*Coccidioides immitis RS*	6e-08
FE526244	gb|AAP23304.1|	NIMA interactive protein	*Emericella nidulans*	1e-23
FE526245	ref|XP_751710.1|	kynurenine aminotransferase, putative	*Aspergillus fumigatus Af293*	3e-27
FE526246	gb|AAG24792.1|AF264028_2	pol protein	*Glomerella cingulata*	2e-43
FE526247	gb|AAG01549.3|AF291822_1	multidrug resistance protein MDR	*Trichophyton rubrum*	2e-16
FE526248	dbj|BAC01275.1|	cytochrome P450nor	*Aspergillus oryzae*	6e-16
FE526249	ref|XP_001269482.1|	Leucine Rich Repeat domain protein	*Aspergillus clavatus NRRL 1*	3e-09
FE526250	ref|XP_001266920.1|	kynurenine aminotransferase, putative	*Neosartorya fischeri NRRL 181*	1e-62
FE526251	ref|XP_001211183.1|	DNA polymerase gamma	*Aspergillus terreus NIH2624*	4e-22
FE526252	gb|AAG01549.3|AF291822_1	multidrug resistance protein MDR	*Trichophyton rubrum*	9e-23
FE526253	gb|AAP23304.1|	NIMA interactive protein	*Emericella nidulans*	3e-27
FE526254	dbj|BAC01275.1|	cytochrome P450nor	*Aspergillus oryzae*	6e-16
FE526255	ref|XP_001266920.1|	kynurenine aminotransferase, putative	*Neosartorya fischeri NRRL 181*	8e-55
FE526256	ref|XP_001211183.1|	DNA polymerase gamma	*Aspergillus terreus NIH2624*	4e-22
FE526257	ref|XP_001265341.1|	ribosomal protein/carboxylic ester hydrolase (Ppe1), putative	*Neosartorya fischeri NRRL 181*	3e-25
FE526258	gb|AAP23304.1|	NIMA interactive protein	*Emericella nidulans*	8e-06
FE526259	dbj|BAC01275.1|	cytochrome P450nor	*Aspergillus oryzae*	1e-15
FE526260	ref|XP_001211183.1|	DNA polymerase gamma	*Aspergillus terreus NIH2624*	4e-22
FE526261	gb|AAG01549.3|AF291822_1	multidrug resistance protein MDR	*Trichophyton rubrum*	7e-23
FE526262	dbj|BAC01275.1|	cytochrome P450nor	*Aspergillus oryzae*	6e-16
FE526263	ref|XP_001265341.1|	ribosomal protein/carboxylic ester hydrolase (Ppe1), putative	*Neosartorya fischeri NRRL 181*	2e-24
FE526264	gb|AAG01549.3|AF291822_1	multidrug resistance protein MDR	*Trichophyton rubrum*	7e-23
FE526265	gb|AAG01549.3|AF291822_1	multidrug resistance protein MDR	*Trichophyton rubrum*	3e-21
FE526266	ref|XP_001265341.1|	ribosomal protein/carboxylic ester hydrolase (Ppe1), putative	*Neosartorya fischeri*	2e-24

			NRRL 181	
FE526267	ref|XP_001265341.1|	ribosomal protein/carboxylic ester hydrolase (Ppe1), putative	*Neosartorya fischeri NRRL 181*	2e-24
FE526268	gb|ABF22673.1|	copper-sulfate regulated protein 1	*Ajellomyces capsulatus*	7e-18
FE526269	gb|AAG01549.3|AF291822_1	multidrug resistance protein MDR	*Trichophyton rubrum*	2e-23
FE526270	ref|XP_001265341.1|	ribosomal protein/carboxylic ester hydrolase (Ppe1), putative	*Neosartorya fischeri NRRL 181*	6e-24
FE526271	ref|XP_001265341.1|	ribosomal protein/carboxylic ester hydrolase (Ppe1), putative	*Neosartorya fischeri NRRL 181*	2e-24
FE526272	gb|AAG01549.3|AF291822_1	multidrug resistance protein MDR	*Trichophyton rubrum*	4e-05
FE526273	gb|ABF22673.1|	copper-sulfate regulated protein 1	*Ajellomyces capsulatus*	7e-18
FE526274	gb|AAG01549.3|AF291822_1	multidrug resistance protein MDR	*Trichophyton rubrum*	7e-23
FE526275	ref|XP_754782.1|	hypothetical protein Afu3g08320	*Aspergillus fumigatus Af293*	3e-07
FE526276	ref|XP_001265341.1|	ribosomal protein/carboxylic ester hydrolase (Ppe1), putative	*Neosartorya fischeri NRRL 181*	2e-24
FE526277	ref|XP_001269482.1|	Leucine Rich Repeat domain protein	*Aspergillus clavatus NRRL 1*	3e-10
FE526278	-	No significant similarity	-	-
FE526279	gb|AAG01549.3|AF291822_1	multidrug resistance protein MDR	*Trichophyton rubrum*	2e-18
FE526280	gb|AAG24792.1|AF264028_2	pol protein	*Glomerella cingulata*	2e-43
FE526281	ref|XP_001240454.1|	hypothetical protein CIMG_07617	*Coccidioides immitis RS*	3e-06
FE526282	ref|XP_001269482.1|	Leucine Rich Repeat domain protein	*Aspergillus clavatus NRRL 1*	3e-10
FE526283	ref|XP_001240454.1|	hypothetical protein CIMG_07617	*Coccidioides immitis RS*	3e-06
FE526284	gb|AAS45677.1|	subtilisin-like protease SUB5	*Trichophyton verrucosum*	8e-48
FE526285	gb|AAP78735.1|	nonribosomal peptide synthase	*Alternaria brassicae*	5e-06
FE526286	gb|AAP23304.1|	NIMA interactive protein	*Emericella nidulans*	3e-25
FE526287	gb|AAP78735.1|	nonribosomal peptide synthase	*Alternaria brassicae*	5e-06
FE526288	ref|XP_001269482.1|	Leucine Rich Repeat domain protein	*Aspergillus clavatus NRRL 1*	3e-10
FE526289	gb|AAP23304.1|	NIMA interactive protein	*Emericella nidulans*	2e-24
FE526290	gb|AAP78735.1|	nonribosomal peptide synthase	*Alternaria brassicae*	5e-06
FE526291	gb|AAP23304.1|	NIMA interactive protein	*Emericella nidulans*	2e-25
FE526292	ref|XP_001269482.1|	Leucine Rich Repeat domain protein	*Aspergillus clavatus NRRL 1*	4e-10
FE526293	gb|AAP23304.1|	NIMA interactive protein	*Emericella nidulans*	6e-20

FE526294	gb|AAS45677.1|	subtilisin-like protease SUB5	*Trichophyton verrucosum*	3e-47
FE526295	gb|AAP78735.1|	nonribosomal peptide synthase	*Alternaria brassicae*	5e-06
FE526296	gb|AAP23304.1|	NIMA interactive protein	*Emericella nidulans*	2e-16
FE526297	gb|AAP78735.1|	nonribosomal peptide synthase	*Alternaria brassicae*	4e-07
FE526298	ref|XP_001269482.1|	Leucine Rich Repeat domain protein	*Aspergillus clavatus NRRL 1*	3e-10
FE526299	gb|AAP23304.1|	NIMA interactive protein	*Emericella nidulans*	2e-19
FE526300	gb|AAS45677.1|	subtilisin-like protease SUB5	*Trichophyton verrucosum*	8e-48
FE526301	ref|XP_001242594.1|	hypothetical protein CIMG_06490	*Coccidioides immitis RS*	2e-10
FE526302	gb|AAP23304.1|	NIMA interactive protein	*Emericella nidulans*	2e-11
FE526303	ref|XP_001242594.1|	hypothetical protein CIMG_06490	*Coccidioides immitis RS*	6e-08
FE526304	gb|AAS45677.1|	subtilisin-like protease SUB5	*Trichophyton verrucosum*	3e-49
FE526305	ref|XP_001242594.1|	hypothetical protein CIMG_06490	*Coccidioides immitis RS*	6e-08
FE526306	ref|YP_001098942.1|	glycerophosphodiester phosphodiesterase, cytosolic	*Herminiimonas arsenicoxydans*	1e-07
FE526307	ref|XP_001242594.1|	hypothetical protein CIMG_06490	*Coccidioides immitis RS*	6e-08
FE526308	ref|XP_001242594.1|	hypothetical protein CIMG_06490	*Coccidioides immitis RS*	6e-08
FE526309	ref|YP_001098942.1|	glycerophosphodiester phosphodiesterase, cytosolic	*Herminiimonas arsenicoxydans*	1e-07
FE526310	ref|XP_001242594.1|	hypothetical protein CIMG_06490	*Coccidioides immitis RS*	6e-08
FE526311	ref|XP_001269482.1|	Leucine Rich Repeat domain protein	*Aspergillus clavatus NRRL 1*	3e-10
FE526312	ref|YP_001098942.1|	glycerophosphodiester phosphodiesterase, cytosolic	*Herminiimonas arsenicoxydans*	1e-07
FE526313	ref|XP_001242594.1|	hypothetical protein CIMG_06490	*Coccidioides immitis RS*	6e-08
FE526314	gb|AAP78735.1|	nonribosomal peptide synthase	*Alternaria brassicae*	5e-06
FE526315	ref|XP_001269482.1|	Leucine Rich Repeat domain protein	*Aspergillus clavatus NRRL 1*	3e-10
FE526316	ref|YP_001098942.1|	glycerophosphodiester phosphodiesterase, cytosolic	*Herminiimonas arsenicoxydans*	1e-07
FE526317	-	No significant similarity	-	-
FE526318	ref|XP_001276510.1|	glucosamine-6-phosphate isomerase	*Aspergillus clavatus NRRL 1*	5e-46
FE526319	ref|XP_001261902.1|	oligopeptide transporter	*Neosartorya fischeri NRRL 181*	3e-40

FE526320	ref|XP_001273963.1|	MFS transporter, putative	*Aspergillus clavatus NRRL 1*	4e-11
FE526321	gb|AAS45677.1|	subtilisin-like protease SUB5	*Trichophyton verrucosum*	8e-48
FE526322	-	No significant similarity	-	-
FE526323	ref|XP_001261902.1|	oligopeptide transporter	*Neosartorya fischeri NRRL 181*	5e-37
FE526324	-	No significant similarity	-	-
FE526325	-	No significant similarity	-	-
FE526326	-	No significant similarity	-	-
FE526327	ref|XP_001275026.1|	oligopeptide transporter	*Aspergillus clavatus NRRL 1*	2e-13
FE526328	ref|XP_001261902.1|	oligopeptide transporter	*Neosartorya fischeri NRRL 181*	3e-28
FE526329	ref|XP_001245309.1|	hypothetical protein CIMG_04750	*Coccidioides immitis RS*	9e-07
FE526330	-	No significant similarity	-	-
FE526331	-	No significant similarity	-	-
FE526332	gb|AAS45677.1|	subtilisin-like protease SUB5	*Trichophyton verrucosum*	2e-47
FE526333	ref|XP_001261902.1|	oligopeptide transporter	*Neosartorya fischeri NRRL 181*	1e-34
FE526334	-	No significant similarity	-	-
FE526335	ref|YP_270051.1|	ISCps6, transposase	*Colwellia psychrerythraea 34H*	2e-11
FE526336	-	No significant similarity	-	-
FE526337	ref|XP_001264535.1|	MFS peptide transporter, putaitve	*Neosartorya fischeri NRRL 181*	5e-19
FE526338	gb|AAS45677.1|	subtilisin-like protease SUB5	*Trichophyton verrucosum*	8e-43
FE526339	-	No significant similarity	-	-
FE526340	-	No significant similarity	-	-
FE526341	gb|AAS45677.1|	subtilisin-like protease SUB5	*Trichophyton verrucosum*	5e-50
FE526342	ref|XP_752247.1|	cytochrome P450 phenylacetate hydroxylase, putative	*Aspergillus fumigatus Af293*	2e-18
FE526343	ref|XP_746951.1|	MFS peptide transporter, putative	*Aspergillus fumigatus Af293*	1e-39
FE526344	-	No significant similarity	-	-

FE526345	-	No significant similarity	-	-
FE526346	gb|AAZ32401.1|	peptide transporter PTR2A	*Hebeloma cylindrosporum*	1e-11
FE526347	gb|AAS45677.1|	subtilisin-like protease SUB5	*Trichophyton verrucosum*	4e-49
FE526348	ref|XP_001260782.1|	V-type ATPase, B subunit, putative	*Neosartorya fischeri NRRL 181*	1e-19
FE526349	gb|AAR02424.1|	subtilisin-like protease SUB5	*Trichophyton rubrum*	6e-46
FE526350	ref|XP_001260782.1|	V-type ATPase, B subunit, putative	*Neosartorya fischeri NRRL 181*	1e-20
FE526351	ref|XP_747457.1|	DUF895 domain membrane protein	*Aspergillus fumigatus Af293*	6e-10
FE526352	ref|XP_747214.1|	glucosamine-6-phosphate deaminase, putative	*Aspergillus fumigatus Af293*	6e-49
FE526353	ref|XP_001244970.1|	glucosamine-6-phosphate deaminase	*Coccidioides immitis RS*	8e-45
FE526354	ref|XP_001244970.1|	glucosamine-6-phosphate deaminase	*Coccidioides immitis RS*	5e-47
FE526355	-	No significant similarity	-	-
FE526356	gb|ABL84984.1|	metalloprotease Mep3	*Trichophyton equinum*	8e-41
FE526357	gb|AAS45677.1|	subtilisin-like protease SUB5	*Trichophyton verrucosum*	3e-72
FE526358	ref|XP_001246883.1|	hypothetical protein CIMG_00654	*Coccidioides immitis RS*	3e-04
FE526359	gb|AAR02424.1|	subtilisin-like protease SUB5	*Trichophyton rubrum*	4e-72
FE526360	ref|XP_001244970.1|	glucosamine-6-phosphate deaminase	*Coccidioides immitis RS*	9e-31
FE526361	ref|XP_001244025.1|	hypothetical protein CIMG_03466	*Coccidioides immitis RS*	3e-29
FE526362	ref|XP_001272561.1|	heat shock Hsp30-like protein, putative	*Aspergillus clavatus NRRL 1*	2e-07
FE526363	-	No significant similarity	-	-
FE526364	-	No significant similarity	-	-
FE526365	ref|XP_001261902.1|	oligopeptide transporter	*Neosartorya fischeri NRRL 181*	5e-40
FE526366	ref|XP_754004.1|	MFS transporter, putative	*Aspergillus fumigatus Af293*	4e-35
FE526367	-	No significant similarity	-	-
FE526368	-	No significant similarity	-	-
FE526369	ref|XP_001270309.1|	benzoate 4-monooxygenase cytochrome P450	*Aspergillus clavatus NRRL 1*	3e-35
FE526370	ref|XP_747214.1|	glucosamine-6-phosphate deaminase, putative	*Aspergillus fumigatus Af293*	2e-55

FE526371	gb|AAS45677.1|	subtilisin-like protease SUB5	*Trichophyton verrucosum*	6e-28
FE526372	-	No significant similarity	-	-
FE526373	ref|XP_001244025.1|	hypothetical protein CIMG_03466	*Coccidioides immitis RS*	4e-27
FE526374	ref|XP_001261902.1|	oligopeptide transporter	*Neosartorya fischeri NRRL 181*	2e-33
FE526375	gb|AAS45677.1|	subtilisin-like protease SUB5	*Trichophyton verrucosum*	1e-47
FE526376	ref|XP_001266791.1|	conserved lysine-rich protein, putative	*Neosartorya fischeri NRRL 181*	8e-04
FE526377	-	No significant similarity	-	-
FE526378	ref|XP_747457.1|	DUF895 domain membrane protein	*Aspergillus fumigatus Af293*	5e-12
FE526379	gb|AAR02424.1|	subtilisin-like protease SUB5	*Trichophyton rubrum*	5e-77
FE526380	ref|XP_001260782.1|	V-type ATPase, B subunit, putative	*Neosartorya fischeri NRRL 181*	3e-20
FE526381	gb|AAS45677.1|	subtilisin-like protease SUB5	*Trichophyton verrucosum*	4e-44
FE526382	ref|XP_001260782.1|	V-type ATPase, B subunit, putative	*Neosartorya fischeri NRRL 181*	5e-21
FE526383	gb|AAR02424.1|	subtilisin-like protease SUB5	*Trichophyton rubrum*	3e-45
FE526384	gb|AAS45677.1|	subtilisin-like protease SUB5	*Trichophyton verrucosum*	5e-41
FE526385	-	No significant similarity	-	-
FE526386	-	No significant similarity	-	-
FE526387	-	No significant similarity	-	-
FE526388	ref|XP_001261902.1|	oligopeptide transporter	*Neosartorya fischeri NRRL 181*	4e-21
FE526389	gb|AAS45677.1|	subtilisin-like protease SUB5	*Trichophyton verrucosum*	2e-48
FE526390	-	No significant similarity	-	-
FE526391	emb|CAJ83813.1|	CHK1 checkpoint homolog (S. pombe)	*Xenopus tropicalis*	7e-08
FE526392	-	No significant similarity	-	-
FE526393	ref|XP_001246883.1|	hypothetical protein CIMG_00654	*Coccidioides immitis RS*	3e-04
FE526394	ref|XP_001244025.1|	hypothetical protein CIMG_03466	*Coccidioides immitis RS*	1e-19
FE526395	ref|XP_001244970.1|	glucosamine-6-phosphate deaminase	*Coccidioides immitis RS*	3e-53
FE526396	gb|AAS45677.1|	subtilisin-like protease SUB5	*Trichophyton verrucosum*	3e-28

FE526397	ref|XP_001244025.1|	hypothetical protein CIMG_03466	*Coccidioides immitis RS*	9e-31
FE526398	gb|AAS45677.1|	subtilisin-like protease SUB5	*Trichophyton verrucosum*	2e-44
FE526399	-	No significant similarity	-	-
FE526400	-	No significant similarity	-	-
FE526401	ref|XP_001244970.1|	glucosamine-6-phosphate deaminase	*Coccidioides immitis RS*	6e-55
FE526402	ref|XP_001229086.1|	hypothetical protein CHGG_02570	*Chaetomium globosum CBS 148.51*	1e-03
FE526403	-	No significant similarity	-	-
FE526404	-	No significant similarity	-	-
FE526405	ref|XP_001246883.1|	hypothetical protein CIMG_00654	*Coccidioides immitis RS*	3e-04
FE526406	ref|YP_080386.1|	oxalate decarboxylase	*Bacillus licheniformis ATCC 14580*	2e-09
FE526407	ref|XP_001261902.1|	oligopeptide transporter	*Neosartorya fischeri NRRL 181*	2e-26
FE526408	-	No significant similarity	-	-
FE526409	gb|AAR02424.1|	subtilisin-like protease SUB5	*Trichophyton rubrum*	4e-41
FE526410	-	No significant similarity	-	-
FE526411	gb|ABL84992.1|	metalloprotease Mep4	*Trichophyton tonsurans*	5e-40
FE526412	-	No significant similarity	-	-
FE526413	gb|AAR11462.1|	subtilisin-like protease SUB3	*Trichophyton rubrum*	1e-24
FE526414	-	No significant similarity	-	-
FE526415	ref|XP_001265751.1|	phytanoyl-CoA dioxygenase family protein	*Neosartorya fischeri NRRL 181*	1e-18
FE526416	emb|CAJ04955.1|	hypothetical protein, unknown function	*Leishmania major*	7e-07
FE526417	gb|AAN62296.1|AF440524_83	transposase domain protein	*Pseudomonas aeruginosa*	1e-22
FE526418	ref|XP_001262224.1|	K+/H+ antiporter, putative	*Neosartorya fischeri NRRL 181*	4e-41
FE526419	ref|XP_001275855.1|	gamma interferon inducible lysosomal thiol reductase (GILT), putative	*Aspergillus clavatus NRRL 1*	3e-06
FE526420	-	No significant similarity	-	-
FE526421	ref|XP_001261902.1|	oligopeptide transporter	*Neosartorya fischeri NRRL 181*	2e-36
FE526422	-	No significant similarity	-	-
FE526423	gb|AAR11462.1|	subtilisin-like protease SUB3	*Trichophyton rubrum*	1e-21
FE526424	-	No significant similarity	-	-
FE526425	-	No significant similarity	-	-

FE526426	-	No significant similarity	-	-
FE526427	gb|AAS45677.1|	subtilisin-like protease SUB5	*Trichophyton verrucosum*	7e-40
FE526428	ref|XP_001261902.1|	oligopeptide transporter	*Neosartorya fischeri NRRL 181*	1e-59
FE526429	-	No significant similarity	-	-
FE526430	gb|AAS45677.1|	subtilisin-like protease SUB5	*Trichophyton verrucosum*	1e-39
FE526431	ref|XP_001275026.1|	oligopeptide transporter	*Aspergillus clavatus NRRL 1*	3e-39
FE526432	gb|AAR02424.1|	subtilisin-like protease SUB5	*Trichophyton rubrum*	3e-47
FE526433	ref|XP_448232.1|	unnamed protein product	*Candida glabrata*	3e-04
FE526434	-	No significant similarity	-	-
FE526435	ref|XP_001244970.1|	glucosamine-6-phosphate deaminase	*Coccidioides immitis RS*	2e-74
FE526436	ref|XP_001242709.1|	hypothetical protein CIMG_06605	*Coccidioides immitis RS*	8e-44
FE526437	ref|XP_754546.1|	Ribosomal protein S7e	*Aspergillus fumigatus Af293*	9e-31
FE526438	-	No significant similarity	-	-
FE526439	emb|CAE74488.1|	Hypothetical protein CBG22239	*Caenorhabditis briggsae*	2e-12
FE526440	ref|XP_001260782.1|	V-type ATPase, B subunit, putative	*Neosartorya fischeri NRRL 181*	7e-21
FE526441	-	No significant similarity	-	-
FE526442	ref|XP_001244025.1|	hypothetical protein CIMG_03466	*Coccidioides immitis RS*	7e-31
FE526443	ref|XP_001244970.1|	glucosamine-6-phosphate deaminase	*Coccidioides immitis RS*	7e-73
FE526444	ref|XP_001244970.1|	glucosamine-6-phosphate deaminase	*Coccidioides immitis RS*	8e-30
FE526445	gb|AAS45677.1|	subtilisin-like protease SUB5	*Trichophyton verrucosum*	2e-38
FE526446	ref|XP_001244970.1|	glucosamine-6-phosphate deaminase	*Coccidioides immitis RS*	4e-22
FE526447	ref|XP_001275026.1|	oligopeptide transporter	*Aspergillus clavatus NRRL 1*	8e-40
FE526448	ref|XP_661159.1|	30 kD heat shock protein	*Aspergillus nidulans FGSC A4*	7e-07
FE526449	ref|XP_001246883.1|	hypothetical protein CIMG_00654	*Coccidioides immitis RS*	1e-03
FE526450	gb|AAS45677.1|	subtilisin-like protease SUB5	*Trichophyton verrucosum*	3e-42
FE526451	ref|XP_001244025.1|	hypothetical protein CIMG_03466	*Coccidioides immitis RS*	7e-31
FE526452	ref|XP_001275026.1|	oligopeptide transporter	*Aspergillus clavatus NRRL 1*	2e-21

FE526453	-	No significant similarity	-	-
FE526454	gb|EEQ35176.1|	urease accessory protein UreD	*Microsporum canis CBS 113480*	8e-66
FE526455	-	No significant similarity	-	-
FE526456	ref|XP_001260782.1|	V-type ATPase, B subunit, putative	*Neosartorya fischeri NRRL 181*	8e-22
FE526457	ref|XP_001260782.1|	V-type ATPase, B subunit, putative	*Neosartorya fischeri NRRL 181*	8e-22
FE526458	gb|EEQ28888.1|	MFS oligopeptide transporter, putative	*Microsporum canis CBS 113480*	2e-19
FE526459	-	No significant similarity	-	-
FE526460	ref|XP_001261902.1|	oligopeptide transporter	*Neosartorya fischeri NRRL 181*	2e-31
FE526461	ref|NP_064450.2|	formin 2	*Homo sapiens*	9e-06
FE526462	-	No significant similarity	-	-
FE526463	-	No significant similarity	-	-
FE526464	gb|AAR11462.1|	subtilisin-like protease SUB3	*Trichophyton rubrum*	5e-25
FE526465	-	No significant similarity	-	-
FE526466	ref|XP_001260782.1|	V-type ATPase, B subunit, putative	*Neosartorya fischeri NRRL 181*	5e-21
FE526467	gb|AAR02424.1|	subtilisin-like protease SUB5	*Trichophyton rubrum*	1e-40
FE526468	-	No significant similarity	-	-
FE526469	ref|XP_001244970.1|	glucosamine-6-phosphate deaminase	*Coccidioides immitis RS*	1e-35
FE526470	ref|XP_001260782.1|	V-type ATPase, B subunit, putative	*Neosartorya fischeri NRRL 181*	5e-21
FE526471	-	No significant similarity	-	-
FE526472	ref|XP_001260782.1|	V-type ATPase, B subunit, putative	*Neosartorya fischeri NRRL 181*	5e-21
FE526473	gb|AAS45677.1|	subtilisin-like protease SUB5	*Trichophyton verrucosum*	3e-42
FE526474	ref|XP_001244970.1|	glucosamine-6-phosphate deaminase	*Coccidioides immitis RS*	2e-65
FE526475	gb|AAR02424.1|	subtilisin-like protease SUB5	*Trichophyton rubrum*	3e-45
FE526476	ref|XP_001244025.1|	hypothetical protein CIMG_03466	*Coccidioides immitis RS*	2e-44
FE526477	ref|XP_001260782.1|	V-type ATPase, B subunit, putative	*Neosartorya fischeri NRRL 181*	5e-21
FE526478	ref|XP_001261902.1|	oligopeptide transporter	*Neosartorya fischeri NRRL 181*	9e-43
FE526479	gb|AAR02424.1|	subtilisin-like protease SUB5	*Trichophyton rubrum*	2e-45

FE526480	ref|XP_001244970.1|	glucosamine-6-phosphate deaminase	*Coccidioides immitis RS*	1e-16
FE526481	ref|XP_001210481.1|	protein MGM1, mitochondrial precursor	*Aspergillus terreus NIH2624*	3e-41
FE526482	gb|AAS45677.1|	subtilisin-like protease SUB5	*Trichophyton verrucosum*	1e-44
FE526483	ref|XP_001275026.1|	oligopeptide transporter	*Aspergillus clavatus NRRL 1*	1e-44
FE526484	-	No significant similarity	-	-
FE526485	ref|XP_001244970.1|	glucosamine-6-phosphate deaminase	*Coccidioides immitis RS*	1e-28
FE526486	ref|XP_001260782.1|	V-type ATPase, B subunit, putative	*Neosartorya fischeri NRRL 181*	4e-20
FE526487	ref|XP_001261902.1|	oligopeptide transporter	*Neosartorya fischeri NRRL 181*	7e-67
FE526488	gb|AAS45677.1|	subtilisin-like protease SUB5	*Trichophyton verrucosum*	2e-78
FE526489	ref|XP_001275026.1|	oligopeptide transporter	*Aspergillus clavatus NRRL 1*	2e-48
FE526490	ref|XP_001244970.1|	glucosamine-6-phosphate deaminase	*Coccidioides immitis RS*	6e-28
FE526491	ref|XP_001275026.1|	oligopeptide transporter	*Aspergillus clavatus NRRL 1*	1e-52
FE526492	ref|XP_001275026.1|	oligopeptide transporter	*Aspergillus clavatus NRRL 1*	8e-40
FE526493	gb|AAR11462.1|	subtilisin-like protease SUB3	*Trichophyton rubrum*	1e-24
FE526494	ref|XP_001246302.1|	hypothetical protein CIMG_00073	*Coccidioides immitis RS*	1e-25
FE526495	ref|XP_001275026.1|	oligopeptide transporter	*Aspergillus clavatus NRRL 1*	7e-39
FE526496	ref|XP_001264535.1|	MFS peptide transporter, putaitve	*Neosartorya fischeri NRRL 181*	1e-18
FE526497	gb|EAX07174.1|	hCG1793893	*Homo sapiens*	2e-05
FE526498	ref|XP_747457.1|	DUF895 domain membrane protein	*Aspergillus fumigatus Af293*	5e-26
FE526499	ref|XP_001211350.1|	sterol 24-C-methyltransferase	*Aspergillus terreus NIH2624*	7e-23
FE526500	gb|AAS45677.1|	subtilisin-like protease SUB5	*Trichophyton verrucosum*	2e-48
FE526501	ref|XP_001260782.1|	V-type ATPase, B subunit, putative	*Neosartorya fischeri NRRL 181*	3e-19
FE526502	gb|AAS45677.1|	subtilisin-like protease SUB5	*Trichophyton*	6e-29

			verrucosum	
FE526503	ref|XP_001260782.1|	V-type ATPase, B subunit, putative	*Neosartorya fischeri NRRL 181*	3e-20
FE526504	ref|XP_001276510.1|	glucosamine-6-phosphate isomerase	*Aspergillus clavatus NRRL 1*	4e-37
FE526505	ref|XP_747457.1|	DUF895 domain membrane protein	*Aspergillus fumigatus Af293*	3e-11
FE526506	ref|XP_970785.1|	PREDICTED: similar to CG6957-PA, isoform A	*Tribolium castaneum*	3e-34
FE526507	ref|XP_001261902.1|	oligopeptide transporter	*Neosartorya fischeri NRRL 181*	2e-17
FE526508	ref|XP_001271762.1|	short chain dehydrogenase, putative	*Aspergillus clavatus NRRL 1*	1e-25
FE526509	ref|XP_001261901.1|	extracellular lipase, putative	*Neosartorya fischeri NRRL 181*	5e-19
FE526510	ref|XP_001244025.1|	hypothetical protein CIMG_03466	*Coccidioides immitis RS*	7e-08
FE526511	gb|AAS45677.1|	subtilisin-like protease SUB5	*Trichophyton verrucosum*	7e-25
FE526512	ref|XP_553256.2|	ENSANGP00000027824	*Anopheles gambiae str. PEST*	6e-13
FE526513	emb|CAK48124.1|	unnamed protein product	*Aspergillus niger*	4e-12
FE526514	ref|XP_001218693.1|	cholinesterase	*Aspergillus terreus NIH2624*	2e-08
FE526515	gb|EEQ29740.1|	Aminoacid permease	*Microsporum canis*	1e-24
FE526516	ref|XP_001244025.1|	hypothetical protein CIMG_03466	*Coccidioides immitis RS*	1e-24
FE526517	ref|XP_001275026.1|	oligopeptide transporter	*Aspergillus clavatus NRRL 1*	6e-22
FE526518	-	No significant similarity	-	-
FE526519	ref|XP_001245309.1|	hypothetical protein CIMG_04750	*Coccidioides immitis RS*	1e-07
FE526520	ref|XP_001244970.1|	glucosamine-6-phosphate deaminase	*Coccidioides immitis RS*	1e-20
FE526521	ref|XP_755153.1|	flotillin domain protein	*Aspergillus fumigatus Af293*	4e-16
FE526522	ref|XP_001246883.1|	hypothetical protein CIMG_00654	*Coccidioides immitis RS*	3e-04
FE526523	-	No significant similarity	-	-
FE526524	ref|XP_001246883.1|	hypothetical protein CIMG_00654	*Coccidioides immitis RS*	3e-04
FE526525	gb|AAS45677.1|	subtilisin-like protease SUB5	*Trichophyton verrucosum*	1e-45
FE526526	gb|AAS45677.1|	subtilisin-like protease SUB5	*Trichophyton verrucosum*	1e-46

FE526527	gb|AAR02424.1|	subtilisin-like protease SUB5	*Trichophyton rubrum*	4e-47
FE526528	gb|AAS45677.1|	subtilisin-like protease SUB5	*Trichophyton verrucosum*	2e-47
FE526529	-	No significant similarity	-	-
FE526530	-	No significant similarity	-	-
FE526531	-	No significant similarity	-	-
FE526532	ref|XP_001275026.1|	oligopeptide transporter	*Aspergillus clavatus NRRL 1*	1e-40
FE526533	gb|AAS45677.1|	subtilisin-like protease SUB5	*Trichophyton verrucosum*	2e-48
FE526534	ref|XP_001275026.1|	oligopeptide transporter	*Aspergillus clavatus NRRL 1*	1e-45
FE526535	-	No significant similarity	-	-
FE526536	gb|AAS45677.1|	subtilisin-like protease SUB5	*Trichophyton verrucosum*	5e-46
FE526537	-	No significant similarity	-	-
FE526538	-	No significant similarity	-	-
FE526539	-	No significant similarity	-	-
FE526540	gb|AAS45677.1|	subtilisin-like protease SUB5	*Trichophyton verrucosum*	1e-34
FE526541	gb|AAR08135.1|	small GTPase RanA	*Emericella nidulans*	2e-40
FE526542	-	No significant similarity	-	-
FE526543	ref|XP_001208665.1|	vacuolar ATP synthase subunit B	*Aspergillus terreus NIH2624*	2e-17
FE526544	-	No significant similarity	-	-
FE526545	-	No significant similarity	-	-
FE526546	-	No significant similarity	-	-
FE526547	ref|XP_001275026.1|	oligopeptide transporter	*Aspergillus clavatus NRRL 1*	7e-37
FE526548	ref|XP_001244970.1|	glucosamine-6-phosphate deaminase	*Coccidioides immitis RS*	8e-61
FE526549	-	No significant similarity	-	-
FE526550	gb|AAS45677.1|	subtilisin-like protease SUB5	*Trichophyton verrucosum*	1e-44
FE526551	-	No significant similarity	-	-
FE526552	ref|XP_747457.1|	DUF895 domain membrane protein	*Aspergillus fumigatus Af293*	4e-18
FE526553	gb|ABL84992.1|	metalloprotease Mep4	*Trichophyton tonsurans*	4e-23
FE526554	-	No significant similarity	-	-

FE526555	ref|XP_001269482.1|	Leucine Rich Repeat domain protein	*Aspergillus clavatus NRRL 1*	9e-09
FE526556	ref|XP_754782.1|	hypothetical protein Afu3g08320	*Aspergillus fumigatus Af293*	2e-05
FE526557	ref|XP_001244184.1|	hypothetical protein CIMG_03625	*Coccidioides immitis RS*	9e-49
FE526558	ref|XP_001209001.1|	predicted protein	*Aspergillus terreus NIH2624*	2e-05
FE526559	-	No significant similarity	-	-
FE526560	-	No significant similarity	-	-
FE526561	ref|XP_001244184.1|	hypothetical protein CIMG_03625	*Coccidioides immitis RS*	1e-51
FE526562	ref|XP_001242594.1|	hypothetical protein CIMG_06490	*Coccidioides immitis RS*	6e-08
FE526563	gb|AAP23304.1|	NIMA interactive protein	*Emericella nidulans*	4e-15
FE526564	ref|XP_001269482.1|	Leucine Rich Repeat domain protein	*Aspergillus clavatus NRRL 1*	2e-05
FE526565	ref|YP_840033.1|	Glyoxalase/bleomycin resistance protein/dioxygenase	*Burkholderia cenocepacia HI2424*	4e-11
FE526566	ref|YP_840033.1|	Glyoxalase/bleomycin resistance protein/dioxygenase	*Burkholderia cenocepacia HI2424*	3e-11
FE526567	ref|XP_001242594.1|	hypothetical protein CIMG_06490	*Coccidioides immitis RS*	6e-08
FE526568	gb|EEQ33141.1|	NIMA interactive protein	*Microsporum canis CBS 113480*	2e-23
FE526569	-	No significant similarity	-	-
FE526570	-	No significant similarity	-	4e-05
FE526571	gb|AAP23304.1|	NIMA interactive protein	*Emericella nidulans*	6e-25
FE526572	ref|XP_001240454.1|	hypothetical protein CIMG_07617	*Coccidioides immitis RS*	8e-07
FE526573	gb|AAP23304.1|	NIMA interactive protein	*Emericella nidulans*	6e-25
FE526574	gb|AAG01549.3|AF291822_1	multidrug resistance protein MDR	*Trichophyton rubrum*	3e-22
FE526575	gb|AAP23304.1|	NIMA interactive protein	*Emericella nidulans*	9e-27
FE526576	-	No significant similarity	-	-
FE526577	ref|YP_840033.1|	Glyoxalase/bleomycin resistance protein/dioxygenase	*Burkholderia cenocepacia HI2424*	3e-11
FE526578	gb|AAP23304.1|	NIMA interactive protein	*Emericella nidulans*	6e-25
FE526579	-	No significant similarity	-	-
FE526580	gb|ABF22673.1|	copper-sulfate regulated protein 1	*Ajellomyces capsulatus*	7e-18
FE526581	ref|ZP_01556758.1|	aldehyde dehydrogenase	*Burkholderia ambifaria MC40-6*	5e-65
FE526582	ref|YP_371113.1|	Aldehyde dehydrogenase	*Burkholderia sp. 383*	7e-78
FE526583	ref|XP_001242594.1|	hypothetical protein CIMG_06490	*Coccidioides immitis RS*	6e-08

FE526584	-	No significant similarity	-	-
FE526585	gb|AAP23304.1|	NIMA interactive protein	*Emericella nidulans*	6e-25
FE526586	-	No significant similarity	-	-
FE526587	-	No significant similarity	-	-
FE526588	gb|AAP23304.1|	NIMA interactive protein	*Emericella nidulans*	6e-25
FE526589	-	No significant similarity	-	-
FE526590	gb|AAP23304.1|	NIMA interactive protein	*Emericella nidulans*	3e-27
FE526591	gb|AAP23304.1|	NIMA interactive protein	*Emericella nidulans*	1e-18
FE526592	gb|AAP23304.1|	NIMA interactive protein	*Emericella nidulans*	7e-10
FE526593	gb|AAG24792.1|AF264028_2	pol protein	*Glomerella cingulata*	2e-28
FE526594	gb|ABF22673.1|	copper-sulfate regulated protein 1	*Ajellomyces capsulatus*	7e-18
FE526595	ref|XP_001269482.1|	Leucine Rich Repeat domain protein	*Aspergillus clavatus NRRL 1*	4e-09
FE526596	gb|AAP23304.1|	NIMA interactive protein	*Emericella nidulans*	7e-27
FE526597	gb|AAP23304.1|	NIMA interactive protein	*Emericella nidulans*	2e-16
FE526598	gb|AAG01549.3|AF291822_1	multidrug resistance protein MDR	*Trichophyton rubrum*	2e-14
FE526599	-	No significant similarity	-	-
FE526600	gb|AAG24792.1|AF264028_2	pol protein	*Glomerella cingulata*	5e-42
FE526601	gb|AAP78735.1|	nonribosomal peptide synthase	*Alternaria brassicae*	5e-06
FE526602	ref|XP_001244184.1|	hypothetical protein CIMG_03625	*Coccidioides immitis RS*	7e-53
FE526603	gb|AAG01549.3|AF291822_1	multidrug resistance protein MDR	*Trichophyton rubrum*	2e-23
FE526604	-	No significant similarity	-	-
FE526605	ref|XP_001269482.1|	Leucine Rich Repeat domain protein	*Aspergillus clavatus NRRL 1*	2e-06
FE526606	gb|AAP23304.1|	NIMA interactive protein	*Emericella nidulans*	8e-26
FE526607	gb|AAP23304.1|	NIMA interactive protein	*Emericella nidulans*	3e-11
FE526608	gb|AAG24792.1|AF264028_2	pol protein	*Glomerella cingulata*	4e-38
FE526609	gb|AAP23304.1|	NIMA interactive protein	*Emericella nidulans*	1e-15
FE526610	gb|AAG24792.1|AF264028_2	pol protein	*Glomerella cingulata*	6e-38
FE526611	gb|AAG24792.1|AF264028_2	pol protein	*Glomerella cingulata*	4e-43
FE526612	emb|CAK37677.1|	unnamed protein product	*Aspergillus niger*	6e-46
FE526613	ref|XP_001240454.1|	hypothetical protein CIMG_07617	*Coccidioides immitis RS*	6e-06
FE526614	-	No significant similarity	-	2e-04
FE526615	ref|XP_001244184.1|	hypothetical protein CIMG_03625	*Coccidioides immitis RS*	8e-36
FE526616	gb|AAP23304.1|	NIMA interactive protein	*Emericella nidulans*	1e-26
FE526617	-	No significant similarity	-	-
FE526618	-	No significant similarity	-	-
FE526619	gb|AAG24792.1|AF264028_2	pol protein	*Glomerella cingulata*	6e-20

FE526620	ref|XP_001239812.1|	hypothetical protein CIMG_09433	*Coccidioides immitis RS*	4e-08
FE526621	ref|ZP_01420399.1|	O-antigen polymerase	*Caulobacter sp. K31*	4e-17
FE526622	gb|AAG24792.1|AF264028_2	pol protein	*Glomerella cingulata*	5e-43
FE526623	gb|AAP23304.1|	NIMA interactive protein	*Emericella nidulans*	6e-25
FE526624	gb|AAP23304.1|	NIMA interactive protein	*Emericella nidulans*	4e-20
FE526625	-	No significant similarity	-	-
FE526626	-	No significant similarity	-	-
FE526627	gb|ABF22673.1|	copper-sulfate regulated protein 1	*Ajellomyces capsulatus*	7e-18
FE526628	gb|AAP78735.1|	nonribosomal peptide synthase	*Alternaria brassicae*	5e-06
FE526629	ref|XP_001269482.1|	Leucine Rich Repeat domain protein	*Aspergillus clavatus NRRL 1*	1e-07
FE526630	gb|AAP78735.1|	nonribosomal peptide synthase	*Alternaria brassicae*	5e-06
FE526631	gb|AAP23304.1|	NIMA interactive protein	*Emericella nidulans*	2e-24
FE526632	-	No significant similarity	-	-
FE526633	gb|AAP23304.1|	NIMA interactive protein	*Emericella nidulans*	6e-25
FE526634	gb|AAP23304.1|	NIMA interactive protein	*Emericella nidulans*	1e-22
FE526635	ref|YP_371113.1|	Aldehyde dehydrogenase	*Burkholderia sp. 383*	3e-75
FE526636	-	No significant similarity	-	-
FE526637	-	No significant similarity	-	-
FE526638	ref|XP_001244184.1|	hypothetical protein CIMG_03625	*Coccidioides immitis RS*	3e-49
FE526639	gb|AAP23304.1|	NIMA interactive protein	*Emericella nidulans*	7e-20
FE526640	-	No significant similarity	-	-
FE526641	ref|XP_001244184.1|	hypothetical protein CIMG_03625	*Coccidioides immitis RS*	2e-48
FE526642	ref|XP_001264989.1|	leucine rich repeat protein	*Neosartorya fischeri NRRL 181*	3e-09
FE526643	gb|AAP23304.1|	NIMA interactive protein	*Emericella nidulans*	1e-17
FE526644	gb|ABF22673.1|	copper-sulfate regulated protein 1	*Ajellomyces capsulatus*	7e-10
FE526645	-	No significant similarity	-	-
FE526646	ref|XP_001269482.1|	Leucine Rich Repeat domain protein	*Aspergillus clavatus NRRL 1*	3e-04
FE526647	gb|AAL26311.2|AF362957_1	polyprotein	*Aspergillus flavus*	2e-31
FE526648	-	No significant similarity	-	-
FE526649	-	No significant similarity	-	-
FE526650	-	No significant similarity	-	-
FE526651	-	No significant similarity	-	-
FE526652	-	No significant similarity	-	-
FE526653	ref|YP_371113.1|	Aldehyde dehydrogenase	*Burkholderia sp. 383*	3e-77
FE526654	gb|AAG01549.3|AF291822_1	multidrug resistance protein MDR	*Trichophyton rubrum*	1e-22

FE526655	-	No significant similarity	-	-
FE526656	ref|XP_001264989.1|	leucine rich repeat protein	*Neosartorya fischeri NRRL 181*	1e-04
FE526657	gb|AAP23304.1|	NIMA interactive protein	*Emericella nidulans*	7e-25
FE526658	ref|XP_001240454.1|	hypothetical protein CIMG_07617	*Coccidioides immitis RS*	1e-05
FE526659	gb|AAP23304.1|	NIMA interactive protein	*Emericella nidulans*	2e-19
FE526660	gb|AAG01549.3|AF291822_1	multidrug resistance protein MDR	*Trichophyton rubrum*	6e-23
FE526661	gb|AAG24792.1|AF264028_2	pol protein	*Glomerella cingulata*	2e-33
FE526662	gb|AAG24792.1|AF264028_2	pol protein	*Glomerella cingulata*	5e-28
FE526663	gb|AAP23304.1|	NIMA interactive protein	*Emericella nidulans*	4e-16
FE526664	gb|AAR29046.2|	gag-pol polyprotein	*Aspergillus flavus*	1e-28
FE526665	-	No significant similarity	-	-
FE526666	gb|AAG24792.1|AF264028_2	pol protein	*Glomerella cingulata*	1e-33
FE526667	gb|AAP23304.1|	NIMA interactive protein	*Emericella nidulans*	1e-22
FE526668	gb|AAG24792.1|AF264028_2	pol protein	*Glomerella cingulata*	4e-42
FE526669	-	No significant similarity	-	-
FE526670	-	No significant similarity	-	-
FE526671	ref|XP_001226392.1|	hypothetical protein CHGG_08465	*Chaetomium globosum CBS 148.51*	4e-10
FE526672	gb|AAP23304.1|	NIMA interactive protein	*Emericella nidulans*	2e-23
FE526673	gb|AAP23304.1|	NIMA interactive protein	*Emericella nidulans*	2e-16
FE526674	gb|AAG01549.3|AF291822_1	multidrug resistance protein MDR	*Trichophyton rubrum*	7e-20
FE526675	gb|AAP23304.1|	NIMA interactive protein	*Emericella nidulans*	5e-25
FE526676	-	No significant similarity	-	-
FE526677	ref|YP_443455.1|	secretion protein, putative	*Burkholderia thailandensis E264*	3e-04
FE526678	-	No significant similarity	-	-
FE526679	-	No significant similarity	-	-
FE526680	-	No significant similarity	-	-
FE526681	gb|AAN62296.1|AF440524_83	transposase domain protein	*Pseudomonas aeruginosa*	8e-22
FE526682	-	No significant similarity	-	-
FE526683	ref|XP_001262797.1|	FYVE zinc finger protein	*Neosartorya fischeri NRRL 181*	2e-09
FE526684	ref|XP_001262797.1|	FYVE zinc finger protein	*Neosartorya fischeri NRRL 181*	4e-26
FE526685	ref|XP_001246883.1|	hypothetical protein CIMG_00654	*Coccidioides immitis RS*	3e-04
FE526686	-	No significant similarity	-	-

FE526687	-	No significant similarity	-	-
FE526688	-	No significant similarity	-	-
FE526689	-	No significant similarity	-	-
FE526690	ref|XP_001260782.1|	V-type ATPase, B subunit, putative	*Neosartorya fischeri NRRL 181*	5e-21
FE526691	gb|AAN62296.1|AF440524_83	transposase domain protein	*Pseudomonas aeruginosa*	2e-22
FE526692	ref|XP_001262797.1|	FYVE zinc finger protein	*Neosartorya fischeri NRRL 181*	4e-12
FE526693	-	No significant similarity	-	-
FE526694	-	No significant similarity	-	-
FE526695	-	No significant similarity	-	-
FE526696	-	No significant similarity	-	-
FE526697	ref|XP_001262797.1|	FYVE zinc finger protein	*Neosartorya fischeri NRRL 181*	1e-27
FE526698	ref|XP_001262797.1|	FYVE zinc finger protein	*Neosartorya fischeri NRRL 181*	2e-27
FE526699	-	No significant similarity	-	-
FE526700	-	No significant similarity	-	-
FE526701	ref|XP_001264529.1|	GABA permease, putative	*Neosartorya fischeri NRRL 181*	3e-17
FE526702	ref|XP_001264853.1|	amino acid permease, putative	*Neosartorya fischeri NRRL 181*	3e-26
FE526703	ref|XP_001262797.1|	FYVE zinc finger protein	*Neosartorya fischeri NRRL 181*	6e-26
FE526704	-	No significant similarity	-	-
FE526705	-	No significant similarity	-	-
FE526706	-	No significant similarity	-	-
FE526707	ref|XP_001263899.1|	60S ribosomal protein L20, putative	*Neosartorya fischeri NRRL 181*	5e-11
FE526708	-	No significant similarity	-	-
FE526709	ref|XP_001262797.1|	FYVE zinc finger protein	*Neosartorya fischeri NRRL 181*	2e-22
FE526710	ref|XP_001262797.1|	FYVE zinc finger protein	*Neosartorya fischeri NRRL 181*	8e-13
FE526711	-	No significant similarity	-	-
FE526712	ref|YP_443455.1|	secretion protein, putative	*Burkholderia thailandensis E264*	3e-04

FE526713	-	No significant similarity	-	-
FE526714	-	No significant similarity	-	-
FE526715	-	No significant similarity	-	-
FE526716	-	No significant similarity	-	-
FE526717	gb|AAN62296.1|AF440524_83	transposase domain protein	*Pseudomonas aeruginosa*	2e-22
FE526718	gb|AAF21678.1|AF051915_2	pol polyprotein	*Cladosporium fulvum*	1e-09
FE526719	ref|XP_001263899.1|	60S ribosomal protein L20, putative	*Neosartorya fischeri NRRL 181*	5e-11
FE526720	-	No significant similarity	-	-
FE526721	-	No significant similarity	-	-
FE526722	-	No significant similarity	-	-
FE526723	-	No significant similarity	-	-
FE526724	ref|XP_001263899.1|	60S ribosomal protein L20, putative	*Neosartorya fischeri NRRL 181*	5e-11
FE526725	-	No significant similarity	-	-
FE526726	ref|XP_001260782.1|	V-type ATPase, B subunit, putative	*Neosartorya fischeri NRRL 181*	5e-21
FE526727	-	No significant similarity	-	-
FE526728	-	No significant similarity	-	-
FE526729	-	No significant similarity	-	-
FE526730	ref|YP_443455.1|	secretion protein, putative	*Burkholderia thailandensis E264*	4e-04
FE526731	-	No significant similarity	-	-
FE526732	ref|XP_001246883.1|	hypothetical protein CIMG_00654	*Coccidioides immitis RS*	3e-04
FE526733	-	No significant similarity	-	-
FE526734	-	No significant similarity	-	-
FE526735	-	No significant similarity	-	-
FE526736	-	No significant similarity	-	-
FE526737	-	No significant similarity	-	-
FE526738	ref|XP_001246883.1|	hypothetical protein CIMG_00654	*Coccidioides immitis RS*	3e-04
FE526739	-	No significant similarity	-	-
FE526740	ref|XP_001246883.1|	hypothetical protein CIMG_00654	*Coccidioides immitis RS*	3e-04
FE526741	gb|EEQ32880.1|	FYVE zinc finger protein	*Microsporum canis* CBS 113480	1e-34
FE526742	-	No significant similarity	-	-
FE526743	-	No significant similarity	-	-
FE526744	-	No significant similarity	-	-

FE526745	-	No significant similarity	-	-
FE526746	-	No significant similarity	-	-
FE526747	-	No significant similarity	-	-
FE526748	ref|XP_001262797.1|	FYVE zinc finger protein	*Neosartorya fischeri NRRL 181*	9e-14
FE526749	ref|XP_001272385.1|	FYVE zinc finger protein	*Aspergillus clavatus NRRL 1*	3e-04
FE526750	-	No significant similarity	-	-
FE526751	gb|AAN62296.1|AF440524_83	transposase domain protein	*Pseudomonas aeruginosa*	2e-22
FE526752	-	No significant similarity	-	-
FE526753	-	No significant similarity	-	-
FE526754	-	No significant similarity	-	-
FE526755	-	No significant similarity	-	-
FE526756	-	No significant similarity	-	-
FE526757	ref|XP_001242709.1|	hypothetical protein CIMG_06605	*Coccidioides immitis RS*	3e-10
FE526758	ref|XP_753997.1|	60s ribosomal protein yl6	*Aspergillus fumigatus Af293*	1e-29
FE526759	-	No significant similarity	-	-
FE526760	ref|XP_753233.1|	ThiJ/PfpI family protein	*Aspergillus fumigatus Af293*	9e-07
FE526761	-	No significant similarity	-	-
FE526762	ref|XP_001243030.1|	hypothetical protein CIMG_06926	*Coccidioides immitis RS*	2e-06
FE526763	sp|Q462Q7|COX19_PARBR	Cytochrome c oxidase assembly protein COX19 gb|AAY64182.1| Cox19p	*Paracoccidioides brasiliensis*	1e-12
FE526764	ref|XP_001259622.1|	pentatricopeptide repeat protein	*Neosartorya fischeri NRRL 181*	1e-20
FE526765	-	No significant similarity	-	-
FE526766	-	No significant similarity	-	-
FE526767	-	No significant similarity	-	-
FE526768	-	No significant similarity	-	-
FE526769	ref|XP_001259414.1|	50S ribosomal protein L4	*Neosartorya fischeri NRRL 181*	7e-05
FE526770	-	No significant similarity	-	-
FE526771	gb|AAG36933.1|AF262955_1	oleate delta-12 desaturase	*Emericella nidulans*	9e-21
FE526772	ref|XP_001248270.1|	hypothetical protein CIMG_02041	*Coccidioides immitis RS*	1e-04
FE526773	ref|XP_001212165.1|	HNRNP arginine N-methyltransferase	*Aspergillus terreus NIH2624*	3e-16

FE526774	-	No significant similarity	-	-
FE526775	ref|XP_001269624.1|	ADP,ATP carrier protein	*Aspergillus clavatus NRRL 1*	2e-23
FE526776	-	No significant similarity	-	-
FE526777	ref|XP_001268371.1|	CECR1 family adenosine deaminase, putative	*Aspergillus clavatus NRRL 1*	4e-14
FE526778	-	No significant similarity	-	-
FE526779	ref|XP_001260012.1|	conserved hypothetical protein	*Neosartorya fischeri NRRL 181*	3e-07
FE526780	ref|XP_001257300.1|	monocarboxylate permease homologue, mch4	*Neosartorya fischeri NRRL 181*	3e-08
FE526781	-	No significant similarity	-	-
FE526782	ref|XP_001260419.1|	60S ribosomal protein L10	*Neosartorya fischeri NRRL 181*	1e-34
FE526783	ref|XP_001275190.1|	amino acid transporter, putative	*Aspergillus clavatus NRRL 1*	4e-13
FE526784	-	No significant similarity	-	-
FE526785	-	No significant similarity	-	-
FE526786	ref|XP_001245093.1|	hypothetical protein CIMG_04534	*Coccidioides immitis RS*	3e-07
FE526787	ref|XP_726427.1|	RNA 3l-terminal phosphate cyclase protein	*Plasmodium yoelii yoelii str. 17XNL*	7e-04
FE526788	ref|XP_001272674.1|	taurine catabolism dioxygenase TauD, TfdA family protein	*Aspergillus clavatus NRRL 1*	1e-24
FE526789	-	No significant similarity	-	-
FE526790	ref|XP_001257403.1|	benzoate 4-monooxygenase cytochrome P450	*Neosartorya fischeri NRRL 181*	1e-12
FE526791	ref|XP_001267237.1|	60S ribosomal protein L38, putative	*Neosartorya fischeri NRRL 181*	5e-14
FE526792	ref|XP_001273971.1|	60S ribosomal protein L8, putative	*Aspergillus clavatus NRRL 1*	9e-23
FE526793	-	No significant similarity	-	-
FE526794	ref|XP_001246583.1|	succinate-semialdehyde dehydrogenase	*Coccidioides immitis RS*	3e-27
FE526795	ref|XP_001267237.1|	60S ribosomal protein L38, putative	*Neosartorya fischeri NRRL 181*	2e-14
FE526796	ref|XP_001272771.1|	40S ribosomal protein S11	*Aspergillus clavatus NRRL 1*	2e-06
FE526797	-	No significant similarity	-	-
FE526798	ref|XP_001267248.1|	60S ribosomal protein L11	*Neosartorya fischeri*	2e-19

			NRRL 181	
FE526799	-	No significant similarity	-	-
FE526800	-	No significant similarity	-	-
FE526801	-	No significant similarity	-	-
FE526802	ref|XP_001213404.1|	predicted protein	*Aspergillus terreus NIH2624*	4e-15
FE526803	-	No significant similarity	-	-
FE526804	-	No significant similarity	-	-
FE526805	ref|XP_001267237.1|	60S ribosomal protein L38, putative	*Neosartorya fischeri NRRL 181*	1e-13
FE526806	gb|ABH10644.1|	aconitase	*Coccidioides posadasii*	2e-42
FE526807	-	No significant similarity	-	-
FE526808	gb|EAT82743.1|	predicted protein	*Phaeosphaeria nodorum SN15*	6e-04
FE526809	-	No significant similarity	-	-
FE526810	ref|XP_001267762.1|	glutathione S-transferase, putative	*Aspergillus clavatus NRRL 1*	1e-23
FE526811	-	No significant similarity	-	-
FE526812	-	No significant similarity	-	-
FE526813	ref|XP_001248510.1|	alanyl-tRNA synthetase, cytoplasmic	*Coccidioides immitis RS*	1e-17
FE526814	-	No significant similarity	-	-
FE526815	ref|XP_001243359.1|	hypothetical protein CIMG_07255	*Coccidioides immitis RS*	7e-04
FE526816	gb|EDJ95143.1|	60S ribosomal protein L7	*Magnaporthe grisea 70-15*	1e-09
FE526817	ref|XP_001216898.1|	ADP-ribose pyrophosphatase	*Aspergillus terreus NIH2624*	2e-22
FE526818	ref|XP_001260458.1|	SNARE protein (Ufe1), putative	*Neosartorya fischeri NRRL 181*	5e-09
FE526819	-	No significant similarity	-	-
FE526820	ref|XP_001212923.1|	60S ribosomal protein L20	*Aspergillus terreus NIH2624*	2e-05
FE526821	ref|XP_001213571.1|	mitochondrial genome maintenance protein MGM101	*Aspergillus terreus NIH2624*	9e-48
FE526822	ref|XP_001260097.1|	cytochrome b5, putative	*Neosartorya fischeri NRRL 181*	6e-16
FE526823	ref|XP_001215797.1|	multicopy enhancer of UAS2	*Aspergillus terreus NIH2624*	5e-50
FE526824	sp|P34763|NMT_AJECA	Glycylpeptide N-tetradecanoyltransferase (Peptide N-myristoyltransferase)	*Ajellomyces capsulatus*	6e-24

		(Myristoyl-CoA:protein N-myristoyltransferase) (NMT) gb|AAA17549.1| N-myristoyltransferase		
FE526825	ref|XP_001269377.1|	DUF814 domain protein	*Aspergillus clavatus NRRL 1*	2e-08
FE526826	ref|XP_001247536.1|	lysyl-tRNA synthetase, cytoplasmic	*Coccidioides immitis RS*	4e-19
FE526827	gb|EAL91735.2|	integral membrane protein	*Aspergillus fumigatus Af293*	4e-23
FE526828	ref|XP_001240306.1|	cytochrome P450 51	*Coccidioides immitis RS*	1e-63
FE526829	-	No significant similarity	-	-
FE526830	ref|XP_752041.1|	Ribosomal L38e protein family	*Aspergillus fumigatus Af293*	5e-14
FE526831	ref|XP_001244411.1|	hypothetical protein CIMG_03852	*Coccidioides immitis RS*	6e-04
FE526832	-	No significant similarity	-	-
FE526833	-	No significant similarity	-	-
FE526834	ref|XP_001259852.1|	WD repeat protein	*Neosartorya fischeri NRRL 181*	2e-22
FE526835	gb|EAL90446.2|	eukaryotic translation elongation factor 1 subunit Eef1-beta, putative	*Aspergillus fumigatus Af293*	2e-13
FE526836	-	No significant similarity	-	-
FE526837	ref|XP_001273261.1|	tropomyosin, putative	*Aspergillus clavatus NRRL 1*	6e-35
FE526838	-	No significant similarity	-	-
FE526839	-	No significant similarity	-	-
FE526840	-	No significant similarity	-	-
FE526841	ref|XP_001260419.1|	60S ribosomal protein L10	*Neosartorya fischeri NRRL 181*	7e-37
FE526842	-	No significant similarity	-	-
FE526843	-	No significant similarity	-	-
FE526844	ref|XP_751502.1|	HAD superfamily hydrolase, putative	*Aspergillus fumigatus Af293*	3e-39
FE526845	emb|CAK97343.1|	unnamed protein product	*Aspergillus niger*	4e-13
FE526846	-	No significant similarity	-	-
FE526847	ref|XP_001270525.1|	conserved hypothetical protein	*Aspergillus clavatus NRRL 1*	9e-07
FE526848	gb|EAL90446.2|	eukaryotic translation elongation factor 1 subunit Eef1-beta, putative	*Aspergillus fumigatus Af293*	4e-13
FE526849	ref|XP_753997.1|	60s ribosomal protein yl6	*Aspergillus fumigatus Af293*	5e-30

FE526850	-	No significant similarity	-	-
FE526851	ref|XP_001274066.1|	60S ribosome biogenesis protein Sqt1, putative	*Aspergillus clavatus NRRL 1*	7e-21
FE526852	ref|XP_001214720.1|	outer mitochondrial membrane protein porin	*Aspergillus terreus NIH2624*	4e-19
FE526853	ref|XP_001257403.1|	benzoate 4-monooxygenase cytochrome P450	*Neosartorya fischeri NRRL 181*	1e-13
FE526854	-	No significant similarity	-	-
FE526855	sp|P07509|SODC_NEUCR	Superoxide dismutase	*Neurospora crassa*	2e-13
FE526856	gb|ABN50029.1|	putative senescence-associated protein	*Trichosanthes dioica*	2e-05
FE526857	ref|XP_001268698.1|	succinate dehydrogenase subunit CybS, putative	*Aspergillus clavatus NRRL 1*	4e-07
FE526858	ref|XP_001211720.1|	60S ribosomal protein L12	*Aspergillus terreus NIH2624*	9e-10
FE526859	-	No significant similarity	-	-
FE526860	-	No significant similarity	-	-
FE526861	-	No significant similarity	-	-
FE526862	-	No significant similarity	-	-
FE526863	-	No significant similarity	-	-
FE526864	ref|XP_001271235.1|	DUF1713 domain protein	*Aspergillus clavatus NRRL 1*	3e-07
FE526865	ref|XP_001246620.1|	40S ribosomal protein S4	*Coccidioides immitis RS*	1e-26
FE526866	ref|XP_001262838.1|	indoleamine 2,3-dioxygenase family protein	*Neosartorya fischeri NRRL 181*	1e-47
FE526867	ref|XP_001387342.1|	60S large subunit ribosomal protein	*Pichia stipitis CBS 6054*	4e-04
FE526868	emb|CAK45164.1|	protein O-mannosyl transferase pmtA-Aspergillus niger	*Aspergillus niger*	1e-27
FE526869	ref|XP_753997.1|	60s ribosomal protein yl6	*Aspergillus fumigatus Af293*	2e-17
FE526870	gb|EAL85168.2|	U5 snRNP component Snu114, putative	*Aspergillus fumigatus Af293*	1e-18
FE526871	ref|XP_001246620.1|	40S ribosomal protein S4	*Coccidioides immitis RS*	1e-26
FE526872	ref|XP_001269346.1|	60S ribosomal protein L9, putative	*Aspergillus clavatus NRRL 1*	2e-20
FE526873	ref|XP_001243698.1|	fibrillarin	*Coccidioides immitis RS*	7e-50
FE526874	ref|XP_001264284.1|	kelch repeat protein	*Neosartorya fischeri NRRL 181*	6e-15
FE526875	-	No significant similarity	-	-
FE526876	-	No significant similarity	-	-

FE526877	-	No significant similarity	-	-
FE526878	ref|XP_001246583.1|	succinate-semialdehyde dehydrogenase	*Coccidioides immitis RS*	3e-27
FE526879	ref|XP_001265211.1|	Ribosomal L18ae protein family	*Neosartorya fischeri NRRL 181*	6e-16
FE526880	-	No significant similarity	-	-
FE526881	-	No significant similarity	-	-
FE526882	ref|XP_001260103.1|	WD repeat protein	*Neosartorya fischeri NRRL 181*	3e-05
FE526883	gb|EDJ98154.1|	GTP cyclohydrolase II, putative	*Magnaporthe grisea 70-15*	3e-50
FE526884	gb|EEQ30276.1|	mitochondrial 2-methylisocitrate lyase	*Microsporum canis CBS113480*	9e-31
FE526885	-	No significant similarity	-	-
FE526886	ref|XP_661668.1|	ADP/ATP carrier protein	*Aspergillus nidulans FGSC A4*	2e-39
FE526887	ref|XP_001178408.1|	PREDICTED: similar to translation initiation factor 2 gamma subunit	*Strongylocentrotus purpuratus*	8e-17
FE526888	ref|XP_001270945.1|	PDCD2_C domain protein, putative	*Aspergillus clavatus NRRL 1*	2e-08
FE526889	ref|XP_001244571.1|	40S ribosomal protein S26E	*Coccidioides immitis RS*	4e-10
FE526890	-	No significant similarity	-	-
FE526891	-	No significant similarity	-	-
FE526892	-	No significant similarity	-	-
FE526893	-	No significant similarity	-	-
FE526894	-	No significant similarity	-	-
FE526895	-	No significant similarity	-	-
FE526896	-	No significant similarity	-	-
FE526897	emb|CAD29478.1|	glutathione transferase F5	*Triticum aestivum*	7e-07
FE526898	-	No significant similarity	-	-
FE526899	gb|AAM54368.1|	elongation factor 1-alpha	*Trichophyton rubrum*	7e-20
FE526900	-	No significant similarity	-	-
FE526901	ref|XP_661668.1|	ADP/ATP carrier protein	*Aspergillus nidulans FGSC A4*	1e-40
FE526902	sp|Q5BGF9|PAM17_EMENI	Presequence translocated-associated motor subunit pam17, mitochondrial precursor	*Emericella nidulans*	8e-22
FE526903	-	No significant similarity	-	-
FE526904	-	No significant similarity	-	-
FE526905	gb|AAS76666.1|	carboxypeptidase S1	*Trichophyton rubrum*	5e-48

FE526906	sp|Q5AZJ7|DPH1_EMENI	Diphthamide biosynthesis protein	*Emericella nidulans*	3e-36
FE526907	ref|XP_001273971.1|	60S ribosomal protein L8, putative	*Aspergillus clavatus NRRL 1*	2e-12
FE526908	ref|XP_001246620.1|	40S ribosomal protein S4	*Coccidioides immitis RS*	6e-28
FE526909	ref|XP_001266080.1|	seryl-tRNA synthetase	*Neosartorya fischeri NRRL 181*	4e-26
FE526910	ref|XP_001240451.1|	aspartyl aminopeptidase, putative	*Coccidioides immitis RS*	5e-47
FE526911	-	No significant similarity	-	-
FE526912	-	No significant similarity	-	-
FE526913	-	No significant similarity	-	-
FE526914	-	No significant similarity	-	-
FE526915	-	No significant similarity	-	-
FE526916	ref|XP_001244035.1|	hypothetical protein CIMG_03476	*Coccidioides immitis RS*	2e-12
FE526917	ref|XP_001246620.1|	40S ribosomal protein S4	*Coccidioides immitis RS*	6e-28
FE526918	-	No significant similarity	-	-
FE526919	gb|EAL90446.2|	eukaryotic translation elongation factor 1 subunit Eef1-beta, putative	*Aspergillus fumigatus Af293*	6e-19
FE526920	emb|CAC18218.1|	probable TRANSKETOLASE	*Neurospora crassa*	6e-22
FE526921	-	No significant similarity	-	-
FE526922	gb|ABH11414.1|	peroxin 3	*Penicillium chrysogenum*	1e-07
FE526923	-	No significant similarity	-	-
FE526924	ref|XP_567138.1|	siderochrome-iron (ferrioxamine) uptake transporter	*Cryptococcus neoformans var. neoformans JEC21*	5e-21
FE526925	ref|XP_001258439.1|	60S ribosomal protein L7a	*Neosartorya fischeri NRRL 181*	1e-08
FE526926	gb|ABH10638.1|	enolase	*Coccidioides posadasii*	5e-29
FE526927	ref|XP_001261926.1|	ELMO/CED-12 family protein	*Neosartorya fischeri NRRL 181*	1e-28
FE526928	-	No significant similarity	-	-
FE526929	-	No significant similarity	-	-
FE526930	-	No significant similarity	-	-
FE526931	ref|XP_001241489.1|	hypothetical protein CIMG_08652	*Coccidioides immitis RS*	3e-23
FE526932	ref|XP_001248308.1|	60S ribosomal protein L28	*Coccidioides immitis RS*	4e-22
FE526933	ref|XP_001241872.1|	hypothetical protein CIMG_05768	*Coccidioides immitis RS*	2e-05
FE526934	ref|XP_753437.1|	integral membrane protein, putative	*Aspergillus fumigatus Af293*	5e-05

FE526935	-	No significant similarity	-	-
FE526936	ref|XP_001248510.1|	alanyl-tRNA synthetase, cytoplasmic	*Coccidioides immitis RS*	6e-17
FE526937	ref|XP_001267248.1|	60S ribosomal protein L11	*Neosartorya fischeri NRRL 181*	4e-21
FE526938	ref|XP_001266988.1|	phosphatase-like protein (PTPLA), putative	*Neosartorya fischeri NRRL 181*	7e-13
FE526939	ref|XP_001220066.1|	40S ribosomal protein S11	*Chaetomium globosum CBS 148.51*	3e-04
FE526940	-	No significant similarity	-	-
FE526941	-	No significant similarity	-	-
FE526942	-	No significant similarity	-	-
FE526943	ref|XP_001270945.1|	PDCD2_C domain protein, putative	*Aspergillus clavatus NRRL 1*	3e-07
FE526944	gb|EAL91735.2|	integral membrane protein	*Aspergillus fumigatus Af293*	2e-24
FE526945	ref|XP_001247826.1|	2-oxoglutarate dehydrogenase E1 component	*Coccidioides immitis RS*	3e-15
FE526946	-	No significant similarity	-	-
FE526947	-	No significant similarity	-	-
FE526948	gb|EDJ95143.1|	60S ribosomal protein L7	*Magnaporthe grisea 70-15*	1e-12
FE526949	-	No significant similarity	-	-
FE526950	-	No significant similarity	-	-
FE526951	-	No significant similarity	-	-
FE526952	ref|XP_001269007.1|	oleate delta-12 desaturase	*Aspergillus clavatus NRRL 1*	7e-13
FE526953	ref|XP_001273282.1|	mitochondrial ribosomal protein DAP3, putative	*Aspergillus clavatus NRRL 1*	2e-12
FE526954	ref|XP_001246620.1|	40S ribosomal protein S4	*Coccidioides immitis RS*	6e-28
FE526955	ref|XP_001265779.1|	fatty acid desaturase, putative	*Neosartorya fischeri NRRL 181*	2e-28
FE526956	ref|XP_001247300.1|	hypothetical protein CIMG_01071	*Coccidioides immitis RS*	1e-05
FE526957	ref|XP_001267424.1|	phosphoethanolamine	*Neosartorya fischeri NRRL 181*	8e-22
FE526958	ref|XP_001242955.1|	hypothetical protein CIMG_06851	*Coccidioides immitis RS*	1e-08
FE526959	-	No significant similarity	-	-
FE526960	gb|EDJ95143.1|	60S ribosomal protein L7	*Magnaporthe grisea 70-15*	8e-14
FE526961	-	No significant similarity	-	-

FE526962	ref|XP_001259336.1|	cell cycle control protein (Cwf8), putative	*Neosartorya fischeri NRRL 181*	1e-27
FE526963	ref|XP_001210588.1|	U3 small nucleolar ribonucleoprotein protein IMP4	*Aspergillus terreus NIH2624*	5e-43
FE526964	ref|XP_001261302.1|	C2 domain protein	*Neosartorya fischeri NRRL 181*	5e-29
FE526965	ref|XP_001212323.1|	predicted protein	*Aspergillus terreus NIH2624*	8e-09
FE526966	ref|XP_001267850.1|	antigenic mitochondrial protein HSP60, putative	*Aspergillus clavatus NRRL 1*	5e-13
FE526967	ref|XP_001244490.1|	ADP,ATP carrier protein	*Coccidioides immitis RS*	8e-19
FE526968	ref|XP_001248397.1|	hypothetical protein CIMG_02168	*Coccidioides immitis RS*	2e-05
FE526969	gb|AAL50803.1|AF452883_1	Y20 protein	*Paracoccidioides brasiliensis*	6e-30
FE526970	-	No significant similarity	-	-
FE526971	-	No significant similarity	-	-
FE526972	pir||S63701	mannosyl-oligosaccharide 1,2-alpha-mannosidase (EC 3.2.1.113) precursor	*Aspergillus phoenicis*	2e-13
FE526973	ref|XP_001242456.1|	hypothetical protein CIMG_06352	*Coccidioides immitis RS*	1e-04
FE526974	ref|XP_001242392.1|	plasma membrane ATPase	*Coccidioides immitis RS*	3e-43
FE526975	gb|AAT40563.1|	small G-protein GPA3	*Paracoccidioides brasiliensis*	2e-29
FE526976	ref|XP_001271126.1|	ubiquitin	*Aspergillus clavatus NRRL 1*	3e-23
FE526977	ref|XP_001266080.1|	seryl-tRNA synthetase	*Neosartorya fischeri NRRL 181*	3e-25
FE526978	-	No significant similarity	-	-
FE526979	emb|CAM37639.1|	proteophosphoglycan ppg1	*Leishmania braziliensis*	1e-23
FE526980	ref|XP_001269939.1|	eukaryotic translation initiation factor 3 subunit EifCb, putative	*Aspergillus clavatus NRRL 1*	2e-36
FE526981	ref|XP_001248308.1|	60S ribosomal protein L28	*Coccidioides immitis RS*	8e-24
FE526982	-	No significant similarity	-	-
FE526983	ref|XP_001248096.1|	acetamidase	*Coccidioides immitis RS*	6e-33
FE526984	-	No significant similarity	-	-
FE526985	ref|XP_001241489.1|	hypothetical protein CIMG_08652	*Coccidioides immitis RS*	7e-26
FE526986	-	No significant similarity	-	-
FE526987	-	No significant similarity	-	-
FE526988	-	No significant similarity	-	-
FE526989	ref|XP_001257403.1|	benzoate 4-monooxygenase cytochrome P450	*Neosartorya fischeri*	1e-13

			NRRL 181	
FE526990	-	No significant similarity	-	-
FE526991	-	No significant similarity	-	-
FE526992	-	No significant similarity	-	-
FE526993	-	No significant similarity	-	-
FE526994	ref|XP_001386580.1|	membrane protein involved in vacuolar protein sorting	*Pichia stipitis CBS 6054*	3e-07
FE526995	-	No significant similarity	-	-
FE526996	-	No significant similarity	-	-
FE526997	ref|XP_001268779.1|	BAP31 domain protein, putative	*Aspergillus clavatus NRRL 1*	8e-14
FE526998	ref|XP_001263178.1|	ubiquitin conjugating enzyme (UbcB), putative	*Neosartorya fischeri NRRL 181*	3e-08
FE526999	ref|XP_001271404.1|	heat shock protein Hsp30/Hsp42, putative	*Aspergillus clavatus NRRL 1*	2e-08
FE527000	ref|XP_001248200.1|	predicted protein	*Coccidioides immitis RS*	1e-09
FE527001	-	No significant similarity	-	-
FE527002	-	No significant similarity	-	-
FE527003	-	No significant similarity	-	-
FE527004	-	No significant similarity	-	-
FE527005	-	No significant similarity	-	-
FE527006	ref|XP_001275968.1|	60S ribosomal protein L37a	*Aspergillus clavatus NRRL 1*	7e-21
FE527007	-	No significant similarity	-	-
FE527008	-	No significant similarity	-	-
FE527009	-	No significant similarity	-	-
FE527010	ref|XP_001273971.1|	60S ribosomal protein L8, putative	*Aspergillus clavatus NRRL 1*	9e-26
FE527011	-	No significant similarity	-	-
FE527012	-	No significant similarity	-	-
FE527013	ref|XP_001240365.1|	hypothetical protein CIMG_07528	*Coccidioides immitis RS*	3e-05
FE527014	-	No significant similarity	-	-
FE527015	-	No significant similarity	-	-
FE527016	ref|XP_001259429.1|	dihydroxyacetone kinase (DakA), putative	*Neosartorya fischeri NRRL 181*	5e-10
FE527017	-	No significant similarity	-	-
FE527018	-	No significant similarity	-	-
FE527019	-	No significant similarity	-	-
FE527020	-	No significant similarity	-	-

FE527021	ref|XP_001266736.1|	MFS monosaccharide transporter, putative	*Neosartorya fischeri NRRL 181*	3e-20
FE527022	-	No significant similarity	-	-
FE527023	-	No significant similarity	-	-
FE527024	-	No significant similarity	-	-
FE527025	ref|XP_001248308.1|	60S ribosomal protein L28	*Coccidioides immitis RS*	2e-21
FE527026	ref|XP_001242709.1|	hypothetical protein CIMG_06605	*Coccidioides immitis RS*	4e-10
FE527027	ref|XP_001245288.1|	heat shock protein hsp1	*Coccidioides immitis RS*	2e-11
FE527028	ref|XP_001248308.1|	60S ribosomal protein L28	*Coccidioides immitis RS*	2e-21
FE527029	-	No significant similarity	-	-
FE527030	-	No significant similarity	-	-
FE527031	emb|CAD70291.1|	related to mitochondrial serine--tRNA ligase	*Neurospora crassa*	7e-05
FE527032	-	No significant similarity	-	-
FE527033	ref|XP_001387342.1|	60S large subunit ribosomal protein	*Pichia stipitis CBS 6054*	3e-04
FE527034	gb|AAK52822.1|AF365926_1	calmodulin-binding coil-coil protein	*Emericella nidulans*	4e-07
FE527035	ref|XP_001387342.1|	60S large subunit ribosomal protein	*Pichia stipitis CBS 6054*	3e-04
FE527036	ref|XP_001268653.1|	TIM barrel metal-dependent hydrolase, putative	*Aspergillus clavatus NRRL 1*	3e-07
FE527037	-	No significant similarity	-	-
FE527038	ref|XP_001265779.1|	fatty acid desaturase, putative	*Neosartorya fischeri NRRL 181*	9e-29
FE527039	ref|XP_753997.1|	60s ribosomal protein yl6	*Aspergillus fumigatus Af293*	1e-29
FE527040	emb|CAA49847.1|	GMP synthase	*Saccharomyces cerevisiae*	7e-12
FE527041	ref|XP_001262785.1|	MFS peptide transporter Ptr2, putative	*Neosartorya fischeri NRRL 181*	1e-15
FE527042	-	No significant similarity	-	-
FE527043	-	No significant similarity	-	-
FE527044	ref|XP_001387342.1|	60S large subunit ribosomal protein	*Pichia stipitis CBS 6054*	3e-04
FE527045	-	No significant similarity	-	-
FE527046	-	No significant similarity	-	-
FE527047	-	No significant similarity	-	-
FE527048	ref|XP_754922.1|	cytosolic small ribosomal subunit S4, putative	*Aspergillus fumigatus Af293*	2e-19
FE527049	-	No significant similarity	-	-
FE527050	-	No significant similarity	-	-
FE527051	-	No significant similarity	-	-

FE527052	ref|XP_001273971.1|	60S ribosomal protein L8, putative	*Aspergillus clavatus NRRL 1*	9e-26
FE527053	gb|EDK03984.1|	mitochondrial NADH-ubiquinone oxidoreductase 20 kD subunit	*Magnaporthe grisea 70-15*	8e-27
FE527054	-	No significant similarity	-	-
FE527055	ref|XP_001218101.1|	seryl-tRNA synthetase	*Aspergillus terreus NIH2624*	2e-08
FE527056	-	No significant similarity	-	-
FE527057	ref|XP_001273971.1|	60S ribosomal protein L8, putative	*Aspergillus clavatus NRRL 1*	3e-23
FE527058	ref|XP_001265211.1|	Ribosomal L18ae protein family	*Neosartorya fischeri NRRL 181*	2e-15
FE527059	ref|XP_001261939.1|	Hsp70 chaperone (HscA), putative	*Neosartorya fischeri NRRL 181*	1e-16
FE527060	ref|XP_001241456.1|	hypothetical protein CIMG_08619	*Coccidioides immitis RS*	2e-05
FE527061	-	No significant similarity	-	-
FE527062	-	No significant similarity	-	-
FE527063	-	No significant similarity	-	-
FE527064	-	No significant similarity	-	-
FE527065	-	No significant similarity	-	-
FE527066	-	No significant similarity	-	-
FE527067	ref|XP_001240912.1|	hypothetical protein CIMG_08075	*Coccidioides immitis RS*	2e-06
FE527068	ref|XP_001268219.1|	3-oxoacyl-(acyl-carrier-protein) reductase	*Aspergillus clavatus NRRL 1*	1e-21
FE527069	ref|XP_001268984.1|	PfkB family carbohydrate kinase (Mak32), putative	*Aspergillus clavatus NRRL 1*	1e-14
FE527070	ref|XP_001215828.1|	tRNA (uridine-2l-O-)-methyltransferase TRM7	*Aspergillus terreus NIH2624*	5e-13
FE527071	-	No significant similarity	-	-
FE527072	ref|XP_001266337.1|	phosphate transporter (Pho88), putative	*Neosartorya fischeri NRRL 181*	6e-07
FE527073	-	No significant similarity	-	-
FE527074	gb|EAT88174.1|	hypothetical protein SNOG_04414	*Phaeosphaeria nodorum SN15*	3e-04
FE527075	-	No significant similarity	-	-
FE527076	ref|XP_001260419.1|	60S ribosomal protein L10	*Neosartorya fischeri NRRL 181*	2e-28
FE527077	-	No significant similarity	-	-

FE527078	gb|AAP22960.1|	14-3-3-like protein	*Paracoccidioides brasiliensis*	9e-07
FE527079	sp|Q8J1M3|DPP5_ARTBE	Dipeptidyl-peptidase 5 precursor (Dipeptidyl-peptidase V) (DPP V) (DppV) (Allergen Tri m 4) emb|CAD23611.1| tri m 4 allergen	*Arthroderma benhamiae*	5e-21
FE527080	-	No significant similarity	-	-
FE527081	-	No significant similarity	-	-
FE527082	dbj|BAB33421.1|	putative senescence-associated protein	*Pisum sativum*	1e-26
FE527083	-	No significant similarity	-	-
FE527084	ref|XP_001273971.1|	60S ribosomal protein L8, putative	*Aspergillus clavatus NRRL 1*	2e-16
FE527085	-	No significant similarity	-	-
FE527086	ref|XP_001274406.1|	DNA-directed RNA polymerases i, ii, and iii 145 kDa polypeptide	*Aspergillus clavatus NRRL 1*	2e-16
FE527087	ref|XP_747704.1|	prpd protein	*Aspergillus fumigatus Af293*	1e-24
FE527088	-	No significant similarity	-	-
FE527089	-	No significant similarity	-	-
FE527090	-	No significant similarity	-	-
FE527091	ref|XP_001267123.1|	60S ribosomal protein L25, putative	*Neosartorya fischeri NRRL 181*	4e-18
FE527092	-	No significant similarity	-	-
FE527093	-	No significant similarity	-	-
FE527094	sp|A1CSR1|MRH4_ASPCL	ATP-dependent RNA helicase mrh4, mitochondrial precursor	*Aspergillus clavatus*	2e-14
FE527095	ref|XP_001266736.1|	MFS monosaccharide transporter, putative	*Neosartorya fischeri NRRL 181*	7e-21
FE527096	ref|XP_753520.1|	D-amino acid oxidase	*Aspergillus fumigatus Af293*	4e-11
FE527097	gb|ABE01845.1|	beta-tubulin	*Microsporum canis*	7e-15
FE527098	-	No significant similarity	-	-
FE527099	-	No significant similarity	-	-
FE527100	gb|EAT76933.1|	hypothetical protein SNOG_15558	*Phaeosphaeria nodorum SN15*	1e-05
FE527101	-	No significant similarity	-	-
FE527102	ref|XP_001268336.1|	protein transport protein Sec24, putative	*Aspergillus clavatus NRRL 1*	2e-20
FE527103	ref|XP_001209399.1|	succinate/fumarate mitochondrial transporter	*Aspergillus terreus NIH2624*	1e-32
FE527104	ref|XP_001273981.1|	vacuolar protein sorting protein DigA	*Aspergillus clavatus*	4e-24

			NRRL 1	
FE527105	-	No significant similarity	-	-
FE527106	ref|XP_001270945.1|	PDCD2_C domain protein, putative	*Aspergillus clavatus NRRL 1*	1e-08
FE527107	ref|XP_001257579.1|	2-methylcitrate dehydratase, putative	*Neosartorya fischeri NRRL 181*	3e-12
FE527108	ref|XP_001264551.1|	RING finger ubiquitin ligase (Tul1), putative	*Neosartorya fischeri NRRL 181*	2e-09
FE527109	dbj|BAC01275.1|	cytochrome P450nor	*Aspergillus oryzae*	1e-19
FE527110	ref|XP_001275968.1|	60S ribosomal protein L37a	*Aspergillus clavatus NRRL 1*	7e-21
FE527111	-	No significant similarity	-	-
FE527112	ref|XP_001273487.1|	short-chain dehydrogenase	*Aspergillus clavatus NRRL 1*	1e-05
FE527113	ref|XP_001264756.1|	adenosylhomocysteinase	*Neosartorya fischeri NRRL 181*	5e-23
FE527114	-	No significant similarity	-	-
FE527115	-	No significant similarity	-	-
FE527116	-	No significant similarity	-	-
FE527117	-	No significant similarity	-	-
FE527118	ref|XP_001267248.1|	60S ribosomal protein L11	*Neosartorya fischeri NRRL 181*	6e-22
FE527119	ref|XP_001273971.1|	60S ribosomal protein L8, putative	*Aspergillus clavatus NRRL 1*	3e-27
FE527120	-	No significant similarity	-	-
FE527121	-	No significant similarity	-	-
FE527122	-	No significant similarity	-	-
FE527123	-	No significant similarity	-	-
FE527124	ref|XP_001387342.1|	60S large subunit ribosomal protein	*Pichia stipitis CBS 6054*	3e-04
FE527125	-	No significant similarity	-	-
FE527126	ref|XP_001271844.1|	WD repeat protein	*Aspergillus clavatus NRRL 1*	1e-07
FE527127	ref|XP_001268989.1|	multidrug resistance protein 1, 2, 3 (p glycoprotein 1, 2, 3)	*Aspergillus clavatus NRRL 1*	2e-09
FE527128	ref|XP_001240756.1|	hypothetical protein CIMG_07919	*Coccidioides immitis RS*	2e-06
FE527129	-	No significant similarity	-	-
FE527130	-	No significant similarity	-	-
FE527131	ref|XP_752593.1|	mitochondrial hypoxia responsive domain protein	*Aspergillus fumigatus*	2e-33

			Af293	
FE527132	ref|XP_001265914.1|	sugar transporter, putative	*Neosartorya fischeri NRRL 181*	1e-06
FE527133	ref|XP_001269812.1|	adenylate kinase 2	*Aspergillus clavatus NRRL 1*	9e-07
FE527134	-	No significant similarity	-	-
FE527135	ref|XP_001544887.1|	conserved hypothetical protein	*Ajellomyces capsulatus NAm1*	4e-11
FE527136	ref|XP_001541343.1|	conserved hypothetical protein	*Ajellomyces capsulatus NAm1*	2e-05
FE527137	ref|XP_001271103.1|	Bromodomain protein	*Aspergillus clavatus NRRL 1*	3e-15
FE527138	ref|XP_001248747.1|	predicted protein	*Coccidioides immitis RS*	4e-08
FE527139	ref|XP_001536346.1|	40S ribosomal protein S18	*Ajellomyces capsulatus NAm1*	1e-31
FE527140	-	No significant similarity	-	-
FE527141	ref|XP_001217407.1|	conserved hypothetical protein	*Aspergillus terreus NIH2624*	1e-06
FE527142	-	No significant similarity	-	-
FE527143	-	No significant similarity	-	-
FE527144	ref|XP_001543575.1|	amino acid permease Dip5	*Ajellomyces capsulatus NAm1*	3e-26
FE527145	ref|XP_001542093.1|	sodium transport ATPase 5	*Ajellomyces capsulatus NAm1*	1e-12
FE527146	-	No significant similarity	-	-
FE527147	-	No significant similarity	-	-
FE527148	-	No significant similarity	-	-
FE527149	-	No significant similarity	-	-
FE527150	-	No significant similarity	-	-
FE527151	ref|XP_752484.2|	eukaryotic translation elongation factor 1 subunit Eef1-beta, putative	*Aspergillus fumigatus Af293*	5e-13
FE527152	ref|XP_001257858.1|	isocitrate dehydrogenase LysB	*Neosartorya fischeri NRRL 181*	2e-47
FE527153	-	No significant similarity	-	-
FE527154	-	No significant similarity	-	-
FE527155	-	No significant similarity	-	-
FE527156	ref|XP_001268212.1|	PF02656 domain protein	*Aspergillus clavatus NRRL 1*	2e-13

FE527157	ref|XP_001248747.1|	predicted protein	*Coccidioides immitis RS*	1e-07
FE527158	ref|XP_001260492.1|	SET and MYND domain protein, putative	*Neosartorya fischeri NRRL 181*	6e-06
FE527159	ref|XP_001273282.1|	mitochondrial ribosomal protein DAP3, putative	*Aspergillus clavatus NRRL 1*	2e-12
FE527160	ref|XP_001273062.1|	non-classical export protein Nce2, putative	*Aspergillus clavatus NRRL 1*	5e-10
FE527161	ref|XP_001273744.1|	arginine permease	*Aspergillus clavatus NRRL 1*	1e-20
FE527162	ref|XP_001536307.1|	predicted protein	*Ajellomyces capsulatus NAm1*	1e-10
FE527163	ref|XP_001259189.1|	F-box domain protein	*Neosartorya fischeri NRRL 181*	7e-25
FE527164	ref|XP_001537488.1|	ER-derived vesicles protein ERV14	*Ajellomyces capsulatus NAm1*	2e-07
FE527165	-	No significant similarity	-	-
FE527166	-	No significant similarity	-	-
FE527167	ref|XP_750288.1|	mitochondrial ADP,ATP carrier protein (Ant), putative	*Aspergillus fumigatus Af293*	4e-22
FE527168	-	No significant similarity	-	-
FE527169	ref|XP_001248747.1|	predicted protein	*Coccidioides immitis RS*	3e-06
FE527170	ref|XP_001265779.1|	fatty acid desaturase, putative	*Neosartorya fischeri NRRL 181*	4e-29
FE527171	gb|ABH10645.1|	ATP synthase beta chain	*Coccidioides posadasii*	4e-21
FE527172	ref|XP_001536346.1|	40S ribosomal protein S18	*Ajellomyces capsulatus NAm1*	1e-26
FE527173	ref|XP_001211936.1|	26S protease regulatory subunit 8	*Aspergillus terreus NIH2624*	9e-12
FE527174	ref|XP_001537882.1|	thiamine-phosphate pyrophosphorylase	*Ajellomyces capsulatus NAm1*	2e-17
FE527175	ref|XP_001260814.1|	translation elongation factor EF-2 subunit, putative	*Neosartorya fischeri NRRL 181*	4e-11
FE527176	-	No significant similarity	-	-
FE527177	-	No significant similarity	-	-
FE527178	ref|XP_001213274.1|	conserved hypothetical protein	*Aspergillus terreus NIH2624*	1e-04
FE527179	ref|XP_001242914.1|	hypothetical protein CIMG_06810	*Coccidioides immitis RS*	3e-08
FE527180	-	No significant similarity	-	-

FE527181	-	No significant similarity	-	-
FE527182	-	No significant similarity	-	-
FE527183	-	No significant similarity	-	-
FE527184	ref|XP_001247341.1|	hypothetical protein CIMG_01112	*Coccidioides immitis RS*	2e-23
FE527185	ref|XP_001266126.1|	oxidosqualene:lanosterol cyclase	*Neosartorya fischeri NRRL 181*	2e-10
FE527186	ref|YP_704246.1|	possible dipeptidase	*Rhodococcus sp. RHA1*	2e-13
FE527187	ref|XP_753972.1|	glutathione synthetase, putative	*Aspergillus fumigatus Af293*	5e-12
FE527188	gb|AAM54368.1|	elongation factor 1-alpha	*Trichophyton rubrum*	2e-64
FE527189	ref|XP_001240587.1|	hypothetical protein CIMG_07750	*Coccidioides immitis RS*	1e-04
FE527190	ref|XP_001247341.1|	hypothetical protein CIMG_01112	*Coccidioides immitis RS*	3e-19
FE527191	ref|XP_001247341.1|	hypothetical protein CIMG_01112	*Coccidioides immitis RS*	3e-16
FE527192	ref|XP_001544403.1|	Cox19p	*Ajellomyces capsulatus NAm1*	1e-11
FE527193	-	No significant similarity	-	-
FE527194	ref|XP_001540148.1|	conserved hypothetical protein	*Ajellomyces capsulatus NAm1*	1e-18
FE527195	-	No significant similarity	-	-
FE527196	ref|XP_001241535.1|	hypothetical protein CIMG_08698	*Coccidioides immitis RS*	4e-06
FE527197	-	No significant similarity	-	-
FE527198	-	No significant similarity	-	-
FE527199	gb|ABF82266.1|	heat shock protein 30	*Penicillium marneffei*	2e-06
FE527200	-	No significant similarity	-	-
FE527201	ref|XP_001247341.1|	hypothetical protein CIMG_01112	*Coccidioides immitis RS*	4e-23
FE527202	-	No significant similarity	-	-
FE527203	ref|XP_750370.1|	N-acetylglucosamine-phosphate mutase	*Aspergillus fumigatus Af293*	1e-15
FE527204	-	No significant similarity	-	-
FE527205	ref|XP_001257807.1|	Rho GTPase Rho1	*Neosartorya fischeri NRRL 181*	3e-81
FE527206	ref|XP_750999.1|	DUF domain protein	*Aspergillus fumigatus Af293*	4e-05
FE527207	ref|XP_001246171.1|	hypothetical protein CIMG_05612	*Coccidioides immitis RS*	2e-08
FE527208	-	No significant similarity	-	-
FE527209	ref|XP_001542733.1|	ATP-binding cassette sub-family F member 2	*Ajellomyces capsulatus NAm1*	7e-43
FE527210	-	No significant similarity	-	-

FE527211	-	No significant similarity	-	-
FE527212	-	No significant similarity	-	-
FE527213	-	No significant similarity	-	-
FE527214	-	No significant similarity	-	-
FE527215	-	No significant similarity	-	-
FE527216	ref|XP_001247341.1|	hypothetical protein CIMG_01112	*Coccidioides immitis RS*	6e-24
FE527217	ref|ZP_01504263.1|	transcriptional regulator, AraC family	*Burkholderia phymatum STM815*	6e-08
FE527218	ref|XP_001264609.1|	CORD and CS domain protein	*Neosartorya fischeri NRRL 181*	5e-05
FE527219	ref|XP_362903.2|	c-14 sterol reductase	*Magnaporthe grisea 70-15*	9e-41
FE527220	ref|XP_001276316.1|	actin interacting protein 2	*Aspergillus clavatus NRRL 1*	2e-53
FE527221	-	No significant similarity	-	-
FE527222	-	No significant similarity	-	-
FE527223	dbj|BAE65103.1|	unnamed protein product	*Aspergillus oryzae*	1e-08
FE527224	-	No significant similarity	-	-
FE527225	ref|XP_001258414.1|	40S ribosomal protein S29, putative	*Neosartorya fischeri NRRL 181*	6e-10
FE527226	-	No significant similarity	-	-
FE527227	gb|EEQ28484.1|	iron sulfur cluster assembly protein 1, mitochondrial precursor	*Microsporum canis CBS113480*	1e-44
FE527228	ref|XP_001238954.1|	cytochrome c1,	*Coccidioides immitis RS*	1e-44
FE527229	-	No significant similarity	-	-
FE527230	ref|XP_001543311.1|	mitochondrial import inner membrane translocase subunit tim22	*Ajellomyces capsulatus NAm1*	9e-25
FE527231	ref|XP_749294.1|	non-classical export protein Nce102, putative	*Aspergillus fumigatus Af293*	3e-11
FE527232	-	No significant similarity	-	-
FE527233	gb|ABG67901.1|	putative phospholipase	*Trichophyton rubrum*	9e-12
FE527234	ref|XP_001217348.1|	lipoic acid synthetase, mitochondrial precursor	*Aspergillus terreus NIH2624*	1e-46
FE527235	-	No significant similarity	-	-
FE527236	ref|XP_001255645.1|	PREDICTED: similar to Keratin associated protein 10-4	*Bos taurus*	1e-07
FE527237	-	No significant similarity	-	-
FE527238	-	No significant similarity	-	-
FE527239	ref|XP_001247341.1|	hypothetical protein CIMG_01112	*Coccidioides immitis RS*	1e-41

FE527240	ref|XP_001542349.1|	nuclear protein SNF4	*Ajellomyces capsulatus NAm1*	1e-20
FE527241	sp|Q0CA25|FYV10_ASPTN	Protein fyv10	*Aspergillus terreus NIH2624*	6e-31
FE527242	-	No significant similarity	-	-
FE527243	-	No significant similarity	-	-
FE527244	gb|AAS45677.1|	subtilisin-like protease SUB5	*Trichophyton verrucosum*	1e-43
FE527245	ref|XP_001544767.1|	coatomer beta subunit	*Ajellomyces capsulatus NAm1*	1e-16
FE527246	ref|XP_001544767.1|	coatomer beta subunit	*Ajellomyces capsulatus NAm1*	2e-31
FE527247	ref|NP_487763.1|	cation-transporting P-type ATPase	*Nostoc sp. PCC 7120*	7e-04
FE527248	ref|XP_001268985.1|	conserved hypothetical protein	*Aspergillus clavatus NRRL 1*	9e-12
FE527249	gb|AAO47089.1|	mannitol-1-phosphate dehydrogenase	*Paracoccidioides brasiliensis*	1e-42
FE527250	-	No significant similarity	-	-
FE527251	dbj|BAE65103.1|	unnamed protein product	*Aspergillus oryzae*	5e-08
FE527252	ref|XP_001273062.1|	non-classical export protein Nce2, putative	*Aspergillus clavatus NRRL 1*	4e-10
FE527253	ref|XP_001248046.1|	casein kinase I homolog	*Coccidioides immitis RS*	6e-35
FE527254	-	No significant similarity	-	-
FE527255	-	No significant similarity	-	-
FE527256	ref|XP_001242709.1|	hypothetical protein CIMG_06605	*Coccidioides immitis RS*	5e-04
FE527257	ref|XP_001542533.1|	40S ribosomal protein S15	*Ajellomyces capsulatus NAm1*	4e-25
FE527258	ref|XP_001538877.1|	conserved hypothetical protein	*Ajellomyces capsulatus NAm1*	1e-17
FE527259	ref|XP_001212080.1|	enolase	*Aspergillus terreus NIH2624*	4e-28
FE527260	-	No significant similarity	-	-
FE527261	ref|XP_001543567.1|	lanosterol synthase	*Ajellomyces capsulatus NAm1*	4e-34
FE527262	-	No significant similarity	-	-
FE527263	ref|XP_001266272.1|	histone acetylase complex subunit Paf400, putative	*Neosartorya fischeri NRRL 181*	1e-43
FE527264	ref|XP_001247341.1|	hypothetical protein CIMG_01112	*Coccidioides immitis RS*	3e-24

FE527265	-	No significant similarity	-	-
FE527266	ref|XP_001261041.1|	calcium dependent mitochondrial carrier protein, putative	*Neosartorya fischeri NRRL 181*	2e-25
FE527267	ref|XP_001274394.1|	nucleoside diphosphatase (Ynd1), putative	*Aspergillus clavatus NRRL 1*	6e-10
FE527268	ref|NP_830817.1|	Oxalate decarboxylase	*Bacillus cereus ATCC 14579*	3e-20
FE527269	gb|EAA29158.2|	predicted protein	*Neurospora crassa OR74A*	1e-14
FE527270	ref|XP_001247341.1|	hypothetical protein CIMG_01112	*Coccidioides immitis RS*	1e-22
FE527271	gb|ABB96277.1|	hesp-767	*Melampsora lini*	2e-06
FE527272	ref|XP_001263556.1|	Coatomer subunit alpha, putative	*Neosartorya fischeri NRRL 181*	2e-07
FE527273	emb|CAC28076.1|	glucoamylase	*Talaromyces emersonii*	2e-29
FE527274	-	No significant similarity	-	-
FE527275	ref|XP_001275305.1|	actin cytoskeleton protein (VIP1), putative	*Aspergillus clavatus NRRL 1*	8e-21
FE527276	-	No significant similarity	-	-
FE527277	-	No significant similarity	-	-
FE527278	-	No significant similarity	-	-
FE527279	-	No significant similarity	-	-
FE527280	ref|XP_001247341.1|	hypothetical protein CIMG_01112	*Coccidioides immitis RS*	9e-22
FE527281	-	No significant similarity	-	-
FE527282	dbj|BAF48663.1|	leucine-rich repeat/extensin	*Nicotiana plumbaginifolia*	1e-04
FE527283	ref|ZP_01617781.1|	glutathione-dependent formaldehyde-activating, GFA	*marine gamma proteobacterium HTCC2143*	8e-05
FE527284	ref|XP_001263728.1|	phosphopantothenate-cysteine ligase, putative	*Neosartorya fischeri NRRL 181*	3e-19
FE527285	-	No significant similarity	-	-
FE527286	ref|XP_001247341.1|	hypothetical protein CIMG_01112	*Coccidioides immitis RS*	5e-24
FE527287	-	No significant similarity	-	-
FE527288	-	No significant similarity	-	-
FE527289	ref|XP_001262431.1|	phthalate transporter, putative	*Neosartorya fischeri NRRL 181*	3e-22
FE527290	gb|ABY21304.1|	thioredoxin TrxA	*Trichophyton mentagrophytes*	9e-41

FE527291	ref|XP_752593.1|	mitochondrial hypoxia responsive domain protein	*Aspergillus fumigatus Af293*	1e-24
FE527292	ref|XP_001263032.1|	HORMA domain protein	*Neosartorya fischeri NRRL 181*	3e-18
FE527293	-	No significant similarity	-	-
FE527294	ref|XP_001247341.1|	hypothetical protein CIMG_01112	*Coccidioides immitis RS*	4e-23
FE527295	-	No significant similarity	-	-
FE527296	-	No significant similarity	-	-
FE527297	-	No significant similarity	-	-
FE527298	ref|XP_754442.2|	galactose-proton symport, putative	*Aspergillus fumigatus Af293*	7e-04
FE527299	ref|XP_001262893.1|	DNA-directed RNA polymerases N/8 kDa subunit superfamily	*Neosartorya fischeri NRRL 181*	4e-35
FE527300	-	No significant similarity	-	-
FE527301	ref|XP_750553.1|	endoplasmic reticulum DnaJ domain protein Erj5, putative	*Aspergillus fumigatus Af293*	9e-21
FE527302	ref|NP_730262.2|	CG13731-PA	*Drosophila melanogaster*	5e-05
FE527303	-	No significant similarity	-	-
FE527304	ref|XP_001227059.1|	hypothetical protein CHGG_09132	*Chaetomium globosum CBS 148.51*	4e-34
FE527305	gb|AAN87885.1|	NADH-ubiquinone oxidoreductase	*Paracoccidioides brasiliensis*	3e-25
FE527306	ref|XP_001264534.1|	mitochondrial hypoxia responsive domain protein	*Neosartorya fischeri NRRL 181*	1e-16
FE527307	-	No significant similarity	-	-
FE527308	-	No significant similarity	-	-
FE527309	ref|XP_001214203.1|	conserved hypothetical protein	*Aspergillus terreus NIH2624*	6e-25
FE527310	-	No significant similarity	-	-
FE527311	ref|XP_001213878.1|	homoserine dehydrogenase	*Aspergillus terreus NIH2624*	8e-52
FE527312	ref|XP_748143.1|	BYS1 domain protein, putative	*Aspergillus fumigatus Af293*	2e-35
FE527313	dbj|BAD90767.1|	histone 3	*Conocephalum supradecompositum*	2e-22
FE527314	ref|XP_001247458.1|	78 kDa glucose-regulated protein homolog precursor	*Coccidioides immitis RS*	3e-73
FE527315	ref|XP_001541705.1|	hydroxymethylglutaryl-CoA lyase, mitochondrial precursor	*Ajellomyces capsulatus*	5e-50

			NAm1	
FE527316	-	No significant similarity	-	-
FE527317	-	No significant similarity	-	-
FE527318	ref|XP_001261075.1|	C-x8-C-x5-C-x3-H type zinc finger protein	*Neosartorya fischeri NRRL 181*	7e-41
FE527319	ref|XP_001268941.1|	methyltransferase small domain protein	*Aspergillus clavatus NRRL 1*	2e-23
FE527320	-	No significant similarity	-	-
FE527321	-	No significant similarity	-	-
FE527322	ref|XP_001247341.1|	hypothetical protein CIMG_01112	*Coccidioides immitis RS*	3e-24
FE527323	sp|Q0CMM5|DBP4_ASPTN	ATP-dependent RNA helicase dbp4	*Aspergillus terreus NIH2624*	4e-31
FE527324	dbj|BAD90801.1|	histone 3	*Conocephalum conicum*	6e-38
FE527325	-	No significant similarity	-	-
FE527326	ref|XP_001266180.1|	thioredoxin reductase, putative	*Neosartorya fischeri NRRL 181*	4e-24
FE527327	-	No significant similarity	-	-
FE527328	ref|XP_748143.1|	BYS1 domain protein, putative	*Aspergillus fumigatus Af293*	8e-15
FE527329	ref|XP_001672380.1|	Hypothetical protein CBG01536	*Caenorhabditis briggsae*	5e-21
FE527330	-	No significant similarity	-	-
FE527331	-	No significant similarity	-	-
FE527332	dbj|BAD90801.1|	histone 3	*Conocephalum conicum*	2e-42
FE527333	ref|XP_001244171.1|	predicted protein	*Coccidioides immitis RS*	2e-19
FE527334	ref|XP_001244171.1|	predicted protein	*Coccidioides immitis RS*	4e-05
FE527335	ref|XP_001270181.1|	sodium P-type ATPase, putative	*Aspergillus clavatus NRRL 1*	9e-20
FE527336	ref|XP_001543624.1|	NADH-ubiquinone oxidoreductase 9.5 kDa subunit	*Ajellomyces capsulatus NAm1*	2e-13

Sequences with similarity in the nonredundant NCBI database (1e-3) using BLASTx.
