# Supplementary material for: Transcriptional profiling reveals the expression of novel genes in response to various stimuli in the human dermatophyte Trichophyton rubrum
Source: BMC Microbiol. 2010 Feb 8;10:39. doi: 10.1186/1471-2180-10-39 (PMC2831883; doi:10.1186/1471-2180-10-39)
Supplement: Additional file 2 — T. rubrum unigenes database. The data show the complete list of unigenes that are differentially expressed in T. rubrum under each experimental condition, the novel T. rubrum genes (highlighted) and their MIPS categorization. [file 1471-2180-10-39-S2.PDF]

Additional file 2. *T. rubrum* unigenes database

ID	Accession no. of one EST	Libraries										Sequence ID	Gene product	Organism	E- value	MIPS
		1	2	3	4	5	6	7	8	9	10					
Contig001	FE527286	2	0	0	0	0	0	0	0	0	3	ref|XP_001247341.1|	hypothetical protein CIMG_01112	*Coccidioides immitis RS*	5e-24	99
Contig002	FE524614	2	0	0	0	0	0	0	0	0	0	-	No significant similarity	-	-	-
Contig003	FE524620	3	0	0	0	0	0	0	0	0	0	sp|P10255|CYPH_NEUCR	Peptidyl-prolyl cis-trans isomerase, mitochondrial precursor (PPIase) (Rotamase) (Cyclophilin) (Cyclosporin A-binding protein) (CPH)	*Neurospora crassa*	3e-17	14, 16
Contig004	FE524623	2	0	0	0	0	0	0	0	0	0	-	No significant similarity	-	-	-
Contig005	FE525448	3	0	0	0	0	0	0	0	0	0	ref|XP_001267336.1|	outer mitochondrial membrane protein porin	*Neosartorya fischeri NRRL 181*	5e-11	20
Contig006	FE525097	22	0	1	0	0	0	0	0	0	0	-	No significant similarity	-	-	-
Contig007	FE525002	8	0	1	0	0	0	1	0	1	0	dbj|BAA10929.1|	cytochrome P450 like_TBP	*Nicotiana tabacum*	4e-25	32
Contig008	FE526467	1	0	0	0	0	2	44	0	0	1	gb|AAR02424.1|	subtilisin-like protease SUB5	*Trichophyton rubrum*	1e-40	14, 32, 43
Contig009	FE524662	2	0	0	0	0	0	0	0	0	0	sp|P0C2C8|ATP7_ASPTN	ATP synthase D chain, mitochondrial precursor	*Aspergillus terreus NIH2624*	1e-55	2, 20
Contig010	FE524664	2	0	0	0	0	0	0	0	0	0	ref|XP_001211604.1|	predicted protein	*Aspergillus terreus NIH2624*	1e-05	99
Contig011	FE524684	1	0	0	0	0	0	0	0	1	0	ref|XP_001211720.1|	60S ribosomal protein L12	*Aspergillus terreus NIH2624*	6e-14	12
Contig012	FE525285	5	0	0	0	0	0	0	0	0	0	gb|ABH10645.1|	ATP synthase beta chain	*Coccidioides posadasii*	2e-15	2, 16, 20
Contig013	FE524917	14	0	0	0	1	0	0	0	0	0	-	No significant similarity	-	-	-
Contig014	FE524741	3	0	0	0	0	0	0	0	0	0	ref|XP_001248928.1|	elongation factor 1-gamma	*Coccidioides immitis RS*	8e-32	12, 16
Contig015	FE524752	2	0	0	0	0	0	0	0	0	0	dbj|BAE64245.1|	unnamed protein product	*Aspergillus oryzae*	5e-22	99
Contig016	FE524754	1	0	0	0	0	0	0	0	0	2	dbj|BAE65103.1|	unnamed protein product	*Aspergillus oryzae*	2e-07	99
Contig017	FE525484	2	0	0	0	0	0	0	0	0	0	ref|NP_941181.1|	chloramphenicol acetyltransferase	*Serratia marcescens*	4e-05	1
Contig018	FE525108	4	0	0	0	0	0	0	0	0	0	-	No significant similarity	-	-	-
Contig019	FE524769	8	0	0	0	0	0	0	0	0	0	ref|XP_001246797.1|	polyubiquitin	*Coccidioides immitis RS*	8e-93	14, 32
Contig020	FE527085	2	0	0	0	0	0	0	0	5	0	-	No significant similarity	-	-	-
Contig021	FE525235	2	0	0	0	0	0	0	0	0	0	-	No significant similarity	-	-	-
Contig022	FE524781	2	0	0	0	0	0	0	0	0	0	-	No significant similarity	-	-	-

Contig023	FE524791	2	0	0	0	0	0	0	0	0	0	-	No significant similarity	-	-	-
Contig024	FE525062	10	0	0	0	0	0	0	0	0	0	-	No significant similarity	-	-	-
Contig025	FE525280	2	0	0	0	0	0	0	0	0	0	-	No significant similarity	-	-	-
Contig026	FE524806	2	0	0	0	0	0	0	0	0	0	ref|XP_750061.1|	ribosomal protein L29/heparin/heparan sulfate interacting protein	*Aspergillus fumigatus Af293*	3e-20	12
Contig027	FE524824	2	0	0	0	0	0	0	0	0	0	ref|XP_750146.1|	Rhodanese domain protein	*Aspergillus fumigatus Af293*	5e-37	99
Contig028	FE524678	2	0	0	0	0	0	0	0	0	0	-	No significant similarity	-	-	-
Contig029	FE524750	2	0	0	0	0	0	0	0	0	0	-	No significant similarity	-	-	-
Contig030	FE527302	5	0	0	0	0	0	0	0	0	2	ref|NP_730262.2|	CG13731-PA	*Drosophila melanogaster*	5e-05	99
Contig031	FE524856	1	0	0	0	0	0	0	0	2	0	-	No significant similarity	-	-	-
Contig032	FE524863	2	0	0	0	0	0	0	0	0	0	ref|XP_001247280.1|	alternative oxidase, mitochondrial precursor	*Coccidioides immitis RS*	4e-11	2
Contig033	FE525567	4	0	0	0	0	0	0	0	0	0	-	No significant similarity	-	-	-
Contig034	FE525114	2	0	0	0	0	0	0	0	0	0	ref|XP_001243230.1|	40S ribosomal protein S23	*Coccidioides immitis RS*	1e-19	12
Contig035	FE524957	2	0	0	0	0	0	0	0	0	0	ref|XP_001257681.1|	actin	*Neosartorya fischeri NRRL 181*	3e-42	10, 14, 20
Contig036	FE524976	3	0	0	0	0	0	0	0	0	0	ref|XP_001274835.1|	60S ribosomal protein L7	*Aspergillus clavatus NRRL 1*	3e-49	12
Contig037	FE525271	2	0	0	0	0	0	0	0	0	0	-	No significant similarity	-	-	-
Contig038	FE524986	2	0	0	0	0	0	0	0	0	0	ref|XP_001265902.1|	alkaline serine protease	*Neosartorya fischeri NRRL 181*	2e-12	1, 40, 43
Contig039	FE524999	4	0	0	0	0	0	0	0	0	0	gb|EEQ29198.1|	glyceraldehyde-3-phosphate dehydrogenase	*Microsporum canis CBS113480*	8e-44	1, 16, 32
Contig040	FE524740	6	0	0	0	0	0	0	0	0	0	-	No significant similarity	-	-	-
Contig041	FE525018	2	0	0	0	0	0	0	0	0	0	ref|XP_001248361.1|	histone H2A	*Coccidioides immitis RS*	6e-04	10, 11, 16
Contig042	FE527184	4	0	0	0	0	0	0	0	0	9	ref|XP_001247341.1|	hypothetical protein CIMG_01112	*Coccidioides immitis RS*	2e-23	99
Contig043	FE524808	4	0	0	0	0	0	0	0	0	0	gb|AAC13689.1|	ubiquitin fusion protein	*Magnaporthe grisea*	3e-47	11, 14, 16
Contig044	FE525085	3	0	1	0	0	0	0	0	0	0	-	No significant similarity	-	-	-
Contig045	FE525120	2	0	0	0	0	0	0	0	0	0	-	No significant similarity	-	-	-
Contig046	FE525098	2	0	0	0	0	0	1	0	0	0	-	No significant similarity	-	-	-
Contig047	FE525117	90	0	0	0	0	0	1	0	0	0	ref|XP_001241788.1|	hypothetical protein CIMG_05684	*Coccidioides immitis RS*	6e-11	99

Contig048	FE525123	2	0	0	0	0	0	0	0	0	0	-	No significant similarity	-	-	-
Contig049	FE525497	2	0	0	0	0	0	0	0	0	0	gb|AAL31950.1|	CDH1-D	*Gallus gallus*	3e-11	99
Contig050	FE525177	2	0	0	0	0	0	0	0	0	0	-	No significant similarity	-	-	-
Contig051	FE524736	2	0	0	0	0	0	0	0	0	0	-	No significant similarity	-	-	-
Contig052	FE525116	2	0	0	0	0	0	0	0	1	0	ref|XP_001274770.1|	60S ribosomal protein L13	*Aspergillus clavatus NRRL 1*	4e-12	12
Contig053	FE524797	2	0	0	0	0	0	0	0	2	3	gb|AAL76232.1|AF408429_1	metallothionein	*Microsporum canis*	2e-04	1, 10, 11
Contig054	FE525246	2	0	0	0	0	0	0	0	0	0	sp|P56205|CYC_ASPNG	Cytochrome c	*Aspergillus niger*	1e-11	1, 20, 32
Contig055	FE525110	4	0	0	0	0	0	0	0	0	0	ref|XP_747995.1|	nucleoside diphosphate kinase	*Aspergillus fumigatus Af293*	2e-32	1, 16
Contig056	FE525256	2	0	0	0	0	0	0	0	0	0	-	No significant similarity	-	-	-
Contig057	FE525277	2	0	0	0	0	0	0	0	0	0	emb|CAD56870.1|	BclA protein	*Bacillus anthracis*	2e-06	99
Contig058	FE525307	3	0	0	0	0	0	0	0	0	0	-	No significant similarity	-	-	-
Contig059	FE524701	11	0	0	0	0	0	0	0	0	0	-	No significant similarity	-	-	-
Contig060	FE525417	5	0	0	0	0	0	0	0	0	0	-	No significant similarity	-	-	-
Contig061	FE524663	3	0	0	0	0	0	0	0	0	0	ref|XP_001268300.1|	chaperone/heat shock protein Hsp12, putative	*Aspergillus clavatus NRRL 1*	5e-20	16, 32
Contig062	FE525371	2	0	0	0	0	0	0	0	0	0	dbj|BAE73006.1|	hypothetical protein	*Macaca fascicularis*	1e-06	99
Contig063	FE525121	27	0	0	6	13	0	0	0	1	0	-	No significant similarity	-	-	-
Contig064	FE525397	2	0	0	0	0	0	0	0	0	0	-	No significant similarity	-	-	-
Contig065	FE524673	3	0	0	0	0	0	0	0	0	0	-	No significant similarity	-	-	-
Contig066	FE525430	2	0	0	0	0	0	0	0	0	0	-	No significant similarity	-	-	-
Contig067	FE527271	1	0	0	0	1	0	0	0	0	1	gb|ABB96277.1|	hesp-767	*Melampsora lini*	2e-06	99
Contig068	FE524919	7	0	0	0	0	0	0	0	0	0	ref|XP_001268551.1|	conidial hydrophobin RodB	*Aspergillus clavatus NRRL 1*	1e-04	43
Contig069	FE524841	2	0	0	0	0	0	0	0	0	0	ref|XP_001210407.1|	elongation factor 1-beta	*Aspergillus terreus NIH2624*	4e-07	12, 16, 18
Contig070	FE525554	2	0	0	0	0	0	0	0	0	0	-	No significant similarity	-	-	-
Contig071	FE525475	1	0	0	1	0	0	0	0	0	0	-	No significant similarity	-	-	-
Contig072	FE525512	1	0	0	0	0	0	0	0	1	0	ref|XP_001267282.1|	60S ribosomal protein L36	*Neosartorya fischeri NRRL 181*	7e-26	12, 16
Contig073	FE525572	2	0	0	0	0	0	0	0	0	0	ref|XP_001261472.1|	40S ribosomal protein S13	*Neosartorya fischeri NRRL 181*	2e-10	12, 16
Contig074	FE526557	0	0	0	0	0	0	0	7	0	0	ref|XP_001244184.1|	hypothetical protein CIMG_03625	*Coccidioides immitis RS*	9e-49	99
Contig075	FE526556	0	1	0	0	0	9	3	5	0	0	ref|XP_754782.1|	hypothetical protein Afu3g08320	*Aspergillus fumigatus Af293*	2e-05	99
Contig076	FE526560	0	0	0	0	0	0	0	2	0	0	-	No significant similarity	-	-	-

Contig077	FE526214	0	5	0	0	0	2	7	3	0	0	ref|XP_001242594.1|	hypothetical protein CIMG_06490	*Coccidioides immitis RS*	1e-06	99
Contig078	FE526224	0	2	0	0	0	8	2	4	0	0	gb|EEQ27465.1|	copper resistance-associated P-type ATPase, putative	*Microsporum canis CBS 113480*	8e-24	20
Contig079	FE526195	0	0	0	0	0	5	8	8	0	0	ref|XP_001269482.1|	Leucine Rich Repeat domain protein	*Aspergillus clavatus NRRL 1*	8e-04	98
Contig080	FE526593	0	2	0	0	0	15	1	12	0	0	gb|AAG24792.1|AF264028_2	pol protein	*Glomerella cingulata*	2e-28	38
Contig081	FE526568	0	2	0	0	0	31	9	34	0	0	gb|EEQ33141.1|	NIMA interactive protein	*Microsporum canis CBS 113480*	2e-23	10
Contig082	FE525630	0	5	0	0	0	0	0	1	0	0	-	No significant similarity	-	-	-
Contig083	FE526670	0	0	0	0	0	0	0	4	0	0	-	No significant similarity	-	-	-
Contig084	FE526635	0	0	0	0	0	0	0	4	0	0	ref|YP_371113.1|	Aldehyde dehydrogenase	*Burkholderia sp. 383*	3e-75	1, 2, 20, 32
Contig085	FE526598	0	4	0	0	0	7	9	6	0	0	gb|AAG01549.3|AF291822_1	multidrug resistance protein MDR	*Trichophyton rubrum*	2e-14	20, 32
Contig086	FE526671	0	1	0	0	0	0	0	16	0	0	ref|XP_001226392.1|	hypothetical protein CHGG_08465	*Chaetomium globosum CBS 148.51*	4e-10	99
Contig087	FE526730	0	0	0	0	0	0	0	4	0	0	ref|YP_443455.1|	secretion protein, putative	*Burkholderia thailandensis E264*	4e-04	20
Contig088	FE526749	0	0	0	0	0	0	0	10	0	0	ref|XP_001272385.1|	FYVE zinc finger protein	*Aspergillus clavatus NRRL 1*	3e-04	14, 16, 20
Contig089	FE526711	0	0	0	0	0	0	0	2	0	0	-	No significant similarity	-	-	-
Contig090	FE526689	0	0	0	0	0	0	0	7	0	0	-	No significant similarity	-	-	-
Contig091	FE526715	0	0	0	0	0	0	0	3	0	0	-	No significant similarity	-	-	-
Contig092	FE526724	0	0	0	0	0	0	0	3	0	0	ref|XP_001263899.1|	60S ribosomal protein L20, putative	*Neosartorya fischeri NRRL 181*	5e-11	12
Contig093	FE526742	0	0	0	0	0	0	0	2	0	0	-	No significant similarity	-	-	-
Contig094	FE526722	0	0	0	0	0	0	0	5	0	0	-	No significant similarity	-	-	-
Contig095	FE526723	0	0	1	0	0	0	0	2	0	0	-	No significant similarity	-	-	-
Contig096	FE526737	0	0	0	0	0	0	0	5	0	0	-	No significant similarity	-	-	-
Contig097	FE526705	0	0	0	0	0	0	0	4	0	0	-	No significant similarity	-	-	-
Contig098	FE525701	0	0	2	0	0	0	0	0	0	0	-	No significant similarity	-	-	-
Contig099	FE525086	1	0	1	0	0	0	0	0	0	0	ref|XP_001264756.1|	adenosylhomocysteinase	*Neosartorya fischeri NRRL 181*	1e-60	1, 16
Contig100	FE525682	0	0	2	0	0	0	0	0	0	0	ref|XP_001215759.1|	woronin body major protein	*Aspergillus terreus NIH2624*	2e-26	16, 43
Contig101	FE525724	1	0	2	1	0	0	0	0	0	0	-	No significant similarity	-	-	-
Contig102	FE525583	0	1	1	0	0	0	0	0	0	0	ref|XP_001246822.1|	hypothetical protein CIMG_00593	*Coccidioides immitis RS*	3e-40	99
Contig103	FE525736	1	0	1	0	0	0	0	0	0	0	-	No significant similarity	-	-	-

Contig104	FE525737	1	0	1	0	0	0	0	0	0	0	-	No significant similarity	-	-	-
Contig105	FE525508	1	0	1	0	0	0	0	0	0	0	-	No significant similarity	-	-	-
Contig106	FE525745	0	0	2	0	0	0	0	0	0	0	ref|XP_750031.1|	mRNA cleavage and polyadenylation specificity factor complex subunit, putative	*Aspergillus fumigatus Af293*	1e-06	11, 16
Contig107	FE525746	0	0	3	0	0	0	0	0	0	0	gb|AAB05810.1|	super cysteine rich protein; SCRP	*Homo sapiens*	4e-14	11, 16
Contig108	FE525757	0	0	1	0	0	0	0	0	1	0	ref|XP_001269377.1|	DUF814 domain protein	*Aspergillus clavatus NRRL 1*	2e-05	99
Contig109	FE525774	4	0	11	0	2	0	0	0	0	0	dbj|BAB12047.1|	alpha-crystallin-related protein	*Arthroderma benhamiae*	2e-19	99
Contig110	FE526055	0	0	1	0	3	0	0	0	1	0	ref|XP_001247978.1|	30 kDa heat shock protein	*Coccidioides immitis RS*	7e-04	32, 34
Contig111	FE525778	0	0	3	0	0	0	0	0	0	0	-	No significant similarity	-	-	-
Contig112	FE525127	2	0	0	0	0	0	0	0	3	0	ref|XP_001271667.1|	60S ribosomal protein L11	*Aspergillus clavatus NRRL 1*	3e-67	12
Contig113	FE526813	0	0	0	0	0	0	0	0	2	0	ref|XP_001248510.1|	alanyl-tRNA synthetase, cytoplasmic	*Coccidioides immitis RS*	1e-17	12, 16
Contig114	FE526827	0	0	0	0	0	0	0	0	2	0	gb|EAL91735.2|	integral membrane protein	*Aspergillus fumigatus Af293*	4e-23	20, 32
Contig115	FE526791	0	0	0	0	0	0	0	0	4	0	ref|XP_001267237.1|	60S ribosomal protein L38, putative	*Neosartorya fischeri NRRL 181*	5e-14	12
Contig116	FE527151	0	0	0	0	0	0	0	0	2	1	ref|XP_752484.2|	eukaryotic translation elongation factor 1 subunit Eef1-beta, putative	*Aspergillus fumigatus Af293*	5e-13	12, 16, 18
Contig117	FE526856	0	0	0	2	0	0	0	0	1	0	gb|ABN50029.1|	putative senescence-associated protein	*Trichosanthes dioica*	2e-05	40
Contig118	FE526819	0	0	0	0	0	0	0	0	2	0	-	No significant similarity	-	-	-
Contig119	FE526878	0	0	0	0	0	0	0	0	2	0	ref|XP_001246583.1|	succinate-semialdehyde dehydrogenase	*Coccidioides immitis RS*	3e-27	1, 2, 16, 32
Contig120	FE526893	0	0	0	0	0	0	0	0	2	0	-	No significant similarity	-	-	-
Contig121	FE526894	0	0	0	0	0	0	0	0	1	1	-	No significant similarity	-	-	-
Contig122	FE526901	0	0	0	0	0	0	0	0	2	0	ref|XP_661668.1|	ADP/ATP carrier protein	*Aspergillus nidulans FGSC A4*	1e-40	1, 16, 20
Contig123	FE526892	0	0	0	0	0	0	0	0	2	0	-	No significant similarity	-	-	-
Contig124	FE526907	0	0	0	0	0	0	0	0	7	0	ref|XP_001273971.1|	60S ribosomal protein L8, putative	*Aspergillus clavatus NRRL 1*	2e-12	12
Contig125	FE526912	0	0	0	0	0	0	0	0	3	0	-	No significant similarity	-	-	-
Contig126	FE526925	0	0	0	3	0	0	0	0	1	0	ref|XP_001258439.1|	60S ribosomal protein L7a	*Neosartorya fischeri NRRL 181*	1e-08	12
Contig127	FE525089	1	0	0	0	0	0	0	0	1	0	-	No significant similarity	-	-	-
Contig128	FE526931	0	0	0	0	0	0	0	0	2	0	ref|XP_001241489.1|	hypothetical protein CIMG_08652	*Coccidioides immitis*	3e-23	99

														RS		
Contig129	FE524997	2	0	0	0	0	0	0	0	4	0	ref|XP_001248308.1|	60S ribosomal protein L28	*Coccidioides immitis RS*	2e-44	12
Contig130	FE527147	0	0	0	0	0	0	0	0	1	2	-	No significant similarity	-	-	-
Contig131	FE526918	0	0	0	0	0	0	0	0	2	0	-	No significant similarity	-	-	-
Contig132	FE525078	2	0	0	0	0	0	0	0	1	0	ref|XP_001275347.1|	40S ribosomal protein S9	*Aspergillus clavatus NRRL 1*	8e-41	12
Contig133	FE526948	0	0	0	0	1	0	0	0	3	0	gb|EDJ95143.1|	60S ribosomal protein L7	*Magnaporthe grisea 70-15*	1e-12	12
Contig134	FE526953	0	0	0	0	0	0	0	0	1	1	ref|XP_001273282.1|	mitochondrial ribosomal protein DAP3, putative	*Aspergillus clavatus NRRL 1*	2e-12	12, 42
Contig135	FE525332	1	0	0	0	0	0	0	0	1	0	ref|XP_001244490.1|	ADP,ATP carrier protein	*Coccidioides immitis RS*	4e-22	1, 16, 20
Contig136	FE526976	2	0	0	0	0	0	0	0	1	0	ref|XP_001271126.1|	ubiquitin	*Aspergillus clavatus NRRL 1*	3e-23	14
Contig137	FE526977	0	0	0	0	0	0	0	0	2	0	ref|XP_001266080.1|	seryl-tRNA synthetase	*Neosartorya fischeri NRRL 181*	3e-25	12, 43
Contig138	FE526979	1	0	0	0	0	0	0	0	1	0	emb|CAM37639.1|	proteophosphoglycan ppg1	*Leishmania braziliensis*	1e-23	99
Contig139	FE526836	0	0	0	0	0	0	0	0	2	0	-	No significant similarity	-	-	-
Contig140	FE526989	0	0	0	0	0	0	0	0	3	0	ref|XP_001257403.1|	benzoate 4-monooxygenase cytochrome P450	*Neosartorya fischeri NRRL 181*	1e-13	1
Contig141	FE526992	0	0	0	0	0	0	0	0	2	0	-	No significant similarity	-	-	-
Contig142	FE526217	0	1	0	0	0	2	0	0	1	0	-	No significant similarity	-	-	-
Contig143	FE526913	0	0	0	0	0	0	0	0	2	0	-	No significant similarity	-	-	-
Contig144	FE527006	0	0	0	0	0	0	0	0	2	0	ref|XP_001275968.1|	60S ribosomal protein L37a	*Aspergillus clavatus NRRL 1*	7e-21	12
Contig145	FE527012	0	0	0	0	0	0	0	0	2	0	-	No significant similarity	-	-	-
Contig146	FE527021	0	0	0	0	0	0	0	0	2	0	ref|XP_001266736.1|	MFS monosaccharide transporter, putative	*Neosartorya fischeri NRRL 181*	3e-20	20
Contig147	FE526971	0	0	0	0	0	0	0	0	9	0	-	No significant similarity	-	-	-
Contig148	FE525035	1	0	0	0	0	0	0	0	1	0	ref|XP_001261370.1|	Ribosomal protein S28e	*Neosartorya fischeri NRRL 181*	9e-24	12, 16
Contig149	FE527030	0	0	0	0	0	0	0	0	2	0	-	No significant similarity	-	-	-
Contig150	FE526869	0	0	0	0	0	0	0	0	4	0	ref|XP_753997.1|	60s ribosomal protein yl6	*Aspergillus fumigatus Af293*	2e-17	12
Contig151	FE526867	0	0	0	0	0	0	0	0	6	0	ref|XP_001387342.1|	60S large subunit ribosomal protein	*Pichia stipitis CBS 6054*	4e-04	12
Contig152	FE526871	0	0	0	0	0	0	0	0	6	0	ref|XP_001246620.1|	40S ribosomal protein S4	*Coccidioides immitis RS*	1e-26	12
Contig153	FE527051	0	0	0	0	0	0	0	0	4	0	-	No significant similarity	-	-	-

Contig154	FE527058	0	0	0	0	0	0	0	0	2	0	ref|XP_001265211.1|	Ribosomal L18ae protein family	*Neosartorya fischeri NRRL 181*	2e-15	12, 16
Contig155	FE527106	0	0	0	0	0	0	0	0	3	0	ref|XP_001270945.1|	PDCD2_C domain protein, putative	*Aspergillus clavatus NRRL 1*	1e-08	99
Contig156	FE525037	1	0	0	0	0	0	0	0	1	0	ref|XP_001257579.1|	2-methylcitrate dehydratase, putative	*Neosartorya fischeri NRRL 181*	5e-42	1
Contig157	FE527111	0	0	0	0	0	0	0	0	2	0	-	No significant similarity	-	-	-
Contig158	FE525608	0	3	0	0	0	0	0	0	0	0	gb|EAL84327.2|	short chain oxidoreductase (CsgA), putative	*Aspergillus fumigatus Af293*	7e-04	1
Contig159	FE525581	0	2	0	0	0	0	0	0	0	0	ref|XP_001248139.1|	hypothetical protein CIMG_01910	*Coccidioides immitis RS*	2e-25	99
Contig160	FE525582	0	2	0	0	0	0	0	0	0	0	-	No significant similarity	-	-	-
Contig161	FE525610	0	7	0	0	0	0	0	0	0	0	ref|XP_001268233.1|	ABC transporter, putative	*Aspergillus clavatus NRRL 1*	2e-17	16, 20
Contig162	FE525594	0	7	0	0	0	0	0	0	0	0	ref|XP_001272698.1|	Dopey, N-terminal domain protein	*Aspergillus clavatus NRRL 1*	2e-09	99
Contig163	FE525597	0	2	0	0	0	0	0	0	0	0	ref|XP_001266096.1|	Pumilio-family RNA binding repeat protein	*Neosartorya fischeri NRRL 181*	3e-10	11, 16
Contig164	FE525603	0	2	0	0	0	0	0	0	0	0	-	No significant similarity	-	-	-
Contig165	FE525605	0	3	0	0	0	0	0	0	0	0	gb|EEQ28523.1|	Salicylate hydroxylase (Salicylate 1-monooxygenase)	*Microsporum canis* CBS 113480	6e-43	1
Contig166	FE525611	0	5	0	0	0	0	0	0	0	0	ref|XP_662910.1|	hypothetical protein AN5306.2	*Aspergillus nidulans FGSC A4*	6e-11	99
Contig167	FE525681	0	2	0	0	0	0	0	0	0	0	-	No significant similarity	-	-	-
Contig168	FE525619	0	5	0	0	0	0	0	3	0	0	ref|YP_673529.1|	Glyoxalase/bleomycin resistance protein/dioxygenase	*Mesorhizobium sp. BNC1*	4e-09	1
Contig169	FE525632	0	4	0	0	0	0	3	0	0	0	ref|XP_001211183.1|	DNA polymerase gamma	*Aspergillus terreus NIH2624*	4e-22	10, 16
Contig170	FE525633	0	2	0	0	0	1	0	0	0	0	-	No significant similarity	-	-	-
Contig171	FE525646	0	10	0	0	0	3	0	0	0	0	-	No significant similarity	-	-	-
Contig172	FE525786	0	0	0	2	0	0	0	0	0	0	ref|XP_001269539.1|	DUF887 domain protein	*Aspergillus clavatus NRRL 1*	3e-12	99
Contig173	FE525796	0	0	0	2	0	0	0	0	0	0	gb|AAX33296.1|	heat shock protein 90	*Paracoccidioides brasiliensis*	7e-21	14, 16, 32
Contig174	FE525800	0	0	0	2	0	0	0	0	0	0	-	No significant similarity	-	-	-
Contig175	FE525960	0	0	0	3	0	0	0	0	0	0	ref|XP_001247141.1|	60S ribosomal protein L19	*Coccidioides immitis RS*	6e-11	12
Contig176	FE525803	0	0	0	2	0	0	0	0	0	0	-	No significant similarity	-	-	-
Contig177	FE525804	0	0	0	3	0	0	0	0	0	0	-	No significant similarity	-	-	-
Contig178	FE525805	0	0	0	3	0	0	0	0	0	0	-	No significant similarity	-	-	-

Contig179	FE525808	0	0	0	1	0	0	0	0	3	0	ref|XP_001260419.1|	60S ribosomal protein L10	*Neosartorya fischeri NRRL 181*	7e-39	12
Contig180	FE525809	0	0	0	2	0	0	0	0	0	0	ref|XP_001265142.1|	DNA-directed DNA polymerase theta, putative	*Neosartorya fischeri NRRL 181*	1e-27	10
Contig181	FE525818	0	0	0	3	0	0	0	0	0	0	ref|XP_754663.1|	DNA polymerase epsilon, catalytic subunit A/POL2, putative	*Aspergillus fumigatus Af293*	8e-06	10, 16, 32
Contig182	FE524981	1	0	0	0	1	0	0	0	0	0	ref|XP_747833.1|	60s ribosomal protein L24, putative	*Aspergillus fumigatus Af293*	2e-52	12
Contig183	FE525893	0	0	0	2	1	0	0	0	0	0	ref|XP_001247301.1|	proteasome component	*Coccidioides immitis RS*	7e-24	14, 16, 18, 32
Contig184	FE526079	0	0	0	1	1	0	0	0	0	0	gb|EAL86837.2|	sodium/phosphate symporter, putative	*Aspergillus fumigatus Af293*	6e-12	20
Contig185	FE526009	0	0	0	2	0	0	0	0	0	0	gb|EAL90029.2|	translation initiation factor EF-2 gamma subunit, putative	*Aspergillus fumigatus Af293*	4e-21	12
Contig186	FE525827	0	0	0	4	0	0	0	0	0	0	ref|ZP_00344940.1|	COG0458: Carbamoylphosphate synthase large subunit (split gene in MJ)	*Nostoc punctiforme PCC 73102*	8e-12	1, 16
Contig187	FE525908	0	0	0	3	0	0	0	0	0	0	ref|XP_001264383.1|	C2H2 transcription factor (Rpn4), putative	*Neosartorya fischeri NRRL 181*	1e-35	11
Contig188	FE525835	0	0	0	3	0	0	0	0	0	0	-	No significant similarity	-	-	-
Contig189	FE525839	0	0	0	2	0	0	0	0	1	0	gb|AAB06687.1|	complement fixation antigen gb|AAA96515.1| complement-fixation antigen	*Coccidioides posadasii*	6e-11	99
Contig190	FE525840	0	0	0	3	0	0	0	0	0	0	ref|XP_001258400.1|	anthranilate synthase component I, putative	*Neosartorya fischeri NRRL 181*	4e-05	1
Contig191	FE525824	0	0	0	2	0	0	0	0	0	0	-	No significant similarity	-	-	-
Contig192	FE525918	0	0	0	9	0	0	0	0	0	0	ref|XP_001263576.1|	eukaryotic translation initiation factor 3 subunit 2i, putative	*Neosartorya fischeri NRRL 181*	1e-27	11, 12, 16
Contig193	FE524735	4	0	0	0	1	0	0	0	0	0	-	No significant similarity	-	-	-
Contig194	FE526362	0	0	1	1	2	0	4	0	3	1	ref|XP_001272561.1|	heat shock Hsp30-like protein, putative	*Aspergillus clavatus NRRL 1*	2e-07	16, 32
Contig195	FE526096	0	0	0	0	2	0	0	0	0	0	-	No significant similarity	-	-	-
Contig196	FE526064	13	0	0	11	23	0	0	0	0	0	-	No significant similarity	-	-	-
Contig197	FE525853	0	0	0	2	0	0	0	0	0	0	-	No significant similarity	-	-	-
Contig198	FE525864	0	0	0	2	0	0	0	0	0	0	-	No significant similarity	-	-	-
Contig199	FE525869	0	0	0	4	0	0	0	0	0	0	ref|XP_754941.1|	aminoalcoholphosphotransferase	*Aspergillus fumigatus Af293*	2e-14	1, 20
Contig200	FE525998	0	0	0	3	0	0	0	0	0	0	ref|XP_001209915.1|	conserved hypothetical protein	*Aspergillus terreus NIH2624*	1e-07	99

Contig201	FE525875	0	0	0	2	0	0	0	0	0	0	ref|XP_001209708.1|	conserved hypothetical protein	*Aspergillus terreus NIH2624*	2e-18	99
Contig202	FE525878	0	0	0	2	0	0	0	0	0	0	ref|XP_001269644.1|	N-acetyltransferase (Nat5), putative	*Aspergillus clavatus NRRL 1*	3e-21	1, 14
Contig203	FE525881	0	0	0	2	0	0	0	0	0	0	-	No significant similarity	-	-	-
Contig204	FE525882	0	0	0	2	0	0	0	0	0	0	-	No significant similarity	-	-	-
Contig205	FE525883	0	0	0	2	0	0	0	0	0	0	sp|P00048|CYC_NEUCR	Cytochrome c emb|CAA29050.1| cytochrome c	*Neurospora crassa*	5e-11	2, 16, 20
Contig206	FE525885	0	0	0	2	0	0	0	0	0	0	ref|XP_001246545.1|	phosphatidylethanolamine N-methyltransferase	*Coccidioides immitis RS*	1e-04	1
Contig207	FE525886	0	0	0	2	0	0	0	0	0	0	ref|XP_001275966.1|	MFS multidrug transporter, putative	*Aspergillus clavatus NRRL 1*	3e-31	20, 32
Contig208	FE525889	1	0	0	3	1	0	0	0	0	0	ref|XP_001213531.1|	component PRE3 precursor	*Aspergillus terreus NIH2624*	6e-31	14, 16, 18, 32
Contig209	FE525855	0	0	0	2	0	0	0	0	0	0	ref|XP_001260488.1|	actin cytoskeleton protein (VIP1), putative	*Neosartorya fischeri NRRL 181*	3e-10	14, 42, 43
Contig210	FE525900	0	0	0	2	1	0	0	0	0	0	ref|XP_001266911.1|	acetyl-coenzyme A synthetase FacA	*Neosartorya fischeri NRRL 181*	9e-26	1, 2
Contig211	FE525903	0	0	0	3	1	0	0	0	0	0	ref|XP_001257912.1|	PH domain protein	*Neosartorya fischeri NRRL 181*	2e-10	99
Contig212	FE526814	0	0	0	0	1	0	0	0	1	0	-	No significant similarity	-	-	-
Contig213	FE526105	0	0	0	0	2	0	0	0	0	0	-	No significant similarity	-	-	-
Contig214	FE526117	0	0	0	0	2	0	0	0	0	0	ref|XP_001271793.1|	DEAD/DEAH box helicase, putative	*Aspergillus clavatus NRRL 1*	1e-49	1, 10, 11
Contig215	FE526136	0	0	0	1	1	0	0	0	0	0	-	No significant similarity	-	-	-
Contig216	FE526095	15	0	0	3	21	0	0	0	1	1	-	No significant similarity	-	-	-
Contig217	FE525916	0	0	0	3	1	0	0	0	0	0	ref|XP_755151.1|	NEDD8-like protein (RubA), putative	*Aspergillus fumigatus Af293*	1e-11	11, 12, 14, 16
Contig218	FE525890	0	0	0	2	0	0	0	0	0	0	-	No significant similarity	-	-	-
Contig219	FE525923	0	0	0	1	0	0	0	0	1	0	ref|XP_001214623.1|	eukaryotic translation initiation factor 2 gamma subunit	*Aspergillus terreus NIH2624*	2e-25	12, 16, 18
Contig220	FE525895	0	0	0	3	0	0	0	0	0	0	ref|XP_721980.1|	putative S-adenosyl-L-homocysteine hydrolase	*Candida albicans SC5314*	8e-08	1, 16
Contig221	FE525928	0	0	0	1	1	0	0	0	0	0	-	No significant similarity	-	-	-
Contig222	FE525931	0	0	0	2	0	0	0	0	0	0	-	No significant similarity	-	-	-
Contig223	FE525934	0	0	0	2	1	0	0	0	0	0	-	No significant similarity	-	-	-
Contig224	FE525937	0	0	0	3	0	0	0	0	0	0	-	No significant similarity	-	-	-
Contig225	FE525939	0	0	0	3	0	0	0	0	0	0	ref|XP_001267735.1|	short chain dehydrogenase/reductase family protein	*Aspergillus clavatus NRRL 1*	4e-21	1, 34

Contig226	FE525943	0	0	0	2	0	0	0	0	0	0	ref|XP_001270328.1|	DUF803 domain protein	*Aspergillus clavatus NRRL 1*	2e-29	99
Contig227	FE525944	0	0	0	2	0	0	0	0	0	0	-	No significant similarity	-	-	-
Contig228	FE525948	0	0	0	3	0	0	0	0	0	0	dbj|BAE62495.1|	unnamed protein product	*Aspergillus oryzae*	1e-14	99
Contig229	FE525957	0	0	0	2	0	0	0	0	0	0	-	No significant similarity	-	-	-
Contig230	FE525812	0	0	0	2	0	0	0	0	0	0	ref|XP_682122.1|	eukaryotic peptide chain release factor subunit 1	*Aspergillus nidulans FGSC A4*	1e-70	1, 10, 12
Contig231	FE525813	0	0	0	2	0	0	0	0	0	0	-	No significant similarity	-	-	-
Contig232	FE525964	0	0	0	3	0	0	0	0	0	0	ref|XP_001248599.1|	eukaryotic translation initiation factor 3 subunit 7 homolog	*Coccidioides immitis RS*	5e-21	11, 12, 16
Contig233	FE525967	0	0	0	4	0	0	0	0	0	0	ref|XP_746577.1|	peroxisomal multifunctional beta-oxidation protein (MFP), putative	*Aspergillus fumigatus Af293*	2e-19	99
Contig234	FE525969	0	0	0	1	0	0	0	0	1	0	ref|XP_001268779.1|	BAP31 domain protein, putative	*Aspergillus clavatus NRRL 1*	4e-14	99
Contig235	FE525880	0	0	0	19	0	0	0	0	0	0	dbj|BAA81686.1|	expressed in cucumber hypocotyls	*Cucumis sativus*	1e-04	99
Contig236	FE525991	0	3	0	2	0	0	0	0	0	0	-	No significant similarity	-	-	-
Contig237	FE525851	0	0	0	3	0	0	0	0	0	0	-	No significant similarity	-	-	-
Contig238	FE526005	0	0	0	2	0	0	0	0	0	0	ref|XP_751277.1|	acyltransferase, putative	*Aspergillus fumigatus Af293*	4e-09	1, 43
Contig239	FE526011	0	0	0	2	0	0	0	0	0	0	-	No significant similarity	-	-	-
Contig240	FE525909	0	0	0	2	0	0	0	0	0	0	gb|ABG56823.1|	hypothetical protein	*Klebsiella pneumoniae*	1e-05	99
Contig241	FE526020	0	0	0	3	0	0	0	0	0	0	-	No significant similarity	-	-	-
Contig242	FE525844	0	0	0	2	0	0	0	0	0	0	-	No significant similarity	-	-	-
Contig243	FE525847	0	0	0	3	0	0	0	0	0	0	ref|XP_808355.1|	formin	*Trypanosoma cruzi strain CL Brener*	2e-04	99
Contig244	FE526255	0	0	0	0	0	6	2	0	0	0	ref|XP_001266920.1|	kynurenine aminotransferase, putative	*Neosartorya fischeri NRRL 181*	8e-55	1
Contig245	FE525618	0	5	0	0	0	3	7	0	0	0	ref|XP_750058.1|	carboxylic ester hydrolase (Ppe1), putative	*Aspergillus fumigatus Af293*	2e-24	32
Contig246	FE526254	0	0	0	0	0	1	4	0	1	0	dbj|BAC01275.1|	cytochrome P450nor	*Aspergillus oryzae*	6e-16	1, 16, 32
Contig247	FE526297	0	5	0	0	0	0	6	3	0	0	gb|AAP78735.1|	nonribosomal peptide synthase	*Alternaria brassicae*	4e-07	1, 20, 32
Contig248	FE526306	0	0	0	0	0	0	4	0	0	0	ref|YP_001098942.1|	glycerophosphodiester phosphodiesterase, cytosolic	*Herminiimonas arsenicoxydans*	1e-07	98
Contig249	FE526352	1	0	0	0	0	0	12	0	0	0	ref|XP_747214.1|	glucosamine-6-phosphate deaminase, putative	*Aspergillus fumigatus Af293*	6e-49	1, 2
Contig250	FE526320	0	0	0	2	0	0	1	0	0	0	ref|XP_001273963.1|	MFS transporter, putative	*Aspergillus clavatus NRRL 1*	4e-11	20

Contig251	FE526478	0	0	0	0	0	0	4	0	0	0	ref|XP_001261902.1|	oligopeptide transporter	*Neosartorya fischeri NRRL 181*	9e-43	20
Contig252	FE526732	0	0	0	0	0	0	7	4	0	0	ref|XP_001246883.1|	hypothetical protein CIMG_00654	*Coccidioides immitis RS*	3e-04	99
Contig253	FE526534	1	0	0	0	0	0	13	0	0	0	ref|XP_001275026.1|	oligopeptide transporter	*Aspergillus clavatus NRRL 1*	1e-45	20
Contig254	FE526368	0	0	0	0	0	0	2	0	0	0	-	No significant similarity	-	-	-
Contig255	FE526388	0	0	0	0	0	0	2	0	0	0	ref|XP_001261902.1|	oligopeptide transporter	*Neosartorya fischeri NRRL 181*	4e-21	20
Contig256	FE526510	0	0	0	0	0	0	9	0	0	0	ref|XP_001244025.1|	hypothetical protein CIMG_03466	*Coccidioides immitis RS*	7e-08	99
Contig257	FE526523	0	0	0	0	0	0	23	11	0	0	-	No significant similarity	-	-	-
Contig258	FE526681	0	0	0	0	0	0	1	4	0	0	gb|AAN62296.1|AF440524_83	transposase domain protein	*Pseudomonas aeruginosa*	8e-22	98
Contig259	FE526545	2	0	0	0	0	0	5	0	0	0	-	No significant similarity	-	-	-
Contig260	FE526350	0	0	0	0	0	0	16	2	0	0	ref|XP_001260782.1|	V-type ATPase, B subunit, putative	*Neosartorya fischeri NRRL 181*	1e-20	14, 16, 20
Contig261	FE526480	1	0	0	0	0	1	5	0	0	0	ref|XP_001244970.1|	glucosamine-6-phosphate deaminase	*Coccidioides immitis RS*	1e-16	1, 2
Contig262	FE526464	0	0	0	0	0	0	4	0	0	0	gb|AAR11462.1|	subtilisin-like protease SUB3	*Trichophyton rubrum*	5e-25	14, 32, 43
Contig263	FE526458	0	0	0	0	1	0	3	0	0	0	gb|EEQ28888.1|	MFS oligopeptide transporter, putative	*Microsporum canis CBS 113480*	2e-19	20
Contig264	FE526512	0	0	0	0	0	0	2	0	0	0	ref|XP_553256.2|	ENSANGP00000027824	*Anopheles gambiae str. PEST*	6e-13	99
Contig265	FE526327	0	0	0	0	0	0	9	0	0	0	ref|XP_001275026.1|	oligopeptide transporter	*Aspergillus clavatus NRRL 1*	2e-13	20
Contig266	FE526552	0	0	0	0	0	0	2	0	0	0	ref|XP_747457.1|	DUF895 domain membrane protein	*Aspergillus fumigatus Af293*	4e-18	99
Contig267	FE526553	0	0	0	0	0	0	2	0	0	0	gb|ABL84992.1|	metalloprotease Mep4	*Trichophyton tonsurans*	4e-23	14, 32
Contig268	FE526537	0	0	0	2	0	0	7	0	0	0	-	No significant similarity	-	-	-
Contig269	FE527129	0	0	0	0	0	0	0	0	0	2	-	No significant similarity	-	-	-
Contig270	FE527130	0	0	0	0	0	0	0	0	0	2	-	No significant similarity	-	-	-
Contig271	FE525126	23	0	0	1	2	0	0	0	0	1	-	No significant similarity	-	-	-
Contig272	FE527135	0	0	0	0	0	0	0	0	0	2	ref|XP_001544887.1|	conserved hypothetical protein	*Ajellomyces capsulatus NAm1*	4e-11	99
Contig273	FE525279	1	0	0	0	0	0	0	0	0	3	-	No significant similarity	-	-	-
Contig274	FE527160	0	0	0	0	0	0	0	0	0	3	ref|XP_001273062.1|	non-classical export protein Nce2, putative	*Aspergillus clavatus NRRL 1*	5e-10	14, 20

Contig275	FE527170	0	0	0	0	0	0	0	0	2	1	ref|XP_001265779.1|	fatty acid desaturase, putative	*Neosartorya fischeri NRRL 181*	4e-29	1
Contig276	FE527139	0	0	0	0	0	0	0	0	0	2	ref|XP_001536346.1|	40S ribosomal protein S18	*Ajellomyces capsulatus NAm1*	1e-31	12, 16
Contig277	FE527181	0	0	0	0	0	0	0	0	0	2	-	No significant similarity	-	-	-
Contig278	FE525288	6	0	0	0	0	0	0	0	0	1	-	No significant similarity	-	-	-
Contig279	FE527192	0	0	0	0	0	0	0	0	1	1	ref|XP_001544403.1|	Cox19p	*Ajellomyces capsulatus NAm1*	1e-11	2, 14
Contig280	FE527125	0	0	0	0	0	0	2	0	4	2	-	No significant similarity	-	-	-
Contig281	FE527200	0	0	0	0	0	0	0	0	0	2	-	No significant similarity	-	-	-
Contig282	FE526434	1	0	0	0	0	0	4	0	0	1	-	No significant similarity	-	-	-
Contig283	FE526529	12	0	0	0	1	0	5	0	0	5	-	No significant similarity	-	-	-
Contig284	FE527222	0	0	0	0	0	0	0	0	0	2	-	No significant similarity	-	-	-
Contig285	FE527236	0	0	0	0	0	0	0	0	0	2	ref|XP_001255645.1|	PREDICTED: similar to Keratin associated protein 10-4	*Bos taurus*	1e-07	99
Contig286	FE527245	0	0	0	0	0	0	0	0	0	2	ref|XP_001544767.1|	coatomer beta subunit	*Ajellomyces capsulatus NAm1*	1e-16	16, 20, 42
Contig287	FE527256	0	0	0	0	0	0	0	0	2	1	ref|XP_001242709.1|	hypothetical protein CIMG_06605	*Coccidioides immitis RS*	5e-04	99
Contig288	FE527278	0	0	0	0	0	0	0	0	1	1	-	No significant similarity	-	-	-
Contig289	FE527279	0	0	0	0	0	0	0	0	0	2	-	No significant similarity	-	-	-
Contig290	FE527291	1	0	0	0	0	0	0	0	0	3	ref|XP_752593.1|	mitochondrial hypoxia responsive domain protein	*Aspergillus fumigatus Af293*	1e-24	99
Contig291	FE525689	0	0	1	0	0	0	0	0	0	1	ref|XP_001268941.1|	methyltransferase small domain protein	*Aspergillus clavatus NRRL 1*	7e-23	16, 32
Contig292	FE527330	0	0	0	0	0	0	0	0	0	2	-	No significant similarity	-	-	-
Contig293	FE527328	0	0	0	0	0	0	0	0	0	2	ref|XP_748143.1|	BYS1 domain protein, putative	*Aspergillus fumigatus Af293*	8e-15	99
Contig294	FE527331	0	0	0	0	0	0	0	0	0	2	-	No significant similarity	-	-	-
Contig295	FE527324	0	0	0	0	0	0	0	0	0	3	dbj|BAD90801.1|	histone 3	*Conocephalum conicum*	6e-38	10, 11, 16
Contig296	FE527334	0	0	0	0	0	0	0	0	0	2	ref|XP_001244171.1|	predicted protein	*Coccidioides immitis RS*	4e-05	99
Singlet0001	FE524604	1	0	0	0	0	0	0	0	0	0	-	No significant similarity	-	-	-
Singlet0002	FE524606	1	0	0	0	0	0	0	0	0	0	ref|XP_001241782.1|	conserved hypothetical protein	*Coccidioides immitis RS*	6e-22	99
Singlet0003	FE524607	1	0	0	0	0	0	0	0	0	0	-	No significant similarity	-	-	-
Singlet0004	FE524608	1	0	0	0	0	0	0	0	0	0	-	No significant similarity	-	-	-
Singlet0005	FE524610	1	0	0	0	0	0	0	0	0	0	emb|CAK40436.1|	unnamed protein product	*Aspergillus niger*	7e-08	99
Singlet0006	FE524612	1	0	0	0	0	0	0	0	0	0	ref|XP_001264707.1|	S-adenosylmethionine synthetase	*Neosartorya fischeri NRRL 181*	8e-22	1, 16

Singlet0007	FE524613	1	0	0	0	0	0	0	0	0	0	-	No significant similarity	-	-	-
Singlet0008	FE524616	1	0	0	0	0	0	0	0	0	0	-	No significant similarity	-	-	-
Singlet0009	FE524618	1	0	0	0	0	0	0	0	0	0	-	No significant similarity	-	-	-
Singlet0010	FE524619	1	0	0	0	0	0	0	0	0	0	-	No significant similarity	-	-	-
Singlet0011	FE524621	1	0	0	0	0	0	0	0	0	0	-	No significant similarity	-	-	-
Singlet0012	FE524622	1	0	0	0	0	0	0	0	0	0	-	No significant similarity	-	-	-
Singlet0013	FE524628	1	0	0	0	0	0	0	0	0	0	-	No significant similarity	-	-	-
Singlet0014	FE524631	1	0	0	0	0	0	0	0	0	0	ref|XP_001247117.1|	60S ribosomal protein L44	*Coccidioides immitis RS*	2e-53	12, 16
Singlet0015	FE524635	1	0	0	0	0	0	0	0	0	0	-	No significant similarity	-	-	-
Singlet0016	FE524636	1	0	0	0	0	0	0	0	0	0	-	No significant similarity	-	-	-
Singlet0017	FE524638	1	0	0	0	0	0	0	0	0	0	-	No significant similarity	-	-	-
Singlet0018	FE524640	1	0	0	0	0	0	0	0	0	0	-	No significant similarity	-	-	-
Singlet0019	FE524645	1	0	0	0	0	0	0	0	0	0	ref|XP_001267438.1|	hypothetical protein NFIA_043590	*Neosartorya fischeri NRRL 181*	1e-11	99
Singlet0020	FE524646	1	0	0	0	0	0	0	0	0	0	ref|XP_001240361.1|	hypothetical protein CIMG_07524	*Coccidioides immitis RS*	3e-06	99
Singlet0021	FE524647	1	0	0	0	0	0	0	0	0	0	-	No significant similarity	-	-	-
Singlet0022	FE524649	1	0	0	0	0	0	0	0	0	0	-	No significant similarity	-	-	-
Singlet0023	FE524656	1	0	0	0	0	0	0	0	0	0	-	No significant similarity	-	-	-
Singlet0024	FE524659	1	0	0	0	0	0	0	0	0	0	ref|XP_001217109.1|	methionyl-tRNA synthetase	*Aspergillus terreus NIH2624*	4e-04	11, 12, 16
Singlet0025	FE524660	1	0	0	0	0	0	0	0	0	0	gb|AAP05987.3|	70 kDa heat shock protein	*Paracoccidioides brasiliensis*	8e-77	1, 10, 14, 16, 20, 32
Singlet0026	FE524661	1	0	0	0	0	0	0	0	0	0	-	No significant similarity	-	-	-
Singlet0027	FE524665	1	0	0	0	0	0	0	0	0	0	ref|XP_001273409.1|	dihydrolipoamide acetyltransferase component of pyruvate dehydrogenase	*Aspergillus clavatus NRRL 1*	1e-11	1, 2, 14, 16
Singlet0028	FE524666	1	0	0	0	0	0	0	0	0	0	-	No significant similarity	-	-	-
Singlet0029	FE524667	1	0	0	0	0	0	0	0	0	0	ref|XP_001273409.1|	dihydrolipoamide acetyltransferase component of pyruvate dehydrogenase	*Aspergillus clavatus NRRL 1*	2e-05	1, 2, 14, 16
Singlet0030	FE524668	1	0	0	0	0	0	0	0	0	0	-	No significant similarity	-	-	-
Singlet0031	FE524670	1	0	0	0	0	0	0	0	0	0	-	No significant similarity	-	-	-
Singlet0032	FE524672	1	0	0	0	0	0	0	0	0	0	ref|XP_001216088.1|	splicing factor U2AF 23 kDa subunit	*Aspergillus terreus NIH2624*	4e-08	11, 16
Singlet0033	FE524674	1	0	0	0	0	0	0	0	0	0	-	No significant similarity	-	-	-
Singlet0034	FE524675	1	0	0	0	0	0	0	0	0	0	-	No significant similarity	-	-	-
Singlet0035	FE524676	1	0	0	0	0	0	0	0	0	0	sp|P09188|PGK_PENCH	Phosphoglycerate kinase emb|CAA31756.1| PGK protein	*Penicillium chrysogenum*	8e-15	1, 2, 16
Singlet0036	FE524677	1	0	0	0	0	0	0	0	0	0	-	No significant similarity	-	-	-

Singlet0037	FE524680	1	0	0	0	0	0	0	0	0	0	gb|AAB05810.1|	super cysteine rich protein; SCRP	*Homo sapiens*	4e-10	11, 16
Singlet0038	FE524681	1	0	0	0	0	0	0	0	0	0	-	No significant similarity	-	-	-
Singlet0039	FE524683	1	0	0	0	0	0	0	0	0	0	-	No significant similarity	-	-	-
Singlet0040	FE524685	1	0	0	0	0	0	0	0	0	0	-	No significant similarity	-	-	-
Singlet0041	FE524686	1	0	0	0	0	0	0	0	0	0	-	No significant similarity	-	-	-
Singlet0042	FE524687	1	0	0	0	0	0	0	0	0	0	-	No significant similarity	-	-	-
Singlet0043	FE524691	1	0	0	0	0	0	0	0	0	0	-	No significant similarity	-	-	-
Singlet0044	FE524692	1	0	0	0	0	0	0	0	0	0	-	No significant similarity	-	-	-
Singlet0045	FE524693	1	0	0	0	0	0	0	0	0	0	-	No significant similarity	-	-	-
Singlet0046	FE524695	1	0	0	0	0	0	0	0	0	0	ref|XP_752703.1|	Arp2/3 complex subunit (Arp2), putative	*Aspergillus fumigatus Af293*	2e-69	14, 16, 43
Singlet0047	FE524697	1	0	0	0	0	0	0	0	0	0	-	No significant similarity	-	-	-
Singlet0048	FE524698	1	0	0	0	0	0	0	0	0	0	-	No significant similarity	-	-	-
Singlet0049	FE524700	1	0	0	0	0	0	0	0	0	0	ref|XP_746929.1|	vacuolar protein sorting protein, putative	*Aspergillus fumigatus Af293*	2e-12	14, 16, 20
Singlet0050	FE524702	1	0	0	0	0	0	0	0	0	0	-	No significant similarity	-	-	-
Singlet0051	FE524703	1	0	0	0	0	0	0	0	0	0	ref|XP_001263333.1|	proteasome subunit alpha type	*Neosartorya fischeri NRRL 181*	5e-26	14, 16, 18, 32
Singlet0052	FE524704	1	0	0	0	0	0	0	0	0	0	ref|XP_001222064.1|	40S ribosomal protein S13	*Chaetomium globosum CBS 148.51*	7e-52	12, 16
Singlet0053	FE524705	1	0	0	0	0	0	0	0	0	0	-	No significant similarity	-	-	-
Singlet0054	FE524707	1	0	0	0	0	0	0	0	0	0	ref|XP_001244930.1|	predicted protein	*Coccidioides immitis RS*	1e-08	99
Singlet0055	FE524708	1	0	0	0	0	0	0	0	0	0	-	No significant similarity	-	-	-
Singlet0056	FE524709	1	0	0	0	0	0	0	0	0	0	-	No significant similarity	-	-	-
Singlet0057	FE524711	1	0	0	0	0	0	0	0	0	0	-	No significant similarity	-	-	-
Singlet0058	FE524712	1	0	0	0	0	0	0	0	0	0	ref|XP_755632.1|	cytochrome c oxidase polypeptide vib	*Aspergillus fumigatus Af293*	3e-31	14
Singlet0059	FE524714	1	0	0	0	0	0	0	0	0	0	gb|EAL88345.2|	60S ribosomal protein L27e	*Aspergillus fumigatus Af293*	1e-18	12
Singlet0060	FE524716	1	0	0	0	0	0	0	0	0	0	-	No significant similarity	-	-	-
Singlet0061	FE524718	1	0	0	0	0	0	0	0	0	0	-	No significant similarity	-	-	-
Singlet0062	FE524719	1	0	0	0	0	0	0	0	0	0	-	No significant similarity	-	-	-
Singlet0063	FE524722	1	0	0	0	0	0	0	0	0	0	-	No significant similarity	-	-	-
Singlet0064	FE524723	1	0	0	0	0	0	0	0	0	0	pir||B24264	proline-rich protein MP3 - mouse (fragment)	*Mus musculus*	1e-05	10
Singlet0065	FE524724	1	0	0	0	0	0	0	0	0	0	-	No significant similarity	-	-	-
Singlet0066	FE524725	1	0	0	0	0	0	0	0	0	0	-	No significant similarity	-	-	-
Singlet0067	FE524727	1	0	0	0	0	0	0	0	0	0	ref|XP_001267666.1|	hypothetical protein NFIA_061330	*Neosartorya fischeri NRRL 181*	3e-19	99

Singlet0068	FE524728	1	0	0	0	0	0	0	0	0	0	ref|XP_001245526.1|	hypothetical protein CIMG_04967	*Coccidioides immitis RS*	3e-28	99
Singlet0069	FE524729	1	0	0	0	0	0	0	0	0	0	-	No significant similarity	-	-	-
Singlet0070	FE524730	1	0	0	0	0	0	0	0	0	0	-	No significant similarity	-	-	-
Singlet0071	FE524731	1	0	0	0	0	0	0	0	0	0	-	No significant similarity	-	-	-
Singlet0072	FE524732	1	0	0	0	0	0	0	0	0	0	-	No significant similarity	-	-	-
Singlet0073	FE524734	1	0	0	0	0	0	0	0	0	0	gb|AAY23172.1|	cytochrome c oxidase polypeptide VIa	*Penicillium chrysogenum*	3e-08	14
Singlet0074	FE524742	1	0	0	0	0	0	0	0	0	0	-	No significant similarity	-	-	-
Singlet0075	FE524743	1	0	0	0	0	0	0	0	0	0	-	No significant similarity	-	-	-
Singlet0076	FE524744	1	0	0	0	0	0	0	0	0	0	emb|CAL46260.1|	putative mitochondrial inner membrane protein 1	*Botryotinia fuckeliana*	6e-13	10, 14, 20
Singlet0077	FE524745	1	0	0	0	0	0	0	0	0	0	ref|XP_001213975.1|	protein kinase dsk1	*Aspergillus terreus NIH2624*	3e-17	10, 14, 30
Singlet0078	FE524746	1	0	0	0	0	0	0	0	0	0	sp|Q9P8I0|PUT2_EMENI	Delta-1-pyrroline-5-carboxylate dehydrogenase, mitochondrial precursor (P5C dehydrogenase) gb|AAF72527.1|AF252630_1 delta-1-pyrroline-5-carboxylate dehydrogenase	*Aspergillus nidulans*	8e-17	1, 20
Singlet0079	FE524747	1	0	0	0	0	0	0	0	0	0	-	No significant similarity	-	-	-
Singlet0080	FE524748	1	0	0	0	0	0	0	0	0	0	-	No significant similarity	-	-	-
Singlet0081	FE524753	1	0	0	0	0	0	0	0	0	0	-	No significant similarity	-	-	-
Singlet0082	FE524755	1	0	0	0	0	0	0	0	0	0	-	No significant similarity	-	-	-
Singlet0083	FE524756	1	0	0	0	0	0	0	0	0	0	gb|AAW69337.1|	ERV2 protein-like protein	*Magnaporthe grisea*	4e-17	1, 14
Singlet0084	FE524758	1	0	0	0	0	0	0	0	0	0	-	No significant similarity	-	-	-
Singlet0085	FE524759	1	0	0	0	0	0	0	0	0	0	-	No significant similarity	-	-	-
Singlet0086	FE524763	1	0	0	0	0	0	0	0	0	0	-	No significant similarity	-	-	-
Singlet0087	FE524764	1	0	0	0	0	0	0	0	0	0	ref|XP_001245839.1|	arginyl-tRNA synthetase	*Coccidioides immitis RS*	1e-53	12, 16
Singlet0088	FE524765	1	0	0	0	0	0	0	0	0	0	-	No significant similarity	-	-	-
Singlet0089	FE524767	1	0	0	0	0	0	0	0	0	0	gb|AAR24348.1|	14-3-3-like protein 2	*Paracoccidioides brasiliensis*	3e-81	14, 16, 30, 43
Singlet0090	FE524768	1	0	0	0	0	0	0	0	0	0	-	No significant similarity	-	-	-
Singlet0091	FE524774	1	0	0	0	0	0	0	0	0	0	-	No significant similarity	-	-	-
Singlet0092	FE524775	1	0	0	0	0	0	0	0	0	0	-	No significant similarity	-	-	-
Singlet0093	FE524776	1	0	0	0	0	0	0	0	0	0	-	No significant similarity	-	-	-
Singlet0094	FE524778	1	0	0	0	0	0	0	0	0	0	-	No significant similarity	-	-	-
Singlet0095	FE524779	1	0	0	0	0	0	0	0	0	0	-	No significant similarity	-	-	-
Singlet0096	FE524780	1	0	0	0	0	0	0	0	0	0	-	No significant similarity	-	-	-

Singlet0097	FE524782	1	0	0	0	0	0	0	0	0	0	ref|XP_001263817.1|	UBX domain protein	*Neosartorya fischeri NRRL 181*	1e-21	99
Singlet0098	FE524784	1	0	0	0	0	0	0	0	0	0	-	No significant similarity	-	-	-
Singlet0099	FE524785	1	0	0	0	0	0	0	0	0	0	-	No significant similarity	-	-	-
Singlet0100	FE524788	1	0	0	0	0	0	0	0	0	0	-	No significant similarity	-	-	-
Singlet0101	FE524789	1	0	0	0	0	0	0	0	0	0	-	No significant similarity	-	-	-
Singlet0102	FE524790	1	0	0	0	0	0	0	0	0	0	gb|AAX27763.1|	SJCHGC01957 protein	*Schistosoma japonicum*	4e-16	99
Singlet0103	FE524793	1	0	0	0	0	0	0	0	0	0	ref|XP_001242478.1|	hypothetical protein CIMG_06374	*Coccidioides immitis RS*	3e-06	99
Singlet0104	FE524795	1	0	0	0	0	0	0	0	0	0	-	No significant similarity	-	-	-
Singlet0105	FE524796	1	0	0	0	0	0	0	0	0	0	-	No significant similarity	-	-	-
Singlet0106	FE524799	1	0	0	0	0	0	0	0	0	0	-	No significant similarity	-	-	-
Singlet0107	FE524801	1	0	0	0	0	0	0	0	0	0	ref|XP_001243274.1|	hypothetical protein CIMG_07170	*Coccidioides immitis RS*	5e-39	99
Singlet0108	FE524803	1	0	0	0	0	0	0	0	0	0	ref|XP_001270912.1|	proteasome regulatory particle subunit (RpnL), putative	*Aspergillus clavatus NRRL 1*	2e-61	14, 16, 18, 32
Singlet0109	FE524804	1	0	0	0	0	0	0	0	0	0	ref|XP_001214565.1|	succinate-semialdehyde dehydrogenase	*Aspergillus terreus NIH2624*	1e-05	1, 2, 16, 32
Singlet0110	FE524805	1	0	0	0	0	0	0	0	0	0	-	No significant similarity	-	-	-
Singlet0111	FE524811	1	0	0	0	0	0	0	0	0	0	ref|XP_001271742.1|	acyl-CoA dehydrogenase, putative	*Aspergillus clavatus NRRL 1*	6e-23	1, 2, 10
Singlet0112	FE524812	1	0	0	0	0	0	0	0	0	0	-	No significant similarity	-	-	-
Singlet0113	FE524813	1	0	0	0	0	0	0	0	0	0	-	No significant similarity	-	-	-
Singlet0114	FE524814	1	0	0	0	0	0	0	0	0	0	ref|XP_750122.1|	UPF0136 domain protein	*Aspergillus fumigatus Af293*	8e-25	1, 10, 11, 12, 16
Singlet0115	FE524816	1	0	0	0	0	0	0	0	0	0	-	No significant similarity	-	-	-
Singlet0116	FE524820	1	0	0	0	0	0	0	0	0	0	-	No significant similarity	-	-	-
Singlet0117	FE524822	1	0	0	0	0	0	0	0	0	0	-	No significant similarity	-	-	-
Singlet0118	FE524823	1	0	0	0	0	0	0	0	0	0	-	No significant similarity	-	-	-
Singlet0119	FE524825	1	0	0	0	0	0	0	0	0	0	-	No significant similarity	-	-	-
Singlet0120	FE524827	1	0	0	0	0	0	0	0	0	0	-	No significant similarity	-	-	-
Singlet0121	FE524828	1	0	0	0	0	0	0	0	0	0	-	No significant similarity	-	-	-
Singlet0122	FE524831	1	0	0	0	0	0	0	0	0	0	-	No significant similarity	-	-	-
Singlet0123	FE524833	1	0	0	0	0	0	0	0	0	0	ref|XP_001247270.1|	hypothetical protein CIMG_01041	*Coccidioides immitis RS*	3e-12	99
Singlet0124	FE524836	1	0	0	0	0	0	0	0	0	0	-	No significant similarity	-	-	-
Singlet0125	FE524838	1	0	0	0	0	0	0	0	0	0	ref|XP_001241990.1|	tubulin gamma chain	*Coccidioides immitis RS*	1e-95	1, 10, 16
Singlet0126	FE524839	1	0	0	0	0	0	0	0	0	0	-	No significant similarity	-	-	-
Singlet0127	FE524842	1	0	0	0	0	0	0	0	0	0	-	No significant similarity	-	-	-

Singlet0128	FE524844	1	0	0	0	0	0	0	0	0	0	-	No significant similarity	-	-	-
Singlet0129	FE524845	1	0	0	0	0	0	0	0	0	0	ref|XP_001275472.1|	60S ribosomal protein L3	*Aspergillus clavatus NRRL 1*	8e-25	12, 16
Singlet0130	FE524846	1	0	0	0	0	0	0	0	0	0	-	No significant similarity	-	-	-
Singlet0131	FE524847	1	0	0	0	0	0	0	0	0	0	-	No significant similarity	-	-	-
Singlet0132	FE524849	1	0	0	0	0	0	0	0	0	0	sp|P39457|PLB1_PENCH	Lysophospholipase precursor (Phospholipase B) emb|CAA42906.1| lysophospholipase	*Penicillium chrysogenum*	4e-04	1
Singlet0133	FE524851	1	0	0	0	0	0	0	0	0	0	-	No significant similarity	-	-	-
Singlet0134	FE524853	1	0	0	0	0	0	0	0	0	0	-	No significant similarity	-	-	-
Singlet0135	FE524855	1	0	0	0	0	0	0	0	0	0	ref|XP_001212274.1|	40S ribosomal protein S19	*Aspergillus terreus NIH2624*	1e-48	12, 16
Singlet0136	FE524858	1	0	0	0	0	0	0	0	0	0	-	No significant similarity	-	-	-
Singlet0137	FE524859	1	0	0	0	0	0	0	0	0	0	-	No significant similarity	-	-	-
Singlet0138	FE524861	1	0	0	0	0	0	0	0	0	0	-	No significant similarity	-	-	-
Singlet0139	FE524865	1	0	0	0	0	0	0	0	0	0	-	No significant similarity	-	-	-
Singlet0140	FE524867	1	0	0	0	0	0	0	0	0	0	-	No significant similarity	-	-	-
Singlet0141	FE524868	1	0	0	0	0	0	0	0	0	0	-	No significant similarity	-	-	-
Singlet0142	FE524869	1	0	0	0	0	0	0	0	0	0	-	No significant similarity	-	-	-
Singlet0143	FE524871	1	0	0	0	0	0	0	0	0	0	-	No significant similarity	-	-	-
Singlet0144	FE524872	1	0	0	0	0	0	0	0	0	0	-	No significant similarity	-	-	-
Singlet0145	FE524873	1	0	0	0	0	0	0	0	0	0	-	No significant similarity	-	-	-
Singlet0146	FE524876	1	0	0	0	0	0	0	0	0	0	ref|XP_001274544.1|	N-acetylglucosaminyl-phosphatidylinositol deacetylase, putative	*Aspergillus clavatus NRRL 1*	2e-20	1
Singlet0147	FE524879	1	0	0	0	0	0	0	0	0	0	-	No significant similarity	-	-	-
Singlet0148	FE524880	1	0	0	0	0	0	0	0	0	0	ref|XP_828313.1|	hypothetical protein Tb11.18.0001	*Trypanosoma brucei TREU927*	9e-21	99
Singlet0149	FE524881	1	0	0	0	0	0	0	0	0	0	ref|XP_385039.1|	hypothetical protein FG04863.1	*Gibberella zeae PH-1*	1e-06	99
Singlet0150	FE524883	1	0	0	0	0	0	0	0	0	0	gb|ABB20530.1|	3-hydroxyphenylacetate 6 hydroxylase	*Emericella nidulans*	6e-12	1, 2
Singlet0151	FE524886	1	0	0	0	0	0	0	0	0	0	-	No significant similarity	-	-	-
Singlet0152	FE524887	1	0	0	0	0	0	0	0	0	0	emb|CAE72981.1|	Hypothetical protein CBG20323	*Caenorhabditis briggsae*	5e-16	99
Singlet0153	FE524888	1	0	0	0	0	0	0	0	0	0	-	No significant similarity	-	-	-
Singlet0154	FE524889	1	0	0	0	0	0	0	0	0	0	-	No significant similarity	-	-	-
Singlet0155	FE524890	1	0	0	0	0	0	0	0	0	0	ref|XP_001257424.1|	ubiquitin C-terminal hydrolase, putative	*Neosartorya fischeri NRRL 181*	2e-08	14
Singlet0156	FE524891	1	0	0	0	0	0	0	0	0	0	ref|XP_001275443.1|	galactose-1-phosphate uridylyltransferase	*Aspergillus clavatus NRRL 1*	2e-34	1, 16

Singlet0157	FE524892	1	0	0	0	0	0	0	0	0	0	-	No significant similarity	-	-	-
Singlet0158	FE524893	1	0	0	0	0	0	0	0	0	0	-	No significant similarity	-	-	-
Singlet0159	FE524895	1	0	0	0	0	0	0	0	0	0	gb|EU076569.1|	Dipeptidyl-peptidase 5 (DPP5)	*Trichophyton equinum*	2e-35	14
Singlet0160	FE524897	1	0	0	0	0	0	0	0	0	0	ref|XP_749924.1|	t-complex protein 1, beta subunit, putative	*Aspergillus fumigatus Af293*	6e-52	14, 16, 32, 42
Singlet0161	FE524898	1	0	0	0	0	0	0	0	0	0	-	No significant similarity	-	-	-
Singlet0162	FE524899	1	0	0	0	0	0	0	0	0	0	ref|XP_001358279.1|	GA19372-PA	*Drosophila pseudoobscura*	3e-10	99
Singlet0163	FE524905	1	0	0	0	0	0	0	0	0	0	-	No significant similarity	-	-	-
Singlet0164	FE524906	1	0	0	0	0	0	0	0	0	0	ref|XP_001247816.1|	superoxide dismutase	*Mn*	1e-08	16, 32, 34
Singlet0165	FE524908	1	0	0	0	0	0	0	0	0	0	-	No significant similarity	-	-	-
Singlet0166	FE524910	1	0	0	0	0	0	0	0	0	0	-	No significant similarity	-	-	-
Singlet0167	FE524911	1	0	0	0	0	0	0	0	0	0	ref|XP_001264967.1|	30S ribosomal subunit S4, putative	*Neosartorya fischeri NRRL 181*	2e-28	12
Singlet0168	FE524914	1	0	0	0	0	0	0	0	0	0	-	No significant similarity	-	-	-
Singlet0169	FE524915	1	0	0	0	0	0	0	0	0	0	gb|ABH10636.1|	elongation factor 2	*Coccidioides posadasii*	1e-33	1, 11, 12, 16
Singlet0170	FE524916	1	0	0	0	0	0	0	0	0	0	-	No significant similarity	-	-	-
Singlet0171	FE524920	1	0	0	0	0	0	0	0	0	0	ref|XP_001208527.1|	cytochrome c oxidase assembly protein COX11, mitochondrial precursor	*Aspergillus terreus NIH2624*	1e-26	14
Singlet0172	FE524924	1	0	0	0	0	0	0	0	0	0	-	No significant similarity	-	-	-
Singlet0173	FE524925	1	0	0	0	0	0	0	0	0	0	-	No significant similarity	-	-	-
Singlet0174	FE524926	1	0	0	0	0	0	0	0	0	0	gb|AAX82553.1|	120 kDa pistil extensin-like protein	*Nicotiana langsdorfii*	2e-04	99
Singlet0175	FE524927	1	0	0	0	0	0	0	0	0	0	-	No significant similarity	-	-	-
Singlet0176	FE524928	1	0	0	0	0	0	0	0	0	0	ref|XP_571345.1|	ketol-acid reductoisomerase	*Cryptococcus neoformans var. neoformans JEC21*	7e-13	1
Singlet0177	FE524929	1	0	0	0	0	0	0	0	0	0	-	No significant similarity	-	-	-
Singlet0178	FE524933	1	0	0	0	0	0	0	0	0	0	-	No significant similarity	-	-	-
Singlet0179	FE524934	1	0	0	0	0	0	0	0	0	0	-	No significant similarity	-	-	-
Singlet0180	FE524935	1	0	0	0	0	0	0	0	0	0	-	No significant similarity	-	-	-
Singlet0181	FE524936	1	0	0	0	0	0	0	0	0	0	gb|EDJ97592.1|	sodium P-type ATPase, putative	*Magnaporthe grisea 70-15*	6e-04	20, 32, 34
Singlet0182	FE524937	1	0	0	0	0	0	0	0	0	0	ref|XP_001258284.1|	polyketide synthase, putative	*Neosartorya fischeri NRRL 181*	5e-14	1, 32
Singlet0183	FE524938	1	0	0	0	0	0	0	0	0	0	ref|XP_001244923.1|	hypothetical protein CIMG_04364	*Coccidioides immitis RS*	2e-04	99
Singlet0184	FE524939	1	0	0	0	0	0	0	0	0	0	-	No significant similarity	-	-	-

Singlet0185	FE524940	1	0	0	0	0	0	0	0	0	0	gb|EAW96235.1|	hCG2043380	*Homo sapiens*	1e-06	99
Singlet0186	FE524942	1	0	0	0	0	0	0	0	0	0	-	No significant similarity	-	-	-
Singlet0187	FE524944	1	0	0	0	0	0	0	0	0	0	-	No significant similarity	-	-	-
Singlet0188	FE524945	1	0	0	0	0	0	0	0	0	0	-	No significant similarity	-	-	-
Singlet0189	FE524947	1	0	0	0	0	0	0	0	0	0	-	No significant similarity	-	-	-
Singlet0190	FE524948	1	0	0	0	0	0	0	0	0	0	-	No significant similarity	-	-	-
Singlet0191	FE524950	1	0	0	0	0	0	0	0	0	0	gb|EAL88703.2|	adenylate cyclase AcyA	*Aspergillus fumigatus Af293*	3e-13	1, 30
Singlet0192	FE524951	1	0	0	0	0	0	0	0	0	0	ref|XP_001264585.1|	COPII-coated vesicle protein SurF4/Erv29, putative	*Neosartorya fischeri NRRL 181*	1e-27	16, 20
Singlet0193	FE524953	1	0	0	0	0	0	0	0	0	0	gb|AAY66614.1|	putative secreted protein	*Ixodes scapularis*	5e-05	99
Singlet0194	FE524958	1	0	0	0	0	0	0	0	0	0	-	No significant similarity	-	-	-
Singlet0195	FE524961	1	0	0	0	0	0	0	0	0	0	ref|XP_001276223.1|	WD domain protein	*Aspergillus clavatus NRRL 1*	2e-23	11, 40
Singlet0196	FE524965	1	0	0	0	0	0	0	0	0	0	ref|XP_001242010.1|	60S ribosomal protein L5	*Coccidioides immitis RS*	2e-46	12, 16
Singlet0197	FE524967	1	0	0	0	0	0	0	0	0	0	-	No significant similarity	-	-	-
Singlet0198	FE524968	1	0	0	0	0	0	0	0	0	0	-	No significant similarity	-	-	-
Singlet0199	FE524970	1	0	0	0	0	0	0	0	0	0	-	No significant similarity	-	-	-
Singlet0200	FE524971	1	0	0	0	0	0	0	0	0	0	-	No significant similarity	-	-	-
Singlet0201	FE524973	1	0	0	0	0	0	0	0	0	0	-	No significant similarity	-	-	-
Singlet0202	FE524975	1	0	0	0	0	0	0	0	0	0	-	No significant similarity	-	-	-
Singlet0203	FE524980	1	0	0	0	0	0	0	0	0	0	ref|XP_001212080.1|	enolase	*Aspergillus terreus NIH2624*	7e-04	1, 2, 16
Singlet0204	FE524982	1	0	0	0	0	0	0	0	0	0	ref|XP_001271153.1|	FHA domain protein SNIP1, putative	*Aspergillus clavatus NRRL 1*	1e-09	99
Singlet0205	FE524984	1	0	0	0	0	0	0	0	0	0	-	No significant similarity	-	-	-
Singlet0206	FE524987	1	0	0	0	0	0	0	0	0	0	-	No significant similarity	-	-	-
Singlet0207	FE524991	1	0	0	0	0	0	0	0	0	0	-	No significant similarity	-	-	-
Singlet0208	FE524992	1	0	0	0	0	0	0	0	0	0	ref|XP_752433.1|	Vacuolar ATP synthase subunit H, putative	*Aspergillus fumigatus Af293*	2e-39	2, 20, 34
Singlet0209	FE524993	1	0	0	0	0	0	0	0	0	0	ref|XP_001268628.1|	40S ribosomal protein S25, putative	*Aspergillus clavatus NRRL 1*	2e-25	12, 16
Singlet0210	FE524994	1	0	0	0	0	0	0	0	0	0	ref|XP_001274344.1|	1,4-alpha-glucan branching enzyme	*Aspergillus clavatus NRRL 1*	3e-19	1
Singlet0211	FE524996	1	0	0	0	0	0	0	0	0	0	-	No significant similarity	-	-	-
Singlet0212	FE524998	1	0	0	0	0	0	0	0	0	0	-	No significant similarity	-	-	-
Singlet0213	FE525000	1	0	0	0	0	0	0	0	0	0	ref|XP_001267428.1|	NADH-ubiquinone oxidoreductase 21 kDa subunit, putative	*Neosartorya fischeri NRRL 181*	8e-41	2
Singlet0214	FE525003	1	0	0	0	0	0	0	0	0	0	-	No significant similarity	-	-	-

Singlet0215	FE525004	1	0	0	0	0	0	0	0	0	0	emb|CAD22154.1|	pherophorin-dz1 protein	*Volvox carteri f. nagariensis*	2e-13	99
Singlet0216	FE525008	1	0	0	0	0	0	0	0	0	0	-	No significant similarity	-	-	-
Singlet0217	FE525009	1	0	0	0	0	0	0	0	0	0	-	No significant similarity	-	-	-
Singlet0218	FE525010	1	0	0	0	0	0	0	0	0	0	-	No significant similarity	-	-	-
Singlet0219	FE525016	1	0	0	0	0	0	0	0	0	0	-	No significant similarity	-	-	-
Singlet0220	FE525019	1	0	0	0	0	0	0	0	0	0	ref|XP_001254275.1|	PREDICTED: hypothetical protein	*Bos taurus*	3e-15	99
Singlet0221	FE525020	1	0	0	0	0	0	0	0	0	0	-	No significant similarity	-	-	-
Singlet0222	FE525029	1	0	0	0	0	0	0	0	0	0	-	No significant similarity	-	-	-
Singlet0223	FE525030	1	0	0	0	0	0	0	0	0	0	-	No significant similarity	-	-	-
Singlet0224	FE525031	1	0	0	0	0	0	0	0	0	0	ref|XP_001258304.1|	benzoate 4-monooxygenase cytochrome P450	*Neosartorya fischeri NRRL 181*	3e-09	1
Singlet0225	FE525032	1	0	0	0	0	0	0	0	0	0	ref|XP_001259888.1|	ABC metal ion transporter, putative	*Neosartorya fischeri NRRL 181*	1e-15	99
Singlet0226	FE525036	1	0	0	0	0	0	0	0	0	0	-	No significant similarity	-	-	-
Singlet0227	FE525038	1	0	0	0	0	0	0	0	0	0	-	No significant similarity	-	-	-
Singlet0228	FE525039	1	0	0	0	0	0	0	0	0	0	sp|Q9C3Z6|RLA0_PODAN	60S acidic ribosomal protein P0 gb|AAK11262.1|AF331714_1 ribosomal protein P0	*Podospora anserina*	1e-05	12, 16
Singlet0229	FE525040	1	0	0	0	0	0	0	0	0	0	gb|ABH10639.1|	fructose biphosphate aldolase	*Coccidioides posadasii*	1e-59	1, 2
Singlet0230	FE525041	1	0	0	0	0	0	0	0	0	0	-	No significant similarity	-	-	-
Singlet0231	FE525043	1	0	0	0	0	0	0	0	0	0	-	No significant similarity	-	-	-
Singlet0232	FE525044	1	0	0	0	0	0	0	0	0	0	-	No significant similarity	-	-	-
Singlet0233	FE525046	1	0	0	0	0	0	0	0	0	0	-	No significant similarity	-	-	-
Singlet0234	FE525047	1	0	0	0	0	0	0	0	0	0	ref|XP_001264877.1|	heavy metal ion transporter, putative	*Neosartorya fischeri NRRL 181*	1e-10	99
Singlet0235	FE525049	1	0	0	0	0	0	0	0	0	0	-	No significant similarity	-	-	-
Singlet0236	FE525050	1	0	0	0	0	0	0	0	0	0	ref|XP_001244689.1|	hypothetical protein CIMG_04130	*Coccidioides immitis RS*	8e-05	99
Singlet0237	FE525052	1	0	0	0	0	0	0	0	0	0	-	No significant similarity	-	-	-
Singlet0238	FE525053	1	0	0	0	0	0	0	0	0	0	ref|NP_173553.1|	ATEXT3 (EXTENSIN 3); structural constituent of cell wall	*Arabidopsis thaliana*	4e-05	99
Singlet0239	FE525054	1	0	0	0	0	0	0	0	0	0	-	No significant similarity	-	-	-
Singlet0240	FE525058	1	0	0	0	0	0	0	0	0	0	-	No significant similarity	-	-	-
Singlet0241	FE525059	1	0	0	0	0	0	0	0	0	0	ref|XP_001265868.1|	ribosomal protein L34 protein, putative	*Neosartorya fischeri NRRL 181*	1e-07	12
Singlet0242	FE525060	1	0	0	0	0	0	0	0	0	0	-	No significant similarity	-	-	-
Singlet0243	FE525061	1	0	0	0	0	0	0	0	0	0	-	No significant similarity	-	-	-
Singlet0244	FE525063	1	0	0	0	0	0	0	0	0	0	gb|EEQ28799.1|	alpha 1,6 mannosyltransferase	*Microsporum canis CBS 113480*	2e-18	1, 14, 32, 43
Singlet0245	FE525064	1	0	0	0	0	0	0	0	0	0	-	No significant similarity	-	-	-

Singlet0246	FE525065	1	0	0	0	0	0	0	0	0	0	-	No significant similarity	-	-	-
Singlet0247	FE525067	1	0	0	0	0	0	0	0	0	0	ref|XP_001261287.1|	tetratricopeptide repeat domain protein	*Neosartorya fischeri NRRL 181*	2e-05	14
Singlet0248	FE525068	1	0	0	0	0	0	0	0	0	0	-	No significant similarity	-	-	-
Singlet0249	FE525069	1	0	0	0	0	0	0	0	0	0	-	No significant similarity	-	-	-
Singlet0250	FE525071	1	0	0	0	0	0	0	0	0	0	-	No significant similarity	-	-	-
Singlet0251	FE525073	1	0	0	0	0	0	0	0	0	0	-	No significant similarity	-	-	-
Singlet0252	FE525074	1	0	0	0	0	0	0	0	0	0	-	No significant similarity	-	-	-
Singlet0253	FE525075	1	0	0	0	0	0	0	0	0	0	ref|XP_001272337.1|	AIF-like mitochondrial oxidoreductase (Nfrl), putative	*Aspergillus clavatus NRRL 1*	3e-17	1, 16
Singlet0254	FE525076	1	0	0	0	0	0	0	0	0	0	-	No significant similarity	-	-	-
Singlet0255	FE525077	1	0	0	0	0	0	0	0	0	0	dbj|BAB12232.1|	fructose 1,6-bisphosphate aldolase	*Aspergillus oryzae*	2e-09	1, 2
Singlet0256	FE525079	1	0	0	0	0	0	0	0	0	0	ref|XP_001268220.1|	TBC domain protein, putative	*Aspergillus clavatus NRRL 1*	1e-12	99
Singlet0257	FE525081	1	0	0	0	0	0	0	0	0	0	-	No significant similarity	-	-	-
Singlet0258	FE525082	1	0	0	0	0	0	0	0	0	0	-	No significant similarity	-	-	-
Singlet0259	FE525083	1	0	0	0	0	0	0	0	0	0	-	No significant similarity	-	-	-
Singlet0260	FE525084	1	0	0	0	0	0	0	0	0	0	-	No significant similarity	-	-	-
Singlet0261	FE525087	1	0	0	0	0	0	0	0	0	0	-	No significant similarity	-	-	-
Singlet0262	FE525090	1	0	0	0	0	0	0	0	0	0	-	No significant similarity	-	-	-
Singlet0263	FE525092	1	0	0	0	0	0	0	0	0	0	-	No significant similarity	-	-	-
Singlet0264	FE525093	1	0	0	0	0	0	0	0	0	0	-	No significant similarity	-	-	-
Singlet0265	FE525094	1	0	0	0	0	0	0	0	0	0	-	No significant similarity	-	-	-
Singlet0266	FE525095	1	0	0	0	0	0	0	0	0	0	-	No significant similarity	-	-	-
Singlet0267	FE525096	1	0	0	0	0	0	0	0	0	0	ref|XP_001246644.1|	protein translation factor SUI1	*Coccidioides immitis RS*	2e-41	12
Singlet0268	FE525099	1	0	0	0	0	0	0	0	0	0	-	No significant similarity	-	-	-
Singlet0269	FE525102	1	0	0	0	0	0	0	0	0	0	ref|XP_001263624.1|	eukaryotic translation initiation factor 4, putative	*Neosartorya fischeri NRRL 181*	2e-25	12
Singlet0270	FE525103	1	0	0	0	0	0	0	0	0	0	gb|AAQ10290.1|	class V chitin synthase	*Coccidioides posadasii*	5e-06	1, 42
Singlet0271	FE525107	1	0	0	0	0	0	0	0	0	0	ref|XP_001276223.1|	WD domain protein	*Aspergillus clavatus NRRL 1*	8e-25	11, 40
Singlet0272	FE525111	1	0	0	0	0	0	0	0	0	0	gb|EAW96235.1|	hCG2043380	*Homo sapiens*	1e-06	99
Singlet0273	FE525112	1	0	0	0	0	0	0	0	0	0	-	No significant similarity	-	-	-
Singlet0274	FE525113	1	0	0	0	0	0	0	0	0	0	-	No significant similarity	-	-	-
Singlet0275	FE525118	1	0	0	0	0	0	0	0	0	0	ref|YP_293958.1|	putative membrane protein	*Emiliania huxleyi virus 86*	8e-08	99
Singlet0276	FE525119	1	0	0	0	0	0	0	0	0	0	ref|XP_001240069.1|	ATP-citrate synthase subunit 1	*Coccidioides immitis RS*	3e-28	1, 2, 42
Singlet0277	FE525122	1	0	0	0	0	0	0	0	0	0	-	No significant similarity	-	-	-

Singlet0278	FE525125	1	0	0	0	0	0	0	0	0	0	ref|XP_001260256.1|	replication factor A 1, rfa1	*Neosartorya fischeri NRRL 181*	1e-22	10, 16, 18, 32
Singlet0279	FE525128	1	0	0	0	0	0	0	0	0	0	ref|XP_001246273.1|	ADP-ribosylation factor 6	*Coccidioides immitis RS*	5e-21	1, 14, 16, 20
Singlet0280	FE525130	1	0	0	0	0	0	0	0	0	0	-	No significant similarity	-	-	-
Singlet0281	FE525131	1	0	0	0	0	0	0	0	0	0	ref|XP_001210992.1|	homocitrate synthase, mitochondrial precursor	*Aspergillus terreus NIH2624*	1e-80	1, 11, 16, 34
Singlet0282	FE525133	1	0	0	0	0	0	0	0	0	0	-	No significant similarity	-	-	-
Singlet0283	FE525134	1	0	0	0	0	0	0	0	0	0	gb|AAL09828.1|	beta-glucosidase 4	*Coccidioides immitis*	1e-16	1
Singlet0284	FE525135	1	0	0	0	0	0	0	0	0	0	-	No significant similarity	-	-	-
Singlet0285	FE525137	1	0	0	0	0	0	0	0	0	0	gb|AAX33296.1|	heat shock protein 90	*Paracoccidioides brasiliensis*	1e-31	14, 16, 32
Singlet0286	FE525138	1	0	0	0	0	0	0	0	0	0	emb|CAK47870.1|	unnamed protein product	*Aspergillus niger*	3e-10	99
Singlet0287	FE525139	1	0	0	0	0	0	0	0	0	0	-	No significant similarity	-	-	-
Singlet0288	FE525140	1	0	0	0	0	0	0	0	0	0	gb|EAT87923.1|	hypothetical protein SNOG_04163	*Phaeosphaeria nodorum SN15*	3e-14	99
Singlet0289	FE525141	1	0	0	0	0	0	0	0	0	0	-	No significant similarity	-	-	-
Singlet0290	FE525142	1	0	0	0	0	0	0	0	0	0	-	No significant similarity	-	-	-
Singlet0291	FE525143	1	0	0	0	0	0	0	0	0	0	ref|XP_001276036.1|	sulfatase domain protein	*Aspergillus clavatus NRRL 1*	9e-18	1, 16, 20
Singlet0292	FE525144	1	0	0	0	0	0	0	0	0	0	gb|ABA33785.1|	RACK1-like protein	*Paracoccidioides brasiliensis*	2e-42	99
Singlet0293	FE525145	1	0	0	0	0	0	0	0	0	0	-	No significant similarity	-	-	-
Singlet0294	FE525146	1	0	0	0	0	0	0	0	0	0	-	No significant similarity	-	-	-
Singlet0295	FE525148	1	0	0	0	0	0	0	0	0	0	-	No significant similarity	-	-	-
Singlet0296	FE525149	1	0	0	0	0	0	0	0	0	0	-	No significant similarity	-	-	-
Singlet0297	FE525150	1	0	0	0	0	0	0	0	0	0	-	No significant similarity	-	-	-
Singlet0298	FE525151	1	0	0	0	0	0	0	0	0	0	ref|XP_001269061.1|	glycine cleavage system H protein	*Aspergillus clavatus NRRL 1*	5e-27	1, 16
Singlet0299	FE525152	1	0	0	0	0	0	0	0	0	0	emb|CAM67156.1|	Hypothetical repeat protein	*Leishmania infantum*	5e-06	99
Singlet0300	FE525153	1	0	0	0	0	0	0	0	0	0	-	No significant similarity	-	-	-
Singlet0301	FE525154	1	0	0	0	0	0	0	0	0	0	-	No significant similarity	-	-	-
Singlet0302	FE525156	1	0	0	0	0	0	0	0	0	0	-	No significant similarity	-	-	-
Singlet0303	FE525158	1	0	0	0	0	0	0	0	0	0	-	No significant similarity	-	-	-
Singlet0304	FE525159	1	0	0	0	0	0	0	0	0	0	-	No significant similarity	-	-	-
Singlet0305	FE525162	1	0	0	0	0	0	0	0	0	0	-	No significant similarity	-	-	-
Singlet0306	FE525163	1	0	0	0	0	0	0	0	0	0	-	No significant similarity	-	-	-
Singlet0307	FE525165	1	0	0	0	0	0	0	0	0	0	ref|XP_001214063.1|	cytochrome c1, mitochondrial precursor	*Aspergillus terreus NIH2624*	8e-14	2, 16, 20

Singlet0308	FE525166	1	0	0	0	0	0	0	0	0	0	ref|XP_755078.1|	fimbrin	*Aspergillus fumigatus Af293*	1e-13	99
Singlet0309	FE525167	1	0	0	0	0	0	0	0	0	0	ref|XP_001248022.1|	threonyl-tRNA synthetase, cytoplasmic	*Coccidioides immitis RS*	7e-09	11, 12, 16
Singlet0310	FE525169	1	0	0	0	0	0	0	0	0	0	-	No significant similarity	-	-	-
Singlet0311	FE525170	1	0	0	0	0	0	0	0	0	0	-	No significant similarity	-	-	-
Singlet0312	FE525171	1	0	0	0	0	0	0	0	0	0	-	No significant similarity	-	-	-
Singlet0313	FE525172	1	0	0	0	0	0	0	0	0	0	ref|XP_001269707.1|	Ribosomal L18ae protein family	*Aspergillus clavatus NRRL 1*	5e-05	12, 16
Singlet0314	FE525173	1	0	0	0	0	0	0	0	0	0	-	No significant similarity	-	-	-
Singlet0315	FE525176	1	0	0	0	0	0	0	0	0	0	-	No significant similarity	-	-	-
Singlet0316	FE525180	1	0	0	0	0	0	0	0	0	0	ref|XP_001272046.1|	splicing factor 3a subunit 2, putative	*Aspergillus clavatus NRRL 1*	1e-10	11
Singlet0317	FE525183	1	0	0	0	0	0	0	0	0	0	-	No significant similarity	-	-	-
Singlet0318	FE525185	1	0	0	0	0	0	0	0	0	0	ref|XP_623110.1|	PREDICTED: similar to CG3195-PA, isoform A	*Apis mellifera*	3e-21	99
Singlet0319	FE525188	1	0	0	0	0	0	0	0	0	0	ref|XP_001243274.1|	hypothetical protein CIMG_07170	*Coccidioides immitis RS*	1e-08	99
Singlet0320	FE525189	1	0	0	0	0	0	0	0	0	0	-	No significant similarity	-	-	-
Singlet0321	FE525191	1	0	0	0	0	0	0	0	0	0	ref|XP_001239918.1|	hypothetical protein CIMG_09539	*Coccidioides immitis RS*	8e-35	99
Singlet0322	FE525194	1	0	0	0	0	0	0	0	0	0	-	No significant similarity	-	-	-
Singlet0323	FE525196	1	0	0	0	0	0	0	0	0	0	-	No significant similarity	-	-	-
Singlet0324	FE525198	1	0	0	0	0	0	0	0	0	0	dbj|BAE65360.1|	unnamed protein product	*Aspergillus oryzae*	4e-04	99
Singlet0325	FE525199	1	0	0	0	0	0	0	0	0	0	-	No significant similarity	-	-	-
Singlet0326	FE525200	1	0	0	0	0	0	0	0	0	0	-	No significant similarity	-	-	-
Singlet0327	FE525201	1	0	0	0	0	0	0	0	0	0	ref|XP_001257727.1|	TOM complex component Tom7, putative	*Neosartorya fischeri NRRL 181*	1e-13	14, 20
Singlet0328	FE525203	1	0	0	0	0	0	0	0	0	0	ref|XP_001273715.1|	acyl-CoA dehydrogenase, putative	*Aspergillus clavatus NRRL 1*	2e-17	1, 2, 10
Singlet0329	FE525204	1	0	0	0	0	0	0	0	0	0	-	No significant similarity	-	-	-
Singlet0330	FE525205	1	0	0	0	0	0	0	0	0	0	ref|XP_001269192.1|	proteasome regulatory particle subunit (Nas6), putative	*Aspergillus clavatus NRRL 1*	2e-10	14, 16, 18, 32
Singlet0331	FE525206	1	0	0	0	0	0	0	0	0	0	ref|XP_001265788.1|	UDP-glucose:sterol glycosyltransferase	*Neosartorya fischeri NRRL 181*	4e-05	1
Singlet0332	FE525207	1	0	0	0	0	0	0	0	0	0	ref|XP_965645.1|	ATP synthase alpha chain, mitochondrial precursor	*Neurospora crassa OR74A*	4e-28	2, 16, 20, 34
Singlet0333	FE525212	1	0	0	0	0	0	0	0	0	0	ref|XP_001213339.1|	glutathione peroxidase	*Aspergillus terreus NIH2624*	5e-12	16. 32
Singlet0334	FE525214	1	0	0	0	0	0	0	0	0	0	-	No significant similarity	-	-	-
Singlet0335	FE525218	1	0	0	0	0	0	0	0	0	0	-	No significant similarity	-	-	-
Singlet0336	FE525219	1	0	0	0	0	0	0	0	0	0	-	No significant similarity	-	-	-

Singlet0337	FE525220	1	0	0	0	0	0	0	0	0	0	-	No significant similarity	-	-	-
Singlet0338	FE525222	1	0	0	0	0	0	0	0	0	0	ref|XP_568855.1|	glucose transporter	*Cryptococcus neoformans var. neoformans JEC21*	2e-22	1, 20, 34
Singlet0339	FE525223	1	0	0	0	0	0	0	0	0	0	-	No significant similarity	-	-	-
Singlet0340	FE525226	1	0	0	0	0	0	0	0	0	0	-	No significant similarity	-	-	-
Singlet0341	FE525227	1	0	0	0	0	0	0	0	0	0	ref|XP_001214668.1|	acetolactate synthase small subunit, mitochondrial precursor	*Aspergillus terreus NIH2624*	3e-05	2, 20
Singlet0342	FE525230	1	0	0	0	0	0	0	0	0	0	-	No significant similarity	-	-	-
Singlet0343	FE525232	1	0	0	0	0	0	0	0	0	0	-	No significant similarity	-	-	-
Singlet0344	FE525237	1	0	0	0	0	0	0	0	0	0	-	No significant similarity	-	-	-
Singlet0345	FE525241	1	0	0	0	0	0	0	0	0	0	-	No significant similarity	-	-	-
Singlet0346	FE525243	1	0	0	0	0	0	0	0	0	0	-	No significant similarity	-	-	-
Singlet0347	FE525244	1	0	0	0	0	0	0	0	0	0	-	No significant similarity	-	-	-
Singlet0348	FE525248	1	0	0	0	0	0	0	0	0	0	-	No significant similarity	-	-	-
Singlet0349	FE525252	1	0	0	0	0	0	0	0	0	0	ref|XP_752345.1|	stomatin family protein	*Aspergillus fumigatus Af293*	2e-06	20, 30
Singlet0350	FE525253	1	0	0	0	0	0	0	0	0	0	ref|XP_001246620.1|	40S ribosomal protein S4	*Coccidioides immitis RS*	3e-14	12
Singlet0351	FE525254	1	0	0	0	0	0	0	0	0	0	ref|XP_001264282.1|	transcription factor TFIIA complex subunit Toa1, putative	*Neosartorya fischeri NRRL 181*	1e-05	11
Singlet0352	FE525257	1	0	0	0	0	0	0	0	0	0	-	No significant similarity	-	-	-
Singlet0353	FE525259	1	0	0	0	0	0	0	0	0	0	-	No significant similarity	-	-	-
Singlet0354	FE525260	1	0	0	0	0	0	0	0	0	0	-	No significant similarity	-	-	-
Singlet0355	FE525265	1	0	0	0	0	0	0	0	0	0	ref|XP_001217121.1|	60S ribosomal protein L9-B	*Aspergillus terreus NIH2624*	2e-12	12
Singlet0356	FE525269	1	0	0	0	0	0	0	0	0	0	-	No significant similarity	-	-	-
Singlet0357	FE525270	1	0	0	0	0	0	0	0	0	0	-	No significant similarity	-	-	-
Singlet0358	FE525272	1	0	0	0	0	0	0	0	0	0	ref|XP_001266191.1|	PHD transcription factor (Rum1), putative	*Neosartorya fischeri NRRL 181*	6e-17	1, 11
Singlet0359	FE525273	1	0	0	0	0	0	0	0	0	0	-	No significant similarity	-	-	-
Singlet0360	FE525274	1	0	0	0	0	0	0	0	0	0	ref|XP_001267282.1|	60S ribosomal protein L36	*Neosartorya fischeri NRRL 181*	7e-05	12, 16
Singlet0361	FE525275	1	0	0	0	0	0	0	0	0	0	-	No significant similarity	-	-	-
Singlet0362	FE525281	1	0	0	0	0	0	0	0	0	0	-	No significant similarity	-	-	-
Singlet0363	FE525282	1	0	0	0	0	0	0	0	0	0	-	No significant similarity	-	-	-
Singlet0364	FE525286	1	0	0	0	0	0	0	0	0	0	-	No significant similarity	-	-	-
Singlet0365	FE525287	1	0	0	0	0	0	0	0	0	0	-	No significant similarity	-	-	-
Singlet0366	FE525292	1	0	0	0	0	0	0	0	0	0	-	No significant similarity	-	-	-
Singlet0367	FE525293	1	0	0	0	0	0	0	0	0	0	-	No significant similarity	-	-	-
Singlet0368	FE525296	1	0	0	0	0	0	0	0	0	0	ref|XP_001246341.1|	probable cation-transporting	*Coccidioides immitis*	6e-24	16, 20,

												ATPase	*RS*		30, 34, 42
Singlet0369	FE525297	1	0	0	0	0	0	0	0	0	0	-	No significant similarity	-	-	-
Singlet0370	FE525299	1	0	0	0	0	0	0	0	0	0	-	No significant similarity	-	-	-
Singlet0371	FE525301	1	0	0	0	0	0	0	0	0	0	-	No significant similarity	-	-	-
Singlet0372	FE525304	1	0	0	0	0	0	0	0	0	0	ref|XP_001264792.1|	aldehyde reductase (AKR1), putative	*Neosartorya fischeri NRRL 181*	1e-25	1
Singlet0373	FE525305	1	0	0	0	0	0	0	0	0	0	-	No significant similarity	-	-	-
Singlet0374	FE525306	1	0	0	0	0	0	0	0	0	0	-	No significant similarity	-	-	-
Singlet0375	FE525312	1	0	0	0	0	0	0	0	0	0	-	No significant similarity	-	-	-
Singlet0376	FE525313	1	0	0	0	0	0	0	0	0	0	ref|XP_001261298.1|	sugar transporter	*Neosartorya fischeri NRRL 181*	4e-24	20
Singlet0377	FE525316	1	0	0	0	0	0	0	0	0	0	-	No significant similarity	-	-	-
Singlet0378	FE525319	1	0	0	0	0	0	0	0	0	0	ref|XP_001268304.1|	BolA domain protein	*Aspergillus clavatus NRRL 1*	9e-15	99
Singlet0379	FE525320	1	0	0	0	0	0	0	0	0	0	gb|AAG01549.3|AF291822_1	multidrug resistance protein MDR	*Trichophyton rubrum*	2e-07	20, 32
Singlet0380	FE525321	1	0	0	0	0	0	0	0	0	0	-	No significant similarity	-	-	-
Singlet0381	FE525323	1	0	0	0	0	0	0	0	0	0	-	No significant similarity	-	-	-
Singlet0382	FE525325	1	0	0	0	0	0	0	0	0	0	ref|XP_001209805.1|	2-methylcitrate synthase, mitochondrial precursor	*Aspergillus terreus NIH2624*	2e-19	1, 2
Singlet0383	FE525326	1	0	0	0	0	0	0	0	0	0	gb|EDK06386.1|	40S ribosomal protein S3	*Magnaporthe grisea 70-15*	2e-17	10, 12, 16, 32
Singlet0384	FE525327	1	0	0	0	0	0	0	0	0	0	-	No significant similarity	-	-	-
Singlet0385	FE525328	1	0	0	0	0	0	0	0	0	0	ref|XP_001259371.1|	chorismate mutase	*Neosartorya fischeri NRRL 181*	7e-08	1
Singlet0386	FE525329	1	0	0	0	0	0	0	0	0	0	-	No significant similarity	-	-	-
Singlet0387	FE525330	1	0	0	0	0	0	0	0	0	0	gb|ABB18373.1|	chitobiase	*Coccidioides posadasii*	4e-14	99
Singlet0388	FE525331	1	0	0	0	0	0	0	0	0	0	-	No significant similarity	-	-	-
Singlet0389	FE525333	1	0	0	0	0	0	0	0	0	0	-	No significant similarity	-	-	-
Singlet0390	FE525334	1	0	0	0	0	0	0	0	0	0	ref|XP_001271628.1|	5-methyltetrahydropteroyltriglutamate--homocysteine S-methyltransferase	*Aspergillus clavatus NRRL 1*	9e-05	1
Singlet0391	FE525335	1	0	0	0	0	0	0	0	0	0	ref|XP_001265922.1|	IMP dehydrogenase, putative	*Neosartorya fischeri NRRL 181*	4e-26	1
Singlet0392	FE525338	1	0	0	0	0	0	0	0	0	0	-	No significant similarity	-	-	-
Singlet0393	FE525339	1	0	0	0	0	0	0	0	0	0	-	No significant similarity	-	-	-
Singlet0394	FE525340	1	0	0	0	0	0	0	0	0	0	-	No significant similarity	-	-	-
Singlet0395	FE525341	1	0	0	0	0	0	0	0	0	0	-	No significant similarity	-	-	-
Singlet0396	FE525343	1	0	0	0	0	0	0	0	0	0	-	No significant similarity	-	-	-

Singlet0397	FE525344	1	0	0	0	0	0	0	0	0	0	ref|XP_001239885.1|	60S ribosomal protein L32	*Coccidioides immitis RS*	1e-15	12, 16
Singlet0398	FE525346	1	0	0	0	0	0	0	0	0	0	-	No significant similarity	-	-	-
Singlet0399	FE525347	1	0	0	0	0	0	0	0	0	0	-	No significant similarity	-	-	-
Singlet0400	FE525349	1	0	0	0	0	0	0	0	0	0	-	No significant similarity	-	-	-
Singlet0401	FE525350	1	0	0	0	0	0	0	0	0	0	-	No significant similarity	-	-	-
Singlet0402	FE525351	1	0	0	0	0	0	0	0	0	0	gb|EEQ30360.1|	GTP-binding nuclear protein RAN/TC4	*Microsporum canis CBS 113480*	9e-30	1, 16, 20, 30, 42
Singlet0403	FE525352	1	0	0	0	0	0	0	0	0	0	gb|EAT91215.1|	predicted protein	*Phaeosphaeria nodorum SN15*	5e-09	99
Singlet0404	FE525353	1	0	0	0	0	0	0	0	0	0	-	No significant similarity	-	-	-
Singlet0405	FE525354	1	0	0	0	0	0	0	0	0	0	gb|AAF23950.1|	NTR	*Cercopithecine herpesvirus 12*	4e-09	99
Singlet0406	FE525355	1	0	0	0	0	0	0	0	0	0	-	No significant similarity	-	-	-
Singlet0407	FE525359	1	0	0	0	0	0	0	0	0	0	ref|XP_001214052.1|	GTP-binding protein ypt3	*Aspergillus terreus NIH2624*	1e-05	14, 20
Singlet0408	FE525360	1	0	0	0	0	0	0	0	0	0	-	No significant similarity	-	-	-
Singlet0409	FE525362	1	0	0	0	0	0	0	0	0	0	-	No significant similarity	-	-	-
Singlet0410	FE525364	1	0	0	0	0	0	0	0	0	0	-	No significant similarity	-	-	-
Singlet0411	FE525368	1	0	0	0	0	0	0	0	0	0	ref|XP_001243753.1|	60S ribosomal protein L17	*Coccidioides immitis RS*	4e-24	12
Singlet0412	FE525369	1	0	0	0	0	0	0	0	0	0	ref|XP_001266324.1|	UPF0047 domain protein	*Neosartorya fischeri NRRL 181*	2e-23	99
Singlet0413	FE525370	1	0	0	0	0	0	0	0	0	0	-	No significant similarity	-	-	-
Singlet0414	FE525376	1	0	0	0	0	0	0	0	0	0	-	No significant similarity	-	-	-
Singlet0415	FE525377	1	0	0	0	0	0	0	0	0	0	gb|AAO73810.2|	heat shock protein CLPA	*Paracoccidioides brasiliensis*	7e-06	14, 16, 20, 32
Singlet0416	FE525379	1	0	0	0	0	0	0	0	0	0	-	No significant similarity	-	-	-
Singlet0417	FE525381	1	0	0	0	0	0	0	0	0	0	-	No significant similarity	-	-	-
Singlet0418	FE525382	1	0	0	0	0	0	0	0	0	0	ref|XP_001270964.1|	extracellular thaumatin domain protein, putative	*Aspergillus clavatus NRRL 1*	1e-08	32
Singlet0419	FE525385	1	0	0	0	0	0	0	0	0	0	ref|XP_001246338.1|	eukaryotic translation initiation factor 2 alpha subunit (eIF-2-alpha)	*Coccidioides immitis RS*	1e-18	12, 16, 18, 32
Singlet0420	FE525388	1	0	0	0	0	0	0	0	0	0	ref|XP_001268377.1|	60S ribosomal protein L31e	*Aspergillus clavatus NRRL 1*	4e-27	12
Singlet0421	FE525390	1	0	0	0	0	0	0	0	0	0	-	No significant similarity	-	-	-
Singlet0422	FE525391	1	0	0	0	0	0	0	0	0	0	-	No significant similarity	-	-	-
Singlet0423	FE525392	1	0	0	0	0	0	0	0	0	0	-	No significant similarity	-	-	-
Singlet0424	FE525394	1	0	0	0	0	0	0	0	0	0	gb|ABF13477.1|	DRK1 histidine kinase	*Ajellomyces dermatitidis*	8e-19	14, 30

Singlet0425	FE525395	1	0	0	0	0	0	0	0	0	0	ref|XP_755487.1|	transmembrane domain-containing protein	*Aspergillus fumigatus Af293*	1e-11	99
Singlet0426	FE525399	1	0	0	0	0	0	0	0	0	0	-	No significant similarity	-	-	-
Singlet0427	FE525401	1	0	0	0	0	0	0	0	0	0	ref|XP_001247329.1|	tyrosyl-tRNA synthetase, cytoplasmic	*Coccidioides immitis RS*	9e-06	1, 12, 16
Singlet0428	FE525402	1	0	0	0	0	0	0	0	0	0	ref|XP_753079.1|	long chain fatty alcohol oxidase, putative	*Aspergillus fumigatus Af293*	2e-07	1
Singlet0429	FE525404	1	0	0	0	0	0	0	0	0	0	gb|ABF13597.1|	dermatan-binding protein PA5541	*Propionibacterium acnes*	7e-06	99
Singlet0430	FE525405	1	0	0	0	0	0	0	0	0	0	ref|XP_001209812.1|	malate synthase	*Aspergillus terreus NIH2624*	8e-32	1, 2
Singlet0431	FE525406	1	0	0	0	0	0	0	0	0	0	-	No significant similarity	-	-	-
Singlet0432	FE525407	1	0	0	0	0	0	0	0	0	0	gb|EAL84560.2|	protein Bem1, putative	*Aspergillus fumigatus Af293*	6e-06	1, 14, 30, 42
Singlet0433	FE525409	1	0	0	0	0	0	0	0	0	0	ref|XP_001212958.1|	40S ribosomal protein S8-B	*Aspergillus terreus NIH2624*	7e-09	12
Singlet0434	FE525411	1	0	0	0	0	0	0	0	0	0	-	No significant similarity	-	-	-
Singlet0435	FE525413	1	0	0	0	0	0	0	0	0	0	-	No significant similarity	-	-	-
Singlet0436	FE525414	1	0	0	0	0	0	0	0	0	0	-	No significant similarity	-	-	-
Singlet0437	FE525416	1	0	0	0	0	0	0	0	0	0	-	No significant similarity	-	-	-
Singlet0438	FE525419	1	0	0	0	0	0	0	0	0	0	-	No significant similarity	-	-	-
Singlet0439	FE525420	1	0	0	0	0	0	0	0	0	0	-	No significant similarity	-	-	-
Singlet0440	FE525422	1	0	0	0	0	0	0	0	0	0	ref|XP_001267290.1|	5-methyltetrahydropteroyltriglutamate--homocysteine S-methyltransferase	*Neosartorya fischeri NRRL 181*	6e-34	1
Singlet0441	FE525424	1	0	0	0	0	0	0	0	0	0	-	No significant similarity	-	-	-
Singlet0442	FE525425	1	0	0	0	0	0	0	0	0	0	ref|XP_001271804.1|	cytochrome c peroxidase Ccp1, putative	*Aspergillus clavatus NRRL 1*	4e-37	20, 32, 42
Singlet0443	FE525426	1	0	0	0	0	0	0	0	0	0	ref|XP_001266962.1|	transcription regulator BDF1, putative	*Neosartorya fischeri NRRL 181*	4e-08	11, 43
Singlet0444	FE525428	1	0	0	0	0	0	0	0	0	0	ref|XP_568822.1|	UDP-glucose:glycoprotein glucosyltransferase	*Cryptococcus neoformans var. neoformans JEC21*	9e-38	14
Singlet0445	FE525431	1	0	0	0	0	0	0	0	0	0	-	No significant similarity	-	-	-
Singlet0446	FE525432	1	0	0	0	0	0	0	0	0	0	-	No significant similarity	-	-	-
Singlet0447	FE525433	1	0	0	0	0	0	0	0	0	0	-	No significant similarity	-	-	-
Singlet0448	FE525435	1	0	0	0	0	0	0	0	0	0	-	No significant similarity	-	-	-
Singlet0449	FE525438	1	0	0	0	0	0	0	0	0	0	ref|XP_001215933.1|	asparaginyl-tRNA synthetase	*Aspergillus terreus NIH2624*	2e-50	12, 16
Singlet0450	FE525439	1	0	0	0	0	0	0	0	0	0	-	No significant similarity	-	-	-
Singlet0451	FE525441	1	0	0	0	0	0	0	0	0	0	-	No significant similarity	-	-	-

Singlet0452	FE525442	1	0	0	0	0	0	0	0	0	0	ref|XP_868852.1|	uricase	*Aspergillus nidulans FGSC A4*	6e-35	1
Singlet0453	FE525445	1	0	0	0	0	0	0	0	0	0	-	No significant similarity	-	-	-
Singlet0454	FE525451	1	0	0	0	0	0	0	0	0	0	-	No significant similarity	-	-	-
Singlet0455	FE525454	1	0	0	0	0	0	0	0	0	0	-	No significant similarity	-	-	-
Singlet0456	FE525457	1	0	0	0	0	0	0	0	0	0	-	No significant similarity	-	-	-
Singlet0457	FE525458	1	0	0	0	0	0	0	0	0	0	-	No significant similarity	-	-	-
Singlet0458	FE525462	1	0	0	0	0	0	0	0	0	0	-	No significant similarity	-	-	-
Singlet0459	FE525465	1	0	0	0	0	0	0	0	0	0	-	No significant similarity	-	-	-
Singlet0460	FE525467	1	0	0	0	0	0	0	0	0	0	ref|XP_001245555.1|	eukaryotic translation initiation factor 5A-2	*Coccidioides immitis RS*	2e-05	1, 12, 16, 40
Singlet0461	FE525470	1	0	0	0	0	0	0	0	0	0	-	No significant similarity	-	-	-
Singlet0462	FE525471	1	0	0	0	0	0	0	0	0	0	ref|XP_001258207.1|	short chain dehydrogenase/reductase family	*Neosartorya fischeri NRRL 181*	5e-05	99
Singlet0463	FE525478	1	0	0	0	0	0	0	0	0	0	-	No significant similarity	-	-	-
Singlet0464	FE525479	1	0	0	0	0	0	0	0	0	0	-	No significant similarity	-	-	-
Singlet0465	FE525482	1	0	0	0	0	0	0	0	0	0	ref|XP_001260275.1|	serine/threonine protein kinase (Ark1), putative	*Neosartorya fischeri NRRL 181*	8e-35	14, 30, 42
Singlet0466	FE525485	1	0	0	0	0	0	0	0	0	0	-	No significant similarity	-	-	-
Singlet0467	FE525487	1	0	0	0	0	0	0	0	0	0	-	No significant similarity	-	-	-
Singlet0468	FE525488	1	0	0	0	0	0	0	0	0	0	-	No significant similarity	-	-	-
Singlet0469	FE525489	1	0	0	0	0	0	0	0	0	0	ref|XP_001240451.1|	aspartyl aminopeptidase, putative	*Coccidioides immitis RS*	1e-08	14, 16
Singlet0470	FE525492	1	0	0	0	0	0	0	0	0	0	-	No significant similarity	-	-	-
Singlet0471	FE525493	1	0	0	0	0	0	0	0	0	0	-	No significant similarity	-	-	-
Singlet0472	FE525494	1	0	0	0	0	0	0	0	0	0	-	No significant similarity	-	-	-
Singlet0473	FE525495	1	0	0	0	0	0	0	0	0	0	-	No significant similarity	-	-	-
Singlet0474	FE525496	1	0	0	0	0	0	0	0	0	0	-	No significant similarity	-	-	-
Singlet0475	FE525498	1	0	0	0	0	0	0	0	0	0	-	No significant similarity	-	-	-
Singlet0476	FE525499	1	0	0	0	0	0	0	0	0	0	ref|XP_749092.1|	integral peroxisomal membrane protein	*Aspergillus fumigatus Af293*	4e-24	42
Singlet0477	FE525500	1	0	0	0	0	0	0	0	0	0	-	No significant similarity	-	-	-
Singlet0478	FE525501	1	0	0	0	0	0	0	0	0	0	ref|XP_001265155.1|	COPII-coated vesicle membrane protein Erv46, putative	*Neosartorya fischeri NRRL 181*	9e-33	16, 20
Singlet0479	FE525503	1	0	0	0	0	0	0	0	0	0	ref|XP_001241599.1|	predicted protein	*Coccidioides immitis RS*	6e-11	99
Singlet0480	FE525505	1	0	0	0	0	0	0	0	0	0	-	No significant similarity	-	-	-
Singlet0481	FE525509	1	0	0	0	0	0	0	0	0	0	ref|XP_001269838.1|	6-phosphogluconolactonase	*Aspergillus clavatus NRRL 1*	2e-34	2, 11
Singlet0482	FE525514	1	0	0	0	0	0	0	0	0	0	-	No significant similarity	-	-	-
Singlet0483	FE525516	1	0	0	0	0	0	0	0	0	0	gb|AAN63566.1|AF429823_1	septin 3	*Coccidioides immitis*	5e-08	1, 10, 16, 43

Singlet0484	FE525520	1	0	0	0	0	0	0	0	0	0	-	No significant similarity	-	-	-
Singlet0485	FE525522	1	0	0	0	0	0	0	0	0	0	-	No significant similarity	-	-	-
Singlet0486	FE525524	1	0	0	0	0	0	0	0	0	0	-	No significant similarity	-	-	-
Singlet0487	FE525526	1	0	0	0	0	0	0	0	0	0	-	No significant similarity	-	-	-
Singlet0488	FE525527	1	0	0	0	0	0	0	0	0	0	ref|XP_660794.1|	hypothetical protein AN3190.2	*Aspergillus nidulans FGSC A4*	7e-23	99
Singlet0489	FE525528	1	0	0	0	0	0	0	0	0	0	-	No significant similarity	-	-	-
Singlet0490	FE525529	1	0	0	0	0	0	0	0	0	0	-	No significant similarity	-	-	-
Singlet0491	FE525531	1	0	0	0	0	0	0	0	0	0	-	No significant similarity	-	-	-
Singlet0492	FE525532	1	0	0	0	0	0	0	0	0	0	-	No significant similarity	-	-	-
Singlet0493	FE525533	1	0	0	0	0	0	0	0	0	0	-	No significant similarity	-	-	-
Singlet0494	FE525536	1	0	0	0	0	0	0	0	0	0	ref|XP_001242406.1|	O-acetylhomoserine	*Coccidioides immitis RS*	5e-21	1, 16
Singlet0495	FE525537	1	0	0	0	0	0	0	0	0	0	-	No significant similarity	-	-	-
Singlet0496	FE525538	1	0	0	0	0	0	0	0	0	0	-	No significant similarity	-	-	-
Singlet0497	FE525540	1	0	0	0	0	0	0	0	0	0	-	No significant similarity	-	-	-
Singlet0498	FE525541	1	0	0	0	0	0	0	0	0	0	-	No significant similarity	-	-	-
Singlet0499	FE525542	1	0	0	0	0	0	0	0	0	0	-	No significant similarity	-	-	-
Singlet0500	FE525546	1	0	0	0	0	0	0	0	0	0	-	No significant similarity	-	-	-
Singlet0501	FE525547	1	0	0	0	0	0	0	0	0	0	ref|XP_001247210.1|	NAD-specific glutamate dehydrogenase (NAD-GDH)	*Coccidioides immitis RS*	3e-34	1, 2, 16, 42
Singlet0502	FE525548	1	0	0	0	0	0	0	0	0	0	gb|ABG74714.1|	Ac1147-like protein	*Diaphorina citri*	4e-21	99
Singlet0503	FE525550	1	0	0	0	0	0	0	0	0	0	-	No significant similarity	-	-	-
Singlet0504	FE525551	1	0	0	0	0	0	0	0	0	0	-	No significant similarity	-	-	-
Singlet0505	FE525552	1	0	0	0	0	0	0	0	0	0	-	No significant similarity	-	-	-
Singlet0506	FE525553	1	0	0	0	0	0	0	0	0	0	ref|XP_001266925.1|	alpha-aminoadipate reductase large subunit, putative	*Neosartorya fischeri NRRL 181*	4e-11	1
Singlet0507	FE525557	1	0	0	0	0	0	0	0	0	0	-	No significant similarity	-	-	-
Singlet0508	FE525558	1	0	0	0	0	0	0	0	0	0	-	No significant similarity	-	-	-
Singlet0509	FE525559	1	0	0	0	0	0	0	0	0	0	-	No significant similarity	-	-	-
Singlet0510	FE525563	1	0	0	0	0	0	0	0	0	0	-	No significant similarity	-	-	-
Singlet0511	FE525564	1	0	0	0	0	0	0	0	0	0	-	No significant similarity	-	-	-
Singlet0512	FE525566	1	0	0	0	0	0	0	0	0	0	-	No significant similarity	-	-	-
Singlet0513	FE525568	1	0	0	0	0	0	0	0	0	0	ref|XP_001276134.1|	vacuolar aspartyl aminopeptidase Lap4, putative	*Aspergillus clavatus NRRL 1*	1e-10	14
Singlet0514	FE525569	1	0	0	0	0	0	0	0	0	0	gb|AAB05810.1|	super cysteine rich protein; SCRP	*Homo sapiens*	9e-15	11, 16
Singlet0515	FE525570	1	0	0	0	0	0	0	0	0	0	ref|XP_001239110.1|	hypothetical protein CIMG_10132	*Coccidioides immitis RS*	1e-16	99
Singlet0516	FE525571	1	0	0	0	0	0	0	0	0	0	-	No significant similarity	-	-	-
Singlet0517	FE525574	1	0	0	0	0	0	0	0	0	0	-	No significant similarity	-	-	-
Singlet0518	FE525575	1	0	0	0	0	0	0	0	0	0	-	No significant similarity	-	-	-

Singlet0519	FE525576	1	0	0	0	0	0	0	0	0	0	-	No significant similarity	-	-	-
Singlet0520	FE525577	1	0	0	0	0	0	0	0	0	0	-	No significant similarity	-	-	-
Singlet0521	FE525578	1	0	0	0	0	0	0	0	0	0	-	No significant similarity	-	-	-
Singlet0522	FE525620	0	1	0	0	0	0	0	0	0	0	-	No significant similarity	-	-	-
Singlet0523	FE525598	0	1	0	0	0	0	0	0	0	0	-	No significant similarity	-	-	-
Singlet0524	FE525624	0	1	0	0	0	0	0	0	0	0	ref|XP_001267191.1|	DNA mismatch repair protein Msh6, putative	*Neosartorya fischeri NRRL 181*	2e-33	1, 10, 16
Singlet0525	FE525626	0	1	0	0	0	0	0	0	0	0	ref|XP_367322.1|	hypothetical protein MG07247.4	*Magnaporthe grisea 70-15*	2e-06	99
Singlet0526	FE525628	0	1	0	0	0	0	0	0	0	0	ref|XP_001262368.1|	DEAD helicases superfamily protein (Aquarius), putative	*Neosartorya fischeri NRRL 181*	1e-19	1, 10, 11, 12, 16
Singlet0527	FE525640	0	1	0	0	0	0	0	0	0	0	ref|XP_380291.1|	hypothetical protein FG00115.1	*Gibberella zeae PH-1*	5e-06	99
Singlet0528	FE525647	0	1	0	0	0	0	0	0	0	0	-	No significant similarity	-	-	-
Singlet0529	FE525657	0	1	0	0	0	0	0	0	0	0	ref|XP_001275274.1|	PB1 domain protein, putative	*Aspergillus clavatus NRRL 1*	8e-06	99
Singlet0530	FE525677	0	1	0	0	0	0	0	0	0	0	-	No significant similarity	-	-	-
Singlet0531	FE525680	0	1	0	0	0	0	0	0	0	0	-	No significant similarity	-	-	-
Singlet0532	FE525683	0	0	1	0	0	0	0	0	0	0	ref|XP_001272343.1|	indoleamine 2,3-dioxygenase family protein	*Aspergillus clavatus NRRL 1*	1e-27	1
Singlet0533	FE525684	0	0	1	0	0	0	0	0	0	0	-	No significant similarity	-	-	-
Singlet0534	FE525685	0	0	1	0	0	0	0	0	0	0	-	No significant similarity	-	-	-
Singlet0535	FE525686	0	0	1	0	0	0	0	0	0	0	-	No significant similarity	-	-	-
Singlet0536	FE525687	0	0	1	0	0	0	0	0	0	0	ref|XP_001269335.1|	AAA family ATPase/60S ribosome export protein Rix7, putative	*Aspergillus clavatus NRRL 1*	2e-12	1, 12, 16, 20
Singlet0537	FE525688	0	0	1	0	0	0	0	0	0	0	-	No significant similarity	-	-	
Singlet0538	FE525690	0	0	1	0	0	0	0	0	0	0	ref|XP_001262277.1|	alpha/beta fold family hydrolase, putative	*Neosartorya fischeri NRRL 181*	2e-06	1
Singlet0539	FE525691	0	0	1	0	0	0	0	0	0	0	ref|XP_001272129.1|	LYR family protein	*Aspergillus clavatus NRRL 1*	2e-07	99
Singlet0540	FE525692	0	0	1	0	0	0	0	0	0	0	ref|XP_001228475.1|	40s ribosomal protein S5	*Chaetomium globosum CBS 148.51*	2e-46	12, 16
Singlet0541	FE525693	0	0	1	0	0	0	0	0	0	0	ref|XP_001268941.1|	methyltransferase small domain protein	*Aspergillus clavatus NRRL 1*	2e-13	16, 32
Singlet0542	FE525694	0	0	1	0	0	0	0	0	0	0	-	No significant similarity	-	-	-
Singlet0543	FE525695	0	0	1	0	0	0	0	0	0	0	-	No significant similarity	-	-	-
Singlet0544	FE525696	0	0	1	0	0	0	0	0	0	0	ref|XP_001264172.1|	3-isopropylmalate dehydrogenase Leu2A	*Neosartorya fischeri NRRL 181*	6e-36	1, 2, 16, 20
Singlet0545	FE525697	0	0	1	0	0	0	0	0	0	0	-	No significant similarity	-	-	-
Singlet0546	FE525700	0	0	1	0	0	0	0	0	0	0	-	No significant similarity	-	-	-
Singlet0547	FE525702	0	0	1	0	0	0	0	0	0	0	-	No significant similarity	-	-	-

Singlet0548	FE525703	0	0	1	0	0	0	0	0	0	0	gb|AAL78196.1|AF126048_1	UDP-N-acetylglucosamine:dolichyl phosphate N-acetylglucosamine-1-phosphate transferase; GPT	*Aspergillus niger*	5e-63	1, 10, 14
Singlet0549	FE525706	0	0	1	0	0	0	0	0	0	0	-	No significant similarity	-	-	-
Singlet0550	FE525708	0	0	1	0	0	0	0	0	0	0	ref|XP_001244644.1|	hypothetical protein CIMG_04085	*Coccidioides immitis RS*	4e-04	99
Singlet0551	FE525709	0	0	1	0	0	0	0	0	0	0	-	No significant similarity	-	-	-
Singlet0552	FE525710	0	0	1	0	0	0	0	0	0	0	ref|XP_001213511.1|	type II proteins geranylgeranyltransferase beta subunit	*Aspergillus terreus NIH2624*	4e-35	14
Singlet0553	FE525711	0	0	1	0	0	0	0	0	0	0	ref|XP_001247006.1|	hypothetical protein CIMG_00777	*Coccidioides immitis RS*	1e-08	99
Singlet0554	FE525713	0	0	1	0	0	0	0	0	0	0	-	No significant similarity	-	-	-
Singlet0555	FE525714	0	0	1	0	0	0	0	0	0	0	ref|XP_748019.1|	pyruvate dehydrogenase kinase, putative	*Aspergillus fumigatus Af293*	5e-56	1, 2, 14, 16, 18
Singlet0556	FE525717	0	0	1	0	0	0	0	0	0	0	dbj|BAE62191.1|	unnamed protein product	*Aspergillus oryzae*	2e-16	99
Singlet0557	FE525720	0	0	1	0	0	0	0	0	0	0	-	No significant similarity	-	-	-
Singlet0558	FE525722	0	0	1	0	0	0	0	0	0	0	ref|XP_001260869.1|	kinase activator (Atg17), putative	*Neosartorya fischeri NRRL 181*	3e-09	18, 40, 42, 43
Singlet0559	FE525725	0	0	1	0	0	0	0	0	0	0	ref|XP_001258025.1|	VHS domain protein	*Neosartorya fischeri NRRL 181*	1e-06	99
Singlet0560	FE525726	0	0	1	0	0	0	0	0	0	0	-	No significant similarity	-	-	-
Singlet0561	FE525728	0	0	1	0	0	0	0	0	0	0	-	No significant similarity	-	-	-
Singlet0562	FE525730	0	0	1	0	0	0	0	0	0	0	ref|XP_001248599.1|	eukaryotic translation initiation factor 3 subunit 7 homolog	*Coccidioides immitis RS*	1e-24	11, 12, 16
Singlet0563	FE525732	0	0	1	0	0	0	0	0	0	0	-	No significant similarity	-	-	-
Singlet0564	FE525733	0	0	1	0	0	0	0	0	0	0	gb|EDK04135.1|	conserved hypothetical protein	*Magnaporthe grisea 70-15*	3e-04	99
Singlet0565	FE525738	0	0	1	0	0	0	0	0	0	0	gb|ABB18373.1|	chitobiase	*Coccidioides posadasii*	4e-50	99
Singlet0566	FE525740	0	0	1	0	0	0	0	0	0	0	ref|XP_001268837.1|	fibrillarin	*Aspergillus clavatus NRRL 1*	3e-36	1, 11, 12, 16
Singlet0567	FE525741	0	0	1	0	0	0	0	0	0	0	-	No significant similarity	-	-	-
Singlet0568	FE525743	0	0	1	0	0	0	0	0	0	0	ref|XP_001276345.1|	histone H2A	*Aspergillus clavatus NRRL 1*	4e-19	10, 11, 16, 42
Singlet0569	FE525744	0	0	1	0	0	0	0	0	0	0	ref|XP_001261116.1|	cyanate hydratase, putative	*Neosartorya fischeri NRRL 181*	9e-21	1
Singlet0570	FE525748	0	0	1	0	0	0	0	0	0	0	ref|XP_001266066.1|	60S ribosomal protein L23	*Neosartorya fischeri NRRL 181*	2e-34	12
Singlet0571	FE525750	0	0	1	0	0	0	0	0	0	0	ref|XP_001274417.1|	oxidoreductase	*Aspergillus clavatus NRRL 1*	4e-44	1
Singlet0572	FE525751	0	0	1	0	0	0	0	0	0	0	-	No significant similarity	-	-	-
Singlet0573	FE525752	0	0	1	0	0	0	0	0	0	0	ref|XP_001272850.1|	mitochondrial ATPase inhibitor,	*Aspergillus clavatus*	1e-05	2, 18,

												putative	*NRRL 1*		20
Singlet0574	FE525753	0	0	1	0	0	0	0	0	0	0	ref|XP_001274092.1|	cysteinyl-tRNA synthetase	*Aspergillus clavatus NRRL 1*	4e-51	1, 12, 16
Singlet0575	FE525754	0	0	1	0	0	0	0	0	0	0	-	No significant similarity	-	-	-
Singlet0576	FE525756	0	0	1	0	0	0	0	0	0	0	gb|EAL84586.2|	DNA repair protein (Tof1), putative	*Aspergillus fumigatus Af293*	1e-24	10
Singlet0577	FE525758	0	0	1	0	0	0	0	0	0	0	-	No significant similarity	-	-	-
Singlet0578	FE525760	0	0	1	0	0	0	0	0	0	0	-	No significant similarity	-	-	-
Singlet0579	FE525761	0	0	1	0	0	0	0	0	0	0	ref|XP_752890.1|	conserved hypothetical protein	*Aspergillus fumigatus Af293*	4e-13	99
Singlet0580	FE525762	0	0	1	0	0	0	0	0	0	0	ref|XP_750435.1|	protein phosphatase 2C family protein	*Aspergillus fumigatus Af293*	3e-30	1, 10, 14, 16, 18
Singlet0581	FE525763	0	0	1	0	0	0	0	0	0	0	ref|XP_001244432.1|	hypothetical protein CIMG_03873	*Coccidioides immitis RS*	8e-56	99
Singlet0582	FE525764	0	0	1	0	0	0	0	0	0	0	ref|XP_001272790.1|	small nucleolar ribonucleoprotein complex subunit, putative	*Aspergillus clavatus NRRL 1*	4e-14	1, 11, 14, 16
Singlet0583	FE525766	0	0	1	0	0	0	0	0	0	0	ref|NP_983024.1|	ABR078Cp	*Ashbya gossypii ATCC 10895*	6e-09	99
Singlet0584	FE525767	0	0	1	0	0	0	0	0	0	0	-	No significant similarity	-	-	-
Singlet0585	FE525768	0	0	1	0	0	0	0	0	0	0	ref|XP_001266177.1|	mitochondrial large ribosomal subunit protein L1, putative	*Neosartorya fischeri NRRL 181*	3e-22	99
Singlet0586	FE525769	0	0	1	0	0	0	0	0	0	0	emb|CAK42954.1|	unnamed protein product	*Aspergillus niger*	4e-24	99
Singlet0587	FE525770	0	0	1	0	0	0	0	0	0	0	ref|XP_001266773.1|	DNA repair protein Rad50	*Neosartorya fischeri NRRL 181*	1e-19	1, 10, 16, 32, 42
Singlet0588	FE525772	0	0	1	0	0	0	0	0	0	0	-	No significant similarity	-	-	-
Singlet0589	FE525773	0	0	1	0	0	0	0	0	0	0	-	No significant similarity	-	-	-
Singlet0590	FE525777	0	0	1	0	0	0	0	0	0	0	-	No significant similarity	-	-	-
Singlet0591	FE525779	0	0	1	0	0	0	0	0	0	0	ref|XP_748881.1|	NACHT domain protein, putative	*Aspergillus fumigatus Af293*	6e-04	99
Singlet0592	FE525780	0	0	1	0	0	0	0	0	0	0	-	No significant similarity	-	-	-
Singlet0593	FE525781	0	0	1	0	0	0	0	0	0	0	-	No significant similarity	-	-	-
Singlet0594	FE525782	0	0	1	0	0	0	0	0	0	0	-	No significant similarity	-	-	-
Singlet0595	FE525783	0	0	0	1	0	0	0	0	0	0	-	No significant similarity	-	-	-
Singlet0596	FE525784	0	0	0	1	0	0	0	0	0	0	ref|XP_001246947.1|	glycolipid 2-alpha-mannosyltransferase	*Coccidioides immitis RS*	2e-06	1, 42
Singlet0597	FE525785	0	0	0	1	0	0	0	0	0	0	ref|XP_868845.1|	hypothetical protein AN9463.2	*Aspergillus nidulans FGSC A4*	8e-04	99
Singlet0598	FE525787	0	0	0	1	0	0	0	0	0	0	-	No significant similarity	-	-	-
Singlet0599	FE525789	0	0	0	1	0	0	0	0	0	0	ref|XP_001268953.1|	regulator of nonsense transcripts, putative	*Aspergillus clavatus NRRL 1*	5e-25	1, 12
Singlet0600	FE525790	0	0	0	1	0	0	0	0	0	0	-	No significant similarity	-	-	-
Singlet0601	FE525797	0	0	0	1	0	0	0	0	0	0	ref|XP_001268331.1|	proteasome regulatory particle	*Aspergillus clavatus*	1e-17	14, 16,

													subunit (RpnI), putative	*NRRL 1*		18, 32
Singlet0602	FE525802	0	0	0	1	0	0	0	0	0	0	ref|XP_001274718.1|	amino acid permease, putative	*Aspergillus clavatus NRRL 1*	3e-12	20
Singlet0603	FE525807	0	0	0	1	0	0	0	0	0	0	ref|XP_749497.1|	Ras GTPase activating protein, putative	*Aspergillus fumigatus Af293*	3e-04	18, 30
Singlet0604	FE525811	0	0	0	1	0	0	0	0	0	0	-	No significant similarity	-	-	-
Singlet0605	FE525816	0	0	0	1	0	0	0	0	0	0	ref|XP_001212104.1|	serine/threonine-protein kinase ssp1	*Aspergillus terreus NIH2624*	1e-03	1, 10, 14, 30, 32, 43
Singlet0606	FE525822	0	0	0	1	0	0	0	0	0	0	ref|XP_001267336.1|	outer mitochondrial membrane protein porin	*Neosartorya fischeri NRRL 181*	8e-41	2, 16, 20, 40, 42
Singlet0607	FE525829	0	0	0	1	0	0	0	0	0	0	-	No significant similarity	-	-	-
Singlet0608	FE525832	0	0	0	1	0	0	0	0	0	0	ref|XP_001265914.1|	sugar transporter, putative	*Neosartorya fischeri NRRL 181*	4e-21	20
Singlet0609	FE525836	0	0	0	1	0	0	0	0	0	0	-	No significant similarity	-	-	-
Singlet0610	FE525843	0	0	0	1	0	0	0	0	0	0	ref|XP_755086.1|	prolidase pepP, putative	*Aspergillus fumigatus Af293*	1e-40	14
Singlet0611	FE525845	0	0	0	1	0	0	0	0	0	0	ref|XP_001271354.1|	glutamate-cysteine ligase Gcs1, putative	*Aspergillus clavatus NRRL 1*	8e-14	1, 32
Singlet0612	FE525852	0	0	0	1	0	0	0	0	0	0	-	No significant similarity	-	-	-
Singlet0613	FE525858	0	0	0	1	0	0	0	0	0	0	ref|XP_001259741.1|	ABC transporter, putative	*Neosartorya fischeri NRRL 181*	1e-22	16, 20
Singlet0614	FE525863	0	0	0	1	0	0	0	0	0	0	-	No significant similarity	-	-	-
Singlet0615	FE525871	0	0	0	1	0	0	0	0	0	0	-	No significant similarity	-	-	-
Singlet0616	FE525874	0	0	0	1	0	0	0	0	0	0	ref|XP_001248278.1|	hypothetical protein CIMG_02049	*Coccidioides immitis RS*	1e-10	99
Singlet0617	FE525876	0	0	0	1	0	0	0	0	0	0	ref|XP_001262831.1|	conserved hypothetical protein	*Neosartorya fischeri NRRL 181*	8e-06	99
Singlet0618	FE525891	0	0	0	1	0	0	0	0	0	0	-	No significant similarity	-	-	-
Singlet0619	FE525892	0	0	0	1	0	0	0	0	0	0	-	No significant similarity	-	-	-
Singlet0620	FE525896	0	0	0	1	0	0	0	0	0	0	gb|ABK60177.1|	putative reverse transcriptase	*Zingiber officinale*	1e-03	99
Singlet0621	FE525901	0	0	0	1	0	0	0	0	0	0	-	No significant similarity	-	-	-
Singlet0622	FE525907	0	0	0	1	0	0	0	0	0	0	ref|ZP_00344940.1|	COG0458: Carbamoylphosphate synthase large subunit (split gene in MJ)	*Nostoc punctiforme PCC 73102*	7e-15	1, 16
Singlet0623	FE525915	0	0	0	1	0	0	0	0	0	0	-	No significant similarity	-	-	-
Singlet0624	FE525920	0	0	0	1	0	0	0	0	0	0	gb|ABF82266.1|	heat shock protein 30	*Penicillium marneffei*	2e-11	32, 34
Singlet0625	FE525921	0	0	0	1	0	0	0	0	0	0	ref|XP_001264679.1|	conserved hypothetical protein	*Neosartorya fischeri NRRL 181*	4e-31	99
Singlet0626	FE525936	0	0	0	1	0	0	0	0	0	0	-	No significant similarity	-	-	-
Singlet0627	FE525946	0	0	0	1	0	0	0	0	0	0	-	No significant similarity	-	-	-
Singlet0628	FE525952	0	0	0	1	0	0	0	0	0	0	ref|XP_001057319.1|	PREDICTED: hypothetical protein	*Rattus norvegicus*	7e-07	99

Singlet0629	FE525961	0	0	0	1	0	0	0	0	0	0	-	No significant similarity	-	-	-
Singlet0630	FE525965	0	0	0	1	0	0	0	0	0	0	-	No significant similarity	-	-	-
Singlet0631	FE525966	0	0	0	1	0	0	0	0	0	0	-	No significant similarity	-	-	-
Singlet0632	FE525973	0	0	0	1	0	0	0	0	0	0	-	No significant similarity	-	-	-
Singlet0633	FE525976	0	0	0	1	0	0	0	0	0	0	-	No significant similarity	-	-	-
Singlet0634	FE525982	0	0	0	1	0	0	0	0	0	0	-	No significant similarity	-	-	-
Singlet0635	FE525986	0	0	0	1	0	0	0	0	0	0	-	No significant similarity	-	-	-
Singlet0636	FE526003	0	0	0	1	0	0	0	0	0	0	-	No significant similarity	-	-	-
Singlet0637	FE526004	0	0	0	1	0	0	0	0	0	0	gb|ABG67901.1|	phospholipase B	*Trichophyton rubrum*	4e-19	1
Singlet0638	FE526010	0	0	0	1	0	0	0	0	0	0	-	No significant similarity	-	-	-
Singlet0639	FE526029	0	0	0	1	0	0	0	0	0	0	emb|CAM14118.1|	novel protein containing SEA domains	*Danio rerio*	1e-4	99
Singlet0640	FE526033	0	0	0	0	1	0	0	0	0	0	ref|XP_001271726.1|	tyrosyl-tRNA synthetase, mitochondrial precursor (tyrosine--tRNA ligase) (tyrrs)	*Aspergillus clavatus NRRL 1*	1e-27	12, 16
Singlet0641	FE526042	0	0	0	0	1	0	0	0	0	0	-	No significant similarity	-	-	-
Singlet0642	FE526051	0	0	0	0	1	0	0	0	0	0	ref|XP_001260474.1|	oxidoreductase, 2-nitropropane dioxygenase family, putative	*Neosartorya fischeri NRRL 181*	2e-49	1, 16, 20
Singlet0643	FE526052	0	0	0	0	1	0	0	0	0	0	ref|XP_751914.1|	cytochrome c peroxidase, putative	*Aspergillus fumigatus Af293*	3e-04	20, 32, 42
Singlet0644	FE526054	0	0	0	0	1	0	0	0	0	0	gb|EEQ32071.1|	glucose-regulated protein, Hsp70 protein	*Microsporum canis CBS 113480*	2e-58	1, 14, 16
Singlet0645	FE526056	0	0	0	0	1	0	0	0	0	0	-	No significant similarity	-	-	-
Singlet0646	FE526058	0	0	0	0	1	0	0	0	0	0	-	No significant similarity	-	-	-
Singlet0647	FE526059	0	0	0	0	1	0	0	0	0	0	-	No significant similarity	-	-	-
Singlet0648	FE526060	0	0	0	0	1	0	0	0	0	0	ref|XP_001257997.1|	NADH-ubiquinone oxidoreductase 304 kDa subunit precursor	*Neosartorya fischeri NRRL 181*	5e-67	2, 20
Singlet0649	FE526063	0	0	0	0	1	0	0	0	0	0	-	No significant similarity	-	-	-
Singlet0650	FE526071	0	0	0	0	1	0	0	0	0	0	-	No significant similarity	-	-	-
Singlet0651	FE526075	0	0	0	0	1	0	0	0	0	0	-	No significant similarity	-	-	-
Singlet0652	FE526088	0	0	0	0	1	0	0	0	0	0	ref|XP_001272465.1|	MFS multidrug transporter, putative	*Aspergillus clavatus NRRL 1*	5e-23	20, 32
Singlet0653	FE526091	0	0	0	0	1	0	0	0	0	0	ref|XP_001240305.1|	hypothetical protein CIMG_07468	*Coccidioides immitis RS*	2e-32	99
Singlet0654	FE526093	0	0	0	0	1	0	0	0	0	0	ref|XP_001239696.1|	predicted protein	*Coccidioides immitis RS*	1e-07	99
Singlet0655	FE526097	0	0	0	0	1	0	0	0	0	0	gb|ABM92787.1|	phytase	*Aspergillus oryzae*	4e-08	1
Singlet0656	FE526098	0	0	0	0	1	0	0	0	0	0	-	No significant similarity	-	-	-
Singlet0657	FE526109	0	0	0	0	1	0	0	0	0	0	ref|XP_001248014.1|	predicted protein	*Coccidioides immitis RS*	7e-04	99
Singlet0658	FE526111	0	0	0	0	1	0	0	0	0	0	-	No significant similarity	-	-	-
Singlet0659	FE526113	0	0	0	0	1	0	0	0	0	0	-	No significant similarity	-	-	-

Singlet0660	FE526118	0	0	0	0	1	0	0	0	0	0	-	No significant similarity	-	-	-
Singlet0661	FE526122	0	0	0	0	1	0	0	0	0	0	-	No significant similarity	-	-	-
Singlet0662	FE526123	0	0	0	0	1	0	0	0	0	0	ref|XP_001239221.1|	GTP-binding nuclear protein GSP1/Ran	*Coccidioides immitis RS*	4e-25	1, 11, 20, 42
Singlet0663	FE526126	0	0	0	0	1	0	0	0	0	0	-	No significant similarity	-	-	-
Singlet0664	FE526128	0	0	0	0	1	0	0	0	0	0	ref|XP_001276376.1|	bZIP transcription factor JlbA/IDI-4	*Aspergillus clavatus NRRL 1*	8e-05	11, 16
Singlet0665	FE526129	0	0	0	0	1	0	0	0	0	0	-	No significant similarity	-	-	-
Singlet0666	FE526137	0	0	0	0	1	0	0	0	0	0	-	No significant similarity	-	-	-
Singlet0667	FE526138	0	0	0	0	1	0	0	0	0	0	-	No significant similarity	-	-	-
Singlet0668	FE526139	0	0	0	0	1	0	0	0	0	0	-	No significant similarity	-	-	-
Singlet0669	FE526141	0	0	0	0	1	0	0	0	0	0	-	No significant similarity	-	-	-
Singlet0670	FE526202	0	0	0	0	0	1	0	0	0	0	-	No significant similarity	-	-	-
Singlet0671	FE526227	0	0	0	0	0	1	0	0	0	0	ref|XP_001262830.1|	sexual development protein EsdC, putative	*Neosartorya fischeri NRRL 181*	2e-11	99
Singlet0672	FE526317	0	0	0	0	0	0	1	0	0	0	-	No significant similarity	-	-	-
Singlet0673	FE526322	0	0	0	0	0	0	1	0	0	0	-	No significant similarity	-	-	-
Singlet0674	FE526329	0	0	0	0	0	0	1	0	0	0	ref|XP_001245309.1|	hypothetical protein CIMG_04750	*Coccidioides immitis RS*	9e-07	99
Singlet0675	FE526335	0	0	0	0	0	0	1	0	0	0	ref|YP_270051.1|	ISCps6, transposase	*Colwellia psychrerythraea 34H*	2e-11	99
Singlet0676	FE526336	0	0	0	0	0	0	1	0	0	0	-	No significant similarity	-	-	-
Singlet0677	FE526340	0	0	0	0	0	0	1	0	0	0	-	No significant similarity	-	-	-
Singlet0678	FE526342	0	0	0	0	0	0	1	0	0	0	ref|XP_752247.1|	cytochrome P450 phenylacetate hydroxylase, putative	*Aspergillus fumigatus Af293*	2e-18	1, 16, 20, 32
Singlet0679	FE526344	0	0	0	0	0	0	1	0	0	0	-	No significant similarity	-	-	-
Singlet0680	FE526346	0	0	0	0	0	0	1	0	0	0	gb|AAZ32401.1|	peptide transporter PTR2A	*Hebeloma cylindrosporum*	1e-11	20
Singlet0681	FE526353	0	0	0	0	0	0	1	0	0	0	ref|XP_001244970.1|	glucosamine-6-phosphate deaminase	*Coccidioides immitis RS*	8e-45	1, 2
Singlet0682	FE526356	0	0	0	0	0	0	1	0	0	0	gb|ABL84984.1|	metalloprotease Mep3	*Trichophyton equinum*	8e-41	14
Singlet0683	FE526360	0	0	0	0	0	0	1	0	0	0	ref|XP_001244970.1|	glucosamine-6-phosphate deaminase	*Coccidioides immitis RS*	9e-31	1, 2
Singlet0684	FE526366	0	0	0	0	0	0	1	0	0	0	ref|XP_754004.1|	MFS transporter, putative	*Aspergillus fumigatus Af293*	4e-35	20
Singlet0685	FE526367	0	0	0	0	0	0	1	0	0	0	-	No significant similarity	-	-	-
Singlet0686	FE526369	0	0	0	0	0	0	1	0	0	0	ref|XP_001270309.1|	benzoate 4-monooxygenase cytochrome P450	*Aspergillus clavatus NRRL 1*	3e-35	1
Singlet0687	FE526372	0	0	0	0	0	0	1	0	0	0	-	No significant similarity	-	-	-
Singlet0688	FE526376	0	0	0	0	0	0	1	0	0	0	ref|XP_001266791.1|	conserved lysine-rich protein, putative	*Neosartorya fischeri NRRL 181*	8e-04	99

Singlet0689	FE526377	0	0	0	0	0	0	1	0	0	0	-	No significant similarity	-	-	-
Singlet0690	FE526396	0	0	0	0	0	0	1	0	0	0	gb|AAS45677.1|	subtilisin-like protease SUB5	*Trichophyton verrucosum*	3e-28	14, 32, 43
Singlet0691	FE526402	0	0	0	0	0	0	1	0	0	0	ref|XP_001229086.1|	hypothetical protein CHGG_02570	*Chaetomium globosum CBS 148.51*	1e-03	99
Singlet0692	FE526403	0	0	0	0	0	0	1	0	0	0	-	No significant similarity	-	-	-
Singlet0693	FE526406	0	0	0	0	0	0	1	0	0	0	ref|YP_080386.1|	oxalate decarboxylase	*Bacillus licheniformis ATCC 14580*	2e-09	1, 2, 32
Singlet0694	FE526410	0	0	0	0	0	0	1	0	0	0	-	No significant similarity	-	-	-
Singlet0695	FE526415	0	0	0	0	0	0	1	0	0	0	ref|XP_001265751.1|	phytanoyl-CoA dioxygenase family protein	*Neosartorya fischeri NRRL 181*	1e-18	99
Singlet0696	FE526416	0	0	0	0	0	0	1	0	0	0	emb|CAJ04955.1|	hypothetical protein, unknown function	*Leishmania major*	7e-07	99
Singlet0697	FE526418	0	0	0	0	0	0	1	0	0	0	ref|XP_001262224.1|	K+/H+ antiporter, putative	*Neosartorya fischeri NRRL 181*	4e-41	20, 34
Singlet0698	FE526419	0	0	0	0	0	0	1	0	0	0	ref|XP_001275855.1|	gamma interferon inducible lysosomal thiol reductase (GILT), putative	*Aspergillus clavatus NRRL 1*	3e-06	99
Singlet0699	FE526436	0	0	0	0	0	0	1	0	0	0	ref|XP_001242709.1|	hypothetical protein CIMG_06605	*Coccidioides immitis RS*	8e-44	-
Singlet0700	FE526437	0	0	0	0	0	0	1	0	0	0	ref|XP_754546.1|	Ribosomal protein S7e	*Aspergillus fumigatus Af293*	9e-31	12
Singlet0701	FE526439	0	0	0	0	0	0	1	0	0	0	emb|CAE74488.1|	Hypothetical protein CBG22239	*Caenorhabditis briggsae*	2e-12	99
Singlet0702	FE526446	0	0	0	0	0	0	1	0	0	0	ref|XP_001244970.1|	glucosamine-6-phosphate deaminase	*Coccidioides immitis RS*	4e-22	1, 2
Singlet0703	FE526452	0	0	0	0	0	0	1	0	0	0	ref|XP_001275026.1|	oligopeptide transporter	*Aspergillus clavatus NRRL 1*	2e-21	20
Singlet0704	FE526453	0	0	0	0	0	0	1	0	0	0	-	No significant similarity	-	-	-
Singlet0705	FE526454	0	0	0	0	0	0	1	0	0	0	gb|EEQ35176.1|	urease accessory protein UreD	*Microsporum canis CBS 113480*	8e-16	1, 16
Singlet0706	FE526455	0	0	0	0	0	0	1	0	0	0	-	No significant similarity	-	-	-
Singlet0707	FE526461	0	0	0	0	0	0	1	0	0	0	ref|NP_064450.2|	formin 2	*Homo sapiens*	9e-06	99
Singlet0708	FE526468	0	0	0	0	0	0	1	0	0	0	-	No significant similarity	-	-	-
Singlet0709	FE526481	0	0	0	0	0	0	1	0	0	0	ref|XP_001210481.1|	protein MGM1, mitochondrial precursor	*Aspergillus terreus NIH2624*	3e-41	10, 16
Singlet0710	FE526494	0	0	0	0	0	0	1	0	0	0	ref|XP_001246302.1|	hypothetical protein CIMG_00073	*Coccidioides immitis RS*	1e-25	99
Singlet0711	FE526497	0	0	0	0	0	0	1	0	0	0	gb|EAX07174.1|	hCG1793893	*Homo sapiens*	2e-05	99
Singlet0712	FE526499	0	0	0	0	0	0	1	0	0	0	ref|XP_001211350.1|	sterol 24-C-methyltransferase	*Aspergillus terreus NIH2624*	7e-23	1
Singlet0713	FE526508	0	0	0	0	0	0	1	0	0	0	ref|XP_001271762.1|	short chain dehydrogenase, putative	*Aspergillus clavatus NRRL 1*	1e-25	1, 34

Singlet0714	FE526509	0	0	0	0	0	0	1	0	0	0	ref|XP_001261901.1|	extracellular lipase, putative	*Neosartorya fischeri NRRL 181*	5e-19	1
Singlet0715	FE526513	0	0	0	0	0	0	1	0	0	0	emb|CAK48124.1|	unnamed protein product	*Aspergillus niger*	4e-12	99
Singlet0716	FE526514	0	0	0	0	0	0	1	0	0	0	ref|XP_001218693.1|	cholinesterase	*Aspergillus terreus NIH2624*	2e-08	1
Singlet0717	FE526515	0	0	0	0	0	0	1	0	0	0	gb|EEQ29740.1|	Aminoacid permease	*Microsporum canis*	1e-24	20
Singlet0718	FE526519	0	0	0	0	0	0	1	0	0	0	ref|XP_001245309.1|	hypothetical protein CIMG_04750	*Coccidioides immitis RS*	1e-07	99
Singlet0719	FE526521	0	0	0	0	0	0	1	0	0	0	ref|XP_755153.1|	flotillin domain protein	*Aspergillus fumigatus Af293*	4e-16	99
Singlet0720	FE526535	0	0	0	0	0	0	1	0	0	0	-	No significant similarity	-	-	-
Singlet0721	FE526541	0	0	0	0	0	0	1	0	0	0	gb|AAR08135.1|	small GTPase RanA	*Emericella nidulans*	2e-40	11, 12, 14, 18, 20
Singlet0722	FE526542	0	0	0	0	0	0	1	0	0	0	-	No significant similarity	-	-	-
Singlet0723	FE526544	0	0	0	0	0	0	1	0	0	0	-	No significant similarity	-	-	-
Singlet0724	FE526589	0	0	0	0	0	0	0	1	0	0	-	No significant similarity	-	-	-
Singlet0725	FE526619	0	0	0	0	0	0	0	1	0	0	gb|AAG24792.1|AF264028_2	pol protein	*Glomerella cingulata*	6e-20	38
Singlet0726	FE526620	0	0	0	0	0	0	0	1	0	0	ref|XP_001239812.1|	hypothetical protein CIMG_09433	*Coccidioides immitis RS*	4e-08	99
Singlet0727	FE526621	0	0	0	0	0	0	0	1	0	0	ref|ZP_01420399.1|	O-antigen polymerase	*Caulobacter sp. K31*	4e-17	99
Singlet0728	FE526625	0	0	0	0	0	0	0	1	0	0	-	No significant similarity	-	-	-
Singlet0729	FE526640	0	0	0	0	0	0	0	1	0	0	-	No significant similarity	-	-	-
Singlet0730	FE526647	0	0	0	0	0	0	0	1	0	0	gb|AAL26311.2|AF362957_1	polyprotein	*Aspergillus flavus*	2e-31	38
Singlet0731	FE526661	0	0	0	0	0	0	0	1	0	0	gb|AAG24792.1|AF264028_2	pol protein	*Glomerella cingulata*	2e-33	38
Singlet0732	FE526665	0	0	0	0	0	0	0	1	0	0	-	No significant similarity	-	-	-
Singlet0733	FE526676	0	0	0	0	0	0	0	1	0	0	-	No significant similarity	-	-	-
Singlet0734	FE526686	0	0	0	0	0	0	0	1	0	0	-	No significant similarity	-	-	-
Singlet0735	FE526701	0	0	0	0	0	0	0	1	0	0	ref|XP_001264529.1|	GABA permease, putative	*Neosartorya fischeri NRRL 181*	3e-17	20
Singlet0736	FE526702	0	0	0	0	0	0	0	1	0	0	ref|XP_001264853.1|	amino acid permease, putative	*Neosartorya fischeri NRRL 181*	3e-26	20
Singlet0737	FE526706	0	0	0	0	0	0	0	1	0	0	-	No significant similarity	-	-	-
Singlet0738	FE526718	0	0	0	0	0	0	0	1	0	0	gb|AAF21678.1|AF051915_2	pol polyprotein	*Cladosporium fulvum*	1e-09	38
Singlet0739	FE526735	0	0	0	0	0	0	0	1	0	0	-	No significant similarity	-	-	-
Singlet0740	FE526741	0	0	0	0	0	0	0	1	0	0	gb|EEQ32880.1|	FYVE zinc finger protein	*Microsporum canis* CBS 113480	1e-34	14, 16, 20
Singlet0741	FE526747	0	0	0	0	0	0	0	1	0	0	-	No significant similarity	-	-	-
Singlet0742	FE526750	0	0	0	0	0	0	0	1	0	0	-	No significant similarity	-	-	-

Singlet0743	FE526754	0	0	0	0	0	0	0	1	0	0	-	No significant similarity	-	-	-
Singlet0744	FE526755	0	0	0	0	0	0	0	0	1	0	-	No significant similarity	-	-	-
Singlet0745	FE526756	0	0	0	0	0	0	0	0	1	0	-	No significant similarity	-	-	-
Singlet0746	FE526759	0	0	0	0	0	0	0	0	1	0	-	No significant similarity	-	-	-
Singlet0747	FE526760	0	0	0	0	0	0	0	0	1	0	ref|XP_753233.1|	ThiJ/PfpI family protein	*Aspergillus fumigatus Af293*	9e-07	14, 16, 32
Singlet0748	FE526762	0	0	0	0	0	0	0	0	1	0	ref|XP_001243030.1|	hypothetical protein CIMG_06926	*Coccidioides immitis RS*	2e-06	-
Singlet0749	FE526764	0	0	0	0	0	0	0	0	1	0	ref|XP_001259622.1|	pentatricopeptide repeat protein	*Neosartorya fischeri NRRL 181*	1e-20	99
Singlet0750	FE526765	0	0	0	0	0	0	0	0	1	0	-	No significant similarity	-	-	-
Singlet0751	FE526766	0	0	0	0	0	0	0	0	1	0	-	No significant similarity	-	-	-
Singlet0752	FE526767	0	0	0	0	0	0	0	0	1	0	-	No significant similarity	-	-	-
Singlet0753	FE526768	0	0	0	0	0	0	0	0	1	0	-	No significant similarity	-	-	-
Singlet0754	FE526769	0	0	0	0	0	0	0	0	1	0	ref|XP_001259414.1|	50S ribosomal protein L4	*Neosartorya fischeri NRRL 181*	7e-05	12, 42
Singlet0755	FE526770	0	0	0	0	0	0	0	0	1	0	-	No significant similarity	-	-	-
Singlet0756	FE526771	0	0	0	0	0	0	0	0	1	0	gb|AAG36933.1|AF262955_1	oleate delta-12 desaturase	*Emericella nidulans*	9e-21	1
Singlet0757	FE526772	0	0	0	0	0	0	0	0	1	0	ref|XP_001248270.1|	hypothetical protein CIMG_02041	*Coccidioides immitis RS*	1e-04	-
Singlet0758	FE526773	0	0	0	0	0	0	0	0	1	0	ref|XP_001212165.1|	HNRNP arginine N-methyltransferase	*Aspergillus terreus NIH2624*	3e-16	1, 14, 16, 20,
Singlet0759	FE526774	0	0	0	0	0	0	0	0	1	0	-	No significant similarity	-	-	-
Singlet0760	FE526775	0	0	0	0	0	0	0	0	1	0	ref|XP_001269624.1|	ADP,ATP carrier protein	*Aspergillus clavatus NRRL 1*	2e-23	1, 2, 16, 20
Singlet0761	FE526776	0	0	0	0	0	0	0	0	1	0	-	No significant similarity	-	-	
Singlet0762	FE526777	0	0	0	0	0	0	0	0	1	0	ref|XP_001268371.1|	CECR1 family adenosine deaminase, putative	*Aspergillus clavatus NRRL 1*	4e-14	1, 40
Singlet0763	FE526779	0	0	0	0	0	0	0	0	1	0	ref|XP_001260012.1|	conserved hypothetical protein	*Neosartorya fischeri NRRL 181*	3e-07	99
Singlet0764	FE526780	0	0	0	0	0	0	0	0	1	0	ref|XP_001257300.1|	monocarboxylate permease homologue, mch4	*Neosartorya fischeri NRRL 181*	3e-08	20
Singlet0765	FE526781	0	0	0	0	0	0	0	0	1	0	-	No significant similarity	-	-	-
Singlet0766	FE526783	0	0	0	0	0	0	0	0	1	0	ref|XP_001275190.1|	amino acid transporter, putative	*Aspergillus clavatus NRRL 1*	4e-13	20
Singlet0767	FE526784	0	0	0	0	0	0	0	0	1	0	-	No significant similarity	-	-	-
Singlet0768	FE526785	0	0	0	0	0	0	0	0	1	0	-	No significant similarity	-	-	-
Singlet0769	FE526786	0	0	0	0	0	0	0	0	1	0	ref|XP_001245093.1|	hypothetical protein CIMG_04534	*Coccidioides immitis RS*	3e-07	99
Singlet0770	FE526787	0	0	0	0	0	0	0	0	1	0	ref|XP_726427.1|	RNA 3l-terminal phosphate cyclase protein	*Plasmodium yoelii yoelii str. 17XNL*	7e-04	11
Singlet0771	FE526788	0	0	0	0	0	0	0	0	1	0	ref|XP_001272674.1|	taurine catabolism dioxygenase TauD, TfdA family protein	*Aspergillus clavatus NRRL 1*	1e-24	1
Singlet0772	FE526789	0	0	0	0	0	0	0	0	1	0	-	No significant similarity	-	-	-

Singlet0773	FE526793	0	0	0	0	0	0	0	0	1	0	-	No significant similarity	-	-	-
Singlet0774	FE526796	0	0	0	0	0	0	0	0	1	0	ref|XP_001272771.1|	40S ribosomal protein S11	*Aspergillus clavatus NRRL 1*	2e-06	12, 16
Singlet0775	FE526797	0	0	0	0	0	0	0	0	1	0	-	No significant similarity	-	-	-
Singlet0776	FE526799	0	0	0	0	0	0	0	0	1	0	-	No significant similarity	-	-	-
Singlet0777	FE526801	0	0	0	0	0	0	0	0	1	0	-	No significant similarity	-	-	-
Singlet0778	FE526802	0	0	0	0	0	0	0	0	1	0	ref|XP_001213404.1|	predicted protein	*Aspergillus terreus NIH2624*	4e-15	99
Singlet0779	FE526803	0	0	0	0	0	0	0	0	1	0	-	No significant similarity	-	-	-
Singlet0780	FE526804	0	0	0	0	0	0	0	0	1	0	-	No significant similarity	-	-	-
Singlet0781	FE526806	0	0	0	0	0	0	0	0	1	0	gb|ABH10644.1|	aconitase	*Coccidioides posadasii*	2e-42	1, 2, 16
Singlet0782	FE526807	0	0	0	0	0	0	0	0	1	0	-	No significant similarity	-	-	-
Singlet0783	FE526808	0	0	0	0	0	0	0	0	1	0	gb|EAT82743.1|	predicted protein	*Phaeosphaeria nodorum SN15*	6e-04	99
Singlet0784	FE526809	0	0	0	0	0	0	0	0	1	0	-	No significant similarity	-	-	-
Singlet0785	FE526810	0	0	0	0	0	0	0	0	1	0	ref|XP_001267762.1|	glutathione S-transferase, putative	*Aspergillus clavatus NRRL 1*	1e-23	32
Singlet0786	FE526811	0	0	0	0	0	0	0	0	1	0	-	No significant similarity	-	-	-
Singlet0787	FE526812	0	0	0	0	0	0	0	0	1	0	-	No significant similarity	-	-	-
Singlet0788	FE526815	0	0	0	0	0	0	0	0	1	0	ref|XP_001243359.1|	hypothetical protein CIMG_07255	*Coccidioides immitis RS*	7e-04	99
Singlet0789	FE526817	0	0	0	0	0	0	0	0	1	0	ref|XP_001216898.1|	ADP-ribose pyrophosphatase	*Aspergillus terreus NIH2624*	2e-22	1, 2
Singlet0790	FE526818	0	0	0	0	0	0	0	0	1	0	ref|XP_001260458.1|	SNARE protein (Ufe1), putative	*Neosartorya fischeri NRRL 181*	5e-09	20, 40, 43
Singlet0791	FE526820	0	0	0	0	0	0	0	0	1	0	ref|XP_001212923.1|	60S ribosomal protein L20	*Aspergillus terreus NIH2624*	2e-05	12
Singlet0792	FE526821	0	0	0	0	0	0	0	0	1	0	ref|XP_001213571.1|	mitochondrial genome maintenance protein MGM101	*Aspergillus terreus NIH2624*	9e-48	10, 16
Singlet0793	FE526822	0	0	0	0	0	0	0	0	1	0	ref|XP_001260097.1|	cytochrome b5, putative	*Neosartorya fischeri NRRL 181*	6e-16	20
Singlet0794	FE526823	0	0	0	0	0	0	0	0	1	0	ref|XP_001215797.1|	multicopy enhancer of UAS2	*Aspergillus terreus NIH2624*	5e-50	1
Singlet0795	FE526824	0	0	0	0	0	0	0	0	1	0	sp|P34763|NMT_AJECA	Glycylpeptide N-tetradecanoyltransferase (Peptide N-myristoyltransferase) (Myristoyl-CoA:protein N-myristoyltransferase) (NMT) gb|AAA17549.1| N-myristoyltransferase	*Ajellomyces capsulatus*	6e-24	1,14
Singlet0796	FE526826	0	0	0	0	0	0	0	0	1	0	ref|XP_001247536.1|	lysyl-tRNA synthetase, cytoplasmic	*Coccidioides immitis RS*	4e-19	12, 16, 42
Singlet0797	FE526828	0	0	0	0	0	0	0	0	1	0	ref|XP_001240306.1|	cytochrome P450 51	*Coccidioides immitis RS*	1e-63	1, 16, 32

Singlet0798	FE526833	0	0	0	0	0	0	0	0	1	0	-	No significant similarity	-	-	-
Singlet0799	FE526834	0	0	0	0	0	0	0	0	1	0	ref|XP_001259852.1|	WD repeat protein	*Neosartorya fischeri NRRL 181*	2e-22	11, 40
Singlet0800	FE526837	0	0	0	0	0	0	0	0	1	0	ref|XP_001273261.1|	tropomyosin, putative	*Aspergillus clavatus NRRL 1*	6e-35	20, 42, 43, 34
Singlet0801	FE526838	0	0	0	0	0	0	0	0	1	0	-	No significant similarity	-	-	-
Singlet0802	FE526839	0	0	0	0	0	0	0	0	1	0	-	No significant similarity	-	-	-
Singlet0803	FE526840	0	0	0	0	0	0	0	0	1	0	-	No significant similarity	-	-	-
Singlet0804	FE526842	0	0	0	0	0	0	0	0	1	0	-	No significant similarity	-	-	-
Singlet0805	FE526843	0	0	0	0	0	0	0	0	1	0	-	No significant similarity	-	-	-
Singlet0806	FE526844	0	0	0	0	0	0	0	0	1	0	ref|XP_751502.1|	HAD superfamily hydrolase, putative	*Aspergillus fumigatus Af293*	3e-39	1
Singlet0807	FE526845	0	0	0	0	0	0	0	0	1	0	emb|CAK97343.1|	unnamed protein product	*Aspergillus niger*	4e-13	99
Singlet0808	FE526847	0	0	0	0	0	0	0	0	1	0	ref|XP_001270525.1|	conserved hypothetical protein	*Aspergillus clavatus NRRL 1*	9e-07	99
Singlet0809	FE526850	0	0	0	0	0	0	0	0	1	0	-	No significant similarity	-	-	-
Singlet0810	FE526851	0	0	0	0	0	0	0	0	1	0	ref|XP_001274066.1|	60S ribosome biogenesis protein Sqt1, putative	*Aspergillus clavatus NRRL 1*	7e-21	12, 14
Singlet0811	FE526852	0	0	0	0	0	0	0	0	1	0	ref|XP_001214720.1|	outer mitochondrial membrane protein porin	*Aspergillus terreus NIH2624*	4e-19	2, 16, 20, 40, 42
Singlet0812	FE526854	0	0	0	0	0	0	0	0	1	0	-	No significant similarity	-	-	-
Singlet0813	FE526855	0	0	0	0	0	0	0	0	1	0	sp|P07509|SODC_NEUCR	Superoxide dismutase	*Neurospora crassa*	2e-13	16, 32, 34
Singlet0814	FE526857	0	0	0	0	0	0	0	0	1	0	ref|XP_001268698.1|	succinate dehydrogenase subunit CybS, putative	*Aspergillus clavatus NRRL 1*	4e-07	1, 2
Singlet0815	FE526859	0	0	0	0	0	0	0	0	1	0	-	No significant similarity	-	-	-
Singlet0816	FE526860	0	0	0	0	0	0	0	0	1	0	-	No significant similarity	-	-	-
Singlet0817	FE526861	0	0	0	0	0	0	0	0	1	0	-	No significant similarity	-	-	-
Singlet0818	FE526862	0	0	0	0	0	0	0	0	1	0	-	No significant similarity	-	-	-
Singlet0819	FE526863	0	0	0	0	0	0	0	0	1	0	-	No significant similarity	-	-	-
Singlet0820	FE526864	0	0	0	0	0	0	0	0	1	0	ref|XP_001271235.1|	DUF1713 domain protein	*Aspergillus clavatus NRRL 1*	3e-07	99
Singlet0821	FE526866	0	0	0	0	0	0	0	0	1	0	ref|XP_001262838.1|	indoleamine 2,3-dioxygenase family protein	*Neosartorya fischeri NRRL 181*	1e-47	1
Singlet0822	FE526868	0	0	0	0	0	0	0	0	1	0	emb|CAK45164.1|	protein O-mannosyl transferase pmtA-Aspergillus niger	*Aspergillus niger*	1e-27	1, 14, 42
Singlet0823	FE526870	0	0	0	0	0	0	0	0	1	0	gb|EAL85168.2|	U5 snRNP component Snu114, putative	*Aspergillus fumigatus Af293*	1e-18	11, 16
Singlet0824	FE526872	0	0	0	0	0	0	0	0	1	0	ref|XP_001269346.1|	60S ribosomal protein L9, putative	*Aspergillus clavatus NRRL 1*	2e-20	12, 16
Singlet0825	FE526873	0	0	0	0	0	0	0	0	1	0	ref|XP_001243698.1|	fibrillarin	*Coccidioides immitis RS*	7e-50	1, 11, 12, 16
Singlet0826	FE526874	0	0	0	0	0	0	0	0	1	0	ref|XP_001264284.1|	kelch repeat protein	*Neosartorya fischeri*	6e-15	99

														NRRL 181		
Singlet0827	FE526875	0	0	0	0	0	0	0	0	1	0	-	No significant similarity	-	-	-
Singlet0828	FE526880	0	0	0	0	0	0	0	0	1	0	-	No significant similarity	-	-	-
Singlet0829	FE526882	0	0	0	0	0	0	0	0	1	0	ref|XP_001260103.1|	WD repeat protein	*Neosartorya fischeri NRRL 181*	3e-05	11, 40
Singlet0830	FE526883	0	0	0	0	0	0	0	0	1	0	gb|EDJ98154.1|	GTP cyclohydrolase II, putative	*Magnaporthe grisea 70-15*	3e-50	1
Singlet0831	FE526884	0	0	0	0	0	0	0	0	1	0	gb|EEQ30276.1|	mitochondrial 2-methylisocitrate lyase	*Microsporum canis CBS113480*	9e-31	1, 2
Singlet0832	FE526885	0	0	0	0	0	0	0	0	1	0	-	No significant similarity	-	-	-
Singlet0833	FE526889	0	0	0	0	0	0	0	0	1	0	ref|XP_001244571.1|	40S ribosomal protein S26E	*Coccidioides immitis RS*	4e-10	12, 16
Singlet0834	FE526890	0	0	0	0	0	0	0	0	1	0	-	No significant similarity	-	-	-
Singlet0835	FE526891	0	0	0	0	0	0	0	0	1	0	-	No significant similarity	-	-	-
Singlet0836	FE526895	0	0	0	0	0	0	0	0	1	0	-	No significant similarity	-	-	-
Singlet0837	FE526896	0	0	0	0	0	0	0	0	1	0	-	No significant similarity	-	-	-
Singlet0838	FE526897	0	0	0	0	0	0	0	0	1	0	emb|CAD29478.1|	glutathione transferase F5	*Triticum aestivum*	7e-07	1
Singlet0839	FE526898	0	0	0	0	0	0	0	0	1	0	-	No significant similarity	-	-	-
Singlet0840	FE526899	0	0	0	0	0	0	0	0	1	0	gb|AAM54368.1|	elongation factor 1-alpha	*Trichophyton rubrum*	7e-20	12, 16, 18, 40
Singlet0841	FE526900	0	0	0	0	0	0	0	0	1	0	-	No significant similarity	-	-	-
Singlet0842	FE526902	0	0	0	0	0	0	0	0	1	0	sp|Q5BGF9|PAM17_EMENI	Presequence translocated-associated motor subunit pam17, mitochondrial precursor	*Emericella nidulans*	8e-22	16, 20, 42
Singlet0843	FE526904	0	0	0	0	0	0	0	0	1	0	-	No significant similarity	-	-	
Singlet0844	FE526905	0	0	0	0	0	0	0	0	1	0	gb|AAS76666.1|	carboxypeptidase S1	*Trichophyton rubrum*	5e-48	1, 14
Singlet0845	FE526906	0	0	0	0	0	0	0	0	1	0	sp|Q5AZJ7|DPH1_EMENI	Diphthamide biosynthesis protein	*Emericella nidulans*	3e-36	1
Singlet0846	FE526910	0	0	0	0	0	0	0	0	1	0	ref|XP_001240451.1|	aspartyl aminopeptidase, putative	*Coccidioides immitis RS*	5e-47	14, 16
Singlet0847	FE526911	0	0	0	0	0	0	0	0	1	0	-	No significant similarity	-	-	-
Singlet0848	FE526914	0	0	0	0	0	0	0	0	1	0	-	No significant similarity	-	-	-
Singlet0849	FE526915	0	0	0	0	0	0	0	0	1	0	-	No significant similarity	-	-	-
Singlet0850	FE526916	0	0	0	0	0	0	0	0	1	0	ref|XP_001244035.1|	hypothetical protein CIMG_03476	*Coccidioides immitis RS*	2e-12	99
Singlet0851	FE526919	0	0	0	0	0	0	0	0	1	0	gb|EAL90446.2|	eukaryotic translation elongation factor 1 subunit Eef1-beta, putative	*Aspergillus fumigatus Af293*	6e-19	12, 16, 18
Singlet0852	FE526920	0	0	0	0	0	0	0	0	1	0	emb|CAC18218.1|	probable TRANSKETOLASE	*Neurospora crassa*	6e-22	1, 2
Singlet0853	FE526921	0	0	0	0	0	0	0	0	1	0	-	No significant similarity	-	-	-
Singlet0854	FE526922	0	0	0	0	0	0	0	0	1	0	gb|ABH11414.1|	peroxin 3	*Penicillium chrysogenum*	1e-07	14, 20, 42
Singlet0855	FE526923	0	0	0	0	0	0	0	0	1	0	-	No significant similarity	-	-	-
Singlet0856	FE526924	0	0	0	0	0	0	0	0	1	0	ref|XP_567138.1|	siderochrome-iron (ferrioxamine)	*Cryptococcus*	5e-21	1, 20,

												uptake transporter	*neoformans var. neoformans JEC21*		32, 34
Singlet0857	FE526926	0	0	0	0	0	0	0	0	1	0	gb|ABH10638.1|	enolase	*Coccidioides posadasii*	5e-29	1, 2, 16
Singlet0858	FE526927	0	0	0	0	0	0	0	0	1	0	ref|XP_001261926.1|	ELMO/CED-12 family protein	*Neosartorya fischeri NRRL 181*	1e-28	99
Singlet0859	FE526928	0	0	0	0	0	0	0	0	1	0	-	No significant similarity	-	-	-
Singlet0860	FE526930	0	0	0	0	0	0	0	0	1	0	-	No significant similarity	-	-	-
Singlet0861	FE526933	0	0	0	0	0	0	0	0	1	0	ref|XP_001241872.1|	hypothetical protein CIMG_05768	*Coccidioides immitis RS*	2e-05	99
Singlet0862	FE526934	0	0	0	0	0	0	0	0	1	0	ref|XP_753437.1|	integral membrane protein, putative	*Aspergillus fumigatus Af293*	5e-05	20, 32
Singlet0863	FE526938	0	0	0	0	0	0	0	0	1	0	ref|XP_001266988.1|	phosphatase-like protein (PTPLA), putative	*Neosartorya fischeri NRRL 181*	7e-13	99
Singlet0864	FE526939	0	0	0	0	0	0	0	0	1	0	ref|XP_001220066.1|	40S ribosomal protein S11	*Chaetomium globosum CBS 148.51*	3e-04	12, 16
Singlet0865	FE526945	0	0	0	0	0	0	0	0	1	0	ref|XP_001247826.1|	2-oxoglutarate dehydrogenase E1 component	*Coccidioides immitis RS*	3e-15	1, 2, 16
Singlet0866	FE526949	0	0	0	0	0	0	0	0	1	0	-	No significant similarity	-	-	-
Singlet0867	FE526951	0	0	0	0	0	0	0	0	1	0	-	No significant similarity	-	-	-
Singlet0868	FE526952	0	0	0	0	0	0	0	0	1	0	ref|XP_001269007.1|	oleate delta-12 desaturase	*Aspergillus clavatus NRRL 1*	7e-13	1
Singlet0869	FE526956	0	0	0	0	0	0	0	0	1	0	ref|XP_001247300.1|	hypothetical protein CIMG_01071	*Coccidioides immitis RS*	1e-05	99
Singlet0870	FE526957	0	0	0	0	0	0	0	0	1	0	ref|XP_001267424.1|	phosphoethanolamine	*Neosartorya fischeri NRRL 181*	8e-22	1
Singlet0871	FE526958	0	0	0	0	0	0	0	0	1	0	ref|XP_001242955.1|	hypothetical protein CIMG_06851	*Coccidioides immitis RS*	1e-08	99
Singlet0872	FE526959	0	0	0	0	0	0	0	0	1	0	-	No significant similarity	-	-	-
Singlet0873	FE526961	0	0	0	0	0	0	0	0	1	0	-	No significant similarity	-	-	-
Singlet0874	FE526962	0	0	0	0	0	0	0	0	1	0	ref|XP_001259336.1|	cell cycle control protein (Cwf8), putative	*Neosartorya fischeri NRRL 181*	1e-27	99
Singlet0875	FE526963	0	0	0	0	0	0	0	0	1	0	ref|XP_001210588.1|	U3 small nucleolar ribonucleoprotein protein IMP4	*Aspergillus terreus NIH2624*	5e-43	11, 12, 16
Singlet0876	FE526964	0	0	0	0	0	0	0	0	1	0	ref|XP_001261302.1|	C2 domain protein	*Neosartorya fischeri NRRL 181*	5e-29	99
Singlet0877	FE526965	0	0	0	0	0	0	0	0	1	0	ref|XP_001212323.1|	predicted protein	*Aspergillus terreus NIH2624*	8e-09	99
Singlet0878	FE526966	0	0	0	0	0	0	0	0	1	0	ref|XP_001267850.1|	antigenic mitochondrial protein HSP60, putative	*Aspergillus clavatus NRRL 1*	5e-13	14, 16, 20, 32, 40
Singlet0879	FE526968	0	0	0	0	0	0	0	0	1	0	ref|XP_001248397.1|	hypothetical protein CIMG_02168	*Coccidioides immitis RS*	2e-05	99
Singlet0880	FE526969	0	0	0	0	0	0	0	0	1	0	gb|AAL50803.1|AF452883_1	Y20 protein	*Paracoccidioides*	6e-30	99

														brasiliensis		
Singlet0881	FE526972	0	0	0	0	0	0	0	0	1	0	pir||S63701	mannosyl-oligosaccharide 1,2-alpha-mannosidase (EC 3.2.1.113) precursor	*Aspergillus phoenicis*	2e-13	1, 14
Singlet0882	FE526973	0	0	0	0	0	0	0	0	1	0	ref|XP_001242456.1|	hypothetical protein CIMG_06352	*Coccidioides immitis RS*	1e-04	99
Singlet0883	FE526974	0	0	0	0	0	0	0	0	1	0	ref|XP_001242392.1|	plasma membrane ATPase	*Coccidioides immitis RS*	3e-43	2, 20, 34
Singlet0884	FE526975	0	0	0	0	0	0	0	0	1	0	gb|AAT40563.1|	small G-protein GPA3	*Paracoccidioides brasiliensis*	2e-29	1, 14, 16, 18, 30, 34, 40, 43
Singlet0885	FE526978	0	0	0	0	0	0	0	0	1	0	-	No significant similarity	-	-	
Singlet0886	FE526980	0	0	0	0	0	0	0	0	1	0	ref|XP_001269939.1|	eukaryotic translation initiation factor 3 subunit EifCb, putative	*Aspergillus clavatus NRRL 1*	2e-36	11, 12, 16
Singlet0887	FE526982	0	0	0	0	0	0	0	0	1	0	-	No significant similarity	-	-	-
Singlet0888	FE526983	0	0	0	0	0	0	0	0	1	0	ref|XP_001248096.1|	acetamidase	*Coccidioides immitis RS*	6e-33	1
Singlet0889	FE526986	0	0	0	0	0	0	0	0	1	0	-	No significant similarity	-	-	-
Singlet0890	FE526987	0	0	0	0	0	0	0	0	1	0	-	No significant similarity	-	-	-
Singlet0891	FE526988	0	0	0	0	0	0	0	0	1	0	-	No significant similarity	-	-	-
Singlet0892	FE526990	0	0	0	0	0	0	0	0	1	0	-	No significant similarity	-	-	-
Singlet0893	FE526991	0	0	0	0	0	0	0	0	1	0	-	No significant similarity	-	-	-
Singlet0894	FE526994	0	0	0	0	0	0	0	0	1	0	ref|XP_001386580.1|	membrane protein involved in vacuolar protein sorting	*Pichia stipitis CBS 6054*	3e-07	14, 20, 42
Singlet0895	FE526995	0	0	0	0	0	0	0	0	1	0	-	No significant similarity	-	-	-
Singlet0896	FE526996	0	0	0	0	0	0	0	0	1	0	-	No significant similarity	-	-	-
Singlet0897	FE526998	0	0	0	0	0	0	0	0	1	0	ref|XP_001263178.1|	ubiquitin conjugating enzyme (UbcB), putative	*Neosartorya fischeri NRRL 181*	3e-08	14, 16, 34
Singlet0898	FE527000	0	0	0	0	0	0	0	0	1	0	ref|XP_001248200.1|	predicted protein	*Coccidioides immitis RS*	1e-09	99
Singlet0899	FE527001	0	0	0	0	0	0	0	0	1	0	-	No significant similarity	-	-	-
Singlet0900	FE527005	0	0	0	0	0	0	0	0	1	0	-	No significant similarity	-	-	-
Singlet0901	FE527007	0	0	0	0	0	0	0	0	1	0	-	No significant similarity	-	-	-
Singlet0902	FE527013	0	0	0	0	0	0	0	0	1	0	ref|XP_001240365.1|	hypothetical protein CIMG_07528	*Coccidioides immitis RS*	3e-05	99
Singlet0903	FE527016	0	0	0	0	0	0	0	0	1	0	ref|XP_001259429.1|	dihydroxyacetone kinase (DakA), putative	*Neosartorya fischeri NRRL 181*	5e-10	1, 16, 32
Singlet0904	FE527017	0	0	0	0	0	0	0	0	1	0	-	No significant similarity	-	-	-
Singlet0905	FE527018	0	0	0	0	0	0	0	0	1	0	-	No significant similarity	-	-	-
Singlet0906	FE527019	0	0	0	0	0	0	0	0	1	0	-	No significant similarity	-	-	-
Singlet0907	FE527022	0	0	0	0	0	0	0	0	1	0	-	No significant similarity	-	-	-
Singlet0908	FE527024	0	0	0	0	0	0	0	0	1	0	-	No significant similarity	-	-	-

Singlet0909	FE527027	0	0	0	0	0	0	0	0	1	0	ref|XP_001245288.1|	heat shock protein hsp1	*Coccidioides immitis RS*	2e-11	14, 16
Singlet0910	FE527031	0	0	0	0	0	0	0	0	1	0	emb|CAD70291.1|	related to mitochondrial serine--tRNA ligase	*Neurospora crassa*	7e-05	12
Singlet0911	FE527032	0	0	0	0	0	0	0	0	1	0	-	No significant similarity	-	-	-
Singlet0912	FE527034	0	0	0	0	0	0	0	0	1	0	gb|AAK52822.1|AF365926_1	calmodulin-binding coil-coil protein	*Emericella nidulans*	4e-07	16
Singlet0913	FE527036	0	0	0	0	0	0	0	0	1	0	ref|XP_001268653.1|	TIM barrel metal-dependent hydrolase, putative	*Aspergillus clavatus NRRL 1*	3e-07	99
Singlet0914	FE527037	0	0	0	0	0	0	0	0	1	0	-	No significant similarity	-	-	-
Singlet0915	FE527040	0	0	0	0	0	0	0	0	1	0	emb|CAA49847.1|	GMP synthase	*Saccharomyces cerevisiae*	7e-12	1
Singlet0916	FE527041	0	0	0	0	0	0	0	0	1	0	ref|XP_001262785.1|	MFS peptide transporter Ptr2, putative	*Neosartorya fischeri NRRL 181*	1e-15	20
Singlet0917	FE527042	0	0	0	0	0	0	0	0	1	0	-	No significant similarity	-	-	-
Singlet0918	FE527043	0	0	0	0	0	0	0	0	1	0	-	No significant similarity	-	-	-
Singlet0919	FE527046	0	0	0	0	0	0	0	0	1	0	-	No significant similarity	-	-	-
Singlet0920	FE527049	0	0	0	0	0	0	0	0	1	0	-	No significant similarity	-	-	-
Singlet0921	FE527053	0	0	0	0	0	0	0	0	1	0	gb|EDK03984.1|	mitochondrial NADH-ubiquinone oxidoreductase 20 kD subunit	*Magnaporthe grisea 70-15*	8e-27	2
Singlet0922	FE527054	0	0	0	0	0	0	0	0	1	0	-	No significant similarity	-	-	-
Singlet0923	FE527056	0	0	0	0	0	0	0	0	1	0	-	No significant similarity	-	-	-
Singlet0924	FE527059	0	0	0	0	0	0	0	0	1	0	ref|XP_001261939.1|	Hsp70 chaperone (HscA), putative	*Neosartorya fischeri NRRL 181*	1e-16	32, 40
Singlet0925	FE527061	0	0	0	0	0	0	0	0	1	0	-	No significant similarity	-	-	-
Singlet0926	FE527063	0	0	0	0	0	0	0	0	1	0	-	No significant similarity	-	-	-
Singlet0927	FE527066	0	0	0	0	0	0	0	0	1	0	-	No significant similarity	-	-	-
Singlet0928	FE527067	0	0	0	0	0	0	0	0	1	0	ref|XP_001240912.1|	hypothetical protein CIMG_08075	*Coccidioides immitis RS*	2e-06	99
Singlet0929	FE527068	0	0	0	0	0	0	0	0	1	0	ref|XP_001268219.1|	3-oxoacyl-(acyl-carrier-protein) reductase	*Aspergillus clavatus NRRL 1*	1e-21	1, 2, 16
Singlet0930	FE527069	0	0	0	0	0	0	0	0	1	0	ref|XP_001268984.1|	PfkB family carbohydrate kinase (Mak32), putative	*Aspergillus clavatus NRRL 1*	1e-14	38
Singlet0931	FE527070	0	0	0	0	0	0	0	0	1	0	ref|XP_001215828.1|	tRNA (uridine-2l-O-)-methyltransferase TRM7	*Aspergillus terreus NIH2624*	5e-13	11
Singlet0932	FE527071	0	0	0	0	0	0	0	0	1	0	-	No significant similarity	-	-	-
Singlet0933	FE527072	0	0	0	0	0	0	0	0	1	0	ref|XP_001266337.1|	phosphate transporter (Pho88), putative	*Neosartorya fischeri NRRL 181*	6e-07	1, 20
Singlet0934	FE527073	0	0	0	0	0	0	0	0	1	0	-	No significant similarity	-	-	-
Singlet0935	FE527074	0	0	0	0	0	0	0	0	1	0	gb|EAT88174.1|	hypothetical protein SNOG_04414	*Phaeosphaeria nodorum SN15*	3e-04	99
Singlet0936	FE527075	0	0	0	0	0	0	0	0	1	0	-	No significant similarity	-	-	-
Singlet0937	FE527077	0	0	0	0	0	0	0	0	1	0	-	No significant similarity	-	-	-
Singlet0938	FE527078	0	0	0	0	0	0	0	0	1	0	gb|AAP22960.1|	14-3-3-like protein	*Paracoccidioides*	9e-07	11, 14,

														brasiliensis		16, 30, 43
Singlet0939	FE527079	0	0	0	0	0	0	0	0	1	0	sp|Q8J1M3|DPP5_ARTBE	Dipeptidyl-peptidase 5 precursor (Dipeptidyl-peptidase V) (DPP V) (DppV) (Allergen Tri m 4) emb|CAD23611.1| tri m 4 allergen	*Arthroderma benhamiae*	5e-21	14
Singlet0940	FE527081	0	0	0	0	0	0	0	0	1	0	-	No significant similarity	-	-	-
Singlet0941	FE527083	0	0	0	0	0	0	0	0	1	0	-	No significant similarity	-	-	-
Singlet0942	FE527086	0	0	0	0	0	0	0	0	1	0	ref|XP_001274406.1|	DNA-directed RNA polymerases i, ii, and iii 145 kDa polypeptide	*Aspergillus clavatus NRRL 1*	2e-16	11, 16
Singlet0943	FE527087	0	0	0	0	0	0	0	0	1	0	ref|XP_747704.1|	prpd protein	*Aspergillus fumigatus Af293*	1e-24	99
Singlet0944	FE527088	0	0	0	0	0	0	0	0	1	0	-	No significant similarity	-	-	-
Singlet0945	FE527089	0	0	0	0	0	0	0	0	1	0	-	No significant similarity	-	-	-
Singlet0946	FE527091	0	0	0	0	0	0	0	0	1	0	ref|XP_001267123.1|	60S ribosomal protein L25, putative	*Neosartorya fischeri NRRL 181*	4e-18	12, 16
Singlet0947	FE527092	0	0	0	0	0	0	0	0	1	0	-	No significant similarity	-	-	-
Singlet0948	FE527093	0	0	0	0	0	0	0	0	1	0	-	No significant similarity	-	-	-
Singlet0949	FE527094	0	0	0	0	0	0	0	0	1	0	sp|A1CSR1|MRH4_ASPCL	ATP-dependent RNA helicase mrh4, mitochondrial precursor	*Aspergillus clavatus*	2e-14	11, 12, 14, 16
Singlet0950	FE527096	0	0	0	0	0	0	0	0	1	0	ref|XP_753520.1|	D-amino acid oxidase	*Aspergillus fumigatus Af293*	4e-11	1
Singlet0951	FE527097	0	0	0	0	0	0	0	0	1	0	gb|ABE01845.1|	beta-tubulin	*Microsporum canis*	7e-15	10, 42
Singlet0952	FE527098	0	0	0	0	0	0	0	0	1	0	-	No significant similarity	-	-	-
Singlet0953	FE527100	0	0	0	0	0	0	0	0	1	0	gb|EAT76933.1|	hypothetical protein SNOG_15558	*Phaeosphaeria nodorum SN15*	1e-05	99
Singlet0954	FE527101	0	0	0	0	0	0	0	0	1	0	-	No significant similarity	-	-	-
Singlet0955	FE527102	0	0	0	0	0	0	0	0	1	0	ref|XP_001268336.1|	protein transport protein Sec24, putative	*Aspergillus clavatus NRRL 1*	2e-20	14, 16, 20
Singlet0956	FE527103	0	0	0	0	0	0	0	0	1	0	ref|XP_001209399.1|	succinate/fumarate mitochondrial transporter	*Aspergillus terreus NIH2624*	1e-32	1, 20, 42
Singlet0957	FE527104	0	0	0	0	0	0	0	0	1	0	ref|XP_001273981.1|	vacuolar protein sorting protein DigA	*Aspergillus clavatus NRRL 1*	4e-24	16, 20, 42
Singlet0958	FE527105	0	0	0	0	0	0	0	0	1	0	-	No significant similarity	-	-	-
Singlet0959	FE527108	0	0	0	0	0	0	0	0	1	0	ref|XP_001264551.1|	RING finger ubiquitin ligase (Tul1), putative	*Neosartorya fischeri NRRL 181*	2e-09	14, 20
Singlet0960	FE527112	0	0	0	0	0	0	0	0	1	0	ref|XP_001273487.1|	short-chain dehydrogenase	*Aspergillus clavatus NRRL 1*	1e-05	1, 34
Singlet0961	FE527113	0	0	0	0	0	0	0	0	1	0	ref|XP_001264756.1|	adenosylhomocysteinase	*Neosartorya fischeri NRRL 181*	5e-23	1,16
Singlet0962	FE527116	0	0	0	0	0	0	0	0	1	0	-	No significant similarity	-	-	-
Singlet0963	FE527117	0	0	0	0	0	0	0	0	1	0	-	No significant similarity	-	-	-
Singlet0964	FE527123	0	0	0	0	0	0	0	0	1	0	ref|NP_446043.1|	dipeptidase 1 (renal)	*Rattus norvegicus*	8e-06	99
Singlet0965	FE527126	0	0	0	0	0	0	0	0	1	0	ref|XP_001271844.1|	WD repeat protein	*Aspergillus clavatus*	1e-07	11, 40

													NRRL 1		
Singlet0966	FE527127	0	0	0	0	0	0	0	0	0	1	ref|XP_001268989.1|	multidrug resistance protein 1, 2, 3 (p glycoprotein 1, 2, 3)	*Aspergillus clavatus NRRL 1*	2e-09	20, 32
Singlet0967	FE527128	0	0	0	0	0	0	0	0	0	1	ref|XP_001240756.1|	hypothetical protein CIMG_07919	*Coccidioides immitis RS*	2e-06	99
Singlet0968	FE527132	0	0	0	0	0	0	0	0	0	1	ref|XP_001265914.1|	sugar transporter, putative	*Neosartorya fischeri NRRL 181*	1e-06	20
Singlet0969	FE527133	0	0	0	0	0	0	0	0	0	1	ref|XP_001269812.1|	adenylate kinase 2	*Aspergillus clavatus NRRL 1*	9e-07	1,10, 16
Singlet0970	FE527136	0	0	0	0	0	0	0	0	0	1	ref|XP_001541343.1|	conserved hypothetical protein	*Ajellomyces capsulatus NAm1*	2e-05	99
Singlet0971	FE527137	0	0	0	0	0	0	0	0	0	1	ref|XP_001271103.1|	Bromodomain protein	*Aspergillus clavatus NRRL 1*	3e-15	11, 43
Singlet0972	FE527140	0	0	0	0	0	0	0	0	0	1	-	No significant similarity	-	-	-
Singlet0973	FE527141	0	0	0	0	0	0	0	0	0	1	ref|XP_001217407.1|	conserved hypothetical protein	*Aspergillus terreus NIH2624*	1e-06	99
Singlet0974	FE527142	0	0	0	0	0	0	0	0	0	1	-	No significant similarity	-	-	-
Singlet0975	FE527144	0	0	0	0	0	0	0	0	0	1	ref|XP_001543575.1|	amino acid permease Dip5	*Ajellomyces capsulatus NAm1*	3e-26	1, 20, 32, 34
Singlet0976	FE527145	0	0	0	0	0	0	0	0	0	1	ref|XP_001542093.1|	sodium transport ATPase 5	*Ajellomyces capsulatus NAm1*	1e-12	99
Singlet0977	FE527148	0	0	0	0	0	0	0	0	0	1	-	No significant similarity	-	-	-
Singlet0978	FE527149	0	0	0	0	0	0	0	0	0	1	-	No significant similarity	-	-	-
Singlet0979	FE527150	0	0	0	0	0	0	0	0	0	1	-	No significant similarity	-	-	-
Singlet0980	FE527152	0	0	0	0	0	0	0	0	0	1	ref|XP_001257858.1|	isocitrate dehydrogenase LysB	*Neosartorya fischeri NRRL 181*	2e-47	1, 2, 16, 20
Singlet0981	FE527153	0	0	0	0	0	0	0	0	0	1	-	No significant similarity	-	-	-
Singlet0982	FE527154	0	0	0	0	0	0	0	0	0	1	-	No significant similarity	-	-	-
Singlet0983	FE527155	0	0	0	0	0	0	0	0	0	1	-	No significant similarity	-	-	-
Singlet0984	FE527156	0	0	0	0	0	0	0	0	0	1	ref|XP_001268212.1|	PF02656 domain protein	*Aspergillus clavatus NRRL 1*	2e-13	99
Singlet0985	FE527158	0	0	0	0	0	0	0	0	0	1	ref|XP_001260492.1|	SET and MYND domain protein, putative	*Neosartorya fischeri NRRL 181*	6e-06	99
Singlet0986	FE527161	0	0	0	0	0	0	0	0	0	1	ref|XP_001273744.1|	arginine permease	*Aspergillus clavatus NRRL 1*	1e-20	20, 34
Singlet0987	FE527162	0	0	0	0	0	0	0	0	0	1	ref|XP_001536307.1|	predicted protein	*Ajellomyces capsulatus NAm1*	1e-10	99
Singlet0988	FE527163	0	0	0	0	0	0	0	0	0	1	ref|XP_001259189.1|	F-box domain protein	*Neosartorya fischeri NRRL 181*	7e-25	14
Singlet0989	FE527164	0	0	0	0	0	0	0	0	0	1	ref|XP_001537488.1|	ER-derived vesicles protein ERV14	*Ajellomyces capsulatus NAm1*	2e-07	20, 30, 43
Singlet0990	FE527165	0	0	0	0	0	0	0	0	0	1	-	No significant similarity	-	-	-
Singlet0991	FE527166	0	0	0	0	0	0	0	0	0	1	-	No significant similarity	-	-	-
Singlet0992	FE527167	0	0	0	0	0	0	0	0	0	1	ref|XP_750288.1|	mitochondrial ADP,ATP carrier	*Aspergillus*	4e-22	2, 20

													protein (Ant), putative	*fumigatus Af293*		
Singlet0993	FE527168	0	0	0	0	0	0	0	0	0	1	ref|XP_001057319.1|	PREDICTED: hypothetical protein	*Rattus norvegicus*	5e-11	99
Singlet0994	FE527171	0	0	0	0	0	0	0	0	0	1	gb|ABH10645.1|	ATP synthase beta chain	*Coccidioides posadasii*	4e-21	2, 16, 20, 34, 40
Singlet0995	FE527173	0	0	0	0	0	0	0	0	0	1	ref|XP_001211936.1|	26S protease regulatory subunit 8	*Aspergillus terreus NIH2624*	9e-12	10, 11, 14, 16, 18, 32
Singlet0996	FE527174	0	0	0	0	0	0	0	0	0	1	ref|XP_001537882.1|	thiamine-phosphate pyrophosphorylase	*Ajellomyces capsulatus NAm1*	2e-17	1
Singlet0997	FE527175	0	0	0	0	0	0	0	0	0	1	ref|XP_001260814.1|	translation elongation factor EF-2 subunit, putative	*Neosartorya fischeri NRRL 181*	4e-11	1, 11, 12, 16
Singlet0998	FE527176	0	0	0	0	0	0	0	0	0	1	-	No significant similarity	-	-	-
Singlet0999	FE527179	0	0	0	0	0	0	0	0	0	1	ref|XP_001242914.1|	hypothetical protein CIMG_06810	*Coccidioides immitis RS*	3e-08	99
Singlet1000	FE527180	0	0	0	0	0	0	0	0	0	1	-	No significant similarity	-	-	-
Singlet1001	FE527183	0	0	0	0	0	0	0	0	0	1	-	No significant similarity	-	-	-
Singlet1002	FE527185	0	0	0	0	0	0	0	0	0	1	ref|XP_001266126.1|	oxidosqualene:lanosterol cyclase	*Neosartorya fischeri NRRL 181*	2e-10	99
Singlet1003	FE527186	0	0	0	0	0	0	0	0	0	1	ref|YP_704246.1|	possible dipeptidase	*Rhodococcus sp. RHA1*	2e-13	14, 16
Singlet1004	FE527187	0	0	0	0	0	0	0	0	0	1	ref|XP_753972.1|	glutathione synthetase, putative	*Aspergillus fumigatus Af293*	5e-12	1, 16, 32
Singlet1005	FE527189	0	0	0	0	0	0	0	0	0	1	ref|XP_001240587.1|	hypothetical protein CIMG_07750	*Coccidioides immitis RS*	1e-04	-
Singlet1006	FE527194	0	0	0	0	0	0	0	0	0	1	ref|XP_001540148.1|	conserved hypothetical protein	*Ajellomyces capsulatus NAm1*	1e-18	99
Singlet1007	FE527195	0	0	0	0	0	0	0	0	0	1	-	No significant similarity	-	-	-
Singlet1008	FE527196	0	0	0	0	0	0	0	0	0	1	ref|XP_001241535.1|	hypothetical protein CIMG_08698	*Coccidioides immitis RS*	4e-06	-
Singlet1009	FE527203	0	0	0	0	0	0	0	0	0	1	ref|XP_750370.1|	N-acetylglucosamine-phosphate mutase	*Aspergillus fumigatus Af293*	1e-15	99
Singlet1010	FE527204	0	0	0	0	0	0	0	0	0	1	-	No significant similarity	-	-	-
Singlet1011	FE527205	0	0	0	0	0	0	0	0	0	1	ref|XP_001257807.1|	Rho GTPase Rho1	*Neosartorya fischeri NRRL 181*	3e-81	14, 18, 30, 42
Singlet1012	FE527206	0	0	0	0	0	0	0	0	0	1	ref|XP_750999.1|	DUF domain protein	*Aspergillus fumigatus Af293*	4e-05	99
Singlet1013	FE527207	0	0	0	0	0	0	0	0	0	1	ref|XP_001246171.1|	hypothetical protein CIMG_05612	*Coccidioides immitis RS*	2e-08	-
Singlet1014	FE527209	0	0	0	0	0	0	0	0	0	1	ref|XP_001542733.1|	ATP-binding cassette sub-family F member 2	*Ajellomyces capsulatus NAm1*	7e-43	20
Singlet1015	FE527212	0	0	0	0	0	0	0	0	0	1	-	No significant similarity	-	-	-
Singlet1016	FE527213	0	0	0	0	0	0	0	0	0	1	-	No significant similarity	-	-	-
Singlet1017	FE527215	0	0	0	0	0	0	0	0	0	1	-	No significant similarity	-	-	-

Singlet1018	FE527218	0	0	0	0	0	0	0	0	0	1	ref|XP_001264609.1|	CORD and CS domain protein	*Neosartorya fischeri NRRL 181*	5e-05	99
Singlet1019	FE527219	0	0	0	0	0	0	0	0	0	1	ref|XP_362903.2|	c-14 sterol reductase	*Magnaporthe grisea 70-15*	9e-41	1, 16
Singlet1020	FE527220	0	0	0	0	0	0	0	0	0	1	ref|XP_001276316.1|	actin interacting protein 2	*Aspergillus clavatus NRRL 1*	2e-53	1, 2, 16, 42
Singlet1021	FE527225	0	0	0	0	0	0	0	0	0	1	ref|XP_001258414.1|	40S ribosomal protein S29, putative	*Neosartorya fischeri NRRL 181*	6e-10	12
Singlet1022	FE527227	0	0	0	0	0	0	0	0	0	1	gb|EEQ28484.1|	iron sulfur cluster assembly protein 1, mitochondrial precursor	*Microsporum canis CBS113480*	1e-44	99
Singlet1023	FE527228	0	0	0	0	0	0	0	0	0	1	ref|XP_001238954.1|	cytochrome c1,	*Coccidioides immitis RS*	1e-44	2, 16, 20
Singlet1024	FE527229	0	0	0	0	0	0	0	0	0	1	-	No significant similarity	-	-	-
Singlet1025	FE527230	0	0	0	0	0	0	0	0	0	1	ref|XP_001543311.1|	mitochondrial import inner membrane translocase subunit tim22	*Ajellomyces capsulatus NAm1*	9e-25	14, 20
Singlet1026	FE527232	0	0	0	0	0	0	0	0	0	1	-	No significant similarity	-	-	-
Singlet1027	FE527233	0	0	0	0	0	0	0	0	0	1	gb|ABG67901.1|	putative phospholipase	*Trichophyton rubrum*	9e-12	1, 43
Singlet1028	FE527234	0	0	0	0	0	0	0	0	0	1	ref|XP_001217348.1|	lipoic acid synthetase, mitochondrial precursor	*Aspergillus terreus NIH2624*	1e-46	1, 16
Singlet1029	FE527235	0	0	0	0	0	0	0	0	0	1	-	No significant similarity	-	-	-
Singlet1030	FE527240	0	0	0	0	0	0	0	0	0	1	ref|XP_001542349.1|	nuclear protein SNF4	*Ajellomyces capsulatus NAm1*	1e-20	1, 11, 18, 32, 42
Singlet1031	FE527241	0	0	0	0	0	0	0	0	0	1	sp|Q0CA25|FYV10_ASPTN	Protein fyv10	*Aspergillus terreus NIH2624*	6e-31	99
Singlet1032	FE527242	0	0	0	0	0	0	0	0	0	1	-	No significant similarity	-	-	-
Singlet1033	FE527243	0	0	0	0	0	0	0	0	0	1	-	No significant similarity	-	-	-
Singlet1034	FE527247	0	0	0	0	0	0	0	0	0	1	ref|NP_487763.1|	cation-transporting P-type ATPase	*Nostoc sp. PCC 7120*	7e-04	1, 16, 20
Singlet1035	FE527248	0	0	0	0	0	0	0	0	0	1	ref|XP_001268985.1|	conserved hypothetical protein	*Aspergillus clavatus NRRL 1*	9e-12	99
Singlet1036	FE527249	0	0	0	0	0	0	0	0	0	1	gb|AAO47089.1|	mannitol-1-phosphate dehydrogenase	*Paracoccidioides brasiliensis*	1e-42	1, 20
Singlet1037	FE527250	0	0	0	0	0	0	0	0	0	1	-	No significant similarity	-	-	-
Singlet1038	FE527253	0	0	0	0	0	0	0	0	0	1	ref|XP_001248046.1|	casein kinase I homolog	*Coccidioides immitis RS*	6e-35	14, 20, 32, 40, 43
Singlet1039	FE527254	0	0	0	0	0	0	0	0	0	1	-	No significant similarity	-	-	
Singlet1040	FE527257	0	0	0	0	0	0	0	0	0	1	ref|XP_001542533.1|	40S ribosomal protein S15	*Ajellomyces capsulatus NAm1*	4e-25	12
Singlet1041	FE527258	0	0	0	0	0	0	0	0	0	1	ref|XP_001538877.1|	conserved hypothetical protein	*Ajellomyces capsulatus NAm1*	1e-17	-

Singlet1042	FE527259	0	0	0	0	0	0	0	0	0	1	ref|XP_001212080.1|	enolase	*Aspergillus terreus NIH2624*	4e-28	1, 2, 16
Singlet1043	FE527261	0	0	0	0	0	0	0	0	0	1	ref|XP_001543567.1|	lanosterol synthase	*Ajellomyces capsulatus NAm1*	4e-34	1
Singlet1044	FE527262	0	0	0	0	0	0	0	0	0	1	-	No significant similarity	-	-	-
Singlet1045	FE527263	0	0	0	0	0	0	0	0	0	1	ref|XP_001266272.1|	histone acetylase complex subunit Paf400, putative	*Neosartorya fischeri NRRL 181*	1e-43	10, 11, 14, 42
Singlet1046	FE527265	0	0	0	0	0	0	0	0	0	1	-	No significant similarity	-	-	-
Singlet1047	FE527266	0	0	0	0	0	0	0	0	0	1	ref|XP_001261041.1|	calcium dependent mitochondrial carrier protein, putative	*Neosartorya fischeri NRRL 181*	2e-25	16. 20
Singlet1048	FE527267	0	0	0	0	0	0	0	0	0	1	ref|XP_001274394.1|	nucleoside diphosphatase (Ynd1), putative	*Aspergillus clavatus NRRL 1*	6e-10	1
Singlet1049	FE527268	0	0	0	0	0	0	0	0	0	1	ref|NP_830817.1|	Oxalate decarboxylase	*Bacillus cereus ATCC 14579*	3e-20	1, 2, 32
Singlet1050	FE527269	0	0	0	0	0	0	0	0	0	1	gb|EAA29158.2|	predicted protein	*Neurospora crassa OR74A*	1e-14	99
Singlet1051	FE527272	0	0	0	0	0	0	0	0	0	1	ref|XP_001263556.1|	Coatomer subunit alpha, putative	*Neosartorya fischeri NRRL 181*	2e-07	14, 16, 20, 42
Singlet1052	FE527273	0	0	0	0	0	0	0	0	0	1	emb|CAC28076.1|	glucoamylase	*Talaromyces emersonii*	2e-29	1
Singlet1053	FE527274	0	0	0	0	0	0	0	0	0	1	-	No significant similarity	-	-	-
Singlet1054	FE527275	0	0	0	0	0	0	0	0	0	1	ref|XP_001275305.1|	actin cytoskeleton protein (VIP1), putative	*Aspergillus clavatus NRRL 1*	8e-21	12, 42, 43
Singlet1055	FE527277	0	0	0	0	0	0	0	0	0	1	-	No significant similarity	-	-	-
Singlet1056	FE527282	0	0	0	0	0	0	0	0	0	1	dbj|BAF48663.1|	leucine-rich repeat/extensin	*Nicotiana plumbaginifolia*	1e-04	99
Singlet1057	FE527283	0	0	0	0	0	0	0	0	0	1	ref|ZP_01617781.1|	glutathione-dependent formaldehyde-activating, GFA	*marine gamma proteobacterium HTCC2143*	8e-05	99
Singlet1058	FE527284	0	0	0	0	0	0	0	0	0	1	ref|XP_001263728.1|	phosphopantothenate-cysteine ligase, putative	*Neosartorya fischeri NRRL 181*	3e-19	99
Singlet1059	FE527285	0	0	0	0	0	0	0	0	0	1	-	No significant similarity	-	-	-
Singlet1060	FE527287	0	0	0	0	0	0	0	0	0	1	-	No significant similarity	-	-	-
Singlet1061	FE527288	0	0	0	0	0	0	0	0	0	1	-	No significant similarity	-	-	-
Singlet1062	FE527289	0	0	0	0	0	0	0	0	0	1	ref|XP_001262431.1|	phthalate transporter, putative	*Neosartorya fischeri NRRL 181*	3e-22	20
Singlet1063	FE527290	0	0	0	0	0	0	0	0	0	1	gb|ABY21304.1|	thioredoxin TrxA	*Trichophyton mentagrophytes*	9e-41	10, 14, 20, 32, 42
Singlet1064	FE527292	0	0	0	0	0	0	0	0	0	1	ref|XP_001263032.1|	HORMA domain protein	*Neosartorya fischeri NRRL 181*	3e-18	99
Singlet1065	FE527295	0	0	0	0	0	0	0	0	0	1	-	No significant similarity	-	-	-
Singlet1066	FE527296	0	0	0	0	0	0	0	0	0	1	-	No significant similarity	-	-	-
Singlet1067	FE527297	0	0	0	0	0	0	0	0	0	1	-	No significant similarity	-	-	-

Singlet1068	FE527298	0	0	0	0	0	0	0	0	0	1	ref|XP_754442.2|	galactose-proton symport, putative	*Aspergillus fumigatus Af293*	7e-04	99
Singlet1069	FE527299	0	0	0	0	0	0	0	0	0	1	ref|XP_001262893.1|	DNA-directed RNA polymerases N/8 kDa subunit superfamily	*Neosartorya fischeri NRRL 181*	4e-35	1, 11, 16
Singlet1070	FE527300	0	0	0	0	0	0	0	0	0	1	-	No significant similarity	-	-	-
Singlet1071	FE527301	0	0	0	0	0	0	0	0	0	1	ref|XP_750553.1|	endoplasmic reticulum DnaJ domain protein Erj5, putative	*Aspergillus fumigatus Af293*	9e-21	14
Singlet1072	FE527303	0	0	0	0	0	0	0	0	0	1	-	No significant similarity	-	-	-
Singlet1073	FE527304	0	0	0	0	0	0	0	0	0	1	ref|XP_001227059.1|	hypothetical protein CHGG_09132	*Chaetomium globosum CBS 148.51*	4e-34	-
Singlet1074	FE527305	0	0	0	0	0	0	0	0	0	1	gb|AAN87885.1|	NADH-ubiquinone oxidoreductase	*Paracoccidioides brasiliensis*	3e-25	2, 20
Singlet1075	FE527307	0	0	0	0	0	0	0	0	0	1	-	No significant similarity	-	-	-
Singlet1076	FE527308	0	0	0	0	0	0	0	0	0	1	-	No significant similarity	-	-	-
Singlet1077	FE527309	0	0	0	0	0	0	0	0	0	1	ref|XP_001214203.1|	conserved hypothetical protein	*Aspergillus terreus NIH2624*	6e-25	99
Singlet1078	FE527310	0	0	0	0	0	0	0	0	0	1	-	No significant similarity	-	-	-
Singlet1079	FE527311	0	0	0	0	0	0	0	0	0	1	ref|XP_001213878.1|	homoserine dehydrogenase	*Aspergillus terreus NIH2624*	8e-52	1
Singlet1080	FE527314	0	0	0	0	0	0	0	0	0	1	ref|XP_001247458.1|	78 kDa glucose-regulated protein homolog precursor	*Coccidioides immitis RS*	3e-73	1, 14, 16
Singlet1081	FE527315	0	0	0	0	0	0	0	0	0	1	ref|XP_001541705.1|	hydroxymethylglutaryl-CoA lyase, mitochondrial precursor	*Ajellomyces capsulatus NAm1*	5e-50	1
Singlet1082	FE527316	0	0	0	0	0	0	0	0	0	1	-	No significant similarity	-	-	-
Singlet1083	FE527317	0	0	0	0	0	0	0	0	0	1	-	No significant similarity	-	-	-
Singlet1084	FE527318	0	0	0	0	0	0	0	0	0	1	ref|XP_001261075.1|	C-x8-C-x5-C-x3-H type zinc finger protein	*Neosartorya fischeri NRRL 181*	7e-41	11
Singlet1085	FE527320	0	0	0	0	0	0	0	0	0	1	-	No significant similarity	-	-	-
Singlet1086	FE527321	0	0	0	0	0	0	0	0	0	1	-	No significant similarity	-	-	-
Singlet1087	FE527323	0	0	0	0	0	0	0	0	0	1	sp|Q0CMM5|DBP4_ASPTN	ATP-dependent RNA helicase dbp4	*Aspergillus terreus NIH2624*	4e-31	10, 11, 12, 16, 20
Singlet1088	FE527326	0	0	0	0	0	0	0	0	0	1	ref|XP_001266180.1|	thioredoxin reductase, putative	*Neosartorya fischeri NRRL 181*	4e-24	1, 16, 32
Singlet1089	FE527327	0	0	0	0	0	0	0	0	0	1	-	No significant similarity	-	-	-
Singlet1090	FE527329	0	0	0	0	0	0	0	0	0	1	ref|XP_001672380.1|	Hypothetical protein CBG01536	*Caenorhabditis briggsae*	5e-21	99
Singlet1091	FE527335	0	0	0	0	0	0	0	0	0	1	ref|XP_001270181.1|	sodium P-type ATPase, putative	*Aspergillus clavatus NRRL 1*	9e-20	20, 32, 34
Singlet1092	FE527336	0	0	0	0	0	0	0	0	0	1	ref|XP_001543624.1|	NADH-ubiquinone oxidoreductase 9.5 kDa subunit	*Ajellomyces capsulatus NAm1*	2e-13	2, 20

MIPS: 1. Metabolism; 2. Energy; 10. Cell cycle and DNA processing; 11. Transcription; 12. Protein synthesis; 14. Protein fate (folding, modification, destination); 16. Protein with binding function or cofactor requirement (structural or catalytic); 18. regulation of metabolism or protein function; 20. Cellular transport, transport facilities and transport routes; 30. Cellular communication/signal transduction mechanism. 32. Cell rescue, defense and virulence; 34. Interaction with the environment; 38. Transposable elements, viral and plasmids proteins; 40. Cell fate; 41, Development (systemic); 42. Biogenesis of cellular components; 43. Cell type differentiation; 99. Unclassified proteins.
